# Zinc chloride-catalyzed cyclizative 1,2-rearrangement enables facile access to morpholinones bearing aza-quaternary carbons

**DOI:** 10.1038/s42004-023-01016-y

**Published:** 2023-10-07

**Authors:** Xing-Zi Li, Yu-Ping He, Hua Wu

**Affiliations:** Shanghai Frontiers Science Center for Drug Target Identification and Delivery, and Shanghai Key Laboratory for Molecular Engineering of Chiral Drugs, School of Pharmaceutical Sciences, Shanghai Jiao Tong University, 800 Dongchuan Road, Minhang District, Shanghai, 200240 China

**Keywords:** Synthetic chemistry methodology, Stereochemistry, Reaction mechanisms

## Abstract

Morpholines and morpholinones are important building blocks in organic synthesis and pharmacophores in medicinal chemistry, however, C3-disubstituted morpholines/morpholinones are extremely difficult to access. Here we show the ZnCl_2_-catalyzed cyclizative 1,2-rearrangement for the efficient synthesis of morpholinones bearing aza-quaternary stereocenters. A series of structurally diverse C3-disubstituted morpholin-2-ones which are difficultly accessible by existing methods were efficiently constructed from readily available two achiral linear compounds. Notably, mechanistic studies reveal that this reaction proceeds via an unusual sequence of direct formal [4 + 2] heteroannulation regioselectively delivering specific α-iminium/imine hemiacetals followed by a 1,2-esters or amides shift process, which is different from the reported mechanism of the aza-benzilic ester rearrangements.

## Abstract (web summary)

C-substituted morpholines and morpholinones are important building blocks in organic synthesis and pharmacophores in medicinal chemistry, however, diverse C3-disubstituted morpholines/morpholinones remain difficult to access. Here, the authors develop a facile construction of C3-disubstituted morpholinone bearing an aza-quaternary center achieved by ZnCl_2_-catalyzed cyclizative 1,2-rearrangement.

## Introduction

**Fig. 1:**
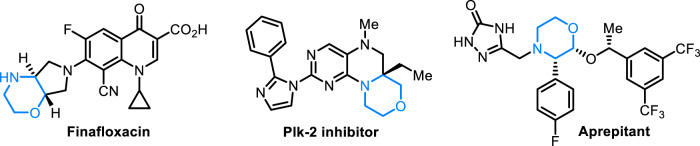
Selected examples of drugs containing chiral morpholine moieties. Chiral morpholines bearing aza-tertiary stereocenters or aza-quaternary stereocenters.

**Fig. 2:**
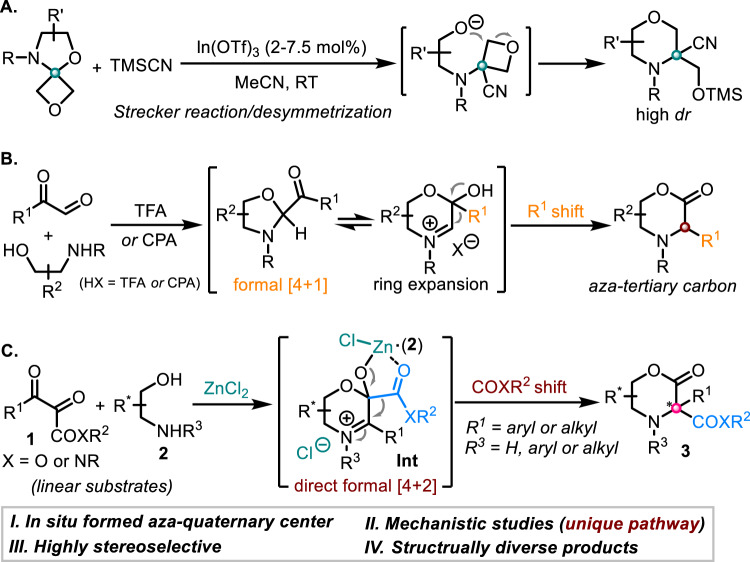
Catalytic synthesis of C3-substituted morpholines/morpholin-2-ones. **A** In(III)-catalyzed synthesis of morpholine containing an aza-quaternary center by Carreira. **B** Aza-benzilic ester rearrangement for the construction of aza-tertiary center of morpholinone. **C** Construction of morpholinone bearing aza-quaternary center by ZnCl_2_ catalysis (this work).

C-substituted morpholines and morpholinones represent important building blocks^1,2^ in organic synthesis and pharmacophores in medicinal chemistry, such as commercially available drugs of Finafloxacin^3^, Plk-2 inhibitor^4^ and Aprepitant^5^ (Fig. 1). Consequently, efficient access to these structural targets have been in great demand. Thus far, there have been a number of methods for the synthesis of C-functionalized morpholines or morpholinones^6–8^ benefiting from metal-catalyzed cyclization reactions^9–14^, allylic alkylation^15^, hydrogenations^16,17^ or other multi-step transformations^18–20^. On the contrary, approaches for the catalytic preparation of C3-disubstituted morpholines/morpholin-2-ones are far less developed, which is due probably to the steric hindrance effect of the incoming aza-quaternary carbon and availability of the corresponding branched starting materials^21–23^. Furthermore, asymmetric synthesis of such compounds was envisioned to be even more challenging, which must overcome the formidable challenges in both reaction reactivity and stereoselectivity. In this regard, an elegant work by Carreira and coworkers dealing with ring expansion of 3-oxetanone-derived spirocycles under In(III) catalysis is noteworthy (Fig. 2A)^24^. Mechanistically, the newly formed aza-quaternary carbon results from the Strecker reaction^25^ of oxazolidine with trimethylsilyl cyanide, and a subsequent stereoselective desymmetrizing 6-*exo*-tet cyclization finally affords morpholine in high dr value. To the best of our knowledge, so far, this is the only example of asymmetric synthesis of morpholine derivative with an aza-quaternary carbon center which is in situ formed rather than originates from a substrate. Thus, the highly useful and efficient approaches for the de novo construction of structurally diverse C3-disubstituted morpholines/morpholin-2-ones from readily available linear compounds are highly desirable.

Condensation of arylglyoxals with vicinal amino alcohols delivering 2-acyloxazolidines which further isomerize to morpholinones is well-known^26–30^. Recently, this reaction has been applied to the diastereoselective synthesis of morpholinones by Walczak group^31^. Very recently, we demonstrated an enantioselective aza-benzilic ester rearrangement for the synthesis of enantioenriched morpholinones by chiral Brønsted acid (Fig. 2B)^32^. In both cases, however, only morpholinones bearing aza-tertiary stereocenters have been successfully synthesized while all efforts aimed at the construction of C3-disubstituted morpholin-2-ones afford either oxazolidine intermediates or degradation at higher temperature^31,32^. To the best of our knowledge, 1,2-rearrangement for the construction of morpholinones bearing aza-quaternary stereocenters has not been achieved in spite of the significant synthetic importance of the resulting compounds^33,34^. In connection with our ongoing research interest in developing catalytic asymmetric cyclizative rearrangement (CACR)^32,35^, we herein report the successful realization of this endeavor, which delivers a wide range of C3-disubstituted morpholin-2-ones **3** from readily available linear 2,3-diketoesters **1** and vicinal amino alcohols **2** under the catalysis of ZnCl_2_ complex (Fig. 2C). Moreover, a highly diastereoselective version of such transformation has also been achieved by employing chiral vicinal amino alcohols as reaction partners. More importantly, rather than previously reported formal [4 + 1] oxazolidination/ring-expansive hemiacetalization/1,2-aryl or alkyl shift cascade (Fig. 2B)^32,35^, mechanistic investigations indicated that this transformation proceeded via a completely different reaction pathway consisting of an initial direct [4 + 2] cyclization (**Int**) followed by a 1,2-ester/amide shift process (Fig. 2C)^36^.

## Results

### Reaction development

With our on-going efforts to further examine its reactivity profile^35,36^, the multi-functionalizable vicinal tricarbonyl compounds^37–47^ were chosen as the reaction partner with aminoethanols. Our studies commenced with a mixture of ethyl 2,3-dioxo-3-phenylpropanoate and its hydrate (**1a**, 1.0 equiv), 2-((4-methoxyphenyl)amino)ethan-1-ol **2a** (1.2 equiv) in 1,2-dichloroethane (*c* 0.1 M) at 80 °C under argon. Initially, screening of various strong Brønsted acids, such as trifluoroacetic acid, trifluoromethanesulfonic acid, and *p*-toluenesulfonic acid, failed to give the rearranged products (entries 1-3, Table 1). Additionally, even aqueous hydrochloric acid could not give the desired product. As typical bifunctional catalysts, chiral phosphoric acids (CPA)^48,49^ could not give the rearranged product **3a** as well. Boron trifluoride-diethyl etherate (BF_3_ ∙ Et_2_O) was also ineffective (entry 4). Screening of a series of strong Lewis acids, such as Mg(OTf)_2_, InCl_3_, and Zn(II) complexes (entries 5-10), in the presence of tricarbonyl compound **1a** and amino alcohol **2a** at 80 °C resulted in the formation of the desired C3-disubstituted morpholin-2-one **3a**, and ZnCl_2_ gave the best result (55% yield). Using ZnCl_2_ as catalyst, the reaction conditions were further optimized by varying the solvent (entries 11–13), catalyst loading (entry 14), and reaction temperature (entries 15–16). Overall, the best conditions consisted of performing the reaction in DCE (*c* 0.1 M) at 100 °C in the presence of ZnCl_2_ (0.2 equiv). Under these conditions, C3-disubstituted morpholin-2-one **3a** was isolated in 61% yield (entry 15).

**Table 1:** Optimization of the reaction conditions.^[a]^.


Entry	Cat.	Solvent	T (°C)	Yield (%)^[b]^
1	TFA	DCE	80	trace
2	TfOH	DCE	80	trace
3	*p*-TSA	DCE	80	trace
4	BF_3_·Et_2_O	DCE	80	trace
5	Mg(OTf)_2_	DCE	80	17
6	InCl_3_	DCE	80	30
7	ZnCl_2_	DCE	80	55
8	Zn(OTf)_2_	DCE	80	36
9	ZnBr_2_	DCE	80	53
10	ZnI_2_	DCE	80	41
11	ZnCl_2_	THF	80	trace
12	ZnCl_2_	CH_3_CN	80	26
13	ZnCl_2_	CHCl_3_	80	37
14	ZnCl_2_	DCE	80	45^[c]^
15	ZnCl_2_	DCE	100	61
16	ZnCl_2_	DCE	60	trace

^[a]^Conditions: **1a** (0.10 mmol), **2a** (0.12 mmol), Cat. (0.02 mmol), solvent (*c* 0.1 M), sealed tube, argon, 12 h.

^[b]^Isolated yields.

^[c]^ZnCl_2_ (10 mol%) was used.

*PMP* *p*-methoxyphenyl, *DCE* 1,2-dichloroethane.

### Substrate scope exploration

**Fig. 3:**
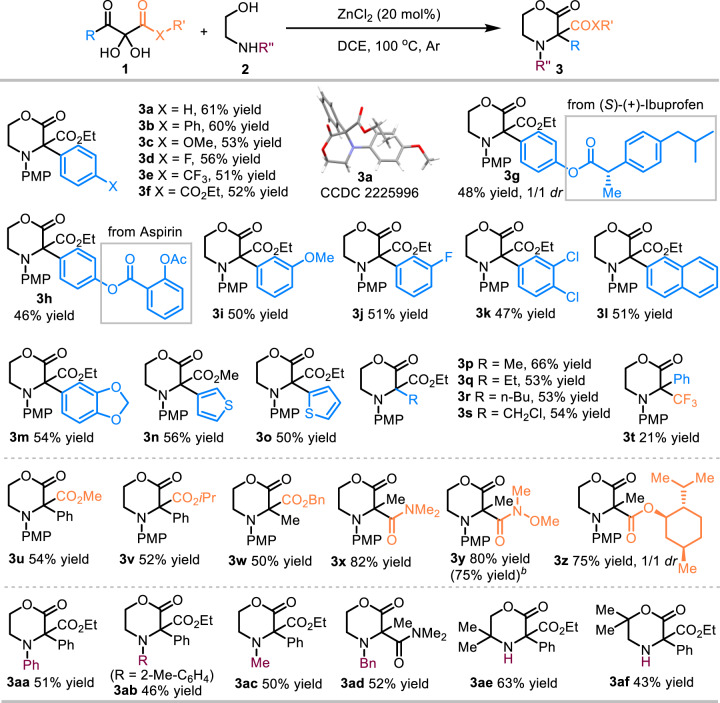
Substrate scope of vicinal tricarbonyl compounds and amino alcohols. [a] Conditions: **1** (0.10 mmol), **2** (0.12 mmol), ZnCl_2_ (0.02 mmol), DCE (*c* 0.1 M), sealed tube, argon, 12 h. [b] Yield of gram scale reaction.

With the optimized conditions in hand, this ZnCl_2_-catalyzed cyclizative 1,2-rearrangement was applicable to a wide range of substrates (Fig. 3). Regarding the aryl group of 3-aryl substituted 2,3-diketoesters, the presence of electron-neutral (H, Ph), electron-donating (MeO) groups and electron-withdrawing (F, CF_3_, CO_2_Et) at *para* and *meta* positions of the phenyl ring were well tolerated (**3a-3j**). Unfortunately, an aryl group bearing an *ortho* substituent failed to give the desired product which is due probably to the steric hinderance effect. The structure of compound **3a** was confirmed by X-ray crystallographic analysis (see Supplementary Data 1). In the presence catalytic amount of ZnCl_2_, (*S*)-(+)-Ibuprofen derived 2,3-diketoester could give the corresponding C3-disubstituted morpholin-2-one **3g** in moderate yield. Moreover, arylcoupled Aspirin could be smoothly functionalized with the present methodology (**3h**). 3,4-Dichlorophenyl, 2-naphthyl, and even heterocycles, such as benzo[*d*][1,3]dioxole, 3-thiophene and 2-thiophene, were transferred without event (**3k**-**3o**). To our delight, with aliphatic 2,3-diketoesters (**1p-1s**), these rearrangement reactions efficiently afforded the corresponding product **3p-3s** as well. Interestingly, instead of vicinal tricarbonyl compounds, 1,2-diketone bearing CF_3_ group (**1t**)^50^ could also give the desired rearranged product (**3t**) with decreased yield. Furthermore, 2,3-diketoesters derived from different alcohols, such as methyl, isopropyl, and benzyl, were all well-accepted substrates (**3u-3w**). Gratefully, 2,3-diketoamides participated in this ZnCl_2_-catalyzed cyclizative 1,2-rearrangement to provide the corresponding rearranged products **3x** and **3y** in high yields. Performing the gram scale reactions of **1y** (6.78 mmol) and **2a** (8.14 mmol) in the presence of ZnCl_2_ (20 mol%) afforded **3y** without obvious erosion of yield (75%). L-(-)-Menthol derived 2,3-diketoesters gave the corresponding product **3z** in good yield as well. Alternatively, other *N*-substituted amino alcohols bearing phenyl, 2-methylphenyl, methyl and benzyl groups were also examined, affording the corresponding products **3aa**-**3ad** without event. Notably, amino alcohol bearing electron-withdrawing protecting group on nitrogen, such as Ts and Ns, failed to afford the desired product **3**. Furthermore, *N*-unsubstituted amino alcohols could give the corresponding rearranged products (**3ae**-**3af**) smoothly.

### Strategy extension

**Fig. 4:**
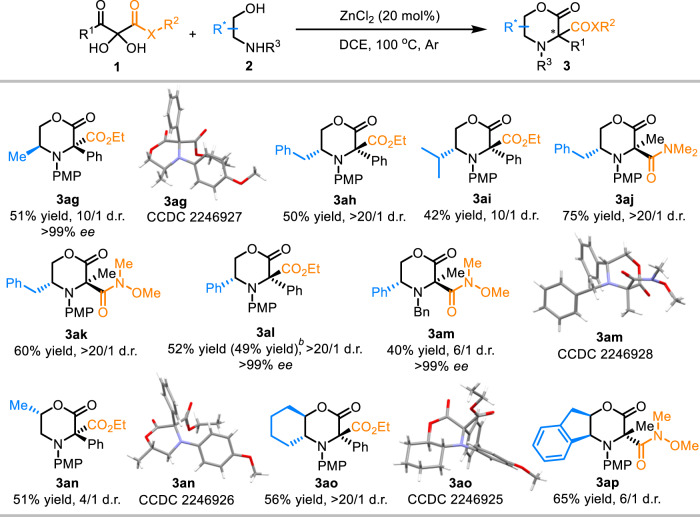
Asymmetric synthesis of morpholin-2-ones with multi-stereocenters.^[a]^ [a] Conditions: **1** (0.10 mmol), **2** (0.12 mmol), ZnCl_2_ (0.02 mmol), DCE (*c* 0.1 M), sealed tube, argon, 12 h. [b] Yield of 1.0 mmol scale.

Asymmetric catalytic construction of multi-functionalized morpholin-2-ones bearing aza-quaternary stereocenters represents a formidable challenge. To further exploit the structural diversity of the products, we divert our attention to the diastereoselective synthesis of C3-disubstituted morpholin-2-ones by employing optically pure *N*-substituted 2-aminoethan-1-ols. Gratefully, without any modifications to the reaction conditions, this protocol was applicable to a wide range of chiral amino alcohols delivering structurally diverse products bearing multi-stereocenters in high stereoselectivities (Fig. 4). Various *N-*substituted 2-aminoethan-1-ol, such as (*S*)-2-aminopropan-1-ol, (*R*)-2-amino-3-phenylpropan-1-ol and (*R*)-2-amino-3-methylbutan-1-ol, underwent this rearrangement reaction smoothly affording the desired products **3ag-3ai** in high diastereoselectivities. 2,3-Diketoamides **1aj** and **1ak** were also well tolerated leading to the formation of the products **3aj** and **3ak** in good yields with excellent stereoselectivities (>20/1 d.r.). Moreover, C2-phenyl substituted amino alcohol **1al** afforded the product **3al** in excellent d.r. value as well. *N*-benzyl substituted amino alcohol **1am** delivered decreased stereoselectivity (6/1 d.r.). Subsequently, Other more challenging chiral amino alcohols were further examined. Interestingly, even *N*-PMP substituted (*S*)-1-aminopropan-2-ol **1an** where the original stereocenter was far away from the newly formed aza-quaternary carbon could also afford the product **3an** in good diastereoselectivity (4/1 d.r.). Substrates **1ao** and **1ap** derived from cyclic amino alcohols underwent the cyclizative 1,2-rearrangement without event, which furnished the corresponding products (**3ao**-**3ap**) bearing three stereocenters in excellent and good diastereoselectivities. The absolute configurations of the products **3ag,** **3am,** **3an** and **3ao** were determined to be (3*S*,5*S*), (3*R*,5*R*), (3*S*,6*S*), and (3 *R*,4a*R*,8a*R*), respectively (see Supplementary Data 2–5). Consequently, the absolute configurations for other morpholinones (**3ah-3al** and **3ap**) were assigned accordingly (Fig. 4). Unfortunately, all efforts aimed at developing a catalytic enantioselective version of this rearrangement gave almost racemic products (see Supplementary Table S1 for details).

### Mechanistic studies

**Fig. 5:**
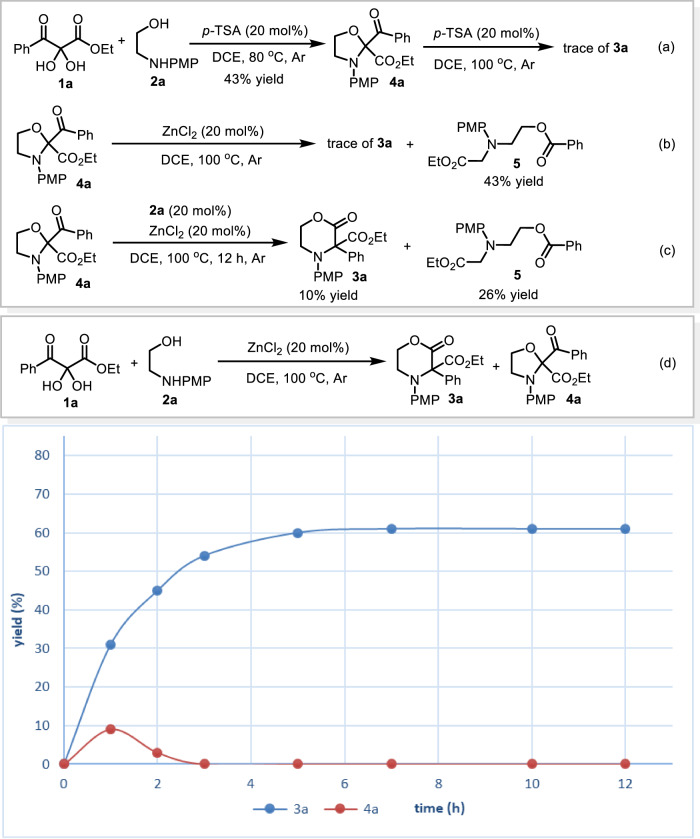
Control experiments. **a**–**c** Conversion of **4a** catalyzed by *p*-TSA, ZnCl_2_ and (**2a**)·ZnCl_2_, respectively. **d** Kinetic curve for the formation of **3a** and **4a**.

**Fig. 6:**
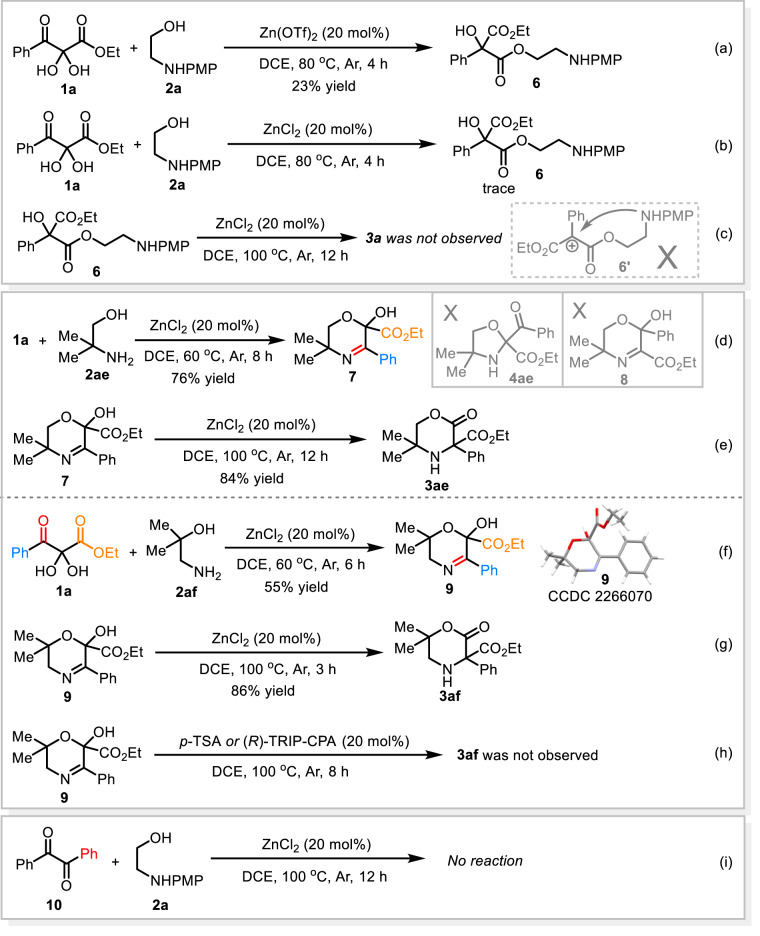
Other control experiments. **a**, **b** Reaction of **1a** and **2a** catalyzed by Zn(OTf)_2_ and ZnCl_2_, respectively. **c** Conversion of **6** catalyzed by ZnCl_2_. **d**, **e** Reaction of **1a** and **2ae**. **f**–**h** Reaction of **1a** and **2af**. **i** Reaction of **10** and **2a** catalyzed by ZnCl_2_.

A series of control experiments were performed to gain insight into the reaction mechanism. In the presence of *p*-toluenesulfonic acid (*p*-TSA), a formal [3 + 2] reaction of **1a** with **2a** occurred at 80 °C affording an oxazolidine compound **4a** in moderate yield (Fig. 5a). Further treating intermediate **4a** with catalytic amount of *p*-TSA (20 mol%) gave trace of the product **3a** (Fig. 5a). To our surprise, however, resubmitting **4a** to the standard reaction conditions also afforded only trace of the rearranged product **3a** with concurrent formation of a linear compound **5** in 43% yield and some decomposition species (Fig. 5b). As 2-aminoethan-1-ol derivatives **2** are capable of serving as bidentate ligands to Zn(II)^51^, we further examine the reactivity of (**2a**)∙ZnCl_2_ complex. Under the catalysis of the in situ formed (**2a**)∙ZnCl_2_ complex, the reaction of the intermediate **4a** gave product **3a** in very low yield (10%) and a linear compound **5** was formed as a main product (Fig. 5c). Alternatively, a set of experiments were performed with 2,3-diketoester **1a** and amino alcohol **2a** under the standard conditions and the yields of both **3a** and **4a** were detected at different time. As it is seen clearly from the diagram shown in Fig. 5d, the yield of **3a** increased steadily as the reaction proceeded with the concurrent formation of the low yield of **4a**. These results revealed that, in this case, oxazolidine intermediate **4a** resulted from formal [4 + 1] condensation of **1a** and **2a** is not a major reservoir for product **3a**, which is different from the reported Brønsted acid catalyzed aza-benzilic ester rearrangements^31,32^. Notably, during the screening of the reaction conditions, another benzilic ester rearrangement product **6**^36^ was observed in the presence of catalytic amount of Zn(OTf)_2_ (Fig. 6a). However, ZnCl_2_ complex only gave a trace of compound **6** (Fig. 6b). Furthermore, resubmitting **6** to our standard reaction conditions failed to give the desired product **3a** (Fig. 6c). These results indicate that an intramolecular aza-cyclization to the final product **3a** via a possible in situ formed carbocation intermediate **6’** derived from dehydration of **6** could be excluded. Subsequently, we realized that, at the first step of this tandem reaction, a direct formal [4 + 2] reaction might be competitive to the [4 + 1] cyclization of **1a** and **2a**. All our efforts aimed at trapping the key intermediate of the rearrangement by employing various excessive nucleophilic reagents failed to give the corresponding products. To our delight, when the reaction of **1a** and *N*-unsubstituted amino alcohol **2ae** was performed at lower temperature (60 °C), a unique α-imine hemiacetal intermediate **7** was successfully isolated in good yield (Fig. 6d). Surprisingly, the imine moiety was formed from the condensation of primary amine with the carbonyl group linked to phenyl rather than with the middle one, which is also different from the previous reports^31,32,35^. Notably, no oxazolidine intermediate **4ae** was detected throughout the whole reaction process^26–30^. Interestingly, a predictable regioisomer **8** resulted from the formal [4 + 2] reaction was actually not observed as well, which is due probably to the relatively high thermodynamic stability of the α-imine hemiacetal **7**. Importantly, under the standard condition, the six-membered ring compound **7** underwent the rearrangement reaction smoothly, which delivered the corresponding product **3ae** in high yield (Fig. 6e). In the absence of ZnCl_2_ catalyst, intermediate **7** failed to give **3ae**. The reaction of **1a** with another branched amino alcohol **2af** gave similar results (Fig. 6f, g). The structure of the important α-imine hemiacetal **9** was further confirmed by X-ray single crystal diffraction technique (see Supplementary Data 6). These results clearly suggested that a six-membered ring intermediate resulting from a direct formal [4 + 2] cyclization should be the real reservoir for product **3**. Moreover, treating compound **9** with catalytic amount of either *p*-TSA or (*R*)-TRIP-CPA at 100 °C failed to yield the product **3af**, which not only underlined the great importance of ZnCl_2_ catalyst in triggering the key 1,2-ester shift step but also highlighted the difference in the reaction mechanism between current rearrangement and Brønsted acid-catalyzed benzilic ester-type rearrangement (Fig. 6h)^31,32,35^. Finally, the reaction of diphenyl-substituted 1,2-diketone **10** with the amino alcohol **2a** gave neither the rearranged product nor any intermediates (Fig. 6i), which indicated that the extra ester group is of vital importance for this rearrangement. For all NMR spectra of the new compounds, see Supplementary Data 7.

**Fig. 7:**
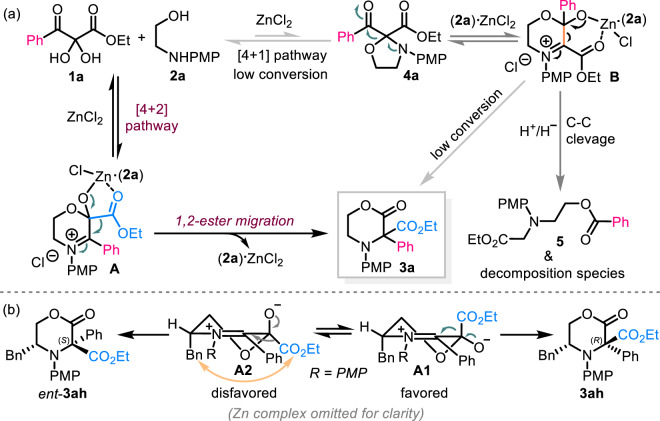
Proposed reaction mechanism and stereochemical pathway. **a** The reaction of **1a** and **2a** was employed to demonstrate the reaction mechanism. **b** The stereochemical pathway for the formation of **3ah** was shown.

As the conversion of **1** and **2** to the five-membered oxazolidine intermediates **4** is extremely low, and the transformation of **4** to the products **3** is also highly difficult (see Fig. 5b–d), the compound **4** should be not the preferred intermediates under the conditions. Moreover, the isolation of the only [4 + 2] cyclization products **7** and **9** which could be smoothly transformed into the rearranged products **3ae** and **3af** clearly indicates the real reaction pathway as well. Therefore, on the basis of the mechanistic experiments, this rearrangement reaction is proposed to proceed via an unprecedent direct [4 + 2] cyclization/1,2-ester or amide migration sequence (Fig. 7a). Initially, the 2,3-diketoester **1a** and amino alcohol **2a** predominately undergo a formal [4 + 2] heteroannulation reaction to generate a cyclic α-iminium hemiacetal **A**. Subsequently, the key intermediate **A** then participate in a 1,2-ester shift reaction under the catalysis of in situ formed (**2a**)∙ZnCl_2_ complex affording the final C3-disubstituted morpholin-2-one **3a**. The in situ formed a small amount of **4a** is more inclined to generate compound **5** rather than the rearrangement product **3a**, which is due probably to the driving force differences to these two compounds, respectively. The side product **5** might result from the carbon-carbon cleavage of intermediate **B** to produce a carbene species which is quenched by water and a potential hydride transfer reagent **2a**^52,53^.

A plausible pathway leading to the observed stereochemical outcome is also illustrated in Fig. 7b. Because the ester moiety of the intermediate **A1** is remote from benzyl group, the *Re* face of the iminium moiety is open to the ester group 1,2-migration, thus favorably affording **3ah**. In contrast, the *Si* face of the iminium moiety is shielded by benzyl group in **A2**, thus making the 1,2-ester shift disfavored and resulting in the formation of the enantiomer *ent*-**3ah** in a minor amount. This scenario is in accordance with the configuration of the products as established by X-ray crystal structure analysis of four representative morpholines (**3ag,** **3am,** **3an** and **3ao**, see Fig. 4).

In conclusion, we have developed the first examples of ZnCl_2_-catalyzed cyclizative 1,2-rearrangement for the asymmetric synthesis of morpholinones bearing aza-quaternary stereocenters which are far more challenging to obtain efficiently by existing methods. A series of structurally diverse C3-disubstituted morpholin-2-ones were efficiently constructed from readily available two achiral linear compounds. Importantly, on the basis of mechanistic studies, this rearrangement proceeds via an unprecedent sequence of direct formal [4 + 2] heteroannulation followed by a 1,2-ester or amide migration process, which is highly distinct from the reported reaction mechanism of aza-benzilic acid-type rearrangements.

## **Methods** (Supplementary Methods in the SI)

### General procedure for the synthesis of 3

To a mixture of **1** (0.1 mmol, 1.0 equiv), **2** (0.12 mmol, 1.2 equiv), and ZnCl_2_ (0.02 mmol, 0.2 equiv) was added 1,2-dichloroethane (1.0 mL) under argon. The reaction mixture was stirred at 100 °C for indicated hours. After completion of the reaction (monitored by TLC), the solvent was removed under vacuum. The residue was purified by column chromatography on silica gel, eluting with petroleum ether/ethyl acetate to afford the products **3** in moderate to high yields.

### Supplementary information

Supplementary Information

Description of Additional Supplementary Files

Supplementary Data 1

Supplementary Data 2

Supplementary Data 3

Supplementary Data 4

Supplementary Data 5

Supplementary Data 6

Supplementary Data 7

## Data Availability

Cif. files (see Supplementary Data 1–6); all NMR spectra of new compounds (see Supplementary Data 7). All data generated and analyzed during this study are included in this article, its Supplementary Information, and also available from the authors upon reasonable request. The X-ray crystallographic coordinates for structures reported in this Article have been deposited at the Cambridge Crystallographic Data Centre (CCDC), under deposition number CCDC 2225996, 2246927, 2246928, 2246926, 2246925 and 2266070 (see Supplementary Notes and Supplementary Tables S2-43 for details). These data can be obtained free of charge from The Cambridge Crystallographic Data Centre via www.ccdc.cam.ac.uk/data request/cif.
