# Supplementary material for: Zinc chloride-catalyzed cyclizative 1,2-rearrangement enables facile access to morpholinones bearing aza-quaternary carbons
Source: Commun Chem. 2023 Oct 7;6:216. doi: 10.1038/s42004-023-01016-y (PMC10560277; doi:10.1038/s42004-023-01016-y)
Supplement: Supplementary file 1 — Supplementary Information [file 42004_2023_1016_MOESM1_ESM.pdf]

Supporting Information

Zinc chloride-catalyzed cyclizative 1,2-rearrangement enables facile access to morpholinones bearing aza-quaternary carbons

Xing-Zi Li, Yu-Ping He, Hua Wu*

Shanghai Frontiers Science Center for Drug Target Identification and Delivery, and Shanghai Key Laboratory for Molecular Engineering of Chiral Drugs, School of Pharmaceutical Sciences, Shanghai Jiao Tong University, 800 Dongchuan Road, Minhang District, Shanghai 200240, China.

Email: hua.wu@sjtu.edu.cn

Table of Contents

1. General information	S1
2. Synthesis and characterization data of **2**	S2
3. General procedure for the synthesis of **3**	S4
4. Synthesis and characterization data of **3**	S4
5. Gram-scale reaction	S23
6. Chiral ligands effect	S24
7. Control experiments	S25
8. Supplementary References	S28
9. Crystallographic data for **3a**	S29
10. Crystallographic data for **3ag**	S36
11. Crystallographic data for **3am**	S44
12. Crystallographic data for **3an**	S52
13. Crystallographic data for **3ao**	S60
14. Crystallographic data for **9**	S72
15. HPLC chromatograms	S78

1. General information

NMR spectra were recorded on Bruker AV 400 MHz spectrometer. Chemical shifts are given in ppm. The spectra are calibrated to the residual ^{1}H and ^{13}C signals of the solvents. Multiplicities are abbreviated as follows: singlet (s), doublet (d), triplet (t), quartet (q), doublet-doublet (dd), quintet (quint), sextet (sext), septet (sept), multiplet (m), and broad (b), doublet of triplets (dt), triplet of doublets (td). Mass spectrometry analysis was carried out using an electrospray spectrometer Waters Micromass Q-TOF Premier Mass Spectrometer. Melting points were measured with SGW X-4 micro melting point apparatus. Optical rotations were measured on a Rudolph Research Analytical Autopol VI automatic polarimeter using a 50 mm path-length cell at 589 nm.

Materials and Methods:

Unless otherwise stated, starting materials were purchased from commercial sources (Adamas-Beta®, Shanghai Haohong Scientific Co., Ltd., Shanghai Bide pharmatech Co., Ltd. J&K®, Aladdin® and Energy chemical), and used without further purification. Sensitive compounds were stored in a desiccator or in a glove box if required. Solvents were purchased in HPLC quality, degassed by purging thoroughly with nitrogen and dried over activated molecular sieves of appropriate size. Alternatively, they were purged with argon and passed through alumina columns in a solvent purification system (Innovative Technology). $ZnBr_2$ was supplied by Shanghai Haohong Scientific Co., Ltd, the $ZnCl_2$ was supplied by Sigma-Aldrich Co. LLC. Reactions were monitored by thin layer chromatography (TLC) using Xinnuo TLC silica gel 60 F254. Compounds were visualized by UV-light at 254 nm and by dipping the plates in an ethanolic vanillin/ sulfuric acid solution or an aqueous potassium permanganate solution followed by heating. Flash column chromatography was performed over silica gel (300-400 mesh). The $CDCl_3$ used in the NMR experiments was stored over anhydrous K_2CO_3 before use. All the substrates **1** were synthesized according to the reported procedure.[1-3]

2. Synthesis and characterization data of 2

All the substrates **2** were synthesized according to the reported procedure.[4]

(*R*)-2-((4-methoxyphenyl)amino)-3-phenylpropan-1-ol (2ah)

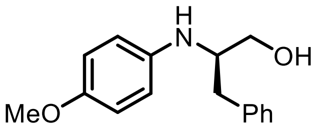

Brown solid, m.p = 60 – 70 °C.

^{1}H NMR (400 MHz, Chloroform-*d*) δ 7.33 – 7.28 (m, 2H), 7.26 – 7.16 (m, 3H), 6.81 (d, *J* = 8.9 Hz, 2H), 6.67 (d, *J* = 8.9 Hz, 2H), 3.77 (s, 3H), 3.73 – 3.63 (m, 2H), 3.47 (dd, *J* = 10.4, 5.4 Hz, 1H), 2.93 (dd, *J* = 13.6, 5.0 Hz, 1H), 2.81 (dd, *J* = 13.6, 7.4 Hz, 1H).

^{13}C NMR (101 MHz, Chloroform-*d*) δ 152.7, 141.2, 138.0, 129.4, 128.7, 126.6, 115.9, 115.1, 63.3, 57.3, 55.8, 37.5.

HRMS (ESI) m/z: $[M + H]^+$ Calcd for $C_{16}H_{20}NO_2^+$ 258.1489; Found 258.1492.

(*R*)-2-((4-methoxyphenyl)amino)-3-methylbutan-1-ol (2ai)

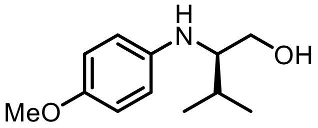

Brown oil.

^{1}H NMR (400 MHz, Chloroform-*d*) δ 6.77 (d, *J* = 8.9 Hz, 2H), 6.65 (d, *J* = 8.9 Hz, 2H), 3.77 – 3.71 (m, 4H), 3.47 (dd, *J* = 10.9, 7.5 Hz, 1H), 3.22 – 3.17 (m, 1H), 1.90 – 1.80 (m, 1H), 0.96 (d, *J* = 6.9 Hz, 3H), 0.92 (d, *J* = 6.8 Hz, 3H).

^{13}C NMR (101 MHz, Chloroform-*d*) δ 152.4, 142.6, 115.5, 115.0, 62.6, 62.4, 55.9, 30.3, 19.4, 19.0.

HRMS (ESI) m/z: $[M + H]^+$ Calcd for $C_{12}H_{20}NO_2^+$ 210.1489; Found 210.1487.

(1*R*,2*R*)-2-((4-methoxyphenyl)amino)cyclohexan-1-ol (2ao)

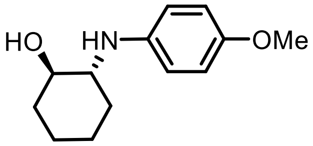

White solid, m.p = 97 – 99 °C.

^{1}H NMR (400 MHz, Chloroform-*d*) δ 6.78 (d, *J* = 9.0 Hz, 2H), 6.69 (d, *J* = 8.9 Hz, 2H), 3.75 (s, 3H), 3.36 – 3.28 (m, 1H), 3.04 – 2.96 (m, 3H), 2.15 – 2.04 (m, 2H), 1.80 – 1.66 (m, 2H), 1.44 – 1.19 (m, 3H), 1.06 – 0.94 (m, 1H).

^{13}C NMR (101 MHz, Chloroform-*d*) δ 153.0, 141.7, 116.5, 114.9, 74.5, 61.8, 55.9, 33.2, 31.7, 25.2, 24.4.

HRMS (ESI) m/z: $[M + H]^+$ Calcd for $C_{13}H_{20}NO_2^+$ 222.1489; Found 222.1491.

(1*S*,2*R*)-1-((4-methoxyphenyl)amino)-2,3-dihydro-1H-inden-2-ol (2ap)

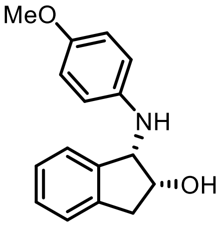

Brown oil.

^{1}H NMR (400 MHz, Chloroform-*d*) δ 7.32 – 7.19 (m, 4H), 6.83 (d, *J* = 9.1 Hz, 2H), 6.76 (d, *J* = 9.1 Hz, 2H), 4.75 (d, *J* = 4.9 Hz, 1H), 4.65 – 4.57 (m, 1H), 3.90 (brs, 1H), 3.76 (s, 3H), 3.13 (dd, *J* = 16.6, 5.1 Hz, 1H), 3.00 (dd, *J* = 16.6, 1.7 Hz, 1H), 2.91 (brs, 1H).

^{13}C NMR (101 MHz, Chloroform-*d*) δ 152.9, 141.8, 141.7, 140.6, 128.3, 127.1, 125.6, 124.1, 115.2, 115.1, 72.3, 64.0, 55.8, 39.6.

HRMS (ESI) m/z: $[M + Na]^+$ Calcd for $C_{16}H_{17}NNaO_2^+$ 278.1151; Found 278.1157.

Supplementary methods

3. Supplementary methods

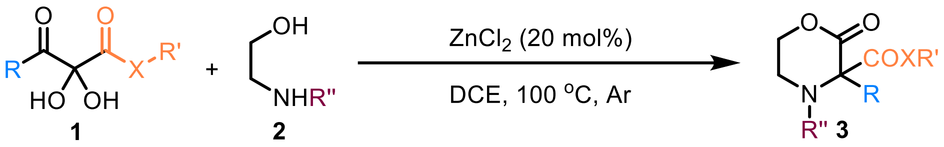

A suspension of **1** (0.1 mmol, 1.0 equiv), **2** (0.12 mmol, 1.2 equiv), $ZnCl_2$ (0.02 mmol, 0.2 equiv) in 1,2-dichloroethane (1.0 mL) was stirred in a dry sealed tube under argon. The reaction mixture was stirred at 100 °C for indicated hours. After completion of the reaction (monitored by TLC), the solvent was removed under vacuum. The residue was purified by column chromatography on silica gel eluting with petroleum ether/ethyl acetate to afford the products **3**.

Note: For substrate **1**, the ratio between hydrate form and tricarbonyl form was detected by ^{1}H NMR, and the molecular weight was calculated accordingly.

4. Synthesis and characterization data of 3

ethyl 4-(4-methoxyphenyl)-2-oxo-3-phenylmorpholine-3-carboxylate (3a)

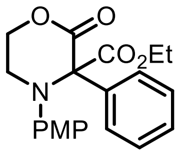

100 °C, 12 h, 21.7 mg, 61% yield, yellow solid, m.p = 134 – 136 °C.

^{1}H NMR (400 MHz, Chloroform-*d*) δ 7.89 – 7.81 (m, 2H), 7.46 – 7.36 (m, 3H), 6.71 (d, *J* = 8.7 Hz, 2H), 6.62 (d, *J* = 8.7 Hz, 2H), 4.62 – 4.53 (m, 1H), 4.52 – 4.46 (m, 1H), 4.00 – 3.87 (m, 3H), 3.77 – 3.72 (m, 1H), 3.71 (s, 3H), 0.86 (t, *J* = 7.2 Hz, 3H).

^{13}C NMR (101 MHz, Chloroform-*d*) δ 167.5, 167.0, 154.0, 140.8, 135.4, 129.5, 129.1, 128.4, 117.0, 114.3, 76.7, 66.4, 62.2, 55.7, 44.8, 13.7.

HRMS (ESI) m/z: $[M + Na]^+$ Calcd for $C_{20}H_{21}NNaO_5^+$ 378.1312; Found 378.1322.

ethyl 3-([1,1'-biphenyl]-4-yl)-4-(4-methoxyphenyl)-2-oxomorpholine-3-carboxylate (3b)

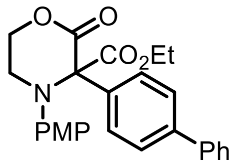

100 °C, 12 h, 25.7 mg, 60% yield, pale yellow foam.

^{1}H NMR (400 MHz, Chloroform-*d*) δ 7.93 (d, *J* = 8.6 Hz, 2H), 7.67 – 7.58 (m, 4H), 7.46 (t, *J* = 7.6 Hz, 2H), 7.40 – 7.34 (m, 1H), 6.74 (d, *J* = 9.2 Hz, 2H), 6.67 (d, *J* = 9.3 Hz, 2H), 4.63 (td, *J* = 11.4, 2.2 Hz, 1H), 4.52 (dt, *J* = 11.3, 2.7 Hz, 1H), 4.03 – 3.94 (m, 2H), 3.91 (dd, *J* = 11.7, 3.0 Hz, 1H), 3.80 – 3.74 (m, 1H), 3.72 (s, 3H), 0.88 (t, *J* = 7.1 Hz, 3H).

^{13}C NMR (101 MHz, Chloroform-*d*) δ 167.5, 166.9, 154.0, 141.9, 140.8, 140.6, 134.4, 129.9, 129.0, 127.7, 127.3, 127.2, 117.0, 114.4, 76.6, 66.5, 62.3, 55.7, 44.8, 13.7.

HRMS (ESI) m/z: $[M + Na]^+$ Calcd for $C_{26}H_{25}NNaO_5^+$ 454.1625; Found 454.1644.

ethyl 3,4-bis(4-methoxyphenyl)-2-oxomorpholine-3-carboxylate (3c)

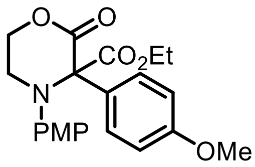

100 °C, 12 h. 20.4 mg, 53% yield, yellow oil.

^{1}H NMR (400 MHz, Chloroform-*d*) δ 7.75 (d, *J* = 9.1 Hz, 2H), 6.94 (d, *J* = 9.0 Hz, 2H), 6.71 (d, *J* = 9.2 Hz, 2H), 6.63 (d, *J* = 9.3 Hz, 2H), 4.57 (td, *J* = 11.4, 2.3 Hz, 1H), 4.49 (dt, *J* = 11.3, 2.6 Hz, 1H), 3.99 – 3.90 (m, 2H), 3.87 (dd, *J* = 11.7, 3.1 Hz, 1H), 3.84 (s, 3H), 3.75 – 3.72 (m, 1H), 3.71 (s, 3H), 0.85 (t, *J* = 7.1 Hz, 3H).

^{13}C NMR (101 MHz, Chloroform-*d*) δ 167.7, 167.2, 160.3, 153.9, 140.9, 130.8, 127.2, 116.9, 114.3, 113.8, 76.2, 66.4, 62.1, 55.7, 55.5, 44.8, 13.7.

HRMS (ESI) m/z: $[M + Na]^+$ Calcd for $C_{21}H_{23}NNaO_6^+$ 408.1418; Found 408.1425.

ethyl 3-(4-fluorophenyl)-4-(4-methoxyphenyl)-2-oxomorpholine-3-carboxylate (3d)

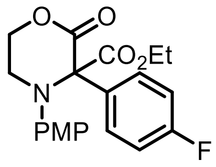

100 °C, 12 h. 20.9 mg, 56% yield, pale yellow foam.

^{1}H NMR (400 MHz, Chloroform-*d*) δ 7.87 – 7.80 (m, 2H), 7.11 (t, *J* = 8.6 Hz, 2H), 6.72 (d, *J* = 9.1 Hz, 2H), 6.61 (d, *J* = 9.2 Hz, 2H), 4.61 – 4.49 (m, 2H), 3.98 – 3.85 (m, 3H), 3.76 – 3.73 (m, 1H), 3.71 (s, 3H), 0.86 (t, *J* = 7.1 Hz, 3H).

^{13}C NMR (101 MHz, Chloroform-*d*) δ 167.4, 166.9, 163.3 (d, *J* = 248.8 Hz), 154.1, 140.6, 131.5 (d, *J* = 8.3 Hz), 131.2 (d, *J* = 3.1 Hz), 117.0, 115.4 (d, *J* = 21.6 Hz), 114.4, 76.2, 66.5, 62.4, 55.7, 44.7, 13.7.

^{19}F NMR (376 MHz, Chloroform-*d*) δ -113.3.

HRMS (ESI) m/z: $[M + Na]^+$ Calcd for $C_{20}H_{20}FNNaO_5^+$ 396.1218; Found 396.1216.

ethyl 4-(4-methoxyphenyl)-2-oxo-3-(4-(trifluoromethyl)phenyl)morpholine-3-carboxylate (3e)

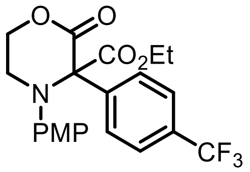

100 °C, 12 h. 21.5 mg, 51% yield, yellow solid, m.p = 133 – 135 °C.

^{1}H NMR (400 MHz, Chloroform-*d*) δ 8.01 (d, *J* = 8.2 Hz, 2H), 7.69 (d, *J* = 8.4 Hz, 2H), 6.73 (d, *J* = 9.2 Hz, 2H), 6.59 (d, *J* = 9.2 Hz, 2H), 4.58 – 4.52 (m, 2H), 4.00 – 3.88 (m, 3H), 3.76 (dt, *J* = 12.2, 2.4 Hz, 1H), 3.71 (s, 3H), 0.87 (t, *J* = 7.1 Hz, 3H).

^{13}C NMR (101 MHz, Chloroform-*d*) δ 167.0, 166.4, 154.3, 140.4, 139.6, 131.2 (q, *J* = 32.6 Hz), 130.1, 125.3 (q, *J* = 3.6 Hz), 124.1 (d, *J* = 273.2 Hz), 117.1, 114.4, 76.7, 66.6, 62.5, 55.7, 44.7, 13.7.

^{19}F NMR (376 MHz, Chloroform-*d*) δ -62.7.

HRMS (ESI) m/z: $[M + Na]^+$ Calcd for $C_{21}H_{20}F_3NNaO_5^+$ 446.1186; Found 446.1194.

ethyl 3-(4-(ethoxycarbonyl)phenyl)-4-(4-methoxyphenyl)-2-oxomorpholine-3-carboxylate (3f)

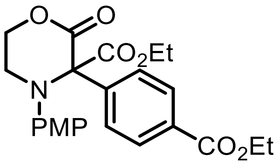

100 °C, 16 h. 22.2 mg, 52% yield, pale yellow foam.

^{1}H NMR (400 MHz, Chloroform-*d*) δ 8.09 (d, *J* = 8.7 Hz, 2H), 7.95 (d, *J* = 8.3 Hz, 2H), 6.71 (d, *J* = 9.3 Hz, 2H), 6.59 (d, *J* = 9.2 Hz, 2H), 4.60 – 4.50 (m, 2H), 4.40 (q, *J* = 7.1 Hz, 2H), 4.01 – 3.88 (m, 3H), 3.75 (dt, *J* = 12.2, 2.3 Hz, 1H), 3.71 (s, 3H), 1.40 (t, *J* = 7.1 Hz, 3H), 0.86 (t, *J* = 7.1 Hz, 3H).

^{13}C NMR (101 MHz, Chloroform-*d*) δ 167.0, 166.5, 166.3, 154.1, 140.5, 140.3, 131.1, 129.6, 129.5, 117.0, 114.3, 76.8, 66.6, 62.4, 61.3, 55.6, 44.7, 14.5, 13.7.

HRMS (ESI) m/z: $[M + Na]^+$ Calcd for $C_{23}H_{25}NNaO_7^+$ 450.1523; Found 450.1536.

ethyl 3-(4-(((S)-2-(4-isobutylphenyl)propanoyl)oxy)phenyl)-4-(4-methoxyphenyl)-2-oxomorpholine-3-carboxylate (3g)

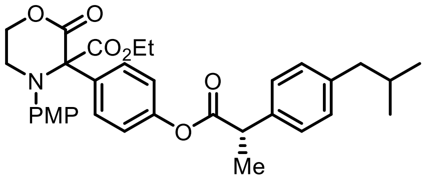

100 °C, 16 h. 26.7 mg, 48% yield, 1:1 d. r., yellow oil.

^{1}H NMR (400 MHz, Chloroform-*d*) δ (mixture of two diastereoisomers) 7.82 (d, *J* = 8.8 Hz, 4H), 7.30 (d, *J* = 8.1 Hz, 4H), 7.14 (d, *J* = 8.1 Hz, 4H), 7.07 (d, *J* = 8.9 Hz, 4H), 6.71 (d, *J* = 9.2 Hz, 2H), 6.70 (d, *J* = 2.0 Hz, 2H), 6.60 (d, *J* = 9.2 Hz, 2H), 6.59 (d, *J* = 9.2 Hz, 2H) 4.58 – 4.46 (m, 4H), 3.99 – 3.83 (m, 8H), 3.75 – 3.72 (m, 2H), 3.705 (s, 3H), 3.702 (s, 3H), 2.47 (d, *J* = 7.1 Hz, 4H), 1.93 – 1.80 (m, 2H), 1.61 (dd, *J* = 7.1, 1.0 Hz, 6H), 0.91 (d, *J* = 6.8 Hz, 12H), 0.84 (t, *J* = 7.1 Hz, 6H).

^{13}C NMR (101 MHz, Chloroform-*d*) δ (mixture of two diastereoisomers) 173.1, 167.3, 166.8, 154.0, 151.6, 141.0, 140.7, 137.2, 132.7, 130.7, 129.7, 127.3, 121.3, 116.9, 114.3, 76.3, 66.4, 62.3, 55.7, 45.4, 45.2, 44.7, 30.3, 22.5, 18.6, 13.7.

HRMS (ESI) m/z: $[M + Na]^+$ Calcd for $C_{33}H_{37}NNaO_7^+$ 582.2462; Found 582.2468.

ethyl 3-(4-((2-acetoxybenzoyl)oxy)phenyl)-4-(4-methoxyphenyl)-2-oxomorpholine-3-carboxylate (3h)

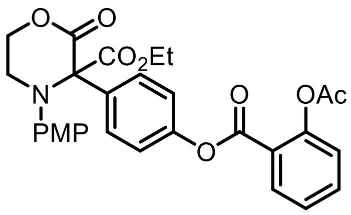

100 °C, 16 h. 24.3 mg, 46% yield, yellow oil.

^{1}H NMR (400 MHz, Chloroform-*d*) δ 8.23 (dd, *J* = 7.9, 1.7 Hz, 1H), 7.92 (d, *J* = 8.8 Hz, 2H), 7.66 (td, *J* = 7.7, 1.7 Hz, 1H), 7.40 (td, *J* = 7.6, 1.2 Hz, 1H), 7.28 – 7.26 (m, 1H), 7.25 – 7.23 (m, 1H), 7.19 (dd, *J* = 8.1, 1.2 Hz, 1H), 6.74 (d, *J* = 9.2 Hz, 2H), 6.64 (d, *J* = 9.2 Hz, 2H), 4.60 (td, *J* = 11.4, 2.2 Hz, 1H), 4.53 (dt, *J* = 11.3, 2.7 Hz, 1H), 4.01 – 3.93 (m, 2H), 3.89 (dd, *J* = 11.6, 3.2 Hz, 1H), 3.77 – 3.73 (m, 1H), 3.72 (s, 3H), 2.34 (s, 3H), 0.86 (t, *J* = 7.1 Hz, 3H).

^{13}C NMR (101 MHz, Chloroform-*d*) δ 169.8, 167.3, 166.8, 162.9, 154.1, 151.4, 151.2, 140.7, 134.8, 133.1, 132.4, 130.9, 126.4, 124.2, 122.6, 121.6, 117.0, 114.4, 76.4, 66.5, 62.3, 55.7, 44.7, 21.2, 13.7.

HRMS (ESI) m/z: $[M + Na]^+$ Calcd for $C_{29}H_{27}NNaO_9^+$ 556.1578; Found 556.1582.

ethyl 3-(3-methoxyphenyl)-4-(4-methoxyphenyl)-2-oxomorpholine-3-carboxylate (3i)

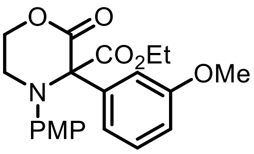

100 °C, 12 h. 19.3 mg, 50% yield, pale yellow foam.

^{1}H NMR (400 MHz, Chloroform-*d*) δ 7.46 – 7.41 (m, 2H), 7.36 – 7.30 (m, 1H), 6.96 – 6.92 (m, 1H), 6.72 (d, *J* = 9.2 Hz, 2H), 6.64 (d, *J* = 9.3 Hz, 2H), 4.59 (td, *J* = 11.4, 2.3 Hz, 1H), 4.51 – 4.46 (m, 1H), 4.01 – 3.86 (m, 3H), 3.82 (s, 3H), 3.75 – 3.72 (m, 1H), 3.71 (s, 3H), 0.85 (t, *J* = 7.1 Hz, 3H).

^{13}C NMR (101 MHz, Chloroform-*d*) δ 167.3, 166.9, 159.7, 154.0, 140.9, 137.1, 129.3, 121.7 117.0, 115.3, 114.7, 114.3, 76.7, 66.4, 62.2, 55.7, 55.5, 44.8, 13.7.

HRMS (ESI) m/z: $[M + Na]^+$ Calcd for $C_{21}H_{23}NNaO_6^+$ 408.1418; Found 408.1428.

ethyl 3-(3-fluorophenyl)-4-(4-methoxyphenyl)-2-oxomorpholine-3-carboxylate (3j)

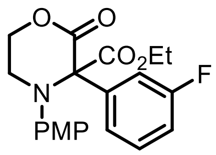

100 °C, 12 h. 18.9 mg, 51% yield, yellow solid, m.p = 103 – 105 °C.

^{1}H NMR (400 MHz, Chloroform-*d*) δ 7.68 – 7.64 (m, 1H), 7.63 – 7.58 (m, 1H), 7.43 – 7.36 (m, 1H), 7.14 – 7.07 (m, 1H), 6.73 (d, *J* = 9.2 Hz, 2H), 6.62 (d, *J* = 9.2 Hz, 2H), 4.62 – 4.49 (m, 2H), 4.00 – 3.92 (m, 2H), 3.91 – 3.85 (m, 1H), 3.77 – 3.73 (m, 1H), 3.71 (s, 3H), 0.86 (t, *J* = 7.1 Hz, 3H).

^{13}C NMR (101 MHz, Chloroform-*d*) δ 167.0, 166.6, 162.9 (d, *J* = 245.7 Hz), 154.2, 140.5, 138.3 (d, *J* = 6.9 Hz), 129.9 (d, *J* = 8.0 Hz), 125.2 (d, *J* = 3.0 Hz), 117.1, 116.8 (d, *J* = 24.3 Hz), 116.1 (d, *J* = 21.2 Hz), 114.4, 76.4, 66.6, 62.4, 55.7, 44.7, 13.7.

^{19}F NMR (376 MHz, Chloroform-*d*) δ -112.4.

HRMS (ESI) m/z: [M + Na]$^+$ Calcd for $C_{20}H_{20}FNNaO_5^+$ 396.1218; Found 396.1223.

ethyl 3-(3,4-dichlorophenyl)-4-(4-methoxyphenyl)-2-oxomorpholine-3-carboxylate (3k)

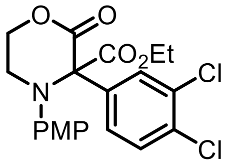

100 °C, 16 h. 19.8 mg, 47% yield, pale yellow foam.

^{1}H NMR (400 MHz, Chloroform-*d*) δ 7.98 (d, *J* = 2.3 Hz, 1H), 7.72 (dd, *J* = 8.5, 2.3 Hz, 1H), 7.51 (d, *J* = 8.6 Hz, 1H), 6.73 (d, *J* = 9.1 Hz, 2H), 6.60 (d, *J* = 9.2 Hz, 2H), 4.63 – 4.52 (m, 2H), 3.98 – 3.91 (m, 2H), 3.91 – 3.84 (m, 1H), 3.77 – 3.73 (m, 1H), 3.72 (s, 3H), 0.86 (t, *J* = 7.1 Hz, 3H).

^{13}C NMR (101 MHz, Chloroform-*d*) δ 166.8, 166.3, 154.4, 140.3, 136.1, 133.6, 132.8, 131.4, 130.4, 129.0, 117.2, 114.5, 76.1, 66.7, 62.6, 55.7, 44.7, 13.7.

HRMS (ESI) m/z: [M + Na]$^+$ Calcd for $C_{20}H_{19}Cl_2NNaO_5^+$ 446.0532; Found 446.0529.

ethyl 4-(4-methoxyphenyl)-3-(naphthalen-2-yl)-2-oxomorpholine-3-carboxylate (3l)

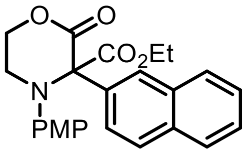

100 °C, 48 h. 20.6 mg, 51% yield, yellow oil.

^{1}H NMR (400 MHz, Chloroform-*d*) δ 8.38 – 8.31 (m, 1H), 7.99 – 7.84 (m, 4H), 7.56 – 7.47 (m, 2H), 6.75 – 6.67 (m, 4H), 4.64 (td, *J* = 11.5, 2.3 Hz, 1H), 4.52 (dt, *J* = 11.2, 2.6 Hz, 1H), 4.04 – 3.93 (m, 3H), 3.81 (dt, *J* = 12.1, 2.2 Hz, 1H), 3.70 (s, 3H), 0.88 (t, *J* = 7.1 Hz, 3H).

^{13}C NMR (101 MHz, Chloroform-*d*) δ 167.5, 166.9, 154.0, 140.9, 133.6, 133.1, 133.0, 129.1, 128.8, 128.1, 127.7, 127.0, 126.8, 126.4, 117.1, 114.3, 77.0, 66.5, 62.3, 55.7, 44.9, 13.7.

HRMS (ESI) m/z: $[M + Na]^+$ Calcd for $C_{24}H_{23}NNaO_5^+$ 428.1468; Found 428.1481.

ethyl 3-(benzo[d][1,3]dioxol-5-yl)-4-(4-methoxyphenyl)-2-oxomorpholine-3-carboxylate (3m)

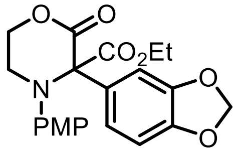

100 °C, 12 h. 21.5 mg, 54% yield, yellow oil.

^{1}H NMR (400 MHz, Chloroform-*d*) δ 7.37 – 7.33 (m, 2H), 6.85 – 6.81 (m, 1H), 6.74 – 6.69 (m, 2H), 6.67 – 6.63 (m, 2H), 6.00 (s, 2H), 4.59 (td, *J* = 11.4, 2.3 Hz, 1H), 4.49 (dt, *J* = 11.2, 2.7 Hz, 1H), 3.99 – 3.89 (m, 2H), 3.86 (dd, *J* = 11.8, 3.1 Hz, 1H), 3.71 (s, 3H), 3.71 – 3.67 (m, 1H), 0.85 (t, *J* = 7.1 Hz, 3H).

^{13}C NMR (101 MHz, Chloroform-*d*) δ 167.5, 167.0, 154.0, 148.4, 148.1, 140.8, 129.1, 123.2, 117.0, 114.3, 110.2, 107.9, 101.6, 76.4, 66.4, 62.2, 55.7, 44.8, 13.7.

HRMS (ESI) m/z: $[M + Na]^+$ Calcd for $C_{21}H_{21}NNaO_7^+$ 422.1210; Found 422.1217.

methyl 4-(4-methoxyphenyl)-2-oxo-3-(thiophen-3-yl)morpholine-3-carboxylate (3n)

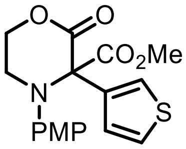

100 °C, 12 h. 19.3 mg, 56% yield, yellow solid, m.p = 154 – 156 °C.

^{1}H NMR (400 MHz, Chloroform-*d*) δ 7.67 – 7.64 (m, 1H), 7.41 – 7.38 (m, 1H), 7.37 – 7.34 (m, 1H), 6.77 – 6.65 (m, 4H), 4.65 (td, *J* = 11.2, 2.3 Hz, 1H), 4.55 (dt, *J* = 11.2, 2.8 Hz, 1H), 3.89 – 3.80 (m, 1H), 3.72 (s, 3H), 3.66 (dt, *J* = 12.3, 2.4 Hz, 1H), 3.49 (s, 3H).

^{13}C NMR (101 MHz, Chloroform-*d*) δ 168.0, 166.4, 154.3, 140.7, 137.8, 128.6, 126.6, 126.0, 117.6, 114.4, 74.9, 67.1, 55.6, 53.0, 44.6.

HRMS (ESI) m/z: $[M + Na]^+$ Calcd for $C_{17}H_{17}NNaO_5S^+$ 370.0720; Found 370.0724.

ethyl 4-(4-methoxyphenyl)-2-oxo-3-(thiophen-2-yl)morpholine-3-carboxylate (3o)

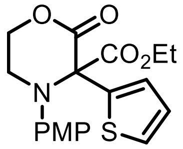

100 °C, 16 h. 17.2 mg, 50% yield, yellow solid, m.p = 142 – 144°C.

^{1}H NMR (400 MHz, Chloroform-*d*) δ 7.55 – 7.51 (m, 1H), 7.40 – 7.36 (m, 1H), 7.12 – 7.09 (m, 1H), 6.79 – 6.70 (m, 4H), 4.84 (td, *J* = 11.4, 2.3 Hz, 1H), 4.59 (dt, *J* = 11.2, 2.6 Hz, 1H), 4.04 – 3.92 (m, 2H), 3.83 (td, *J* = 11.9, 2.8 Hz, 1H), 3.71 (s, 3H), 3.69 – 3.61 (m, 1H), 0.89 (t, *J* = 7.1 Hz, 3H).

^{13}C NMR (101 MHz, Chloroform-*d*) δ 166.9, 166.3, 154.6, 141.6, 140.6, 128.9, 128.2, 127.4, 117.7, 114.3, 74.5, 67.2, 62.5, 55.6, 44.6, 13.7.

HRMS (ESI) m/z: $[M + Na]^+$ Calcd for $C_{18}H_{19}NNaO_5S^+$ 384.0876; Found 384.0879.

ethyl 4-(4-methoxyphenyl)-3-methyl-2-oxomorpholine-3-carboxylate (3p)

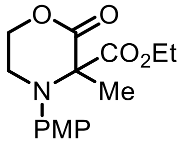

100 °C, 12 h. 19.3 mg, 66% yield, yellow oil.

^{1}H NMR (400 MHz, Chloroform-*d*) δ 6.99 (d, *J* = 9.0 Hz, 2H), 6.81 (d, *J* = 8.9 Hz, 2H), 4.62 – 4.47 (m, 2H), 4.17 (q, *J* = 7.1 Hz, 2H), 3.92 – 3.81 (m, 1H), 3.77 (s, 3H), 3.23 (dt, *J* = 12.9, 4.1 Hz, 1H), 1.64 (s, 3H), 1.23 (t, *J* = 7.1 Hz, 3H).

^{13}C NMR (101 MHz, Chloroform-*d*) δ 169.4, 168.9, 157.2, 139.7, 126.0, 114.3, 70.6, 68.8, 62.0, 55.6, 46.0, 22.2, 14.1.

HRMS (ESI) m/z: [M + Na]$^+$ Calcd for $C_{15}H_{19}NNaO_5^+$ 316.1155; Found 316.1149.

ethyl 3-ethyl-4-(4-methoxyphenyl)-2-oxomorpholine-3-carboxylate (3q)

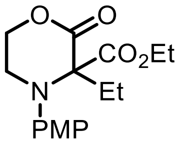

100 ℃, 12 h. 16.3 mg, 53% yield, yellow oil.

^{1}H NMR (400 MHz, Chloroform-*d*) δ 7.00 (d, *J* = 9.0 Hz, 2H), 6.81 (d, *J* = 9.0 Hz, 2H), 4.59 (td, *J* = 10.6, 2.9 Hz, 1H), 4.48 (dt, *J* = 10.6, 2.8 Hz, 1H), 4.17 – 4.07 (m, 3H), 3.77 (s, 3H), 3.14 (dt, *J* = 12.8, 2.8 Hz, 1H), 2.26 –2.08 (m, 2H), 1.21 (t, *J* = 7.1 Hz, 3H), 1.10 (t, *J* = 7.4 Hz, 3H).

^{13}C NMR (101 MHz, Chloroform-*d*) δ 169.2, 168.6, 157.2, 139.7, 125.6, 114.3, 75.2, 68.9, 61.7, 55.6, 46.4, 28.2, 14.2, 8.2.

HRMS (ESI) m/z: [M + Na]$^+$ Calcd for $C_{16}H_{21}NNaO_5^+$ 330.1312; Found 330.1314.

ethyl 3-butyl-4-(4-methoxyphenyl)-2-oxomorpholine-3-carboxylate (3r)

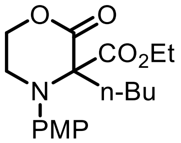

100 ℃, 12 h. 17.8 mg, 53% yield, yellow oil.

^{1}H NMR (400 MHz, Chloroform-*d*) δ 6.98 (d, *J* = 9.0 Hz, 2H), 6.81 (d, *J* = 9.0 Hz, 2H), 4.59 (td, *J* = 10.6, 2.9 Hz, 1H), 4.48 (dt, *J* = 10.6, 2.7 Hz, 1H), 4.15 – 4.06 (m, 3H), 3.77 (s, 3H), 3.12 (dt, *J* = 12.8, 2.8 Hz, 1H), 2.24 – 2.04 (m, 2H), 1.87 – 1.73 (m, 1H), 1.42 – 1.34 (m, 2H), 1.33 – 1.25 (m, 1H), 1.22 (t, *J* = 7.1 Hz, 3H), 0.93 (t, *J* = 7.3 Hz, 3H).

^{13}C NMR (101 MHz, Chloroform-*d*) δ 169.2, 168.7, 157.2, 139.7, 125.6, 114.3, 74.6, 68.9, 61.7, 55.6, 46.4, 35.0, 25.9, 22.7, 14.18, 14.15.

HRMS (ESI) m/z: [M + Na]$^+$ Calcd for $C_{18}H_{25}NNaO_5^+$ 358.1625; Found 358.1624.

ethyl 3-(chloromethyl)-4-(4-methoxyphenyl)-2-oxomorpholine-3-carboxylate (3s)

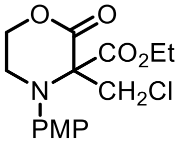

100 °C, 12 h. 17.7 mg, 54% yield, yellow oil.

^{1}H NMR (400 MHz, Chloroform-*d*) δ 7.10 (d, *J* = 8.9 Hz, 2H), 6.84 (d, *J* = 9.0 Hz, 2H), 4.72 (td, *J* = 10.4, 2.8 Hz, 1H), 4.52 (dt, *J* = 10.6, 2.9 Hz, 1H), 4.25 – 4.13 (m, 3H), 4.12 – 4.06 (m, 1H), 3.87 (d, *J* = 11.8 Hz, 1H), 3.79 (s, 3H), 3.25 (dt, *J* = 12.7, 3.0 Hz, 1H), 1.24 (t, *J* = 7.1 Hz, 3H).

^{13}C NMR (101 MHz, Chloroform-*d*) δ 167.6, 166.1, 157.9, 138.5, 126.4, 114.6, 74.9, 69.1, 62.4, 55.6, 47.0, 46.1, 14.1.

HRMS (ESI) m/z: $[M + Na]^+$ Calcd for $C_{15}H_{18}ClNNaO_5^+$ 350.0766; Found 350.0760.

4-(4-methoxyphenyl)-3-phenyl-3-(trifluoromethyl)morpholin-2-one (3t)

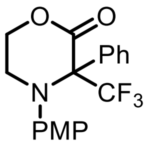

100 °C, 36 h. 7.4 mg, 21% yield, yellow oil.

^{1}H NMR (400 MHz, Chloroform-*d*) δ 7.44 – 7.33 (m, 5H), 6.63 (d, *J* = 8.7 Hz, 2H), 6.65 (d, *J* = 9.0 Hz, 2H), 4.68 – 4.61 (m, 1H), 4.54 – 4.46 (m, 1H), 3.73 (s, 3H), 3.58 – 3.48 (m, 2H).

^{13}C NMR (101 MHz, Chloroform-*d*) δ 165.2, 157.6, 140.0, 135.0, 129.4, 128.9, 128.8, 128.4 (q, *J* = 1.8 Hz), 124.54 (q, *J* = 291.3 Hz), 113.7, 75.3 (q, *J* = 24.3 Hz), 68.4, 55.4, 47.9.

^{19}F NMR (376 MHz, Chloroform-*d*) δ -64.1.

HRMS (ESI) m/z: $[M + Na]^+$ Calcd for $C_{18}H_{16}F_3NNaO_3^+$ 374.0974; Found 374.0972.

methyl 4-(4-methoxyphenyl)-2-oxo-3-phenylmorpholine-3-carboxylate (3u)

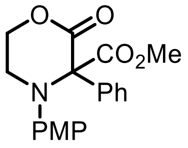

100 °C, 12 h. 18.5 mg, 54% yield, yellow solid, m.p = 195 – 197 °C.

^{1}H NMR (400 MHz, Chloroform-*d*) δ 7.86 – 7.81 (m, 2H), 7.47 – 7.37 (m, 3H), 6.72 (d, *J* = 9.2 Hz, 2H), 6.63 (d, *J* = 9.2 Hz, 2H), 4.58 (td, *J* = 11.4, 2.3 Hz, 1H), 4.53 – 4.47 (m, 1H), 3.92 (td, *J* = 11.8, 3.1 Hz, 1H), 3.77 – 3.73 (m, 1H), 3.71 (s, 3H), 3.47 (s, 3H).

^{13}C NMR (101 MHz, Chloroform-*d*) δ 168.1, 166.9, 153.9, 140.7, 135.3, 129.4, 129.1, 128.5, 117.0, 114.3, 76.8, 66.5, 55.6, 53.0, 44.8.

HRMS (ESI) m/z: $[M + Na]^+$ Calcd for $C_{19}H_{19}NNaO_5^+$ 364.1155; Found 364.1160.

isopropyl 4-(4-methoxyphenyl)-2-oxo-3-phenylmorpholine-3-carboxylate (3v)

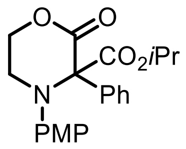

100 °C, 12 h. 19.2 mg, 52% yield, yellow oil.

^{1}H NMR (400 MHz, Chloroform-*d*) δ 7.88 – 7.83 (m, 2H), 7.47 – 7.37 (m, 3H), 6.72 (d, *J* = 9.2 Hz, 2H), 6.62 (d, *J* = 9.2 Hz, 2H), 4.79 (sept, *J* = 6.3 Hz, 1H), 4.57 (td, *J* = 11.3, 2.3 Hz, 1H), 4.52 – 4.46 (m, 1H), 3.88 (td, *J* = 11.8, 3.1 Hz, 1H), 3.74 (dt, *J* = 12.1, 2.2 Hz, 1H), 3.70 (s, 3H), 1.15 (d, *J* = 6.3 Hz, 3H), 0.63 (d, *J* = 6.2 Hz, 3H).

^{13}C NMR (101 MHz, Chloroform-*d*) δ 167.0, 166.8, 153.9, 140.9, 135.3, 129.5, 129.0, 128.4, 116.8, 114.3, 76.6, 70.1, 66.3, 55.7, 44.8, 21.4, 21.1.

HRMS (ESI) m/z: $[M + Na]^+$ Calcd for $C_{21}H_{23}NNaO_5^+$ 392.1468; Found 392.1473.

benzyl 4-(4-methoxyphenyl)-3-methyl-2-oxomorpholine-3-carboxylate (3w)

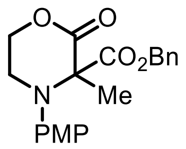

100 °C, 12 h. 17.9 mg, 50% yield, yellow oil.

^{1}H NMR (400 MHz, Chloroform-*d*) δ 7.38 – 7.33 (m, 3H), 7.30 – 7.27 (m, 2H), 6.85 (d, *J* = 9.0 Hz, 2H), 6.71 (d, *J* = 9.0 Hz, 2H), 5.18 (d, *J* = 12.2 Hz, 1H), 5.12 (d, *J* = 12.2 Hz, 1H), 4.59 – 4.46 (m, 2H), 3.88 – 3.79 (m, 1H), 3.76 (s, 3H), 3.20 – 3.13 (m, 1H), 1.67 (s, 3H).

^{13}C NMR (101 MHz, Chloroform-*d*) δ 169.2, 168.8, 157.2, 139.5, 135.2, 128.72, 128.66, 128.6, 126.2, 114.2, 70.7, 68.8, 67.6, 55.5, 46.0, 22.4.

HRMS (ESI) m/z: $[M + Na]^+$ Calcd for $C_{20}H_{21}NNaO_5^+$ 378.1312; Found 378.1313.

4-(4-methoxyphenyl)-*N*, *N*,3-trimethyl-2-oxomorpholine-3-carboxamide (3x)

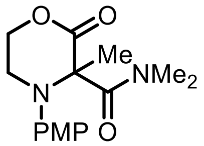

100 °C, 12 h. 23.8 mg, 82% yield, white oil.

^{1}H NMR (400 MHz, Chloroform-*d*) δ 6.85 (d, *J* = 9.1 Hz, 2H), 6.79 (d, *J* = 9.1 Hz, 2H), 4.69 – 4.61 (m, 1H), 4.57 – 4.50 (m, 1H), 3.75 (s, 3H), 3.72 – 3.66 (m, 1H), 3.27 (s, 3H), 3.25 – 3.21 (m, 1H), 3.00 (s, 3H), 1.58 (s, 3H).

^{13}C NMR (101 MHz, Chloroform-*d*) δ 169.4, 168.2, 156.6, 139.5, 124.2, 114.4, 70.2, 67.6, 55.5, 44.7, 37.6, 37.2, 18.3.

HRMS (ESI) m/z: $[M + Na]^+$ Calcd for $C_{15}H_{20}N_2NaO_4^+$ 315.1315; Found 315.1311.

***N*-methoxy-4-(4-methoxyphenyl)-*N*,3-dimethyl-2-oxomorpholine-3-carboxamide (3y)**

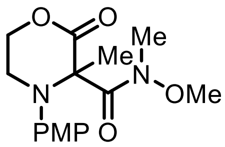

100 °C, 48 h. 24.5 mg, 80% yield, white solid, m.p = 108 – 110 °C.

^{1}H NMR (400 MHz, Chloroform-*d*) δ 6.93 (d, *J* = 9.1 Hz, 2H), 6.80 (d, *J* = 9.1 Hz, 2H), 4.59 (td, *J* = 10.5, 3.0 Hz 1H), 4.47 (dt, *J* = 10.8, 3.2 Hz, 1H), 3.82 (s, 3H), 3.76 (s, 3H), 3.67 –3.58 (m, 1H), 3.28 – 3.20 (m, 4H), 1.47 (s, 3H).

^{13}C NMR (101 MHz, Chloroform-*d*) δ 170.9, 169.0, 156.5, 139.9, 124.2, 114.3, 69.1, 67.7, 60.8, 55.5, 44.6, 33.7, 18.3.

HRMS (ESI) m/z: $[M + Na]^+$ Calcd for $C_{15}H_{20}N_2NaO_5^+$ 331.1264; Found 331.1259.

(1*R*,2*S*,5*R*)-2-isopropyl-5-methylcyclohexyl 4-(4-methoxyphenyl)-3-methyl-2-oxomorpholine-3-carboxylate (3z)

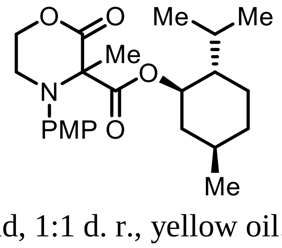

100 °C, 16 h. 30.2 mg, 75% yield, 1:1 d. r., yellow oil.

^{1}H NMR (400 MHz, Chloroform-*d*) δ (mixture of two diastereoisomers) 7.01 (d, *J* = 12.9 Hz, 1H), 6.98 (d, *J* = 12.9 Hz, 1H), 6.80 (d, *J* = 9.0 Hz, 1H), 6.79 (d, *J* = 9.0 Hz, 1H), 4.73 – 4.61 (m, 1H), 4.59 – 4.48 (m, 2H), 3.91 – 3.82 (m, 1H), 3.76 (s, 3H), 3.37 –3.22 (m, 1H), 1.96 – 1.87 (m, 1H), 1.82 – 1.74 (m, 1H), 1.66 (s, 3H), 1.62 (s, 3H),

1.52 – 1.31 (m, 3H), 1.07 – 0.91 (m, 2H), 0.88 (d, *J* = 6.4 Hz, 3H), 0.86 – 0.80 (m, 5H), 0.68 (dd, *J* = 17.3, 6.9 Hz, 3H).

^{13}C NMR (101 MHz, Chloroform-*d*) δ (mixture of two diastereoisomers) 169.7, 169.2, 169.0, 168.9, 156.8, 156.5, 139.8, 139.8, 125.4, 124.8, 114.2, 114.1, 76.7, 76.6, 70.50, 70.49, 68.5, 68.4, 55.53, 55.51, 47.1, 46.8, 46.1, 45.9, 40.6, 39.9, 34.2, 34.1, 31.5, 31.4, 25.9, 25.7, 23.1, 23.0, 22.8, 22.1, 22.0, 21.9, 21.00, 20.96, 15.90, 15.88.

HRMS (ESI) m/z: $[M + Na]^+$ Calcd for $C_{23}H_{33}NNaO_5^+$ 426.2251; Found 426.2262.

ethyl 2-oxo-3,4-diphenylmorpholine-3-carboxylate (3aa)

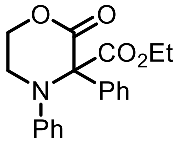

100 °C, 12 h. 16.6 mg, 51% yield, pale yellow foam.

^{1}H NMR (400 MHz, Chloroform-*d*) δ 7.84 – 7.78 (m, 2H), 7.47 – 7.37 (m, 3H), 7.19 – 7.11 (m, 2H), 6.87 – 6.81 (m, 1H), 6.68 –6.62 (m, 2H), 4.60 – 4.46 (m, 2H), 4.03 – 3.87 (m, 3H), 3.84 (dt, *J* = 12.1, 2.4 Hz, 1H), 0.79 (t, *J* = 7.1 Hz, 3H).

^{13}C NMR (101 MHz, Chloroform-*d*) δ 167.4, 166.8, 146.9, 134.7, 129.4, 129.2, 129.0, 128.5, 120.2, 115.2, 76.3, 66.0, 62.4, 44.4, 13.5.

HRMS (ESI) m/z: $[M + Na]^+$ Calcd for $C_{19}H_{19}NNaO_4^+$ 348.1206; Found 348.1205.

ethyl 2-oxo-3-phenyl-4-(o-tolyl)morpholine-3-carboxylate (3ab)

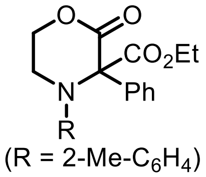

100 °C, 12 h. 15.6 mg, 46% yield, pale yellow foam.

^{1}H NMR (400 MHz, Chloroform-*d*) δ 7.59 – 7.51 (m, 2H), 7.31 – 7.26 (m, 3H), 7.20 – 7.09 (m, 2H), 7.02 – 6.95 (m, 2H), 4.53 – 4.46 (m, 2H), 4.18 – 4.06 (m, 2H), 3.74 – 3.64 (m, 1H), 3.56 – 3.42 (m, 1H), 2.28 (s, 3H), 1.07 (t, *J* = 7.1 Hz, 3H).

^{13}C NMR (101 MHz, Chloroform-*d*) δ 168.1, 167.1, 145.4, 135.6, 135.5, 131.4, 128.8, 128.6, 128.2, 126.4, 126.0, 125.6, 76.1, 66.8, 62.4, 46.0, 19.0, 13.7.

HRMS (ESI) m/z: $[M + Na]^+$ Calcd for $C_{20}H_{21}NNaO_4^+$ 362.1363; Found 362.1364.

ethyl 4-methyl-2-oxo-3-phenylmorpholine-3-carboxylate (3ac)

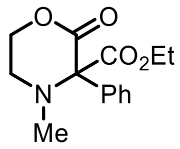

100 °C, 16 h. 13.2 mg, 50% yield, yellow oil.

^{1}H NMR (400 MHz, Chloroform-*d*) δ 7.75 – 7.70 (m, 2H), 7.40 – 7.30 (m, 3H), 4.64 (td, *J* = 11.2, 3.5 Hz, 1H), 4.44 – 4.29 (m, 3H), 3.50 – 3.40 (m, 1H), 2.95 – 2.89 (m, 1H), 2.32 (s, 3H), 1.41 – 1.33 (m, 3H).

^{13}C NMR (101 MHz, Chloroform-*d*) δ 167.1, 166.8, 136.0, 129.1, 128.7, 128.0, 76.7, 68.7, 62.0, 46.6, 39.6, 14.7.

HRMS (ESI) m/z: $[M + Na]^+$ Calcd for $C_{14}H_{17}NNaO_4^+$ 286.1050; Found 286.1058.

4-benzyl-*N, N*,3-trimethyl-2-oxomorpholine-3-carboxamide (3ad)

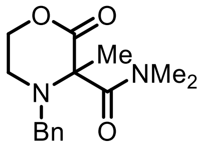

100 °C, 48 h. 14.4 mg, 52% yield, yellow oil.

^{1}H NMR (400 MHz, Chloroform-*d*) δ 7.36 – 7.27 (m, 3H), 7.22 – 7.17 (m, 2H), 4.44 (td, *J* = 11.6, 3.9 Hz, 1H), 4.36 (dd, *J* = 11.3, 4.0 Hz, 1H), 3.72 (d, *J* = 13.6 Hz, 1H), 3.33 – 3.26 (m, 4H), 3.05 (s, 3H), 2.90 (td, *J* = 12.5, 4.1 Hz, 1H), 2.73 (dd, *J* = 13.1, 3.9 Hz, 1H), 1.68 (s, 3H).

^{13}C NMR (101 MHz, Chloroform-*d*) δ 169.2, 168.4, 137.9, 128.8, 128.74, 127.69, 69.7, 68.7, 55.0, 41.3, 37.7, 37.6, 15.1.

HRMS (ESI) m/z: $[M + Na]^+$ Calcd for $C_{15}H_{20}N_2NaO_3^+$ 299.1366; Found 299.1372.

ethyl 5,5-dimethyl-2-oxo-3-phenylmorpholine-3-carboxylate (3ae)

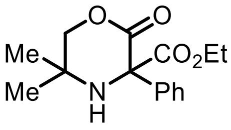

100 °C, 12 h. 14.4 mg, 63% yield, colorless oil.

^{1}H NMR (400 MHz, Chloroform-d) δ 7.63 – 7.57 (m, 2H), 7.41 – 7.30 (m, 3H), 4.32 – 4.12 (m, 2H), 3.88 – 3.79 (m, 2H), 2.80 (brs, 1H), 1.34 (s, 3H), 1.21 (t, *J* = 7.1 Hz, 3H), 1.14 (s, 3H).

^{13}C NMR (101 MHz, Chloroform-d) δ 169.8, 168.4, 137.7, 128.6, 128.6, 127.2, 74.6, 69.5, 63.24, 5.15, 27.55, 27.48, 13.9.

HRMS (ESI) m/z: $[M + Na]^+$ Calcd for $C_{15}H_{19}NNaO_4^+$ 300.1206; Found 300.1208.

ethyl 6,6-dimethyl-2-oxo-3-phenylmorpholine-3-carboxylate (3af)

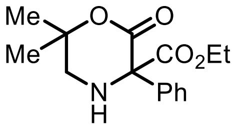

100 °C, 12 h. 9.8 mg, 43% yield, colorless oil.

^{1}H NMR (400 MHz, Chloroform-*d*) δ 7.61 – 7.55 (m, 2H), 7.41 – 7.31 (m, 3H), 4.38 – 4.26 (m, 2H), 2.97 (brs, 1H), 2.92 (s, 2H), 1.50 (s, 3H), 1.47 (s, 3H), 1.31 (t, *J* = 7.1 Hz, 3H).

^{13}C NMR (101 MHz, Chloroform-*d*) δ 170.5, 166.1, 137.3, 128.7, 128.5, 127.5, 83.8, 71.3, 63.0, 49.7, 26.9, 26.7, 14.2.

HRMS (ESI) m/z: $[M + Na]^+$ Calcd for $C_{15}H_{19}NNaO_4^+$ 300.1206; Found 300.1205.

ethyl (3*S*,5*S*)-4-(4-methoxyphenyl)-5-methyl-2-oxo-3-phenylmorpholine-3-carboxylate (3ag)

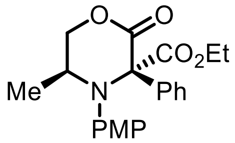

100 °C, 4 days. 18.8 mg, 51% yield, 10:1 d. r., >99% *ee*, yellow solid, m.p =174 – 176 °C.

^{1}H NMR (400 MHz, Chloroform-*d*) δ 7.86 (d, *J* = 6.7 Hz, 2H), 7.49 – 7.35 (m, 3H), 6.86 (d, *J* = 8.9 Hz, 2H), 6.70 (d, *J* = 8.9 Hz, 2H), 4.36 – 4.24 (m, 2H), 4.20 – 4.11 (m, 1H), 3.98 – 3.87 (m, 1H), 3.80 – 3.74 (m, 1H), 3.72 (s, 3H), 1.26 (d, *J* = 5.7 Hz, 3H), 0.96 (t, *J* = 7.2 Hz, 3H).

^{13}C NMR (101 MHz, Chloroform-*d*) δ 167.6, 167.4, 155.6, 138.7, 137.3, 129.4, 128.9, 128.3, 123.9, 113.8, 77.8, 70.8, 62.1, 55.5, 48.2, 16.2, 13.7.

HRMS (ESI) m/z: $[M + Na]^+$ Calcd for $C_{21}H_{23}NNaO_5^+$ 392.1468; Found 392.1475.

$[\alpha]^{24}_D$ -279.7 (*c* 1.0, $CHCl_3$).

Chiral HPLC: Chiralpak AS, hexane:*i*PrOH = 90:10, 1.0 mL/min, 254 nm; tR = 9.2 min (major), 10.0 min (minor).

ethyl (3*R*,5*R*)-5-benzyl-4-(4-methoxyphenyl)-2-oxo-3-phenylmorpholine-3-carboxylate (3ah)

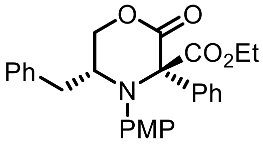

100 °C, 48 h. 22.3 mg, 50% yield, >20:1 d. r., colorless oil.

^{1}H NMR (400 MHz, Chloroform-*d*) δ 7.77 – 7.69 (m, 2H), 7.43 – 7.37 (m, 3H), 7.36 – 7.27 (m, 3H), 7.11 (d, *J* = 7.3 Hz, 2H), 7.01 (d, *J* = 9.0 Hz, 2H), 6.78 (d, *J* = 9.0 Hz, 2H), 4.38 (tt, *J* = 9.7, 3.2 Hz, 1H), 4.26 (dd, *J* = 11.5, 3.3 Hz, 1H), 4.19 – 4.11 (m, 1H), 3.98 – 3.88 (m, 1H), 3.75 (s, 3H), 3.75 – 3.69 (m, 1H), 3.33 (dd, *J* = 14.1, 2.9 Hz, 1H), 2.65 (dd, *J* = 14.1, 9.4 Hz, 1H), 0.96 (t, *J* = 7.1 Hz, 3H).

^{13}C NMR (101 MHz, Chloroform-*d*) δ 167.7, 167.5, 155.9, 138.6, 137.3, 136.7, 129.4, 129.3, 128.91, 128.86, 128.3, 127.2, 124.5, 114.0, 77.8, 68.7, 62.2, 55.6, 54.7, 36.8, 13.7.

HRMS (ESI) m/z: $[M + Na]^+$ Calcd for $C_{27}H_{27}NNaO_5^+$ 468.1781; Found 468.1799.

$[\alpha]^{24}_D$ +173.2 (*c* 1.0, $CHCl_3$).

ethyl (3*R*,5*R*)-5-isopropyl-4-(4-methoxyphenyl)-2-oxo-3-phenylmorpholine-3-carboxylate (3ai)

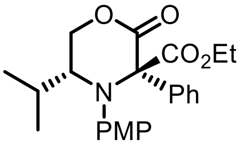

100 °C, 12 h. 16.5 mg, 42% yield, 10:1 d. r., yellow oil.

^{1}H NMR (400 MHz, Chloroform-*d*) δ 7.87 – 7.83 (m, 2H), 7.42 – 7.32 (m, 3H), 7.00 (d, *J* = 9.0 Hz, 2H), 6.67 (d, *J* = 9.1 Hz, 2H), 4.39 – 4.33 (m, 2H), 4.06 – 3.92 (m, 2H), 3.84 – 3.76 (m, 1H), 3.71 (s, 3H), 2.33 – 2.19 (m, 1H), 1.10 (t, *J* = 7.1 Hz, 3H), 1.01 (d, *J* = 7.1 Hz, 3H), 0.97 (d, *J* = 7.0 Hz, 3H).

^{13}C NMR (101 MHz, Chloroform-*d*) δ 168.0, 167.9, 156.5, 137.9, 137.6, 129.7, 128.7, 128.1, 127.2, 113.5, 78.0, 66.3, 62.1, 58.6, 55.4, 27.4, 19.6, 15.9, 13.9.

HRMS (ESI) m/z: $[M + Na]^+$ Calcd for $C_{23}H_{27}NNaO_5^+$ 420.1781; Found 420.1784.

$[\alpha]^{24}_D$ +87.5 (*c* 1.0, $CHCl_3$).

(3*R*,5*R*)-5-benzyl-4-(4-methoxyphenyl)-*N*,*N*,3-trimethyl-2-oxomorpholine-3-carboxamide (3aj)

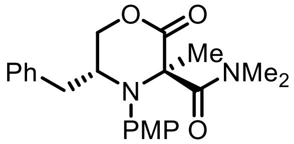

100 °C, 12 h. 28.5 mg, 75% yield, >20:1 d. r., yellow oil.

^{1}H NMR (400 MHz, Chloroform-*d*) δ 7.32 – 7.28 (m, 2H), 7.28 – 7.25 (m, 2H), 7.23 – 7.17 (m, 1H), 7.05 (d, *J* = 6.8 Hz, 2H), 6.88 (d, *J* = 8.9 Hz, 2H), 4.28 (d, *J* = 6.0 Hz, 2H), 4.08 (dt, *J* = 8.4, 6.0 Hz, 1H), 3.82 (s, 3H), 2.96 (d, *J* = 21.3 Hz, 6H), 2.63 (dd, *J* = 14.1, 5.7 Hz, 1H), 2.44 (dd, *J* = 14.0, 8.2 Hz, 1H), 1.39 (s, 3H).

^{13}C NMR (101 MHz, Chloroform-*d*) δ 170.4, 170.2, 158.1, 137.7, 134.4, 131.4, 129.3, 128.6, 126.8, 114.1, 69.8, 68.3, 56.7, 55.5, 37.5, 36.9, 21.2.

HRMS (ESI) m/z: $[M + Na]^+$ Calcd for $C_{22}H_{26}N_2NaO_4^+$ 405.1785; Found 405.1791.
$[\alpha]^{24}_D$ +81.1 (*c* 1.0, $CHCl_3$).

(3*R*,5*R*)-5-benzyl-N-methoxy-4-(4-methoxyphenyl)-*N*,3-dimethyl-2-oxomorpholine-3-carboxamide (3ak)

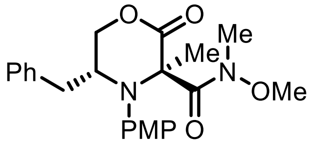

100 °C, 12 h. 23.9 mg, 60% yield, >20:1 d. r., white solid, m.p= 115 – 117 °C.

^{1}H NMR (400 MHz, Chloroform-*d*) δ 7.71 (d, *J* = 8.9 Hz, 2H), 7.60 – 7.57 (m, 2H), 7.56 – 7.51 (m, 1H), 7.40 – 7.35 (m, 2H), 7.21 (d, *J* = 9.0 Hz, 2H), 4.52 (dd, *J* = 4.8, 1.0 Hz, 2H), 4.43 –4.35 (m, 1H), 4.14 (s, 3H), 3.94 (s, 3H), 3.33 (s, 3H), 3.12 (dd, *J* = 13.9, 3.4 Hz, 1H), 2.72 (dd, *J* = 13.8, 10.2 Hz, 1H), 1.94 (s, 3H).

^{13}C NMR (101 MHz, Chloroform-*d*) δ 171.3, 170.0, 157.7, 137.7, 136.3, 130.8, 129.4, 128.7, 126.7, 113.9, 68.0, 67.2, 60.2, 56.4, 55.5, 37.8, 32.9, 25.4.

HRMS (ESI) m/z: $[M + Na]^+$ Calcd for $C_{22}H_{26}N_2NaO_5^+$ 421.1734; Found 421.1741.
$[\alpha]^{24}_D$ +93.6 (*c* 1.0, $CHCl_3$).

ethyl (3*R*,5*R*)-4-(4-methoxyphenyl)-2-oxo-3,5-diphenylmorpholine-3-carboxylate (3al)

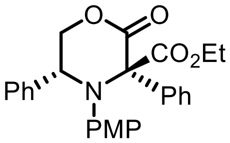

100 °C, 36 h. 22.5 mg, 52% yield, >20:1 d. r., >99% *ee*, yellow solid, m.p = 133 – 135 °C.

^{1}H NMR (400 MHz, Chloroform-*d*) δ 8.19 – 8.12 (m, 2H), 7.56 – 7.43 (m, 5H), 7.37 – 7.30 (m, 2H), 7.26 – 7.23 (m, 1H), 6.73 (d, *J* = 9.1 Hz, 2H), 6.50 (d, *J* = 9.1 Hz, 2H), 5.21 (dd, *J* = 10.2, 3.2 Hz, 1H), 4.46 – 4.31 (m, 2H), 3.99 – 3.90 (m, 1H), 3.81 – 3.70 (m, 1H), 3.61 (s, 3H), 1.00 (t, *J* = 7.1 Hz, 3H).

^{13}C NMR (101 MHz, Chloroform-*d*) δ 167.4, 167.2, 155.3, 139.1, 137.5, 137.1, 129.7, 129.2, 129.1, 128.43, 128.40, 127.9, 123.7, 113.4, 78.1, 71.7, 62.2, 59.4, 55.3, 13.8.

HRMS (ESI) m/z: $[M + Na]^+$ Calcd for $C_{26}H_{25}NNaO_5^+$ 454.1625; Found 454.1638.

$[\alpha]^{24}_D$ +269.3 (*c* 1.0, $CHCl_3$).

Chiral HPLC: Chiralpak AD, hexane:*i*PrOH = 90:10, 1.0 mL/min, 254 nm; tR = 8.3 min (major), 9.0 min (minor).

(3*R*,5*R*)-4-benzyl-*N*-methoxy-*N*,3-dimethyl-2-oxo-5-phenylmorpholine-3-carboxamide (3am)

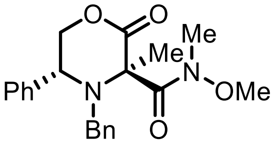

100 °C, 48 h. 14.7 mg, 40% yield, 6:1 d. r., >99% *ee*, white solid, m.p = 132 – 134 °C.

^{1}H NMR (400 MHz, Chloroform-d) δ 7.36 – 7.31 (m, 2H), 7.30 – 7.26 (m, 2H), 7.22 – 7.08 (m, 6H), 4.62 (dd, *J* = 11.7, 4.7 Hz, 1H), 4.45 (dd, *J* = 11.7, 2.4 Hz, 1H), 4.22 – 4.11 (m, 2H), 3.91 (d, *J* = 15.9 Hz, 1H), 3.70 (s, 3H), 3.26 (s, 3H), 1.49 (s, 3H).

^{13}C NMR (101 MHz, Chloroform-d) δ 171.4, 169.6, 140.1, 139.6, 128.7, 128.6, 128.3, 128.2, 127.8, 126.8, 71.1, 67.1, 60.1, 58.1, 52.2, 33.0, 21.9.

HRMS (ESI) m/z: $[M + Na]^+$ Calcd for $C_{21}H_{24}N_2NaO_4^+$ 391.1628; Found 391.1635.

$[\alpha]^{24}_D$ -69.7 (*c* 1.0, $CHCl_3$).

Chiral HPLC: Chiralpak AS, hexane:*i*PrOH = 90:10, 1.0 mL/min, 220 nm; tR = 9.3 min (major), 13.8 min (minor).

ethyl (3*S*,6*S*)-4-(4-methoxyphenyl)-6-methyl-2-oxo-3-phenylmorpholine-3-carboxylate (3an)

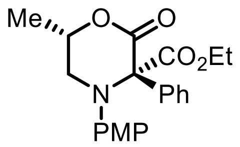

100 °C, 12 h. 18.8 mg, 51% yield, 4:1 d. r., white solid, m.p = 140 – 142 °C.

^{1}H NMR (400 MHz, Chloroform-d) δ (major diastereoisomer) 7.87 – 7.81 (m, 2H), 7.46 –7.37 (m, 3H), 6.71 (d, *J* = 8.5 Hz, 2H), 6.61 (d, *J* = 7.5 Hz, 2H), 4.80 – 4.68 (m, 1H), 4.01 – 3.89 (m, 2H), 3.77 –3.73 (m, 1H), 3.71 (s, 3H), 3.60 – 3.51 (m, 1H), 1.46 (d, *J* = 6.4 Hz, 3H), 0.85 (t, *J* = 7.1 Hz, 3H).

^{13}C NMR (101 MHz, Chloroform-d) δ (major diastereoisomer) 167.6, 167.3, 153.9, 140.8, 135.3, 129.5, 129.0, 128.4, 116.9, 114.3, 75.6, 73.4, 62.2, 55.7, 51.1, 18.6, 13.7.

HRMS (ESI) m/z: $[M + Na]^+$ Calcd for $C_{21}H_{23}NNaO_5^+$ 392.1468; Found 392.1473.

$[\alpha]^{24}_D$ –31.2 (*c* 1.0, $CHCl_3$).

ethyl (3*R*,4a*R*,8a*R*)-4-(4-methoxyphenyl)-2-oxo-3-phenyloctahydro-2H-benzo[b][1,4]oxazine-3-carboxylate (3ao)

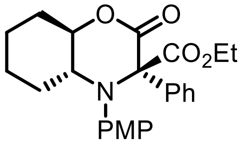

100 °C, 16 h. 22.9 mg, 56% yield, >20:1 d. r., white solid, m.p = 148 – 150 °C.

^{1}H NMR (400 MHz, Chloroform-d) δ 7.91 – 7.84 (m, 2H), 7.48 – 7.37 (m, 3H), 6.98 – 6.55 (m, 4H), 4.24 – 4.15 (m, 1H), 3.95 – 3.85 (m, 1H), 3.81 – 3.73 (m, 2H), 3.71 (s, 3H), 3.70 – 3.65 (m, 1H), 2.49 –2.41 (m, 1H), 2.15 – 2.05 (m, 1H), 1.88 – 1.82 (m, 1H), 1.78 – 1.70 (m, 1H), 1.66 – 1.59 (m, 1H), 1.38 – 1.22 (m, 2H), 0.94 (t, *J* = 7.1 Hz, 3H).

^{13}C NMR (101 MHz, Chloroform-d) δ 167.54, 167.51, 155.5, 138.3, 137.3, 129.4, 128.8, 128.3, 124.0, 113.7, 79.0, 78.2, 62.0, 57.3, 55.5, 30.8, 29.9, 24.1, 23.8, 13.7.

HRMS (ESI) m/z: $[M + Na]^+$ Calcd for $C_{24}H_{27}NNaO_5^+$ 432.1781; Found 432.1790.

$[\alpha]^{24}_D$ +182.5 (*c* 1.0, $CHCl_3$).

(3*S*,4a*S*,9a*R*)-*N*-methoxy-4-(4-methoxyphenyl)-*N*,3-dimethyl-2-oxo-2,3,4,4a,9,9a-hexahydroindeno[2,1-b] [1,4]oxazine-3-carboxamide (3ap)

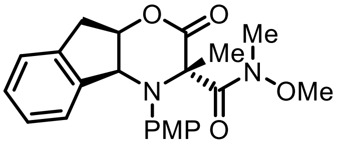

100 °C, 16 h. 25.9 mg, 65% yield, 6:1 d. r., colorless oil.

^{1}H NMR (400 MHz, Chloroform-d) δ 7.49 (d, *J* = 8.9 Hz, 2H), 7.22 –7.12 (m, 2H), 6.99 – 6.93 (m, 1H), 6.87 (d, *J* = 8.9 Hz, 2H), 6.46 (d, *J* = 7.7 Hz, 1H), 5.46 (d, *J* = 5.2 Hz, 1H), 5.09 (td, *J* = 4.7, 2.3 Hz, 1H), 3.83 (s, 3H), 3.68 (s, 3H), 3.31 – 3.27 (m, 2H), 3.24 (s, 3H), 1.44 (s, 3H).

^{13}C NMR (101 MHz, Chloroform-d) δ 172.3, 169.0, 157.5, 141.3, 139.1, 137.4, 130.9, 128.1, 126.9, 125.6, 125.0, 113.8, 79.3, 66.5, 64.0, 61.0, 55.5, 37.9, 33.4, 22.1.

HRMS (ESI) m/z: $[M + Na]^+$ Calcd for $C_{22}H_{24}N_2NaO_5^+$ 419.1577; Found 419.1584.
$[\alpha]^{24}_{D}$ +23.8 (*c* 1.0, $CHCl_3$).

5. Gram-scale reaction

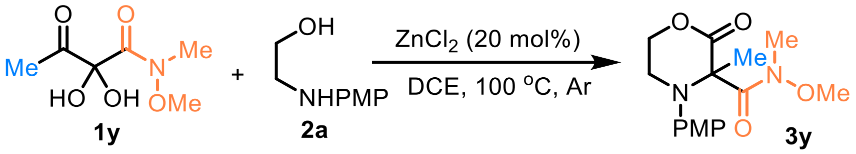

A suspension of **1y** (6.78 mmol, 1.0 equiv), **2a** (8.14 mmol, 1.2 equiv), $ZnCl_2$ (1.36 mmol, 0.2 equiv) in 1,2-dichloroethane (40.0 mL) was stirred in a flask under argon. The reaction mixture was stirred at 100 °C for 48 h. After completion of the reaction (monitored by TLC), the solvent was removed under vacuum. The residue was purified by column chromatography on silica gel eluting with petroleum ether/ethyl acetate to afford the products **3y** in 75% yield (1.57 g) as a yellow solid.

6. Supplementary Table S1: Chiral ligands effect[a]

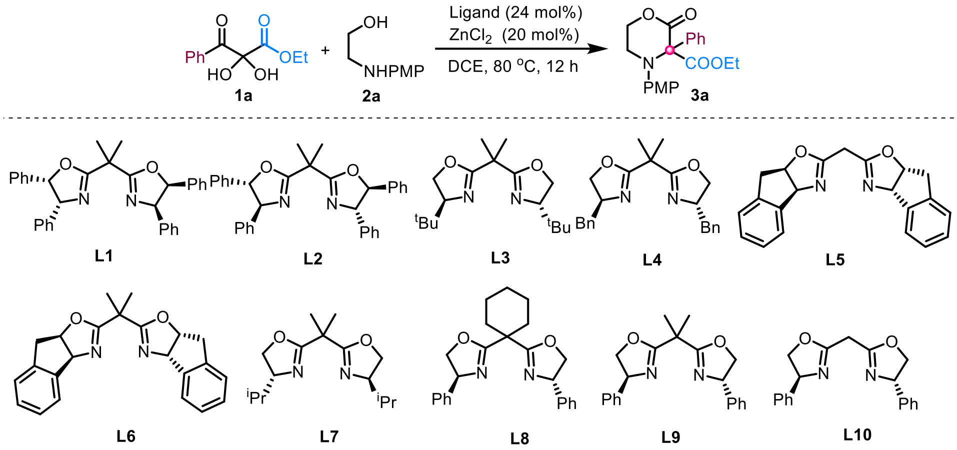

Entry	Ligand	Yield (%)[b]	Ee (%)[c]
1	L1	42	0
2	L2	26	0
3	L3	42	2
4	L4	23	2
5	L5	28	0
6	L6	50	0
7	L7	52	1
8	L8	41	2
9	L9	36	2
10	L10	33	2

[a] Conditions: **1a** (0.10 mmol), **2a** (0.12 mmol), Ligand (0.02 mmol), Chiral Box ligand (0.012 mmol), solvent (*c* 0.1 M), sealed tube, argon, 12 h. [b] Isolated yields. [c] Determined by SFC analysis on a chiral stationary phase.

7. Control experiments

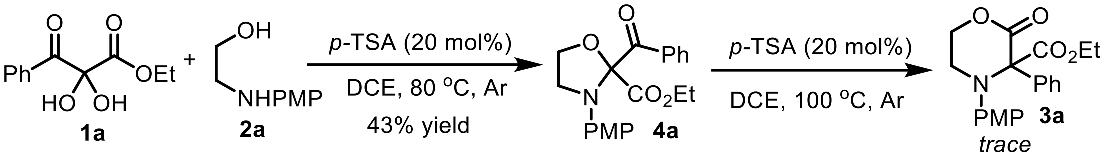

A suspension of **1a** (0.1 mmol, 1.0 equiv), **2a** (0.12 mmol, 1.2 equiv), *p*-TSA (0.02 mmol, 0.2 equiv) in 1,2-dichloroethane (1.0 mL) was stirred in a dry sealed tube under argon. The reaction mixture was stirred at 80 °C for 12 h. After completion of the reaction (monitored by TLC), the solvent was removed under vacuum. The residue was purified by column chromatography on silica gel eluting with petroleum ether/ethyl acetate to afford **4a** in 43% yield (15.1 mg) as a yellow solid.

^{1}H NMR (400 MHz, Chloroform-*d*) δ 8.02 – 7.94 (m, 2H), 7.56 – 7.48 (m, 1H), 7.44 – 7.35 (m, 2H), 6.92 – 6.85 (m, 2H), 6.80 – 6.72 (m, 2H), 4.46 – 4.38 (m, 1H), 4.29 – 4.20 (m, 1H), 4.14 – 3.98 (m, 2H), 3.79 – 3.75 (m, 1H), 3.73 (s, 3H), 3.72 – 3.65 (m, 1H), 1.00 (t, *J* = 7.1 Hz, 3H).

^{13}C NMR (101 MHz, Chloroform-*d*) δ 193.7, 168.8, 154.2, 137.6, 134.9, 133.1, 129.6, 128.3, 118.8, 114.2, 96.4, 67.2, 62.0, 55.6, 49.1, 13.9.

HRMS (ESI) m/z: $[M + Na]^+$ Calcd for $C_{20}H_{21}NNaO_5^+$ 378.1312; Found 378.1313.

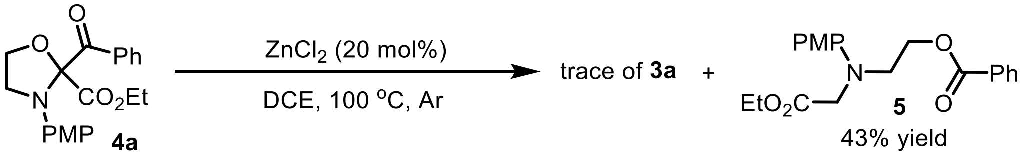

A suspension of **4a** (0.05 mmol, 1.0 equiv), $ZnCl_2$ (0.01 mmol, 0.2 equiv) in 1,2-dichloroethane (0.5 mL) was stirred in a dry sealed tube under argon. The reaction mixture was stirred at 100 °C for 12 h. After completion of the reaction (monitored by TLC), the solvent was removed under vacuum. The residue was purified by column chromatography on silica gel eluting with petroleum ether/ethyl acetate to afford **5** in 43% yield (7.7 mg) as a colorless oil.

^{1}H NMR (400 MHz, Chloroform-*d*) δ 8.03 – 7.98 (m, 2H), 7.60 – 7.52 (m, 1H), 7.47 – 7.40 (m, 2H), 6.83 (d, *J* = 9.1 Hz, 2H), 6.71 (d, *J* = 8.5 Hz, 2H), 4.52 (t, *J* = 6.2 Hz, 2H),

4.15 (q, J = 7.0 Hz, 2H), 4.09 (s, 2H), 3.78 (t, J = 6.0 Hz, 2H), 3.75 (s, 3H), 1.23 (t, J = 7.1 Hz, 3H).

^{13}C NMR (101 MHz, Chloroform-*d*) δ 171.4, 166.7, 152.4, 142.3, 133.2, 130.1, 129.8, 128.5, 115.1, 114.3, 62.8, 61.1, 55.9, 53.9, 51.2, 14.3.

HRMS (ESI) m/z: $[M + H]^+$ Calcd for $C_{20}H_{24}NO_5^+$ 358.1649; Found 358.1647.

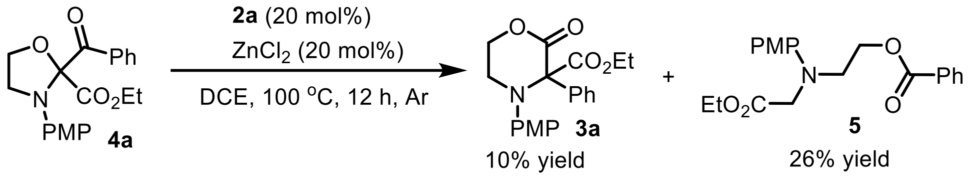

A suspension of **2a** (0.008 mmol, 0.2 equiv), $ZnCl_2$ (0.008 mmol, 0.2 equiv) in 1,2-dichloroethane (0.3 mL) was stirred in a dry sealed tube under argon. The mixture was stirred at 100 °C for 20 min. Then **4a** (0.04 mmol, 1.0 equiv) was added to the mixture. After completion of the reaction (monitored by TLC), the solvent was removed. The residue was purified by column chromatography on silica gel eluting with petroleum ether/ethyl acetate to afford **3a** in 10% yield (1.5 mg) and **5** in 26% yield (3.7 mg).

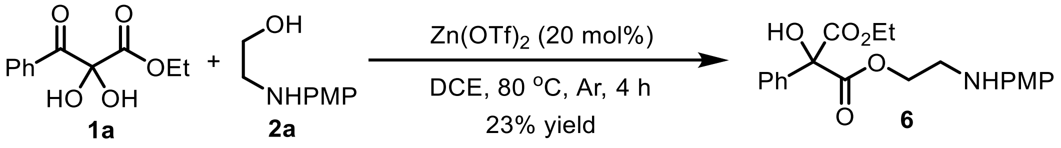

A suspension of **1a** (0.1 mmol, 1.0 equiv), **2a** (0.12 mmol, 1.2 equiv), $Zn(OTf)_2$ (0.02 mmol, 0.2 equiv) in 1,2-dichloroethane (1.0 mL) was stirred in a dry sealed tube under argon. The reaction mixture was stirred at 80 °C for 4 h. After completion of the reaction (monitored by TLC), the solvent was removed under vacuum. The residue was purified by column chromatography on silica gel eluting with petroleum ether/ethyl acetate to afford **6** in 23% yield (8.6 mg) as a colorless oil.

^{1}H NMR (400 MHz, Chloroform-*d*) δ 7.66 – 7.60 (m, 2H), 7.42 – 7.35 (m, 3H), 6.80 – 6.74 (m, 2H), 6.57 – 6.51 (m, 2H), 4.50 – 4.42 (m, 1H), 4.38 – 4.23 (m, 3H), 3.75 (s, 3H), 3.39 – 3.34 (m, 2H), 1.27 (t, J = 7.1 Hz, 3H).

^{13}C NMR (101 MHz, Chloroform-*d*) δ 169.9, 169.8, 152.6, 141.4, 135.9, 128.7, 128.1, 126.5, 114.9, 114.6, 80.2, 65.3, 63.1, 55.8, 43.5, 13.9.

HRMS (ESI) m/z: $[M + Na]^+$ Calcd for $C_{20}H_{23}NNaO_6^+$ 396.1423; Found 396.1422.

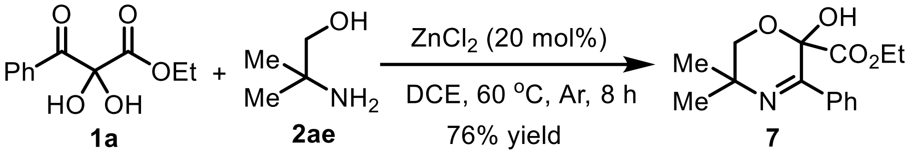

1a 2ae 7

A suspension of **1a** (0.1 mmol, 1.0 equiv), **2ae** (0.12 mmol, 1.2 equiv), $ZnCl_2$ (0.02 mmol, 0.2 equiv) in 1,2-dichloroethane (1.0 mL) was stirred in a dry sealed tube under argon. The reaction mixture was stirred at 60 °C for 8 h. After completion of the reaction (monitored by TLC), the solvent was removed under vacuum. The residue was purified by column chromatography on silica gel eluting with petroleum ether/ethyl acetate to afford **7** in 76% yield (17.2 mg) as a colorless oil.

^{1}H NMR (400 MHz, Chloroform-*d*) δ 7.78 (d, *J* = 7.4 Hz, 2H), 7.40 – 7.28 (m, 3H), 4.43 (brs, 1H), 4.19 – 4.01 (m, 2H), 3.86 (d, *J* = 11.3 Hz, 1H), 3.69 (d, *J* = 11.2 Hz, 1H), 1.34 (s, 3H), 1.30 (s, 3H), 0.97 (t, *J* = 7.0 Hz, 3H).

^{13}C NMR (100 MHz, Chloroform-*d*) δ 169.9, 158.6, 136.6, 129.9, 128.3, 127.6, 89.0, 67.6, 63.1, 53.6, 26.3, 25.0, 13.6.

HRMS (ESI) m/z: $[M + Na]^+$ Calcd for $C_{15}H_{19}NNaO_4^+$ 300.1206; Found 300.1201.

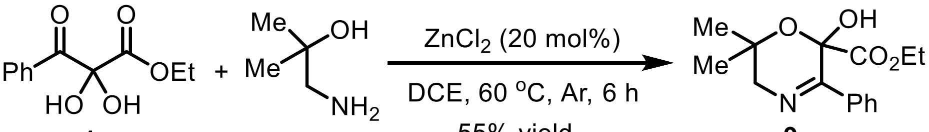

1a 2af 9

A suspension of **1a** (0.1 mmol, 1.0 equiv), **2af** (0.12 mmol, 1.2 equiv), $ZnCl_2$ (0.02 mmol, 0.2 equiv) in 1,2-dichloroethane (1.0 mL) was stirred in a dry sealed tube under argon. The reaction mixture was stirred at 60 °C for 6 h. After completion of the reaction (monitored by TLC), the solvent was removed under vacuum. The residue was purified by column chromatography on silica gel eluting with petroleum ether/ethyl acetate to afford **9** in 55% yield (12.5 mg) as a colorless oil.

^{1}H NMR (400 MHz, Chloroform-*d*) δ 7.83 – 7.77 (m, 2H), 7.41 – 7.30 (m, 3H), 4.48 (brs, 1H), 4.24 – 4.07 (m, 2H), 3.96 (d, *J* = 17.1 Hz, 1H), 3.65 (d, *J* = 17.1 Hz, 1H), 1.36 (s, 3H), 1.31 (s, 3H), 1.05 (t, *J* = 7.0 Hz, 3H).

^{13}C NMR (100 MHz, Chloroform-*d*) δ 170.1, 162.0, 136.2, 130.1, 128.3, 127.7, 89.4,

69.7, 63.0, 58.6, 27.9, 25.1, 13.7.

HRMS (ESI) m/z: $[M + Na]^+$ Calcd for $C_{15}H_{19}NNaO_4^+$ 300.1206; Found 300.1206.

8. Supplementary References

[1] P. M. Truong, P. Y. Zavalij, M. P. Doyle, *Angew. Chem. Int. Ed.* **2014**, *53*, 6468.

[2] P. A. Peixoto, A. Boulangé, S. Leleu, X. Franck, *Eur. J. Org. Chem.* **2013**, 3316.

[3] M. G. Ferlin, G. Chiarelotto, V. Gasparotto, L. Dalla Via, V. Pezzi, L. Barzon, G. Palù, I. Castagliuolo, *J. Med. Chem.* **2005**, *48*, 3417.

[4] H. Yin, M. Jin, W. Chen, C. Chen, L. Zheng, P. Wei, S. Han, *Tetrahedron Lett.* **2012**, *53*, 1265.

Supplementary Notes

9. Crystallographic data for 3a (CCDC 2225996)

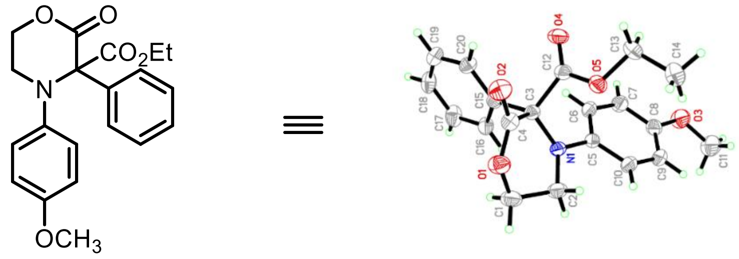

Bond precision:	C-C =0.0025 A		Wavelength=1.54178
Cell:	a=13.6325(6)	b=8.0587(3)	c=17.4146(7)
	alpha=90	beta=108.698(2)	gamma=90
Temperature:	294 K		
	Calculated		Reported
Volume	1812.20(13)		1812.20(13)
Space group	P 21/c		P 21/c
Hall group	-P 2ybc		-P 2ybc
Moiety formula	C20 H21 N O5		C20 H21 N O5
Sum formula	C20 H21 N O5		C20 H21 N O5
Mr	355.38		355.38
Dx,g cm-3	1.303		1.303
Z	4		4
Mu (mm-1)	0.774		0.774
F000	752.0		752.0
F000’	754.46		
h,k,lmax	16,9,20		16,9,20
Nref	3320		3299
Tmin,Tmax	0.843,0.870		0.601,0.753
Tmin’	0.843		

Correction method= # Reported T Limits: Tmin=0.601 Tmax=0.753

AbsCorr = MULTI-SCAN		
Data completeness= 0.994		Theta(max)= 68.273
R(reflections)= 0.0428(2624)		wR2(reflections)= 0.1245(3299)
S = 1.048	Npar= 237	

Supplementary Table S2: Crystal data and structure refinement for t_a.

Identification code	t_a	
Empirical formula	C20H21NO5	
Formula weight	355.38	
Temperature	294(2) K	
Wavelength	1.54178 A	
Crystal system, space group	Monoclinic, P2(1)/c	
Unit cell dimensions	a = 13.6325(6) A	a = 90°
	b = 8.0587(3) A	b = 90°
	c = 17.4146(7) A	c = 90°
Volume	1812.20(13) A^3	
Z, Calculated density	4,1.303 Mg/m^3	
Absorption coefficient	0.774 mm^-1	
F (000)	752	
Crystal size	0.220 x 0.200 x 0.180 mm	
Theta range for data collection	3.423 to 68.273 deg.	
Limiting indices	-15<=h<=16, -9<=k<=9, -20<=l<=20	
Reflections collected / unique	14931 / 3299 [R(int) = 0.0449]	
Completeness to theta = 67.679	99.4 %	
Absorption correction	Semi-empirical from equivalents	
Max. and min. transmission	0.7531 and 0.6013	
Refinement method	Full-matrix least-squares on F^2	
Data / restraints / parameters	3299 / 0 / 237	
Goodness-of-fit on F^2	1.048	
Final R indices [I>2sigma(I)]	R1 = 0.0428, wR2 = 0.1159	
R indices (all data)	R1 = 0.0547, wR2 = 0.1245	
Extinction coefficient	n/a	
Largest diff. peak and hole	0.156 and -0.200 e.A^-3	

Supplementary Table S3: Atomic coordinates (x 10^4) and equivalent isotropic displacement parameters (A^2 x 10^3) for t_a. U(eq) is defined as one third of the trace of the orthogonalized Uij tensor.

	x	y	z	U(eq)
N(1)	7169 (1)	4237 (1)	4250 (1)	42 (1)

C(1)	8459(1)	6291(2)	4857(1)	69(1)
O(2)	9417(1)	2478(2)	5710(1)	60(1)
C(2)	7345(1)	5819(2)	4664(1)	60(1)
O(3)	3117(1)	2311(2)	2540(1)	78(1)
C(3)	7991(1)	2985(2)	4505(1)	39(1)
O(4)	7601(1)	20(2)	4537(1)	64(1)
C(4)	8891(1)	3510(2)	5271(1)	46(1)
O(5)	7116(1)	1796(1)	5335(1)	53(1)
C(5)	6140(1)	3737(2)	3848(1)	40(1)
C(6)	5918(1)	2457(2)	3277(1)	46(1)
C(7)	4912(1)	2025(2)	2861(1)	54(1)
C(8)	4092(1)	2849(2)	2990(1)	52(1)
C(9)	4291(1)	4105(2)	3554(1)	55(1)
C(10)	5310(1)	4527(2)	3983(1)	50(1)
C(11)	2264(2)	3303(3)	2529(2)	91(1)
C(12)	7561(1)	1392(2)	4782(1)	44(1)
C(13)	6607(2)	466(2)	5623(1)	64(1)
C(14)	5938(2)	1258(3)	6043(2)	96(1)
C(15)	8504(1)	2630(2)	3853(1)	39(1)
C(16)	8258(1)	3569(2)	3152(1)	51(1)
C(17)	8741(2)	3255(3)	2578(1)	66(1)
C(18)	9474(1)	2027(3)	2694(1)	62(1)
C(19)	9727(1)	1102(2)	3393(1)	55(1)
C(20)	9248(1)	1391(2)	3967(1)	47(1)
O(1)	9109(1)	5102(2)	5415(1)	63(1)

Supplementary Table S4: Bond lengths [A] and angles [deg] for t_a.

N(1)-C(5)	1.4102(19)
N(1)-C(2)	1.447(2)
N(1)-C(3)	1.4670(18)
C(1)-O(1)	1.447(2)
C(1)-C(2)	1.496(3)
O(2)-C(4)	1.1995(19)
O(3)-C(8)	1.378(2)
O(3)-C(11)	1.407(3)
C(3)-C(15)	1.539(2)
C(3)-C(12)	1.551(2)
C(3)-C(4)	1.554(2)
O(4)-C(12)	1.1931(19)
C(4)-O(1)	1.323(2)
O(5)-C(12)	1.3321(19)
O(5)-C(13)	1.451(2)
C(5)-C(10)	1.384(2)
C(5)-C(6)	1.397(2)

C(6)-C(7)	1.374(2)
C(7)-C(8)	1.380(2)
C(8)-C(9)	1.375(3)
C(9)-C(10)	1.392(2)
C(13)-C(14)	1.485(3)
C(15)-C(16)	1.383(2)
C(15)-C(20)	1.392(2)
C(16)-C(17)	1.385(2)
C(17)-C(18)	1.374(3)
C(18)-C(19)	1.373(3)
C(19)-C(20)	1.378(2)
C(5)-N(1)-C(2)	118.65(12)
C(5)-N(1)-C(3)	119.65(12)
C(2)-N(1)-C(3)	117.81(12)
O(1)-C(1)-C(2)	109.98(17)
N(1)-C(2)-C(1)	109.29(15)
C(8)-O(3)-C(11)	118.12(16)
N(1)-C(3)-C(15)	112.83(12)
N(1)-C(3)-C(12)	109.61(12)
C(15)-C(3)-C(12)	112.47(12)
N(1)-C(3)-C(4)	112.89(12)
C(15)-C(3)-C(4)	105.24(12)
C(12)-C(3)-C(4)	103.38(12)
O(2)-C(4)-O(1)	119.95(15)
O(2)-C(4)-C(3)	120.32(15)
O(1)-C(4)-C(3)	119.65(13)
C(12)-O(5)-C(13)	116.62(14)
C(10)-C(5)-C(6)	117.23(14)
C(10)-C(5)-N(1)	121.44(14)
C(6)-C(5)-N(1)	121.29(13)
C(7)-C(6)-C(5)	121.00(15)
C(6)-C(7)-C(8)	121.04(16)
C(9)-C(8)-O(3)	124.81(16)
C(9)-C(8)-C(7)	119.10(15)
O(3)-C(8)-C(7)	116.08(16)
C(8)-C(9)-C(10)	119.80(16)
C(5)-C(10)-C(9)	121.79(16)
O(4)-C(12)-O(5)	124.72(15)
O(4)-C(12)-C(3)	126.19(15)
O(5)-C(12)-C(3)	109.09(13)
O(5)-C(13)-C(14)	106.91(17)
C(16)-C(15)-C(20)	118.56(15)
C(16)-C(15)-C(3)	120.69(14)

C(20)-C(15)-C(3)	120.72(13)
C(15)-C(16)-C(17)	119.99(16)
C(18)-C(17)-C(16)	121.16(17)
C(19)-C(18)-C(17)	119.02(17)
C(18)-C(19)-C(20)	120.57(17)
C(19)-C(20)-C(15)	120.70(15)
C(4)-O(1)-C(1)	117.54(13)

Symmetry transformations used to generate equivalent atoms:

Supplementary Table S5: Anisotropic displacement parameters (A^2 x 10^3) for t_a. The anisotropic displacement factor exponent takes the form: -2 pi^2 [h^2 a*^2 U11 + ... + 2 h k a* b* U12]

	U^{11}	U^{22}	U^{33}	U^{23}	U^{13}	U^{12}
N(1)	38(1)	33(1)	51(1)	-1(1)	12(1)	3(1)
C(1)	53(1)	39(1)	101(2)	-5(1)	5(1)	-1(1)
O(2)	62(1)	66(1)	43(1)	9(1)	5(1)	9(1)
C(2)	50(1)	40(1)	83(1)	-12(1)	12(1)	2(1)
O(3)	41(1)	70(1)	112(1)	-16(1)	7(1)	-5(1)
C(3)	39(1)	36(1)	41(1)	3(1)	11(1)	4(1)
O(4)	85(1)	35(1)	80(1)	3(1)	39(1)	4(1)
C(4)	46(1)	50(1)	42(1)	0(1)	13(1)	2(1)
O(5)	64(1)	46(1)	55(1)	4(1)	30(1)	-4(1)
C(5)	40(1)	36(1)	43(1)	5(1)	15(1)	3(1)
C(6)	42(1)	45(1)	53(1)	-4(1)	17(1)	3(1)
C(7)	49(1)	51(1)	60(1)	-10(1)	15(1)	-3(1)
C(8)	39(1)	49(1)	66(1)	1(1)	11(1)	-3(1)
C(9)	40(1)	52(1)	75(1)	0(1)	22(1)	7(1)
C(10)	47(1)	46(1)	58(1)	-7(1)	20(1)	3(1)
C(11)	42(1)	90(2)	128(2)	-5(2)	9(1)	3(1)
C(12)	48(1)	39(1)	46(1)	5(1)	15(1)	4(1)
C(13)	72(1)	53(1)	75(1)	15(1)	36(1)	-5(1)
C(14)	108(2)	81(2)	129(2)	16(2)	80(2)	-1(1)
C(15)	36(1)	39(1)	40(1)	2(1)	10(1)	0(1)
C(16)	43(1)	59(1)	53(1)	18(1)	16(1)	7(1)
C(17)	55(1)	94(2)	53(1)	27(1)	22(1)	6(1)
C(18)	49(1)	88(1)	55(1)	0(1)	26(1)	-1(1)
C(19)	47(1)	62(1)	59(1)	-2(1)	20(1)	9(1)
C(20)	49(1)	48(1)	44(1)	4(1)	13(1)	10(1)
O(1)	55(1)	51(1)	71(1)	-10(1)	2(1)	-2(1)

Supplementary Table S6: Hydrogen coordinates (x 10^4) and isotropic displacement parameters (A^2 x 10^3) for t_a.

	x	y	z	U(eq)
H(1A)	8641	6319	4363	83
H(1B)	8571	7389	5097	83
H(2A)	7169	5742	5160	72
H(2B)	6909	6661	4322	72
H(6)	6458	1889	3177	55
H(7)	4783	1164	2486	65
H(9)	3746	4672	3648	66
H(10)	5435	5362	4371	60
H(11A)	1653	2891	2126	136
H(11B)	2388	4427	2402	136
H(11C)	2168	3268	3051	136
H(13A)	7117	-245	5994	77
H(13B)	6193	-201	5172	77
H(14A)	5559	418	6220	144
H(14B)	5461	2001	5677	144
H(14C)	6360	1868	6504	144
H(16)	7767	4410	3066	62
H(17)	8567	3887	2107	79
H(18)	9793	1825	2305	74
H(19)	10226	274	3479	66
H(20)	9424	751	4436	57

Supplementary Table S7: Torsion angles [deg] for t_a.

C(5)-N(1)-C(2)-C(1)	-165.57(15)
C(3)-N(1)-C(2)-C(1)	36.7(2)
O(1)-C(1)-C(2)-N(1)	-63.7(2)
C(5)-N(1)-C(3)-C(15)	90.26(16)
C(2)-N(1)-C(3)-C(15)	-112.22(16)
C(5)-N(1)-C(3)-C(12)	-35.92(18)
C(2)-N(1)-C(3)-C(12)	21.60(15)
C(5)-N(1)-C(3)-C(4)	-150.57(13)
C(2)-N(1)-C(3)-C(4)	6.95(19)
N(1)-C(3)-C(4)-O(2)	154.18(15)
C(15)-C(3)-C(4)-O(2)	-82.35(18)
C(12)-C(3)-C(4)-O(2)	35.82(19)
N(1)-C(3)-C(4)-O(1)	-29.2(2)
C(15)-C(3)-C(4)-O(1)	94.29(17)
C(12)-C(3)-C(4)-O(1)	-147.54(15)
(2)-N(1)-C(5)-C(10)	-14.1(2)
C(3)-N(1)-C(5)-C(10)	143.27(15)
C(2)-N(1)-C(5)-C(6)	163.83(15)
C(3)-N(1)-C(5)-C(6)	-38.8(2)
C(10)-C(5)-C(6)-C(7)	0.9(2)

N(1)-C(5)-C(6)-C(7)	-177.04(15)
C(5)-C(6)-C(7)-C(8)	0.4(3)
C(11)-O(3)-C(8)-C(9)	13.3(3)
C(11)-O(3)-C(8)-C(7)	-167.7(2)
C(6)-C(7)-C(8)-C(9)	-0.9(3)
C(6)-C(7)-C(8)-O(3)	-179.93(17)
O(3)-C(8)-C(9)-C(10)	178.95(17)
C(7)-C(8)-C(9)-C(10)	0.1(3)
C(6)-C(5)-C(10)-C(9)	-1.8(3)
N(1)-C(5)-C(10)-C(9)	176.13(15)
C(8)-C(9)-C(10)-C(5)	1.4(3)
C(13)-O(5)-C(12)-O(4)	-3.1(2)
C(13)-O(5)-C(12)-C(3)	175.93(13)
N(1)-C(3)-C(12)-O(4)	126.26(17)
C(15)-C(3)-C(12)-O(4)	-0.1(2)
C(4)-C(3)-C(12)-O(4)	-113.12(18)
N(1)-C(3)-C(12)-O(5)	-52.72(16)
C(15)-C(3)-C(12)-O(5)	-179.09(12)
C(4)-C(3)-C(12)-O(5)	67.90(15)
C(12)-O(5)-C(13)-C(14)	-164.71(17)
N(1)-C(3)-C(15)-C(16)	6.6(2)
C(12)-C(3)-C(15)-C(16)	131.18(15)
C(4)-C(3)-C(15)-C(16)	-116.96(16)
N(1)-C(3)-C(15)-C(20)	-175.36(13)
C(12)-C(3)-C(15)-C(20)	-50.73(18)
C(4)-C(3)-C(15)-C(20)	61.13(18)
C(20)-C(15)-C(16)-C(17)	0.6(3)
C(3)-C(15)-C(16)-C(17)	178.74(16)
C(15)-C(16)-C(17)-C(18)	-0.5(3)
C(16)-C(17)-C(18)-C(19)	-0.1(3)
C(17)-C(18)-C(19)-C(20)	0.5(3)
C(18)-C(19)-C(20)-C(15)	-0.4(3)
C(16)-C(15)-C(20)-C(19)	-0.2(2)
C(3)-C(15)-C(20)-C(19)	-178.32(15)
O(2)-C(4)-O(1)-C(1)	178.94(17)
C(3)-C(4)-O(1)-C(1)	2.3(2)
C(2)-C(1)-O(1)-C(4)	44.2(2)

Symmetry transformations used to generate equivalent atoms:

Supplementary Table S8: Hydrogen bonds for t_a [A and deg.].

D-H...A	d(D-H)	d(H...A)	d(D...A)	<(DHA)

10. Crystallographic data for 3ag (CCDC 2246927)

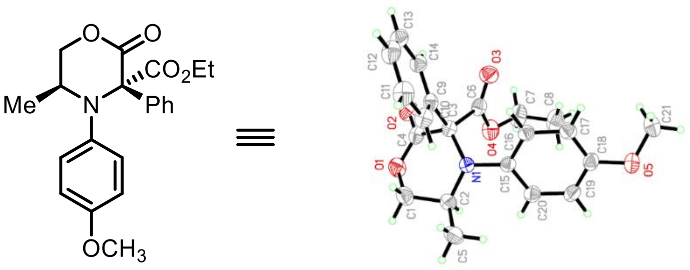

Bond precision:	C-C =0.0028 A		Wavelength=1.54178
Cell:	a=8.3680 (6)	b=13.7815 (3)	c=16.1830 (7)
	alpha=90	beta=90	gamma=90
Temperature:	173 K		

	Calculated	Reported
Volume	1866.28(8)	1866.28(8)
Space group	P 21 21 21	P 21 21 21
Hall group	P 2ac 2ab	P 2ac 2ab
Moiety formula	C21 H23 N O5	C21 H23 N O5
Sum formula	C21 H23 N O5	C21 H23 N O5
Mr	369.40	369.40
Dx,g cm-3	1.315	1.315
Z	4	4
Mu (mm-1)	0.771	0.771
F000	784.0	784.0
F000’	786.52	
h,k,lmax	10,16,19	10,16,19
Nref	3432[1975]	3426
Tmin,Tmax	0.891,0.912	0.630,0.753
Tmin’	0.891	

Correction method= # Reported T Limits: Tmin=0.630 Tmax=0.753

AbsCorr = MULTI-SCAN

Data completeness= 1.73/1.00		Theta(max)= 68.471
R(reflections)= 0.0278(3268)		wR2(reflections)= 0.0759(3426)
S = 1.019	Npar= 247	

Supplementary Table S9: Crystal data and structure refinement for a_a.

Identification code	a_a	
Empirical formula	C21 H23 N O5	
Formula weight	369.40	
Temperature	173(2) K	
Wavelength	1.54178 Å	
Crystal system	Orthorhombic	
Space group	$P2_12_12_1$	
Unit cell dimensions	a = 8.3680(2) Å	a= 90°.
	b = 13.7815(3) Å	b= 90°.
	c = 16.1830(4) Å	g = 90°.
Volume	1866.28(8) Å^3	
Z	4	
Density (calculated)	1.315 Mg/m^3	
Absorption coefficient	0.771 mm^{-1}	
F(000)	784	
Crystal size	0.150 x 0.140 x 0.120 mm^3	
Theta range for data collection	4.213 to 68.471°.	
Index ranges	-9<=h<=10, -16<=k<=16, -19<=l<=19	
Reflections collected	25075	
Independent reflections	3426 [R(int) = 0.0413]	
Completeness to theta = 67.679°	99.8 %	
Absorption correction	Semi-empirical from equivalents	
Max. and min. transmission	0.7531 and 0.6297	
Refinement method	Full-matrix least-squares on F^2	
Data / restraints / parameters	3426 / 0 / 247	
Goodness-of-fit on F^2	1.019	
Final R indices [I>2sigma(I)]	R1 = 0.0278, wR2 = 0.0744	
R indices (all data)	R1 = 0.0296, wR2 = 0.0759	
Absolute structure parameter	0.04(6)	
Extinction coefficient	n/a	
Largest diff. peak and hole	0.158 and -0.151 e.Å^{-3}	

Supplementary Table S10: Atomic coordinates (x 10^4) and equivalent isotropic displacement parameters (A^2 x 10^3) for t_a. U(eq) is defined as one third of the trace of the orthogonalized Uij tensor

	x	y	z	U(eq)
O(2)	7882(2)	1499(1)	1283(1)	34(1)
C(2)	6491(2)	676(1)	3544(1)	30(1)
O(3)	6850(2)	3635(1)	2070(1)	35(1)
C(3)	6198(2)	1945(1)	2426(1)	23(1)
O(4)	8595(2)	2730(1)	2797(1)	34(1)
C(4)	7215(2)	1228(1)	1901(1)	27(1)
O(5)	5791(2)	4596(1)	5669(1)	39(1)
C(5)	5435(3)	262(2)	4230(1)	42(1)
C(6)	7225(2)	2888(1)	2399(1)	26(1)
C(7)	9675(3)	3560(2)	2882(1)	41(1)
C(8)	9231(3)	4165(2)	3605(2)	48(1)
C(9)	4609(2)	2044(1)	1950(1)	24(1)
C(10)	3217(2)	1660(2)	2284(1)	29(1)
C(11)	1781(2)	1710(2)	1854(1)	36(1)
C(12)	1724(2)	2148(2)	1083(1)	38(1)
C(13)	3103(3)	2531(2)	743(1)	35(1)
C(14)	4543(2)	2474(1)	1168(1)	28(1)
C(15)	5881(2)	2390(1)	3891(1)	23(1)
C(16)	4951(2)	3216(1)	3759(1)	24(1)
C(17)	4908(2)	3975(1)	4329(1)	27(1)
C(18)	5763(2)	3903(1)	5061(1)	28(1)
C(19)	6656(2)	3072(2)	5215(1)	30(1)
C(20)	6739(2)	2334(1)	4635(1)	28(1)
C(21)	5384(4)	5553(2)	5424(2)	54(1)
O(1)	7312(2)	301(1)	2125(1)	34(1)
N(1)	5950(2)	1642(1)	3287(1)	24(1)
C(1)	6332(3)	-5(1)	2817(1)	35(1)

Supplementary Table S11: Bond lengths [A] and angles [deg] for a_a.

O(2)-C(4)	1.204(2)
C(2)-N(1)	1.466(2)
C(2)-C(1)	1.511(3)
C(2)-C(5)	1.530(3)

O(3)-C(6)	1.200(2)
C(3)-N(1)	1.469(2)
C(3)-C(9)	1.543(2)
C(3)-C(4)	1.557(2)
C(3)-C(6)	1.559(2)
O(4)-C(6)	1.333(2)
O(4)-C(7)	1.464(2)
C(4)-O(1)	1.330(2)
O(5)-C(18)	1.371(2)
O(5)-C(21)	1.418(3)
C(7)-C(8)	1.485(3)
C(9)-C(10)	1.390(3)
C(9)-C(14)	1.399(3)
C(10)-C(11)	1.391(3)
C(11)-C(12)	1.386(3)
C(12)-C(13)	1.383(3)
C(13)-C(14)	1.389(3)
C(15)-C(16)	1.395(2)
C(15)-C(20)	1.404(3)
C(15)-N(1)	1.422(2)
C(16)-C(17)	1.395(3)
C(17)-C(18)	1.388(3)
C(18)-C(19)	1.390(3)
C(19)-C(20)	1.385(3)
O(1)-C(1)	1.451(2)
N(1)-C(2)-C(1)	108.44(16)
N(1)-C(2)-C(5)	111.45(17)
C(1)-C(2)-C(5)	106.38(17)
N(1)-C(3)-C(9)	112.15(14)
N(1)-C(3)-C(4)	114.52(15)
C(9)-C(3)-C(4)	104.77(14)
N(1)-C(3)-C(6)	109.94(14)
C(9)-C(3)-C(6)	112.76(15)
C(4)-C(3)-C(6)	102.29(14)
C(6)-O(4)-C(7)	116.64(16)
O(2)-C(4)-O(1)	119.76(17)
O(2)-C(4)-C(3)	120.63(18)
O(1)-C(4)-C(3)	119.59(16)

C(18)-O(5)-C(21)	116.39(16)
O(3)-C(6)-O(4)	125.41(18)
O(3)-C(6)-C(3)	125.70(17)
O(4)-C(6)-C(3)	108.89(16)
O(4)-C(7)-C(8)	111.03(18)
C(10)-C(9)-C(14)	118.75(17)
C(10)-C(9)-C(3)	119.62(16)
C(14)-C(9)-C(3)	121.57(16)
C(9)-C(10)-C(11)	120.67(19)
C(12)-C(11)-C(10)	120.15(19)
C(13)-C(12)-C(11)	119.71(18)
C(12)-C(13)-C(14)	120.37(18)
C(13)-C(14)-C(9)	120.36(18)
C(16)-C(15)-C(20)	117.45(16)
C(16)-C(15)-N(1)	120.63(16)
C(20)-C(15)-N(1)	121.92(16)
C(17)-C(16)-C(15)	121.66(17)
C(18)-C(17)-C(16)	119.85(17)
O(5)-C(18)-C(17)	124.81(18)
O(5)-C(18)-C(19)	115.91(17)
C(17)-C(18)-C(19)	119.28(17)
C(20)-C(19)-C(18)	120.63(17)
C(19)-C(20)-C(15)	121.05(17)
C(4)-O(1)-C(1)	117.10(14)
C(15)-N(1)-C(2)	118.45(14)
C(15)-N(1)-C(3)	116.84(14)
C(2)-N(1)-C(3)	118.86(14)
O(1)-C(1)-C(2)	111.70(16)

Symmetry transformations used to generate equivalent atoms:

Supplementary Table S12: Anisotropic displacement parameters (A^2 x 10^3) for t_a. The anisotropic displacement factor exponent takes the form: -2 pi^2 [h^2 a*^2 U11 + ... + 2 h k a* b* U12]

	U^{11}	U^{22}	U^{33}	U^{23}	U^{13}	U^{12}
O(2)	28(1)	46(1)	28(1)	-3(1)	6(1)	2(1)
C(2)	34(1)	24(1)	31(1)	0(1)	0(1)	4(1)
O(3)	36(1)	34(1)	35(1)	5(1)	0(1)	-8(1)
C(3)	20(1)	26(1)	23(1)	-2(1)	1(1)	0(1)

O(4)	21(1)	39(1)	40(1)	-7(1)	-3(1)	-4(1)
C(4)	18(1)	34(1)	28(1)	-6(1)	-2(1)	0(1)
O(5)	58(1)	31(1)	29(1)	-8(1)	1(1)	0(1)
C(5)	64(2)	26(1)	36(1)	2(1)	12(1)	1(1)
C(6)	24(1)	31(1)	24(1)	-4(1)	5(1)	-3(1)
C(7)	26(1)	51(1)	45(1)	-10(1)	3(1)	-16(1)
C(8)	53(1)	49(1)	43(1)	-9(1)	6(1)	-24(1)
C(9)	20(1)	25(1)	28(1)	-7(1)	-1(1)	2(1)
C(10)	22(1)	35(1)	30(1)	-4(1)	1(1)	0(1)
C(11)	20(1)	44(1)	46(1)	-8(1)	1(1)	-1(1)
C(12)	25(1)	43(1)	45(1)	-12(1)	-11(1)	7(1)
C(13)	38(1)	34(1)	32(1)	-4(1)	-9(1)	8(1)
C(14)	27(1)	30(1)	28(1)	-2(1)	-2(1)	2(1)
C(15)	22(1)	25(1)	21(1)	1(1)	3(1)	-2(1)
C(16)	24(1)	26(1)	23(1)	2(1)	1(1)	-1(1)
C(17)	29(1)	26(1)	27(1)	2(1)	5(1)	1(1)
C(18)	33(1)	28(1)	23(1)	-2(1)	6(1)	-5(1)
C(19)	33(1)	35(1)	23(1)	0(1)	-4(1)	-1(1)
C(20)	28(1)	29(1)	28(1)	0(1)	-3(1)	3(1)
C(21)	93(2)	28(1)	43(1)	-9(1)	-3(1)	1(1)
O(1)	35(1)	31(1)	35(1)	-6(1)	7(1)	6(1)
N(1)	27(1)	22(1)	23(1)	-1(1)	-1(1)	1(1)
C(1)	42(1)	27(1)	36(1)	-3(1)	7(1)	0(1)

Supplementary Table S13: Hydrogen coordinates (x 10^4) and isotropic displacement parameters (A^2 x 10^3) for a_a.

	x	y	z	U(eq)
H(2)	7629	705	3731	36
H(5A)	5847	-373	4400	64
H(5B)	4339	190	4025	64
H(5C)	5442	704	4704	64
H(7A)	9630	3959	2374	49
H(7B)	10785	3324	2949	49
H(8A)	10020	4684	3676	72
H(8B)	9206	3761	4103	72
H(8C)	8173	4451	3514	72
H(10)	3246	1359	2813	35
H(11)	836	1444	2088	44
H(12)	742	2184	790	45

H(13)	3065	2834	216	41
H(14)	5488	2729	925	34
H(16)	4332	3263	3268	29
H(17)	4295	4540	4216	33
H(19)	7216	3009	5723	36
H(20)	7386	1782	4742	34
H(21A)	5666	6008	5866	82
H(21B)	4232	5590	5317	82
H(21C)	5971	5723	4921	82
H(1A)	6654	-667	2988	42
H(1B)	5199	-31	2642	42

Supplementary Table S14: Torsion angles [deg] for a_a.

N(1)-C(3)-C(4)-O(2)	-158.68(16)
C(9)-C(3)-C(4)-O(2)	78.0(2)
C(6)-C(3)-C(4)-O(2)	-39.8(2)
N(1)-C(3)-C(4)-O(1)	23.1(2)
C(9)-C(3)-C(4)-O(1)	-100.16(18)
C(6)-C(3)-C(4)-O(1)	142.01(17)
C(7)-O(4)-C(6)-O(3)	4.3(3)
C(7)-O(4)-C(6)-C(3)	-176.13(15)
N(1)-C(3)-C(6)-O(3)	-124.12(19)
C(9)-C(3)-C(6)-O(3)	1.8(3)
C(4)-C(3)-C(6)-O(3)	113.8(2)
N(1)-C(3)-C(6)-O(4)	56.33(18)
C(9)-C(3)-C(6)-O(4)	-177.72(14)
C(4)-C(3)-C(6)-O(4)	-65.73(17)
C(6)-O(4)-C(7)-C(8)	83.9(2)
N(1)-C(3)-C(9)-C(10)	-13.7(2)
C(4)-C(3)-C(9)-C(10)	111.13(18)
C(6)-C(3)-C(9)-C(10)	-138.43(17)
N(1)-C(3)-C(9)-C(14)	169.35(15)
C(4)-C(3)-C(9)-C(14)	-65.9(2)
C(6)-C(3)-C(9)-C(14)	44.6(2)
C(14)-C(9)-C(10)-C(11)	-0.7(3)
C(3)-C(9)-C(10)-C(11)	-177.73(17)
C(9)-C(10)-C(11)-C(12)	-0.1(3)
C(10)-C(11)-C(12)-C(13)	0.2(3)

C(11)-C(12)-C(13)-C(14)	0.3(3)
C(12)-C(13)-C(14)-C(9)	-1.1(3)
C(10)-C(9)-C(14)-C(13)	1.2(3)
C(3)-C(9)-C(14)-C(13)	178.25(17)
C(20)-C(15)-C(16)-C(17)	2.0(3)
N(1)-C(15)-C(16)-C(17)	-177.75(16)
C(15)-C(16)-C(17)-C(18)	-2.2(3)
C(21)-O(5)-C(18)-C(17)	-22.0(3)
C(21)-O(5)-C(18)-C(19)	158.7(2)
C(16)-C(17)-C(18)-O(5)	-179.14(17)
C(16)-C(17)-C(18)-C(19)	0.2(3)
O(5)-C(18)-C(19)-C(20)	-178.61(18)
C(17)-C(18)-C(19)-C(20)	2.0(3)
C(18)-C(19)-C(20)-C(15)	-2.2(3)
C(16)-C(15)-C(20)-C(19)	0.2(3)
N(1)-C(15)-C(20)-C(19)	179.96(17)
O(2)-C(4)-O(1)-C(1)	-172.85(17)
C(3)-C(4)-O(1)-C(1)	5.4(2)
C(16)-C(15)-N(1)-C(2)	-160.61(17)
C(20)-C(15)-N(1)-C(2)	19.7(3)
C(16)-C(15)-N(1)-C(3)	46.6(2)
C(20)-C(15)-N(1)-C(3)	-133.16(18)
C(1)-C(2)-N(1)-C(15)	175.42(15)
C(5)-C(2)-N(1)-C(15)	58.6(2)
C(1)-C(2)-N(1)-C(3)	-32.3(2)
C(5)-C(2)-N(1)-C(3)	-149.09(17)
C(9)-C(3)-N(1)-C(15)	-95.10(17)
C(4)-C(3)-N(1)-C(15)	145.67(16)
C(6)-C(3)-N(1)-C(15)	31.2(2)
C(9)-C(3)-N(1)-C(2)	112.20(17)
C(4)-C(3)-N(1)-C(2)	-7.0(2)
C(6)-C(3)-N(1)-C(2)	-121.51(17)
C(4)-O(1)-C(1)-C(2)	-48.6(2)
N(1)-C(2)-C(1)-O(1)	61.2(2)
C(5)-C(2)-C(1)-O(1)	-178.85(18)

Symmetry transformations used to generate equivalent atoms:

Supplementary Table S15: Hydrogen bonds for t_a [A and deg.].

D-H...A	d(D-H)	d(H...A)	d(D...A)	<(DHA)

11. Crystallographic data for 3am (CCDC 2246928)

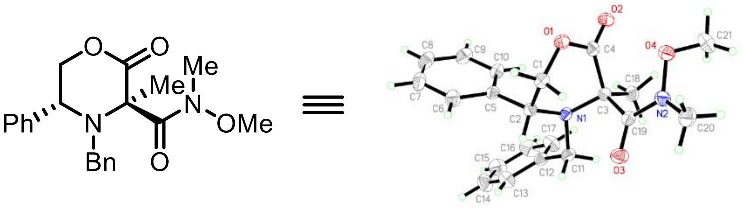

Bond precision:	C-C =0.0043 A		Wavelength=1.54178
Cell:	a=11.1720(3)	b=6.5053(2)	c=13.2819(4)
	alpha=90	beta=104.262(2)	gamma=90
Temperature:	173 K		
	Calculated		Reported
Volume	935.54(5)		935.54(5)
Space group	P 21		P 21
Hall group	P 2yb		P 2yb
Moiety formula	C21 H24 N2 O4		C21 H24 N2 O4
Sum formula	C21 H24 N2 O4		C21 H24 N2 O4
Mr	368.42		368.42
Dx,g cm-3	1.308		1.308
Z	2		2
Mu (mm-1)	0.741		0.741
F000	392.0		392.0
F000’	393.22		
h,k,lmax	13,7,16		13,7,16
Nref	3459[1894]		3425
Tmin,Tmax	0.875,0.901		0.648,0.753
Tmin’	0.875		

Correction method= # Reported T Limits: Tmin=0.648 Tmax=0.753

AbsCorr = MULTI-SCAN

Data completeness= 1.81/0.99 Theta(max)= 68.521

		wR2(reflections)=
R(reflections)= 0.0356(3021)		0.0900(3425)
S = 1.019	Npar= 247	

Supplementary Table S16: Crystal data and structure refinement for t_a.

Identification code	t_a	
Empirical formula	C24H28N2O3	
Formula weight	368.42	
Temperature	173(2) K	
Wavelength	1.54178 Å	
Crystal system, space group	Monoclinic, $P2_1$	
Unit cell dimensions	a = 11.1720(3) Å	a= 90°
	b = 6.5053(2) Å	b=104.262(2)°
	c = 13.2819(4) Å	g = 90°
Volume	935.54(5) $Å^3$	
Z, Calculated density	2, 1.308 Mg/m^3	
Absorption coefficient	0.741 mm^{-1}	
F (000)	392	
Crystal size	0.180 x 0.160 x 0.140 mm^3	
Theta range for data collection	3.433 to 68.521°.	
Limiting indices	-13<=h<=12, -7<=k<=7, -16<=l<=16	
Reflections collected / unique	10494 / 3425 [R(int) = 0.0488]	
Completeness to theta = 67.679	99.9 %	
Absorption correction	Semi-empirical from equivalents	
Max. and min. transmission	0.7531 and 0.6481	
Refinement method	Full-matrix least-squares on F^2	
Data / restraints / parameters	3425 / 1 / 247	
Goodness-of-fit on F^2	1.029	
Final R indices [I>2sigma(I)]	R1 = 0.0356, wR2 = 0.0857	
R indices (all data)	R1 = 0.0434, wR2 = 0.0900	
Absolute structure parameter	0.09(12)	
Extinction coefficient	n/a	
Largest diff. peak and hole	0.125 and -0.204 e.$Å^{-3}$	

Supplementary Table S17: Atomic coordinates (x 10^4) and equivalent isotropic displacement parameters (A^2 x 10^3) for t_a. U(eq) is defined as one third of the trace of the orthogonalized Uij tensor.

	x	y	z	U(eq)
C(9)	8231(2)	4654(5)	10189(2)	39(1)
C(10)	7905(2)	4651(5)	9105(2)	31(1)

C(11)	5643(2)	3690(5)	6589(2)	29(1)
C(12)	5038(2)	3714(5)	7491(2)	28(1)
C(13)	4882(3)	1928(5)	8006(2)	40(1)
C(14)	4293(3)	1962(6)	8816(3)	49(1)
C(15)	3853(3)	3785(6)	9104(3)	50(1)
C(16)	3999(3)	5574(6)	8596(3)	53(1)
C(17)	4591(3)	5544(5)	7793(3)	41(1)
C(18)	6490(2)	7626(4)	5876(2)	31(1)
C(19)	7336(2)	4595(4)	5100(2)	28(1)
C(20)	8119(3)	4350(5)	3514(2)	39(1)
C(21)	8469(3)	8843(5)	4219(3)	44(1)
O(1)	9453(2)	5092(3)	7151(1)	28(1)
N(1)	6935(2)	4427(3)	6881(2)	24(1)
C(1)	9010(2)	2998(4)	7043(2)	28(1)
O(2)	8891(2)	8328(3)	6897(1)	32(1)
N(2)	7941(2)	5415(4)	4431(2)	33(1)
C(2)	7807(2)	2822(4)	7385(2)	25(1)
O(3)	6729(2)	3027(3)	4874(2)	37(1)
C(3)	7342(2)	5766(4)	6138(2)	25(1)
O(4)	8806(2)	6974(3)	4801(2)	34(1)
C(4)	8620(2)	6546(4)	6736(2)	26(1)
C(5)	8068(2)	2893(4)	8569(2)	26(1)
C(6)	8544(3)	1140(5)	9127(2)	36(1)
C(7)	8869(3)	1157(5)	10204(2)	42(1)
C(8)	8715(3)	2922(6)	10733(2)	42(1)

Supplementary Table S18: Bond lengths [A] and angles [deg] for t_a.

C(9)-C(8)	1.375(5)
C(9)-C(10)	1.395(4)
C(10)-C(5)	1.383(4)
C(11)-N(1)	1.479(3)
C(11)-C(12)	1.513(3)
C(12)-C(13)	1.381(4)
C(12)-C(17)	1.387(4)
C(13)-C(14)	1.392(4)
C(14)-C(15)	1.373(5)
C(15)-C(16)	1.376(5)
C(16)-C(17)	1.386(4)

C(18)-C(3)	1.526(4)
C(19)-O(3)	1.220(3)
C(19)-N(2)	1.352(3)
C(19)-C(3)	1.573(4)
C(20)-N(2)	1.457(3)
C(21)-O(4)	1.441(4)
O(1)-C(4)	1.346(3)
O(1)-C(1)	1.444(3)
N(1)-C(3)	1.470(3)
N(1)-C(2)	1.472(3)
C(1)-C(2)	1.525(3)
O(2)-C(4)	1.204(3)
N(2)-O(4)	1.404(3)
C(2)-C(5)	1.528(4)
C(3)-C(4)	1.539(3)
C(5)-C(6)	1.393(4)
C(6)-C(7)	1.386(4)
C(7)-C(8)	1.379(5)
C(8)-C(9)-C(10)	120.7(3)
C(5)-C(10)-C(9)	119.9(3)
N(1)-C(11)-C(12)	112.7(2)
C(13)-C(12)-C(17)	118.7(2)
C(13)-C(12)-C(11)	121.3(3)
C(17)-C(12)-C(11)	120.0(3)
C(12)-C(13)-C(14)	120.7(3)
C(15)-C(14)-C(13)	119.9(3)
C(14)-C(15)-C(16)	120.0(3)
C(15)-C(16)-C(17)	120.2(3)
C(16)-C(17)-C(12)	120.6(3)
O(3)-C(19)-N(2)	120.3(2)
O(3)-C(19)-C(3)	120.2(2)
N(2)-C(19)-C(3)	119.3(2)
C(4)-O(1)-C(1)	115.85(19)
C(3)-N(1)-C(2)	116.44(19)
C(3)-N(1)-C(11)	117.4(2)
C(2)-N(1)-C(11)	112.6(2)
O(1)-C(1)-C(2)	110.3(2)
C(19)-N(2)-O(4)	117.9(2)

C(19)-N(2)-C(20)	123.7(2)
O(4)-N(2)-C(20)	114.4(2)
N(1)-C(2)-C(1)	110.2(2)
N(1)-C(2)-C(5)	112.5(2)
C(1)-C(2)-C(5)	110.2(2)
N(1)-C(3)-C(18)	110.1(2)
N(1)-C(3)-C(4)	104.47(19)
C(18)-C(3)-C(4)	107.7(2)
N(1)-C(3)-C(19)	111.5(2)
C(18)-C(3)-C(19)	108.4(2)
C(4)-C(3)-C(19)	114.56(19)
N(2)-O(4)-C(21)	110.8(2)
O(2)-C(4)-O(1)	119.0(2)
O(2)-C(4)-C(3)	124.7(2)
O(1)-C(4)-C(3)	116.1(2)
C(10)-C(5)-C(6)	119.0(2)
C(10)-C(5)-C(2)	122.7(2)
C(6)-C(5)-C(2)	118.3(2)
C(7)-C(6)-C(5)	120.8(3)
C(8)-C(7)-C(6)	119.9(3)
C(9)-C(8)-C(7)	119.8(3)

Symmetry transformations used to generate equivalent atoms:

Supplementary Table S19: Anisotropic displacement parameters (A^2 x 10^3) for t_a. The anisotropic displacement factor exponent takes the form: -2 pi^2 [h^2 a*^2 U11 + ... + 2 h k a* b* U12]

	U^{11}	U^{22}	U^{33}	U^{23}	U^{13}	U^{12}
C(9)	33(1)	55(2)	30(1)	-7(2)	10(1)	1(1)
C(10)	30(1)	37(2)	26(1)	1(1)	6(1)	3(1)
C(11)	24(1)	36(2)	26(1)	-2(1)	4(1)	-3(1)
C(12)	22(1)	33(1)	29(1)	-1(1)	4(1)	-1(1)
C(13)	41(2)	34(2)	48(2)	2(1)	18(1)	1(1)
C(14)	50(2)	54(2)	48(2)	13(2)	20(2)	-6(2)
C(15)	47(2)	65(2)	46(2)	-5(2)	25(2)	-8(2)
C(16)	53(2)	50(2)	66(2)	-15(2)	35(2)	-1(2)
C(17)	39(1)	35(2)	54(2)	1(2)	22(1)	2(1)
C(18)	31(1)	31(2)	32(1)	6(1)	8(1)	4(1)
C(19)	27(1)	31(2)	23(1)	0(1)	5(1)	1(1)

C(20)	46(2)	47(2)	28(1)	-6(1)	15(1)	-1(1)
C(21)	49(2)	37(2)	53(2)	10(2)	23(2)	1(1)
O(1)	25(1)	29(1)	30(1)	-3(1)	7(1)	0(1)
N(1)	21(1)	30(1)	22(1)	2(1)	4(1)	1(1)
C(1)	28(1)	27(1)	28(1)	-2(1)	7(1)	3(1)
O(2)	35(1)	26(1)	34(1)	-6(1)	7(1)	-5(1)
N(2)	39(1)	35(1)	27(1)	-3(1)	13(1)	-9(1)
C(2)	25(1)	25(1)	24(1)	-2(1)	6(1)	0(1)
O(3)	42(1)	42(1)	31(1)	-10(1)	14(1)	-13(1)
C(3)	28(1)	26(1)	22(1)	-2(1)	7(1)	-1(1)
O(4)	33(1)	35(1)	34(1)	-1(1)	9(1)	-6(1)
C(4)	29(1)	29(2)	21(1)	-2(1)	11(1)	0(1)
C(5)	21(1)	32(2)	26(1)	2(1)	6(1)	0(1)
C(6)	35(1)	36(2)	36(2)	5(1)	10(1)	6(1)
C(7)	35(1)	54(2)	34(2)	17(2)	6(1)	7(1)
C(8)	30(1)	73(2)	24(1)	5(2)	8(1)	-1(1)

Supplementary Table S20: Hydrogen coordinates (x 10^4) and isotropic displacement parameters (A^2 x 10^3) for t_a.

	x	y	z	U(eq)
H(9)	8117	5864	10554	46
H(10)	7572	5855	8736	38
H(11A)	5627	2270	6318	35
H(11B)	5159	4568	6025	35
H(13)	5179	663	7806	48
H(14)	4197	727	9169	59
H(15)	3446	3809	9654	61
H(16)	3695	6834	8795	63
H(17)	4691	6786	7446	49
H(18A)	6874	8665	5523	47
H(18B)	6348	8208	6517	47
H(18C)	5699	7200	5419	47
H(20A)	7571	3153	3370	59
H(20B)	8979	3896	3638	59
H(20C)	7927	5284	2917	59
H(21A)	9062	9928	4511	67
H(21B)	7640	9265	4258	67
H(21C)	8477	8609	3492	67

H(1A)	9640	2078	7472	33
H(1B)	8871	2563	6309	33
H(2)	7431	1455	7149	30
H(6)	8648	-82	8766	43
H(7)	9197	-46	10577	50
H(8)	8942	2943	11471	50

Supplementary Table S21: Torsion angles [deg] for t_a.

C(8)-C(9)-C(10)-C(5)	0.1(4)
N(1)-C(11)-C(12)-C(13)	-102.9(3)
N(1)-C(11)-C(12)-C(17)	79.2(3)
C(17)-C(12)-C(13)-C(14)	-0.3(4)
C(11)-C(12)-C(13)-C(14)	-178.2(3)
C(12)-C(13)-C(14)-C(15)	0.5(5)
C(13)-C(14)-C(15)-C(16)	-0.4(5)
C(14)-C(15)-C(16)-C(17)	0.0(5)
C(15)-C(16)-C(17)-C(12)	0.2(5)
C(13)-C(12)-C(17)-C(16)	0.0(4)
C(11)-C(12)-C(17)-C(16)	177.9(3)
C(12)-C(11)-N(1)-C(3)	-139.2(2)
C(12)-C(11)-N(1)-C(2)	81.5(3)
C(4)-O(1)-C(1)-C(2)	-49.5(3)
O(3)-C(19)-N(2)-O(4)	-168.1(2)
C(3)-C(19)-N(2)-O(4)	16.3(3)
O(3)-C(19)-N(2)-C(20)	-12.1(4)
C(3)-C(19)-N(2)-C(20)	172.3(2)
C(3)-N(1)-C(2)-C(1)	2.9(3)
C(11)-N(1)-C(2)-C(1)	142.6(2)
C(3)-N(1)-C(2)-C(5)	126.4(2)
C(11)-N(1)-C(2)-C(5)	-94.0(2)
O(1)-C(1)-C(2)-N(1)	49.6(3)
O(1)-C(1)-C(2)-C(5)	-75.2(3)
C(2)-N(1)-C(3)-C(18)	-167.0(2)
C(11)-N(1)-C(3)-C(18)	55.3(3)
C(2)-N(1)-C(3)-C(4)	-51.6(3)
C(11)-N(1)-C(3)-C(4)	170.7(2)
C(2)-N(1)-C(3)-C(19)	72.6(3)
C(11)-N(1)-C(3)-C(19)	-65.1(3)

O(3)-C(19)-C(3)-N(1)	19.4(3)
N(2)-C(19)-C(3)-N(1)	-165.0(2)
O(3)-C(19)-C(3)-C(18)	-102.0(3)
N(2)-C(19)-C(3)-C(18)	73.7(3)
O(3)-C(19)-C(3)-C(4)	137.8(3)
N(2)-C(19)-C(3)-C(4)	-46.6(3)
C(19)-N(2)-O(4)-C(21)	-120.0(3)
C(20)-N(2)-O(4)-C(21)	81.7(3)
C(1)-O(1)-C(4)-O(2)	172.1(2)
C(1)-O(1)-C(4)-C(3)	-4.2(3)
N(1)-C(3)-C(4)-O(2)	-121.5(3)
C(18)-C(3)-C(4)-O(2)	-4.4(3)
C(19)-C(3)-C(4)-O(2)	116.3(3)
N(1)-C(3)-C(4)-O(1)	54.6(3)
C(18)-C(3)-C(4)-O(1)	171.7(2)
C(19)-C(3)-C(4)-O(1)	-67.6(3)
C(9)-C(10)-C(5)-C(6)	0.8(4)
C(9)-C(10)-C(5)-C(2)	-176.1(2)
N(1)-C(2)-C(5)-C(10)	-23.1(3)
C(1)-C(2)-C(5)-C(10)	100.4(3)
N(1)-C(2)-C(5)-C(6)	160.1(2)
C(1)-C(2)-C(5)-C(6)	-76.5(3)
C(10)-C(5)-C(6)-C(7)	-1.0(4)
C(2)-C(5)-C(6)-C(7)	176.0(2)
C(5)-C(6)-C(7)-C(8)	0.4(4)
C(10)-C(9)-C(8)-C(7)	-0.7(4)
C(6)-C(7)-C(8)-C(9)	0.5(4)

Symmetry transformations used to generate equivalent atoms:

Supplementary Table S22: Hydrogen bonds for t_a [A and deg.].

D-H...A	d(D-H)	d(H...A)	d(D...A)	<(DHA)

12. Crystallographic data for 3an (CCDC 2246926)

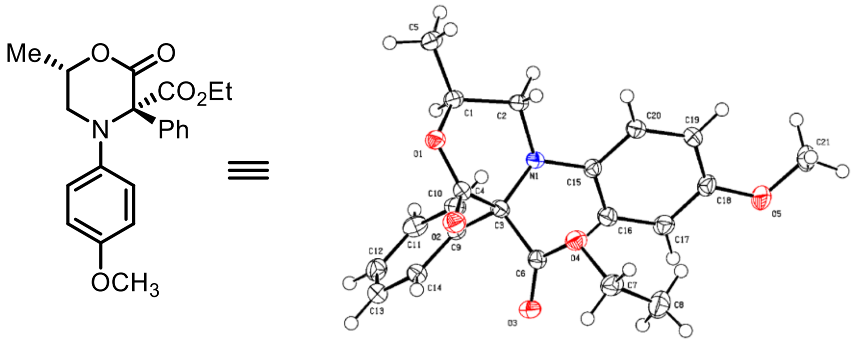

Bond precision:	C-C =0.0030 A		Wavelength=1.54178
Cell:	a=7.5177(2)	b=15.0878(5)	c=15.9713(5)
	alpha=90	beta=90	gamma=90
Temperature:	173 K		

	Calculated	Reported
Volume	1811.55(10)	1811.55(10)
Space group	P 21 21 21	P 21 21 21
Hall group	P 2ac 2ab	P 2ac 2ab
Moiety formula	C21 H23 N O5	C21 H23 N O5
Sum formula	C21 H23 N O5	C21 H23 N O5
Mr	369.40	369.40
Dx,g cm-3	1.355	1.355
Z	4	4
Mu (mm-1)	0.794	0.794
F000	784.0	784.0
F000’	786.52	
h,k,lmax	9,18,19	9,18,19
Nref	3317[1919]	3313
Tmin,Tmax	0.888,0.909	0.695,0.733
Tmin’	0.888	

Correction method= # Reported T Limits: Tmin=0.695 Tmax=0.733

AbsCorr = MULTI-SCAN

Data completeness= 1.73/1.00 Theta(max)= 68.207

R(reflections)= 0.0345(3120) wR2(reflections)= 0.0901(3313)

S = 1.047 Npar= 247

Supplementary Table S23: Crystal data and structure refinement for t_a.

Identification code	t_a	
Empirical formula	C21H23NO5	
Formula weight	369.40	
Temperature	173(2) K	
Wavelength	1.54178 Å	
Crystal system, space group	Orthorhombic, P2(1)2(1)2(1)	
Unit cell dimensions	a = 7.5177(2) Å	a = 90°
	b = 15.0878(5) Å	b = 90°
	c = 15.9713(5) Å	γ = 90°
Volume	1811.55(10) Å^3	
Z, Calculated density	4, 1.354 Mg/m^3	
Absorption coefficient	0.794 mm^{-1}	
F (000)	784	
Crystal size	0.150 x 0.140 x 0.120 mm^3	
Theta range for data collection	4.030 to 68.207°	
Limiting indices	-9<=h<=9, -18<=k<=18, -19<=l<=19	
Reflections collected / unique	21518 / 3313 [R(int) = 0.0819]	
Completeness to theta = 67.679	100.0 %	
Absorption correction	Semi-empirical from equivalents	
Max. and min. transmission	0.7333 and 0.6950	
Refinement method	Full-matrix least-squares on F^2	
Data / restraints / parameters	3313 / 0 / 247	
Goodness-of-fit on F^2	1.047	
Final R indices [I>2sigma(I)]	R1 = 0.0345, wR2 = 0.0871	
R indices (all data)	R1 = 0.0374, wR2 = 0.0901	
Absolute structure parameter	-0.04(9)	
Extinction coefficient	n/a	
Largest diff. peak and hole	0.172 and -0.275 e.Å^{-3}	

Supplementary Table S24: Atomic coordinates (x 10^4) and equivalent isotropic displacement parameters (A^2 x 10^3) for t_a. U(eq) is defined as one third of the trace of the orthogonalized Uij tensor.

	x	y	z	U(eq)
O(3)	8680(2)	4425(1)	4217(1)	28(1)
C(3)	7144(3)	5330(1)	5255(1)	20(1)

O(4)	9523(2)	5841(1)	4400(1)	24(1)
C(4)	8286(3)	5401(1)	6059(1)	21(1)
O(5)	5214(3)	7409(1)	1841(1)	33(1)
C(5)	5658(3)	6830(2)	7383(1)	28(1)
C(6)	8518(3)	5134(2)	4554(1)	21(1)
C(7)	10914(3)	5719(2)	3772(2)	28(1)
C(8)	10172(4)	5756(2)	2896(2)	36(1)
C(9)	5927(3)	4517(1)	5402(1)	19(1)
C(10)	4083(3)	4590(2)	5353(1)	23(1)
C(11)	3008(3)	3856(2)	5502(2)	27(1)
C(12)	3756(3)	3046(2)	5703(2)	27(1)
C(13)	5592(3)	2971(2)	5760(2)	26(1)
C(14)	6670(3)	3701(2)	5620(1)	22(1)
C(15)	5924(3)	6490(1)	4292(1)	19(1)
C(16)	5559(3)	5920(1)	3617(1)	22(1)
C(17)	5339(3)	6244(2)	2812(1)	25(1)
C(18)	5460(3)	7153(2)	2658(1)	24(1)
C(19)	5792(3)	7731(1)	3317(2)	25(1)
C(20)	6031(3)	7396(2)	4127(1)	22(1)
C(21)	5347(4)	8332(2)	1660(2)	35(1)
O(1)	7535(2)	5769(1)	6732(1)	23(1)
N(1)	6146(3)	6153(1)	5107(1)	21(1)
C(1)	5868(3)	6254(2)	6616(1)	22(1)
O(2)	9765(2)	5104(1)	6103(1)	27(1)
C(2)	6025(3)	6768(1)	5807(1)	21(1)

Supplementary Table S25: Bond lengths [A] and angles [deg] for t_a.

O(3)-C(6)	1.205(3)
C(3)-N(1)	1.470(3)
C(3)-C(9)	1.548(3)
C(3)-C(4)	1.548(3)
C(3)-C(6)	1.552(3)
O(4)-C(6)	1.330(3)
O(4)-C(7)	1.461(3)
C(4)-O(2)	1.202(3)
C(4)-O(1)	1.335(3)
O(5)-C(18)	1.374(3)
O(5)-C(21)	1.425(3)

C(5)-C(1)	1.510(3)
C(7)-C(8)	1.506(3)
C(9)-C(10)	1.393(3)
C(9)-C(14)	1.396(3)
C(10)-C(11)	1.391(3)
C(11)-C(12)	1.383(4)
C(12)-C(13)	1.387(4)
C(13)-C(14)	1.386(3)
C(15)-C(20)	1.395(3)
C(15)-C(16)	1.406(3)
C(15)-N(1)	1.408(3)
C(16)-C(17)	1.386(3)
C(17)-C(18)	1.396(3)
C(18)-C(19)	1.391(3)
C(19)-C(20)	1.400(3)
O(1)-C(1)	1.464(3)
N(1)-C(2)	1.456(3)
C(1)-C(2)	1.511(3)
N(1)-C(3)-C(9)	113.08(18)
N(1)-C(3)-C(4)	110.94(17)
C(9)-C(3)-C(4)	104.95(17)
N(1)-C(3)-C(6)	112.60(17)
C(9)-C(3)-C(6)	110.61(17)
C(4)-C(3)-C(6)	104.02(17)
C(6)-O(4)-C(7)	115.62(17)
O(2)-C(4)-O(1)	120.0(2)
O(2)-C(4)-C(3)	122.4(2)
O(1)-C(4)-C(3)	117.56(18)
C(18)-O(5)-C(21)	117.2(2)
O(3)-C(6)-O(4)	124.9(2)
O(3)-C(6)-C(3)	124.0(2)
O(4)-C(6)-C(3)	111.03(18)
O(4)-C(7)-C(8)	111.6(2)
C(10)-C(9)-C(14)	118.8(2)
C(10)-C(9)-C(3)	121.2(2)
C(14)-C(9)-C(3)	119.97(19)
C(9)-C(10)-C(11)	120.4(2)
C(12)-C(11)-C(10)	120.4(2)

C(11)-C(12)-C(13)	119.5(2)
C(14)-C(13)-C(12)	120.4(2)
C(13)-C(14)-C(9)	120.5(2)
C(20)-C(15)-C(16)	117.8(2)
C(20)-C(15)-N(1)	121.44(19)
C(16)-C(15)-N(1)	120.77(19)
C(17)-C(16)-C(15)	121.2(2)
C(16)-C(17)-C(18)	120.2(2)
O(5)-C(18)-C(19)	124.5(2)
O(5)-C(18)-C(17)	115.8(2)
C(19)-C(18)-C(17)	119.7(2)
C(18)-C(19)-C(20)	119.7(2)
C(15)-C(20)-C(19)	121.4(2)
C(4)-O(1)-C(1)	117.82(16)
C(15)-N(1)-C(2)	118.19(17)
C(15)-N(1)-C(3)	120.95(17)
C(2)-N(1)-C(3)	116.57(17)
O(1)-C(1)-C(5)	105.88(17)
O(1)-C(1)-C(2)	107.39(17)
C(5)-C(1)-C(2)	113.96(18)
N(1)-C(2)-C(1)	109.48(17)

Symmetry transformations used to generate equivalent atoms:

Supplementary Table S26: Anisotropic displacement parameters (A^2 x 10^3) for t_a. The anisotropic displacement factor exponent takes the form: -2 pi^2 [h^2 a*^2 U11 + ... + 2 h k a* b* U12]

	U^{11}	U^{22}	U^{33}	U^{23}	U^{13}	U^{12}
O(3)	37(1)	21(1)	26(1)	-4(1)	9(1)	2(1)
C(3)	24(1)	16(1)	20(1)	0(1)	-2(1)	3(1)
O(4)	24(1)	23(1)	25(1)	-2(1)	3(1)	-2(1)
C(4)	24(1)	17(1)	20(1)	2(1)	0(1)	1(1)
O(5)	44(1)	36(1)	20(1)	6(1)	-2(1)	10(1)
C(5)	34(1)	27(1)	21(1)	-4(1)	2(1)	6(1)
C(6)	24(1)	20(1)	19(1)	1(1)	-2(1)	2(1)
C(7)	25(1)	28(1)	30(1)	-2(1)	7(1)	-2(1)
C(8)	44(2)	38(1)	27(1)	3(1)	4(1)	-9(1)
C(9)	22(1)	19(1)	15(1)	-2(1)	-1(1)	-1(1)

C(10)	26(1)	21(1)	23(1)	-3(1)	-2(1)	4(1)
C(11)	22(1)	33(1)	28(1)	-6(1)	1(1)	0(1)
C(12)	31(1)	26(1)	24(1)	-1(1)	4(1)	-6(1)
C(13)	33(1)	21(1)	24(1)	1(1)	3(1)	2(1)
C(14)	24(1)	21(1)	23(1)	1(1)	0(1)	3(1)
C(15)	19(1)	21(1)	18(1)	1(1)	0(1)	2(1)
C(16)	25(1)	20(1)	22(1)	-1(1)	-1(1)	1(1)
C(17)	26(1)	27(1)	21(1)	-2(1)	-3(1)	4(1)
C(18)	24(1)	31(1)	18(1)	4(1)	0(1)	7(1)
C(19)	30(1)	21(1)	24(1)	4(1)	2(1)	6(1)
C(20)	25(1)	20(1)	22(1)	-1(1)	0(1)	3(1)
C(21)	42(1)	38(1)	25(1)	13(1)	5(1)	10(1)
O(1)	27(1)	23(1)	18(1)	-1(1)	-2(1)	5(1)
N(1)	30(1)	17(1)	17(1)	-1(1)	0(1)	5(1)
C(1)	24(1)	22(1)	19(1)	-2(1)	0(1)	4(1)
O(2)	27(1)	28(1)	26(1)	-1(1)	-3(1)	6(1)
C(2)	27(1)	17(1)	19(1)	-3(1)	0(1)	5(1)

Supplementary Table S27: Hydrogen coordinates (x 10^4) and isotropic displacement parameters (A^2 x 10^3) for t_a.

	x	y	z	U(eq)
H(5A)	4514	7140	7358	41
H(5B)	6626	7265	7402	41
H(5C)	5700	6459	7886	41
H(7A)	11499	5139	3861	33
H(7B)	11825	6188	3839	33
H(8A)	11153	5751	2492	54
H(8B)	9478	6301	2827	54
H(8C)	9405	5241	2800	54
H(10)	3556	5144	5217	28
H(11)	1751	3911	5465	33
H(12)	3020	2545	5800	32
H(13)	6112	2416	5897	31
H(14)	7924	3646	5672	27
H(16)	5461	5300	3714	27
H(17)	5106	5847	2363	30
H(19)	5856	8352	3219	30
H(20)	6272	7795	4573	27

H(21A)	5118	8431	1064	53
H(21B)	6544	8542	1800	53
H(21C)	4469	8658	1994	53
H(1)	4853	5828	6578	26
H(2A)	4973	7156	5736	25
H(2B)	7099	7148	5823	25

Supplementary Table S28: Torsion angles [deg] for t_a.

N(1)-C(3)-C(4)-O(2)	-140.2(2)
C(9)-C(3)-C(4)-O(2)	97.3(2)
C(6)-C(3)-C(4)-O(2)	-18.9(3)
N(1)-C(3)-C(4)-O(1)	43.4(3)
C(9)-C(3)-C(4)-O(1)	-79.1(2)
C(6)-C(3)-C(4)-O(1)	164.66(19)
C(7)-O(4)-C(6)-O(3)	-0.1(3)
C(7)-O(4)-C(6)-C(3)	177.66(17)
N(1)-C(3)-C(6)-O(3)	-132.3(2)
C(9)-C(3)-C(6)-O(3)	-4.7(3)
C(4)-C(3)-C(6)-O(3)	107.5(2)
N(1)-C(3)-C(6)-O(4)	49.9(2)
C(9)-C(3)-C(6)-O(4)	177.45(17)
C(4)-C(3)-C(6)-O(4)	-70.3(2)
C(6)-O(4)-C(7)-C(8)	78.4(3)
N(1)-C(3)-C(9)-C(10)	2.7(3)
C(4)-C(3)-C(9)-C(10)	123.8(2)
C(6)-C(3)-C(9)-C(10)	-124.6(2)
N(1)-C(3)-C(9)-C(14)	-174.69(19)
C(4)-C(3)-C(9)-C(14)	-53.6(2)
C(6)-C(3)-C(9)-C(14)	58.0(3)
C(14)-C(9)-C(10)-C(11)	-1.4(3)
C(3)-C(9)-C(10)-C(11)	-178.79(19)
C(9)-C(10)-C(11)-C(12)	0.2(4)
C(10)-C(11)-C(12)-C(13)	0.4(4)
C(11)-C(12)-C(13)-C(14)	0.1(4)
C(12)-C(13)-C(14)-C(9)	-1.3(4)
C(10)-C(9)-C(14)-C(13)	1.9(3)
C(3)-C(9)-C(14)-C(13)	179.4(2)
C(20)-C(15)-C(16)-C(17)	0.9(3)

N(1)-C(15)-C(16)-C(17)	-179.9(2)
C(15)-C(16)-C(17)-C(18)	-0.7(4)
C(21)-O(5)-C(18)-C(19)	1.3(3)
C(21)-O(5)-C(18)-C(17)	-179.4(2)
C(16)-C(17)-C(18)-O(5)	-179.7(2)
C(16)-C(17)-C(18)-C(19)	-0.3(4)
O(5)-C(18)-C(19)-C(20)	-179.7(2)
C(17)-C(18)-C(19)-C(20)	1.0(3)
C(16)-C(15)-C(20)-C(19)	-0.2(3)
N(1)-C(15)-C(20)-C(19)	-179.3(2)
C(18)-C(19)-C(20)-C(15)	-0.8(3)
O(2)-C(4)-O(1)-C(1)	169.8(2)
C(3)-C(4)-O(1)-C(1)	-13.7(3)
C(20)-C(15)-N(1)-C(2)	15.9(3)
C(16)-C(15)-N(1)-C(2)	-163.2(2)
C(20)-C(15)-N(1)-C(3)	-140.0(2)
C(16)-C(15)-N(1)-C(3)	40.9(3)
C(9)-C(3)-N(1)-C(15)	-101.3(2)
C(4)-C(3)-N(1)-C(15)	141.2(2)
C(6)-C(3)-N(1)-C(15)	25.0(3)
C(9)-C(3)-N(1)-C(2)	102.4(2)
C(4)-C(3)-N(1)-C(2)	-15.2(3)
C(6)-C(3)-N(1)-C(2)	-131.3(2)
C(4)-O(1)-C(1)-C(5)	-161.94(19)
C(4)-O(1)-C(1)-C(2)	-39.8(2)
C(15)-N(1)-C(2)-C(1)	166.80(18)
C(3)-N(1)-C(2)-C(1)	-36.2(3)
O(1)-C(1)-C(2)-N(1)	65.4(2)
C(5)-C(1)-C(2)-N(1)	-177.66(19)

Symmetry transformations used to generate equivalent atoms:

Supplementary Table S29: Hydrogen bonds for t_a [A and deg.].

D-H...A	d(D-H)	d(H...A)	d(D...A)	<(DHA)

13. Crystallographic data for 3ao (CCDC 2246925)

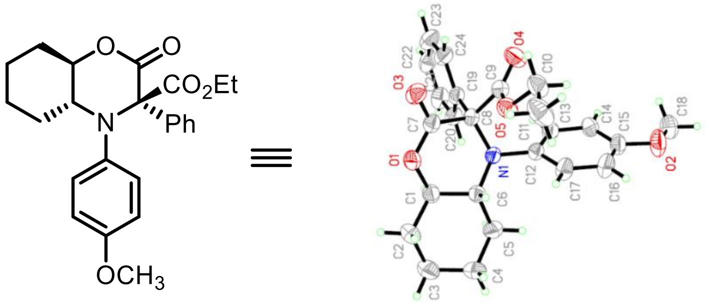

Bond precision:	C-C =0.0034 A		Wavelength=1.54178
Cell:	a=10.667(2)	b=14.276(3)	c=14.284(3)
	alpha=90	beta=90	gamma=90
Temperature:	293 K		
	Calculated		Reported
Volume	2175.2(8)		2175.2(8)
Space group	P 21 21 21		P 21 21 21
Hall group	P 2ac 2ab		P 2ac 2ab
Moiety formula	C24 H27 N O5		C24 H27 N O5
Sum formula	C24 H27 N O5		C24 H27 N O5
Mr	409.47		409.47
Dx,g cm-3	1.250		1.250
Z	4		4
Mu (mm-1)	0.711		0.711
F000	872.0		872.0
F000’	874.73		874.73
h,k,lmax	12,17,17		12,17,17
Nref	4000[2279]		3997
Tmin,Tmax	0.867,0.892		0.632,0.753
Tmin’	0.867		

Correction method= # Reported T Limits: Tmin=0.632 Tmax=0.753

AbsCorr = MULTI-SCAN

Data completeness= 1.75/1.00		Theta(max)= 68.348
R(reflections)= 0.0340(3554)		wR2(reflections)= 0.0925(3997)
S = 1.044	Npar= 247	

Supplementary Table S30: Crystal data and structure refinement for t_a.

Identification code	t_a	
Empirical formula	C24 H27 N O5	
Formula weight	409.46	
Temperature	293(2) K	
Wavelength	1.54178 Å	
Crystal system	Orthorhombic	
Space group	$P2_12_12_1$	
Unit cell dimensions	a = 10.667(2) Å	a= 90°.
	b = 14.276(3) Å	b= 90°.
	c = 14.284(3) Å	γ = 90°.
Volume	2175.2(8) Å^3	
Z	4	
Density (calculated)	1.250 Mg/m^3	
Absorption coefficient	0.711 mm^{-1}	
F(000)	872	
Crystal size	0.200 x 0.180 x 0.160 mm^3	
Theta range for data collection	5.176 to 68.348°.	
Index ranges	-12<=h<=12, -17<=k<=17, -17<=l<=17	
Reflections collected	28280	
Independent reflections	3997 [R(int) = 0.0503]	
Completeness to theta = 67.679°	99.8 %	
Absorption correction	Semi-empirical from equivalents	
Max. and min. transmission	0.7531 and 0.6324	
Refinement method	Full-matrix least-squares on F^2	
Data / restraints / parameters	3997 / 0 / 274	
Goodness-of-fit on F^2	1.044	
Final R indices [I>2sigma(I)]	R1 = 0.0340, wR2 = 0.0881	
R indices (all data)	R1 = 0.0399, wR2 = 0.0925	
Absolute structure parameter	-0.1(2)	
Extinction coefficient	n/a	
Largest diff. peak and hole	0.094 and -0.150 e.Å^{-3}	

Supplementary Table S31: Atomic coordinates (x 10^4) and equivalent isotropic displacement parameters (A^2 x 10^3) for t_a. U(eq) is defined as one third of the trace of the orthogonalized Uij tensor.

	x	y	z	U(eq)
O(1)	-659(2)	-1268(1)	-5278(1)	54(1)
O(2)	1869(2)	-3039(2)	-30(1)	84(1)
O(3)	-1893(2)	-2492(1)	-5237(1)	56(1)
O(4)	-1765(2)	-3472(1)	-3002(1)	65(1)
O(5)	-107(2)	-3391(1)	-3972(1)	53(1)
N(1)	-82(2)	-1496(1)	-3330(1)	41(1)
C(1)	110(2)	-603(2)	-4758(2)	46(1)
C(2)	962(3)	-145(2)	-5467(2)	60(1)
C(3)	1838(3)	552(2)	-4999(2)	70(1)
C(4)	2553(3)	81(2)	-4210(2)	71(1)
C(5)	1658(2)	-371(2)	-3512(2)	57(1)
C(6)	820(2)	-1097(2)	-3993(2)	42(1)
C(7)	-1254(2)	-1939(2)	-4812(2)	45(1)
C(8)	-1186(2)	-1979(2)	-3734(1)	41(1)
C(9)	-1093(2)	-3039(2)	-3516(2)	46(1)
C(10)	171(3)	-4377(2)	-3816(2)	61(1)
C(11)	1242(3)	-4625(2)	-4418(2)	82(1)
C(12)	412(2)	-1915(2)	-2496(1)	42(1)
C(13)	-253(2)	-1851(2)	-1672(2)	50(1)
C(14)	178(2)	-2228(2)	-838(2)	56(1)
C(15)	1334(3)	-2666(2)	-818(2)	57(1)
C(16)	2015(3)	-2742(2)	-1637(2)	65(1)
C(17)	1559(2)	-2382(2)	-2468(2)	56(1)
C(18)	1274(3)	-2878(3)	838(2)	85(1)
C(19)	-2425(2)	-1549(2)	-3396(1)	43(1)
C(20)	-2452(2)	-651(2)	-3012(2)	51(1)
C(21)	-3575(2)	-244(2)	-2749(2)	61(1)
C(22)	-4690(2)	-713(2)	-2866(2)	66(1)
C(23)	-4678(2)	-1593(2)	-3253(2)	66(1)
C(24)	-3570(2)	-2011(2)	-3518(2)	55(1)

Supplementary Table S32: Bond lengths [A] and angles [deg] for t_a.

O(1)-C(7)	1.328(3)

O(1)-C(1)	1.458(3)
O(2)-C(15)	1.370(3)
O(2)-C(18)	1.411(4)
O(3)-C(7)	1.207(3)
O(4)-C(9)	1.198(3)
O(5)-C(9)	1.335(3)
O(5)-C(10)	1.455(3)
N(1)-C(12)	1.434(3)
N(1)-C(6)	1.465(3)
N(1)-C(8)	1.482(3)
C(1)-C(6)	1.506(3)
C(1)-C(2)	1.509(3)
C(1)-H(1)	0.9800
C(2)-C(3)	1.520(4)
C(2)-H(2A)	0.9700
C(2)-H(2B)	0.9700
C(3)-C(4)	1.518(4)
C(3)-H(3A)	0.9700
C(3)-H(3B)	0.9700
C(4)-C(5)	1.523(4)
C(4)-H(4A)	0.9700
C(4)-H(4B)	0.9700
C(5)-C(6)	1.531(3)
C(5)-H(5A)	0.9700
C(5)-H(5B)	0.9700
C(6)-H(6)	0.9800
C(7)-C(8)	1.542(3)
C(8)-C(19)	1.536(3)
C(8)-C(9)	1.548(3)
C(10)-C(11)	1.474(4)
C(10)-H(10A)	0.9700
C(10)-H(10B)	0.9700
C(11)-H(11A)	0.9600
C(11)-H(11B)	0.9600
C(11)-H(11C)	0.9600
C(12)-C(13)	1.378(3)
C(12)-C(17)	1.394(3)
C(13)-C(14)	1.386(3)

C(13)-H(13)	0.9300
C(14)-C(15)	1.382(4)
C(14)-H(14)	0.9300
C(15)-C(16)	1.381(4)
C(16)-C(17)	1.383(3)
C(16)-H(16)	0.9300
C(17)-H(17)	0.9300
C(18)-H(18A)	0.9600
C(18)-H(18B)	0.9600
C(18)-H(18C)	0.9600
C(19)-C(20)	1.394(3)
C(19)-C(24)	1.399(3)
C(20)-C(21)	1.384(3)
C(20)-H(20)	0.9300
C(21)-C(22)	1.375(4)
C(21)-H(21)	0.9300
C(22)-C(23)	1.373(4)
C(22)-H(22)	0.9300
C(23)-C(24)	1.378(4)
C(23)-H(23)	0.9300
C(24)-H(24)	0.9300
C(7)-O(1)-C(1)	118.91(16)
C(15)-O(2)-C(18)	118.1(2)
C(9)-O(5)-C(10)	116.70(18)
C(12)-N(1)-C(6)	117.26(16)
C(12)-N(1)-C(8)	114.88(16)
C(6)-N(1)-C(8)	116.86(16)
O(1)-C(1)-C(6)	110.36(18)
O(1)-C(1)-C(2)	106.23(18)
C(6)-C(1)-C(2)	112.75(19)
O(1)-C(1)-H(1)	109.1
C(6)-C(1)-H(1)	109.1
C(2)-C(1)-H(1)	109.1
C(1)-C(2)-C(3)	111.0(2)
C(1)-C(2)-H(2A)	109.4
C(3)-C(2)-H(2A)	109.4
C(1)-C(2)-H(2B)	109.4

C(3)-C(2)-H(2B)	109.4
H(2A)-C(2)-H(2B)	108.0
C(4)-C(3)-C(2)	110.2(2)
C(4)-C(3)-H(3A)	109.6
C(2)-C(3)-H(3A)	109.6
C(4)-C(3)-H(3B)	109.6
C(2)-C(3)-H(3B)	109.6
H(3A)-C(3)-H(3B)	108.1
C(3)-C(4)-C(5)	111.0(2)
C(3)-C(4)-H(4A)	109.4
C(5)-C(4)-H(4A)	109.4
C(3)-C(4)-H(4B)	109.4
C(5)-C(4)-H(4B)	109.4
H(4A)-C(4)-H(4B)	108.0
C(4)-C(5)-C(6)	111.0(2)
C(4)-C(5)-H(5A)	109.4
C(6)-C(5)-H(5A)	109.4
C(4)-C(5)-H(5B)	109.4
C(6)-C(5)-H(5B)	109.4
H(5A)-C(5)-H(5B)	108.0
N(1)-C(6)-C(1)	108.69(17)
N(1)-C(6)-C(5)	110.92(18)
C(1)-C(6)-C(5)	107.63(19)
N(1)-C(6)-H(6)	109.9
C(1)-C(6)-H(6)	109.9
C(5)-C(6)-H(6)	109.9
O(3)-C(7)-O(1)	119.33(19)
O(3)-C(7)-C(8)	120.3(2)
O(1)-C(7)-C(8)	120.32(19)
N(1)-C(8)-C(19)	112.02(16)
N(1)-C(8)-C(7)	114.12(17)
C(19)-C(8)-C(7)	105.00(17)
N(1)-C(8)-C(9)	109.05(17)
C(19)-C(8)-C(9)	112.48(18)
C(7)-C(8)-C(9)	103.91(17)
O(4)-C(9)-O(5)	125.2(2)
O(4)-C(9)-C(8)	126.1(2)
O(5)-C(9)-C(8)	108.65(18)

O(5)-C(10)-C(11)	107.5(2)
O(5)-C(10)-H(10A)	110.2
C(11)-C(10)-H(10A)	110.2
O(5)-C(10)-H(10B)	110.2
C(11)-C(10)-H(10B)	110.2
H(10A)-C(10)-H(10B)	108.5
C(10)-C(11)-H(11A)	109.5
C(10)-C(11)-H(11B)	109.5
H(11A)-C(11)-H(11B)	109.5
C(10)-C(11)-H(11C)	109.5
H(11A)-C(11)-H(11C)	109.5
H(11B)-C(11)-H(11C)	109.5
C(13)-C(12)-C(17)	117.4(2)
C(13)-C(12)-N(1)	119.55(19)
C(17)-C(12)-N(1)	123.1(2)
C(12)-C(13)-C(14)	122.5(2)
C(12)-C(13)-H(13)	118.7
C(14)-C(13)-H(13)	118.7
C(15)-C(14)-C(13)	119.3(2)
C(15)-C(14)-H(14)	120.4
C(13)-C(14)-H(14)	120.4
O(2)-C(15)-C(14)	124.4(2)
O(2)-C(15)-C(16)	116.5(2)
C(14)-C(15)-C(16)	119.2(2)
C(15)-C(16)-C(17)	120.9(2)
C(15)-C(16)-H(16)	119.6
C(17)-C(16)-H(16)	119.6
C(16)-C(17)-C(12)	120.7(2)
C(16)-C(17)-H(17)	119.6
C(12)-C(17)-H(17)	119.6
O(2)-C(18)-H(18A)	109.5
O(2)-C(18)-H(18B)	109.5
H(18A)-C(18)-H(18B)	109.5
O(2)-C(18)-H(18C)	109.5
H(18A)-C(18)-H(18C)	109.5
H(18B)-C(18)-H(18C)	109.5
C(20)-C(19)-C(24)	117.7(2)
C(20)-C(19)-C(8)	120.59(19)

C(24)-C(19)-C(8)	121.5(2)
C(21)-C(20)-C(19)	120.7(2)
C(21)-C(20)-H(20)	119.7
C(19)-C(20)-H(20)	119.7
C(22)-C(21)-C(20)	120.8(3)
C(22)-C(21)-H(21)	119.6
C(20)-C(21)-H(21)	119.6
C(21)-C(22)-C(23)	119.1(2)
C(21)-C(22)-H(22)	120.4
C(23)-C(22)-H(22)	120.4
C(22)-C(23)-C(24)	121.0(2)
C(22)-C(23)-H(23)	119.5
C(24)-C(23)-H(23)	119.5
C(23)-C(24)-C(19)	120.7(2)
C(23)-C(24)-H(24)	119.7
C(19)-C(24)-H(24)	119.7

Symmetry transformations used to generate equivalent atoms:

Supplementary Table S33: Anisotropic displacement parameters (A^2 x 10^3) for t_a. The anisotropic displacement factor exponent takes the form: -2 pi^2 [h^2 a*^2 U11 + ... + 2 h k a* b* U12]

	U^{11}	U^{22}	U^{33}	U^{23}	U^{13}	U^{12}
O (1)	62(1)	64(1)	37(1)	6(1)	-7(1)	-11(1)
O(2)	92(2)	105(2)	55(1)	24(1)	-19(1)	19(1)
O(3)	57(1)	66(1)	46(1)	-10(1)	-7(1)	-8(1)
O(4)	68(1)	60(1)	67(1)	17(1)	12(1)	-4(1)
O(5)	56(1)	43(1)	58(1)	4(1)	5(1)	4(1)
N(1)	38(1)	49(1)	36(1)	2(1)	-4(1)	-1(1)
C(1)	49(1)	45(1)	42(1)	2(1)	-2(1)	0(1)
C(2)	68(2)	55(1)	56(1)	13(1)	6(1)	-5(1)
C(3)	74(2)	56(1)	81(2)	18(1)	4(2)	-11(1)
C(4)	57(2)	67(2)	90(2)	14(2)	-1(2)	-20(1)
C(5)	53(1)	61(1)	57(1)	0(1)	-5(1)	-13(1)
C(6)	41(1)	44(1)	41(1)	2(1)	2(1)	0(1)
C(7)	43(1)	52(1)	40(1)	-2(1)	-3(1)	2(1)
C(8)	42(1)	45(1)	35(1)	2(1)	-4(1)	-2(1)
C(9)	47(1)	51(1)	42(1)	4(1)	-4(1)	-3(1)
C(10)	69(2)	46(1)	68(2)	12(1)	3(1)	9(1)
C(11)	96(2)	61(2)	90(2)	20(2)	28(2)	22(2)

C(12)	43(1)	44(1)	39(1)	1(1)	-6(1)	-2(1)
C(13)	46(1)	62(1)	43(1)	4(1)	-2(1)	3(1)
C(14)	61(1)	67(2)	39(1)	8(1)	0(1)	-4(1)
C(15)	64(1)	58(1)	48(1)	8(1)	-16(1)	1(1)
C(16)	58(1)	79(2)	57(1)	3(1)	-10(1)	20(1)
C(17)	49(1)	72(2)	45(1)	-1(1)	-2(1)	15(1)
C(18)	98(2)	108(3)	49(2)	24(2)	-18(2)	-9(2)
C(19)	41(1)	53(1)	36(1)	2(1)	-3(1)	2(1)
C(20)	47(1)	60(1)	45(1)	-2(1)	-3(1)	3(1)
C(21)	59(2)	71(2)	52(1)	-6(1)	-2(1)	18(1)
C(22)	47(1)	102(2)	48(1)	-4(1)	-2(1)	18(1)
C(23)	43(1)	104(2)	50(1)	-6(1)	-1(1)	-4(1)
C(24)	46(1)	72(2)	46(1)	-6(1)	-3(1)	-6(1)

Supplementary Table S34: Hydrogen coordinates (x 10^4) and isotropic displacement parameters (A^2 x 10^3) for t_a.

	x	y	z	U(eq)
H(1)	-434	-125	-4479	55
H(2A)	459	177	-5933	71
H(2B)	1451	-621	-5784	71
H(3A)	2425	796	-5457	85
H(3B)	1357	1073	-4752	85
H(4A)	3066	542	-3890	86
H(4B)	3105	-393	-4467	86
H(5A)	1139	109	-3227	69
H(5B)	2137	-672	-3019	69
H(6)	1337	-1596	-4261	50
H(10A)	-552	-4759	-3973	73
H(10B)	379	-4483	-3163	73
H(11A)	1433	-5278	-4344	124
H(11B)	1958	-4258	-4243	124
H(11C)	1033	-4502	-5060	124
H(13)	-1021	-1543	-1675	61
H(14)	-304	-2186	-297	67
H(16)	2791	-3038	-1628	78
H(17)	2023	-2452	-3015	67
H(18A)	1711	-3211	1322	128
H(18B)	423	-3093	807	128

H(18C)	1284	-2219	974	128
H(20)	-1707	-322	-2933	61
H(21)	-3576	354	-2490	73
H(22)	-5442	-437	-2686	79
H(23)	-5430	-1912	-3338	79
H(24)	-3582	-2608	-3780	66

Supplementary Table S35: Torsion angles [deg] for t_a.

C(7)-O(1)-C(1)-C(6)	39.9(3)
C(7)-O(1)-C(1)-C(2)	162.5(2)
O(1)-C(1)-C(2)-C(3)	-178.5(2)
C(6)-C(1)-C(2)-C(3)	-57.5(3)
C(1)-C(2)-C(3)-C(4)	54.2(3)
C(2)-C(3)-C(4)-C(5)	-55.3(3)
C(3)-C(4)-C(5)-C(6)	58.5(3)
C(12)-N(1)-C(6)-C(1)	-173.84(18)
C(8)-N(1)-C(6)-C(1)	43.8(2)
C(12)-N(1)-C(6)-C(5)	-55.7(3)
C(8)-N(1)-C(6)-C(5)	161.98(19)
O(1)-C(1)-C(6)-N(1)	-62.8(2)
C(2)-C(1)-C(6)-N(1)	178.54(19)
O(1)-C(1)-C(6)-C(5)	176.96(18)
C(2)-C(1)-C(6)-C(5)	58.3(2)
C(4)-C(5)-C(6)-N(1)	-177.1(2)
C(4)-C(5)-C(6)-C(1)	-58.3(3)
C(1)-O(1)-C(7)-O(3)	179.9(2)
C(1)-O(1)-C(7)-C(8)	3.1(3)
C(12)-N(1)-C(8)-C(19)	94.3(2)
C(6)-N(1)-C(8)-C(19)	-122.46(19)
C(12)-N(1)-C(8)-C(7)	-146.51(19)
C(6)-N(1)-C(8)-C(7)	-3.3(3)
C(12)-N(1)-C(8)-C(9)	-30.8(2)
C(6)-N(1)-C(8)-C(9)	112.4(2)
O(3)-C(7)-C(8)-N(1)	160.6(2)
O(1)-C(7)-C(8)-N(1)	-22.7(3)
O(3)-C(7)-C(8)-C(19)	-76.4(3)
O(1)-C(7)-C(8)-C(19)	100.4(2)

O(3)-C(7)-C(8)-C(9)	41.9(3)
O(1)-C(7)-C(8)-C(9)	-141.3(2)
C(10)-O(5)-C(9)-O(4)	-0.8(3)
C(10)-O(5)-C(9)-C(8)	177.0(2)
N(1)-C(8)-C(9)-O(4)	111.9(2)
C(19)-C(8)-C(9)-O(4)	-13.0(3)
C(7)-C(8)-C(9)-O(4)	-126.0(2)
N(1)-C(8)-C(9)-O(5)	-66.0(2)
C(19)-C(8)-C(9)-O(5)	169.14(17)
C(7)-C(8)-C(9)-O(5)	56.1(2)
C(9)-O(5)-C(10)-C(11)	177.2(2)
C(6)-N(1)-C(12)-C(13)	145.7(2)
C(8)-N(1)-C(12)-C(13)	-71.3(3)
C(6)-N(1)-C(12)-C(17)	-33.3(3)
C(8)-N(1)-C(12)-C(17)	109.7(2)
C(17)-C(12)-C(13)-C(14)	0.1(4)
N(1)-C(12)-C(13)-C(14)	-179.0(2)
C(12)-C(13)-C(14)-C(15)	1.7(4)
C(18)-O(2)-C(15)-C(14)	-7.3(4)
C(18)-O(2)-C(15)-C(16)	172.6(3)
C(13)-C(14)-C(15)-O(2)	178.0(3)
C(13)-C(14)-C(15)-C(16)	-1.8(4)
O(2)-C(15)-C(16)-C(17)	-179.6(3)
C(14)-C(15)-C(16)-C(17)	0.3(4)
C(15)-C(16)-C(17)-C(12)	1.5(4)
C(13)-C(12)-C(17)-C(16)	-1.6(4)
N(1)-C(12)-C(17)-C(16)	177.4(2)
N(1)-C(8)-C(19)-C(20)	18.7(3)
C(7)-C(8)-C(19)-C(20)	-105.7(2)
C(9)-C(8)-C(19)-C(20)	142.0(2)
N(1)-C(8)-C(19)-C(24)	-165.7(2)
C(7)-C(8)-C(19)-C(24)	69.9(3)
C(9)-C(8)-C(19)-C(24)	-42.5(3)
C(24)-C(19)-C(20)-C(21)	1.1(3)
C(8)-C(19)-C(20)-C(21)	176.8(2)
C(19)-C(20)-C(21)-C(22)	-0.5(4)
C(20)-C(21)-C(22)-C(23)	-0.3(4)
C(21)-C(22)-C(23)-C(24)	0.5(4)

C(22)-C(23)-C(24)-C(19)	0.1(4)
C(20)-C(19)-C(24)-C(23)	-0.9(3)
C(8)-C(19)-C(24)-C(23)	-176.6(2)

Symmetry transformations used to generate equivalent atoms:

Supplementary Table S36: Hydrogen bonds for t_a [A and deg.].

D-H...A	d(D-H)	d(H...A)	d(D...A)	<(DHA)

14. Crystallographic data for 9 (CCDC 2266070)

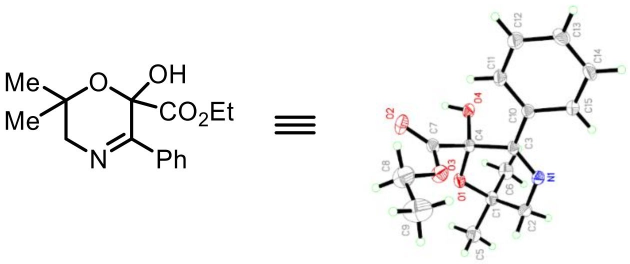

Bond precision:	C-C =0.0019 A	Wavelength=1.54178	
Cell:	a=6.0010(2)	b=29.9965(10)	c=8.1178(3)
	alpha=90	beta=99.559(2)	gamma=90
Temperature:	223 K		
	Calculated	Reported	
Volume	1440.99(9)	1440.99(9)	
Space group	P 21/n	P 21/n	
Hall group	-P 2yn	-P 2yn	
Moiety formula	C15 H19 N O4	C15 H19 N O4	
Sum formula	C15 H19 N O4	C15 H19 N O4	
Mr	277.31	277.31	
Dx,g cm-3	1.278	1.278	
Z	4	4	
Mu (mm-1)	0.763	0.763	
F000	592.0	592.0	
F000’	593.92	593.92	
h,k,lmax	7,36,9	7,36,9	
Nref	2639	2629	
Tmin,Tmax	0.872,0.899	0.636,0.753	
Tmin’	0.872		

Correction method= # Reported T Limits: Tmin=0.636 Tmax=0.753

AbsCorr = MULTI-SCAN

Data completeness= 0.996 Theta(max)= 68.336

		wR2(reflections)=
R(reflections)= 0.0356(2279)		0.0988(2629)
S = 1.036	Npar= 185	

Supplementary Table S37: Crystal data and structure refinement for t_a.

Identification code	t_a	
Empirical formula	C15H19NO4	
Formula weight	277.31	
Temperature	223(2) K	
Wavelength	1.54178 A	
Crystal system, space group	Monoclinic, P2(1)/n	
Unit cell dimensions	a = 6.0010(2) A	a = 90°
	b = 29.9965(10) A	b = 90°
	c = 8.1178(3) A	c = 90°
Volume	1440.99(9) A^3	
Z, Calculated density	4, 1.278 Mg/m^3	
Absorption coefficient	0.763 mm^-1	
F (000)	592	
Crystal size	0.180 x 0.160 x 0.140 mm	
Theta range for data collection	2.946 to 68.336 deg	
Limiting indices	-7<=h<=7, -36<=k<=36, -9<=l<=9	
Reflections collected / unique	14815 / 2629 [R(int) = 0.0396]	
Completeness to theta = 67.679	99.6 %	
Absorption correction	Semi-empirical from equivalents	
Max. and min. transmission	0.7531 and 0.6359	
Refinement method	Full-matrix least-squares on F^2	
Data / restraints / parameters	2629 / 0 / 185	
Goodness-of-fit on F^2	1.036	
Final R indices [I>2sigma(I)]	R1 = 0.0356, wR2 = 0.0937	
R indices (all data)	R1 = 0.0420, wR2 = 0.0988	
Extinction coefficient	n/a	
Largest diff. peak and hole	0.256 and -0.218 e.A^-3	

Supplementary Table S38: Atomic coordinates (x 10^4) and equivalent isotropic displacement parameters (A^2 x 10^3) for t_a. U(eq) is defined as one third of the trace of the orthogonalized Uij tensor.

	x	y	z	U(eq)
O(3)	2613(2)	3180(1)	3450(1)	34(1)
C(3)	4333(2)	3946(1)	5149(2)	21(1)
O(4)	582(1)	4143(1)	5270(1)	28(1)
C(4)	1862(2)	3922(1)	4260(2)	22(1)
C(5)	2395(3)	4542(1)	436(2)	38(1)

C(6)	2314(3)	4882(1)	3250(2)	37(1)
C(7)	1036(2)	3436(1)	3931(2)	26(1)
C(8)	1985(3)	2716(1)	3131(3)	52(1)
C(9)	3964(4)	2471(1)	2733(3)	64(1)
C(10)	4893(2)	3753(1)	6861(2)	23(1)
C(11)	3356(2)	3498(1)	7576(2)	31(1)
C(12)	3935(2)	3325(1)	9173(2)	36(1)
C(13)	6052(2)	3401(1)	10098(2)	34(1)
C(14)	7585(2)	3657(1)	9413(2)	32(1)
C(15)	7018(2)	3832(1)	7822(2)	28(1)
O(1)	1551(1)	4090(1)	2609(1)	25(1)
N(1)	5890(2)	4130(1)	4499(1)	24(1)
C(1)	2926(2)	4472(1)	2312(2)	26(1)
O(2)	-831(2)	3317(1)	4066(1)	39(1)
C(2)	5373(2)	4336(1)	2846(2)	29(1)

Supplementary Table S39: Bond lengths [A] and angles [deg] for t_a.

O(3)-C(7)	1.3273(16)
O(3)-C(8)	1.4536(18)
C(3)-N(1)	1.2725(16)
C(3)-C(10)	1.4917(17)
C(3)-C(4)	1.5392(16)
O(4)-C(4)	1.3832(15)
C(4)-O(1)	1.4156(15)
C(4)-C(7)	1.5482(17)
C(5)-C(1)	1.5175(19)
C(6)-C(1)	1.5229(19)
C(7)-O(2)	1.1983(16)
C(8)-C(9)	1.477(3)
C(10)-C(11)	1.3970(18)
C(10)-C(15)	1.3998(17)
C(11)-C(12)	1.386(2)
C(12)-C(13)	1.383(2)
C(13)-C(14)	1.384(2)
C(14)-C(15)	1.3836(19)
O(1)-C(1)	1.4535(15)
N(1)-C(2)	1.4620(17)
C(1)-C(2)	1.5168(17)
C(7)-O(3)-C(8)	115.51(11)
N(1)-C(3)-C(10)	119.16(10)
N(1)-C(3)-C(4)	122.89(11)

C(10)-C(3)-C(4)	117.94(10)
O(4)-C(4)-O(1)	112.99(10)
O(4)-C(4)-C(3)	106.58(9)
O(1)-C(4)-C(3)	113.07(10)
O(4)-C(4)-C(7)	111.16(10)
O(1)-C(4)-C(7)	100.68(9)
C(3)-C(4)-C(7)	112.47(10)
O(2)-C(7)-O(3)	125.24(12)
O(2)-C(7)-C(4)	122.86(12)
O(3)-C(7)-C(4)	111.88(10)
O(3)-C(8)-C(9)	108.73(15)
C(11)-C(10)-C(15)	117.66(12)
C(11)-C(10)-C(3)	122.54(11)
C(15)-C(10)-C(3)	119.79(11)
C(12)-C(11)-C(10)	120.93(12)
C(13)-C(12)-C(11)	120.66(13)
C(12)-C(13)-C(14)	119.11(13)
C(13)-C(14)-C(15)	120.55(12)
C(14)-C(15)-C(10)	121.09(12)
C(4)-O(1)-C(1)	116.70(9)
C(3)-N(1)-C(2)	120.30(10)
O(1)-C(1)-C(2)	106.85(10)
O(1)-C(1)-C(5)	104.16(10)
C(2)-C(1)-C(5)	110.85(11)
O(1)-C(1)-C(6)	111.23(11)
C(2)-C(1)-C(6)	112.13(11)
C(5)-C(1)-C(6)	111.25(12)
N(1)-C(2)-C(1)	115.06(10)

Symmetry transformations used to generate equivalent atoms:

Supplementary Table S40: Anisotropic displacement parameters (A^2 x 10^3) for t_a. The anisotropic displacement factor exponent takes the form: -2 pi^2 [h^2 a*^2 U11 + ... + 2 h k a* b* U12]

	U^{11}	U^{22}	U^{33}	U^{23}	U^{13}	U^{12}
O(3)	36(1)	24(1)	44(1)	-3(1)	13(1)	-2(1)
C(3)	17(1)	22(1)	24(1)	-3(1)	1(1)	1(1)
O(4)	16(1)	35(1)	31(1)	-5(1)	3(1)	1(1)
C(4)	18(1)	26(1)	22(1)	0(1)	1(1)	0(1)
C(5)	38(1)	40(1)	31(1)	10(1)	-2(1)	-4(1)
C(6)	38(1)	26(1)	46(1)	-1(1)	2(1)	2(1)
C(7)	24(1)	29(1)	22(1)	2(1)	-1(1)	-2(1)
C(8)	68(1)	25(1)	70(1)	-7(1)	27(1)	-7(1)
C(9)	90(2)	32(1)	78(1)	6(1)	37(1)	13(1)
C(10)	21(1)	25(1)	23(1)	-2(1)	1(1)	2(1)

C(11)	24(1)	38(1)	29(1)	3(1)	-2(1)	-5(1)
C(12)	35(1)	39(1)	32(1)	8(1)	2(1)	-8(1)
C(13)	39(1)	35(1)	24(1)	4(1)	-2(1)	1(1)
C(14)	26(1)	39(1)	26(1)	-2(1)	-5(1)	-1(1)
C(15)	22(1)	33(1)	26(1)	-1(1)	2(1)	-3(1)
O(1)	24(1)	26(1)	24(1)	2(1)	-3(1)	-4(1)
N(1)	19(1)	28(1)	25(1)	1(1)	3(1)	1(1)
C(1)	26(1)	24(1)	28(1)	3(1)	1(1)	-2(1)
O(2)	26(1)	38(1)	52(1)	-4(1)	4(1)	-10(1)
C(2)	24(1)	35(1)	26(1)	4(1)	4(1)	-1(1)

Supplementary Table S41: Hydrogen coordinates (x 10^4) and isotropic displacement parameters (A^2 x 10^3) for t_a.

	x	y	z	U(eq)
H(4)	-774	4084	4965	41
H(5A)	3221	4798	133	56
H(5B)	786	4592	104	56
H(5C)	2838	4279	-128	56
H(6A)	3135	5138	2934	56
H(6B)	2716	4832	4443	56
H(6C)	703	4937	2969	56
H(8A)	730	2695	2193	63
H(8B)	1496	2585	4118	63
H(9A)	3614	2155	2630	96
H(9B)	5241	2516	3619	96
H(9C)	4336	2580	1687	96
H(11)	1910	3443	6966	38
H(12)	2879	3153	9632	43
H(13)	6444	3281	11176	40
H(14)	9025	3713	10035	38
H(15)	8076	4007	7378	33
H(2A)	5781	4126	2021	34
H(2B)	6326	4601	2832	34

Supplementary Table S42: Torsion angles [deg] for t_a.

N(1)-C(3)-C(4)-O(4)	-119.88(12)
C(10)-C(3)-C(4)-O(4)	58.82(13)
N(1)-C(3)-C(4)-O(1)	4.86(16)
C(10)-C(3)-C(4)-O(1)	-176.44(10)
N(1)-C(3)-C(4)-C(7)	118.07(13)
C(10)-C(3)-C(4)-C(7)	-63.23(14)
C(8)-O(3)-C(7)-O(2)	-2.3(2)

C(8)-O(3)-C(7)-C(4)	179.37(12)
O(4)-C(4)-C(7)-O(2)	21.98(17)
O(1)-C(4)-C(7)-O(2)	-97.97(14)
C(3)-C(4)-C(7)-O(2)	141.40(13)
O(4)-C(4)-C(7)-O(3)	-159.63(10)
O(1)-C(4)-C(7)-O(3)	80.43(12)
C(3)-C(4)-C(7)-O(3)	-40.21(14)
C(7)-O(3)-C(8)-C(9)	-175.89(14)
N(1)-C(3)-C(10)-C(11)	-171.77(12)
C(4)-C(3)-C(10)-C(11)	9.48(17)
N(1)-C(3)-C(10)-C(15)	9.24(17)
C(4)-C(3)-C(10)-C(15)	-169.50(11)
C(15)-C(10)-C(11)-C(12)	-0.9(2)
C(3)-C(10)-C(11)-C(12)	-179.86(13)
C(10)-C(11)-C(12)-C(13)	0.1(2)
C(11)-C(12)-C(13)-C(14)	0.5(2)
C(12)-C(13)-C(14)-C(15)	-0.4(2)
C(13)-C(14)-C(15)-C(10)	-0.4(2)
C(11)-C(10)-C(15)-C(14)	0.99(19)
C(3)-C(10)-C(15)-C(14)	-179.97(12)
O(4)-C(4)-O(1)-C(1)	85.20(12)
C(3)-C(4)-O(1)-C(1)	-35.97(13)
C(7)-C(4)-O(1)-C(1)	-156.18(10)
C(10)-C(3)-N(1)-C(2)	-177.91(11)
C(4)-C(3)-N(1)-C(2)	0.77(17)
C(4)-O(1)-C(1)-C(2)	56.94(13)
C(4)-O(1)-C(1)-C(5)	174.32(10)
C(4)-O(1)-C(1)-C(6)	-65.74(13)
C(3)-N(1)-C(2)-C(1)	22.73(17)
O(1)-C(1)-C(2)-N(1)	-49.17(14)
C(5)-C(1)-C(2)-N(1)	-162.05(11)
C(6)-C(1)-C(2)-N(1)	72.95(15)

Symmetry transformations used to generate equivalent atoms:

Supplementary Table S43: Hydrogen bonds for t_a [A and deg.].

D-H...A	d(D-H)	d(H...A)	d(D...A)	<(DHA)

15. HPLC chromatograms

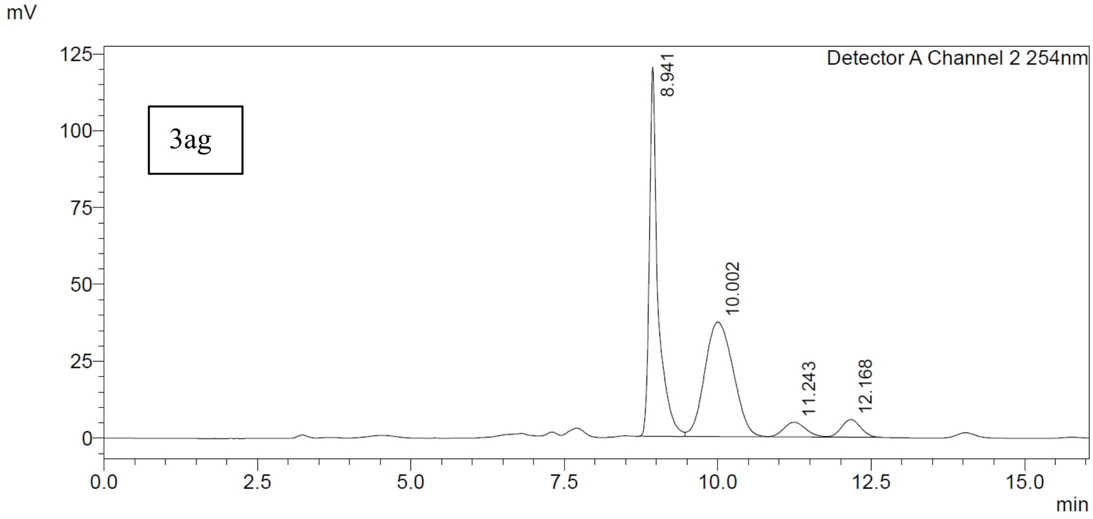

Detector A Channel 2 254nm

Peak#	Ret. Time	Area	Height	Conc.
1	8.941	1170659	120106	44.761
2	10.002	1202435	37264	45.976
3	11.243	119075	4781	4.553
4	12.168	123193	5699	4.710
Total		2615362	167851	

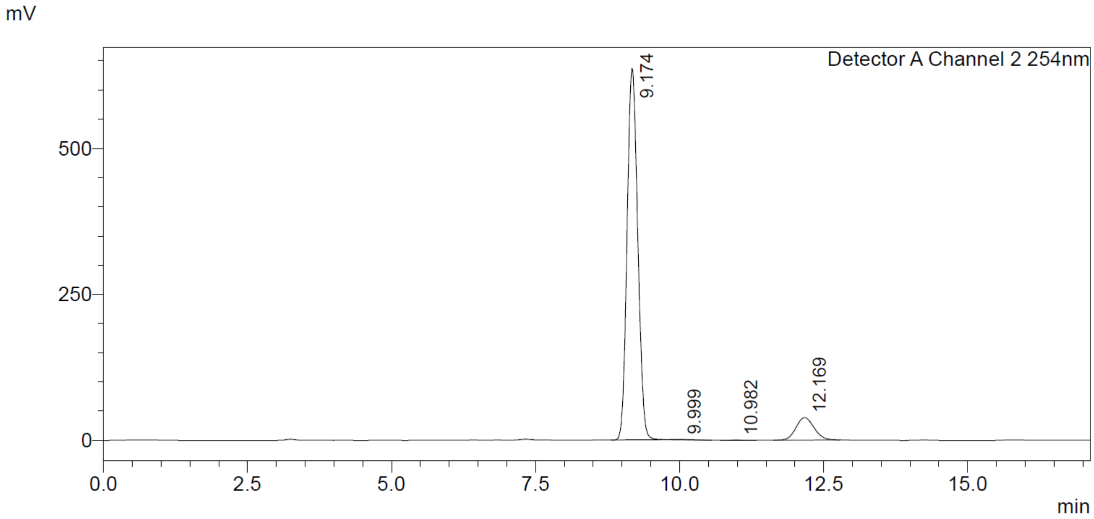

Detector A Channel 2 254nm

Peak#	Ret. Time	Area	Height	Conc.
1	9.174	8170071	636007	90.542
2	9.999	8735	493	0.097
3	10.982	4076	249	0.045
4	12.169	840652	38447	9.316
Total		9023535	675197	

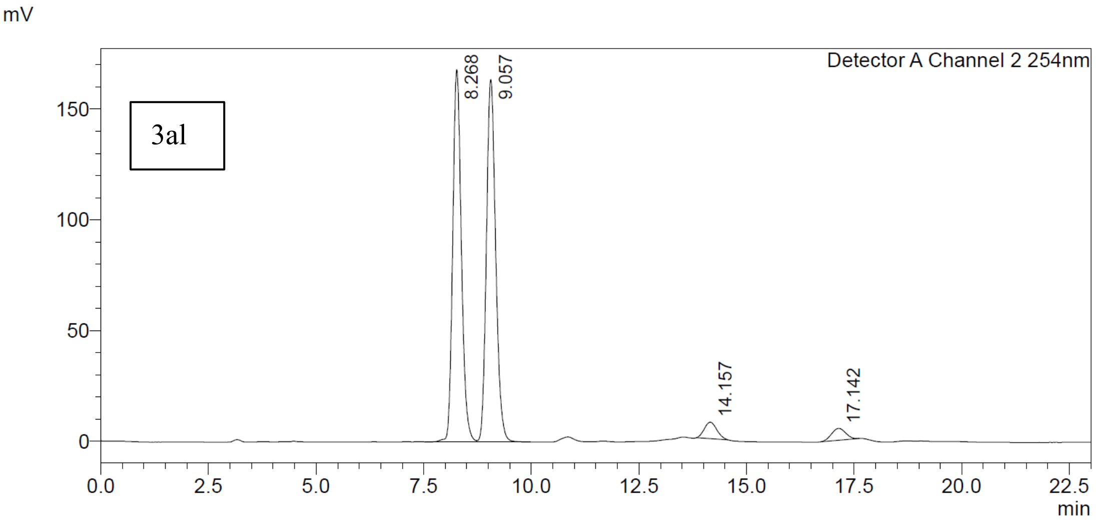

Detector A Channel 2 254nm

Peak#	Ret. Time	Area	Height	Conc.
1	8.268	2350037	168242	46.288
2	9.057	2453417	163912	48.324
3	14.157	150553	7430	2.965
4	17.142	123017	5355	2.423
Total		5077025	344939	

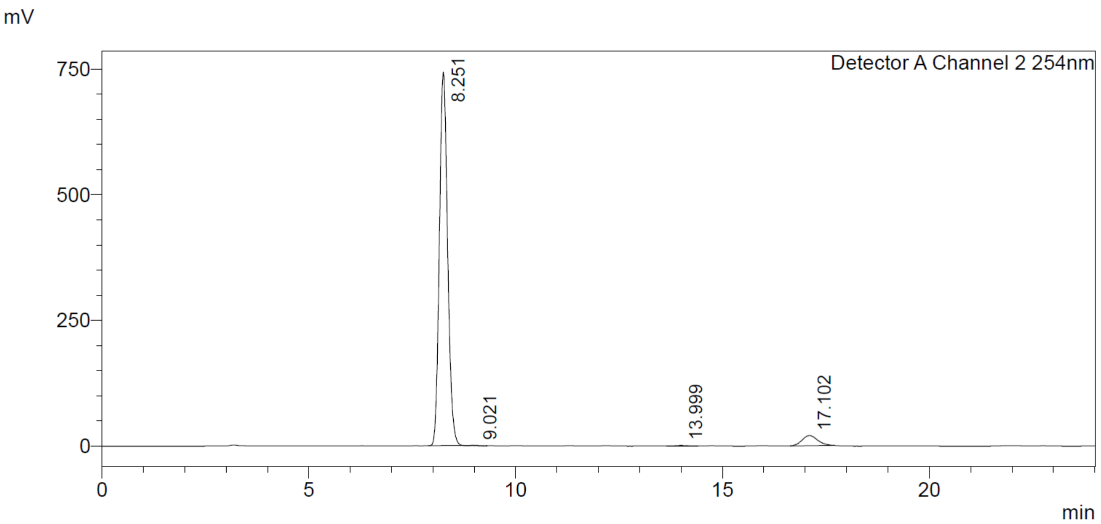

Detector A Channel 2 254nm

Peak#	Ret. Time	Area	Height	Conc.
1	8.251	10272710	742779	94.843
2	9.021	6657	703	0.061
3	13.999	20439	1081	0.189
4	17.102	531510	20235	4.907
Total		10831316	764797	

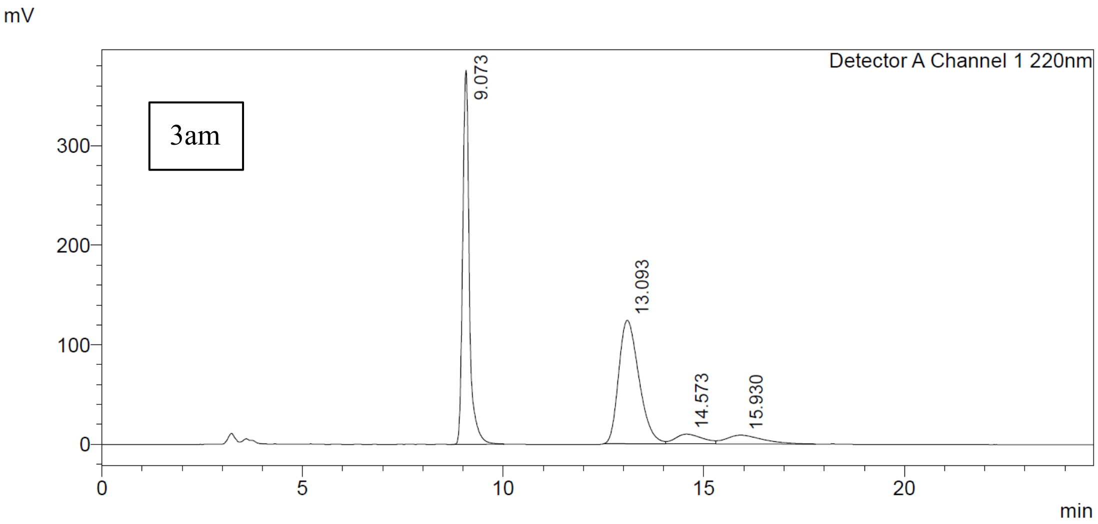

Detector A Channel 1 220nm

Peak#	Ret. Time	Area	Height	Conc.
1	9.073	4324148	375455	44.594
2	13.093	4353010	123886	44.891
3	14.573	459266	9703	4.736
4	15.930	560366	8794	5.779
Total		9696790	517838	

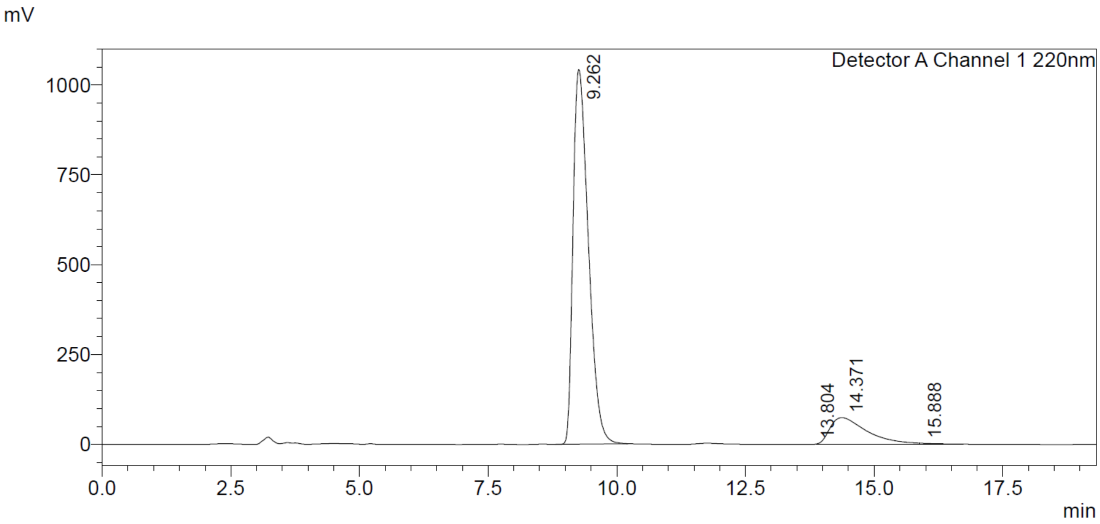

Detector A Channel 1 220nm

Peak#	Ret. Time	Area	Height	Conc.
1	9.262	21577465	1042371	85.430
2	13.804	8022	367	0.032
3	14.371	3627149	73557	14.361
4	15.888	44761	2507	0.177
Total		25257397	1118802	
