# Supplementary material for: Zinc chloride-catalyzed cyclizative 1,2-rearrangement enables facile access to morpholinones bearing aza-quaternary carbons
Source: Commun Chem. 2023 Oct 7;6:216. doi: 10.1038/s42004-023-01016-y (PMC10560277; doi:10.1038/s42004-023-01016-y)
Supplement: Supplementary file 2 — Description of Additional Supplementary Files [file 42004_2023_1016_MOESM2_ESM.pdf]

# Description of Additional Supplementary Files

**File name:** Supplementary Data 1

**Description:** cif. file of **3a**

**File name:** Supplementary Data 2

**Description:** cif. file of **3ag**

**File name:** Supplementary Data 3

**Description:** cif. file of **3am**

**File name:** Supplementary Data 4

**Description:** cif. file of **3an**

**File name:** Supplementary Data 5

**Description:** cif. file of **3ao**

**File name:** Supplementary Data 6

**Description:** cif. file of **9**

**File name:** Supplementary Data 7

**Description:** NMR spectra
