# Supplementary figures and images for: Zinc chloride-catalyzed cyclizative 1,2-rearrangement enables facile access to morpholinones bearing aza-quaternary carbons
Source: Commun Chem. 2023 Oct 7;6:216. doi: 10.1038/s42004-023-01016-y (PMC10560277; doi:10.1038/s42004-023-01016-y)

## Copies of NMR spectra

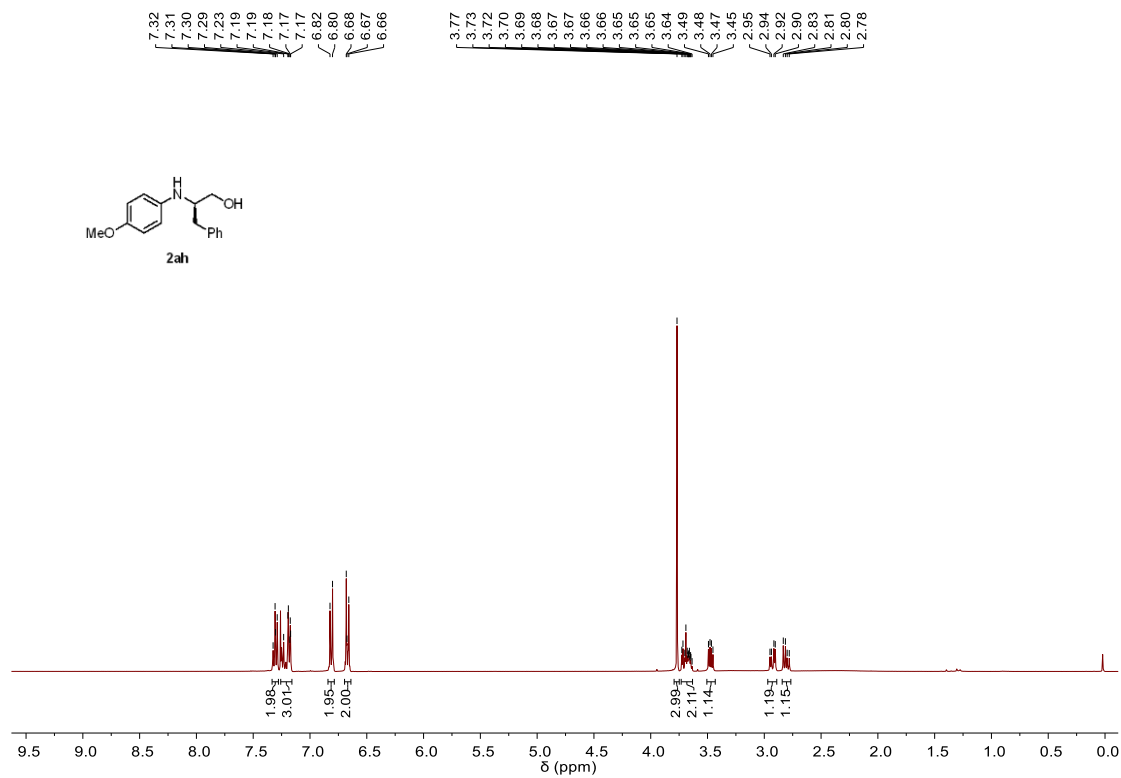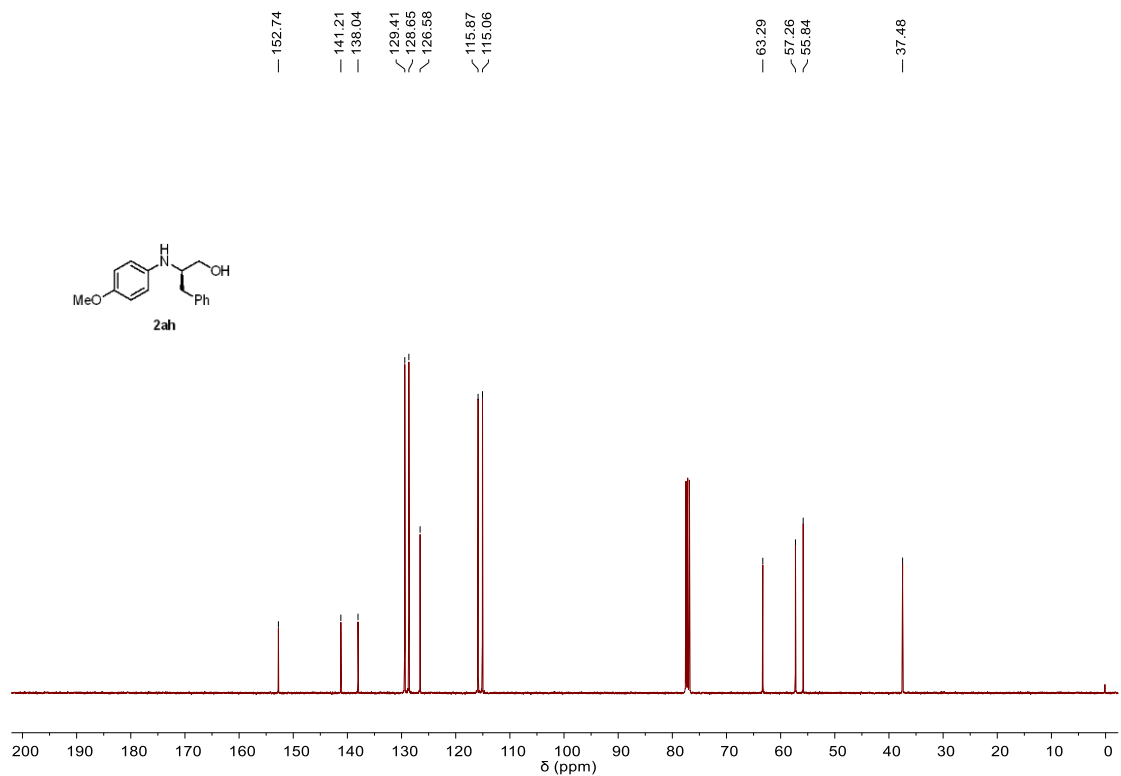

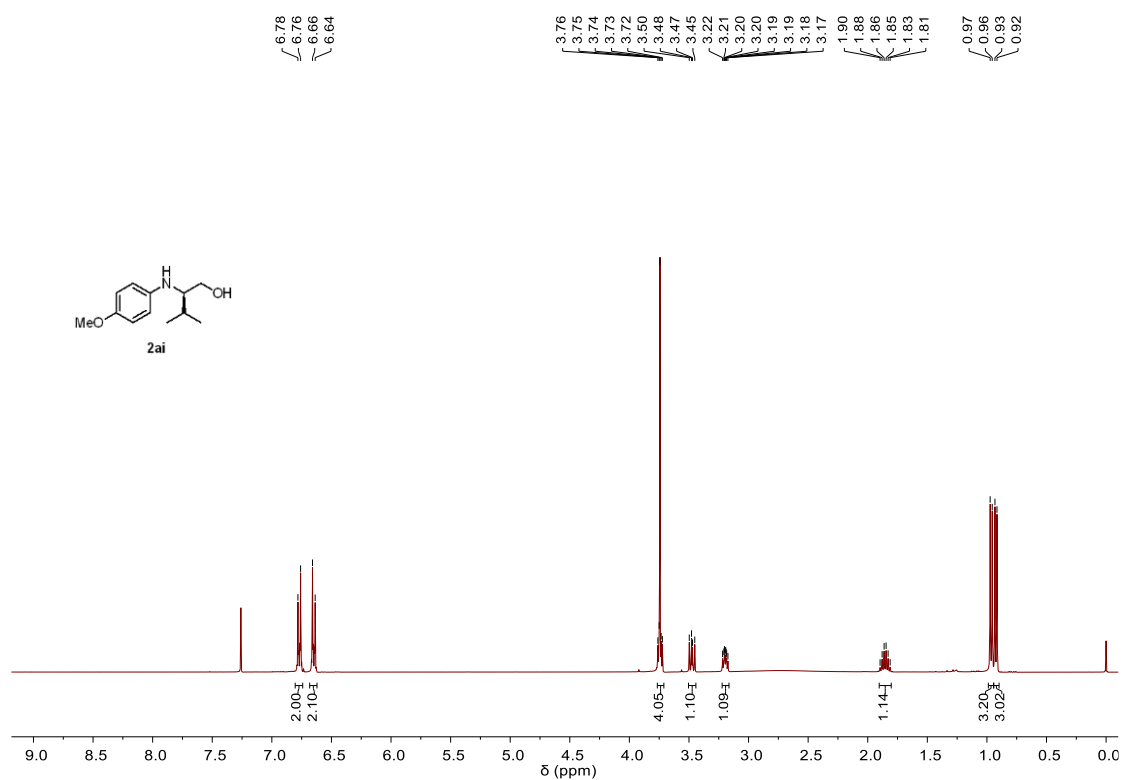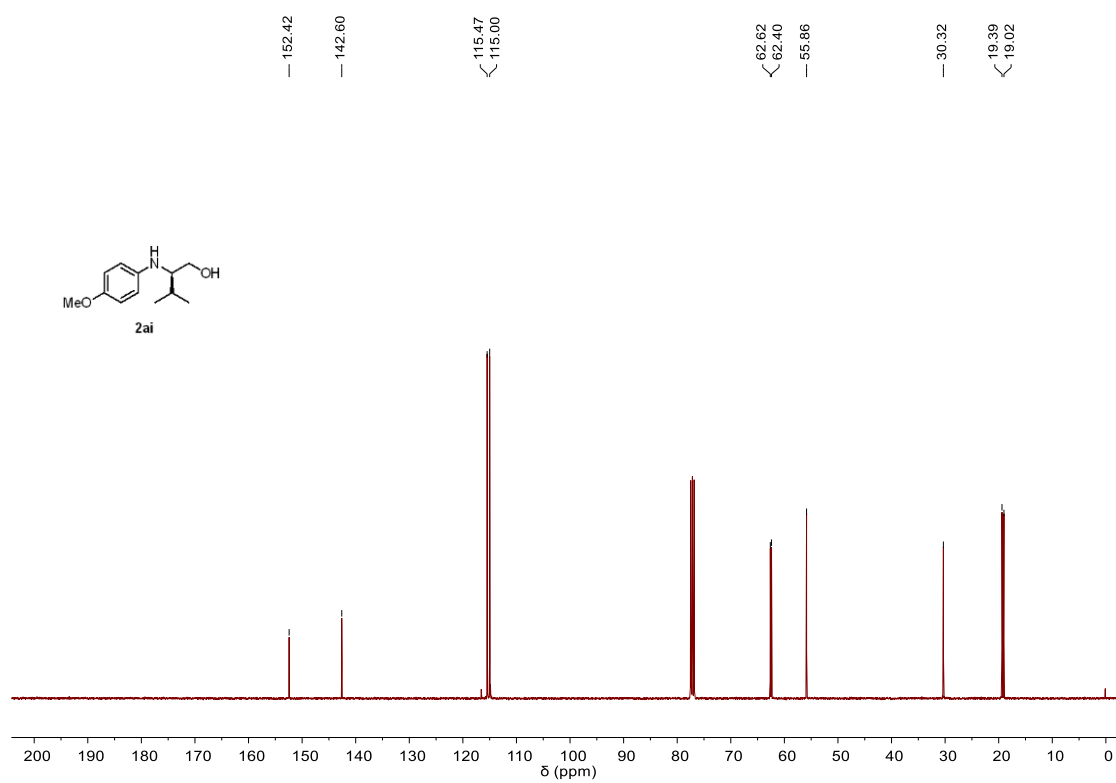

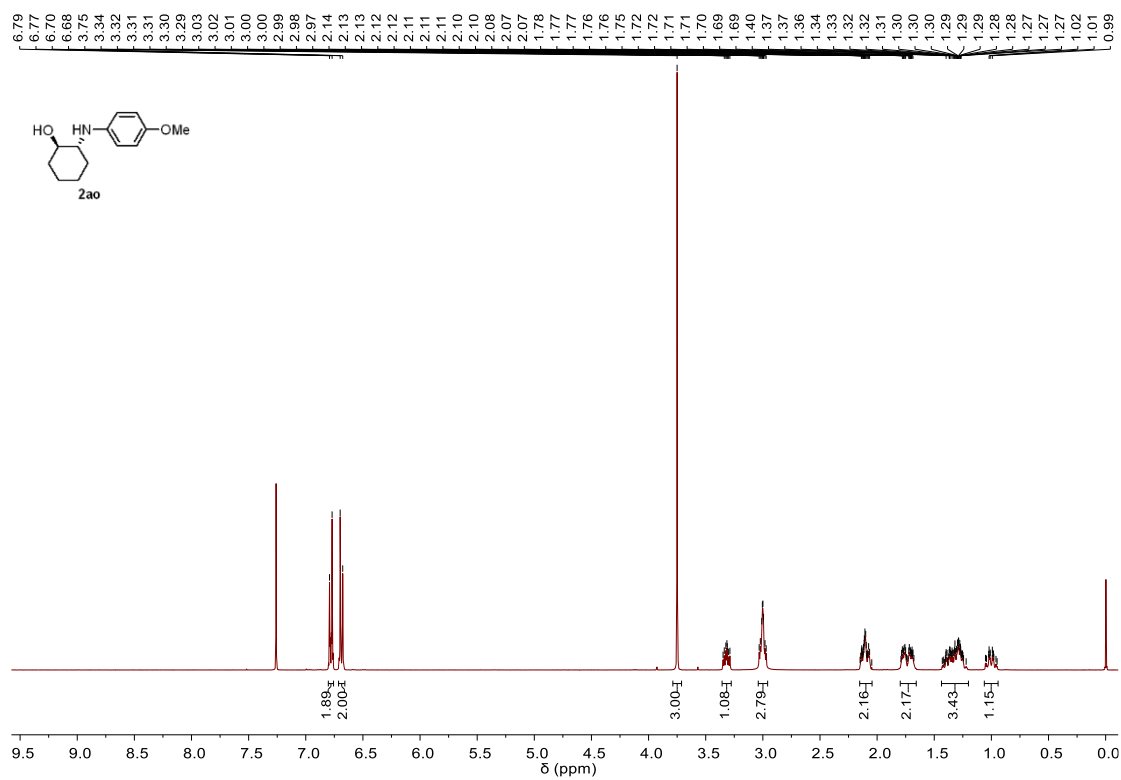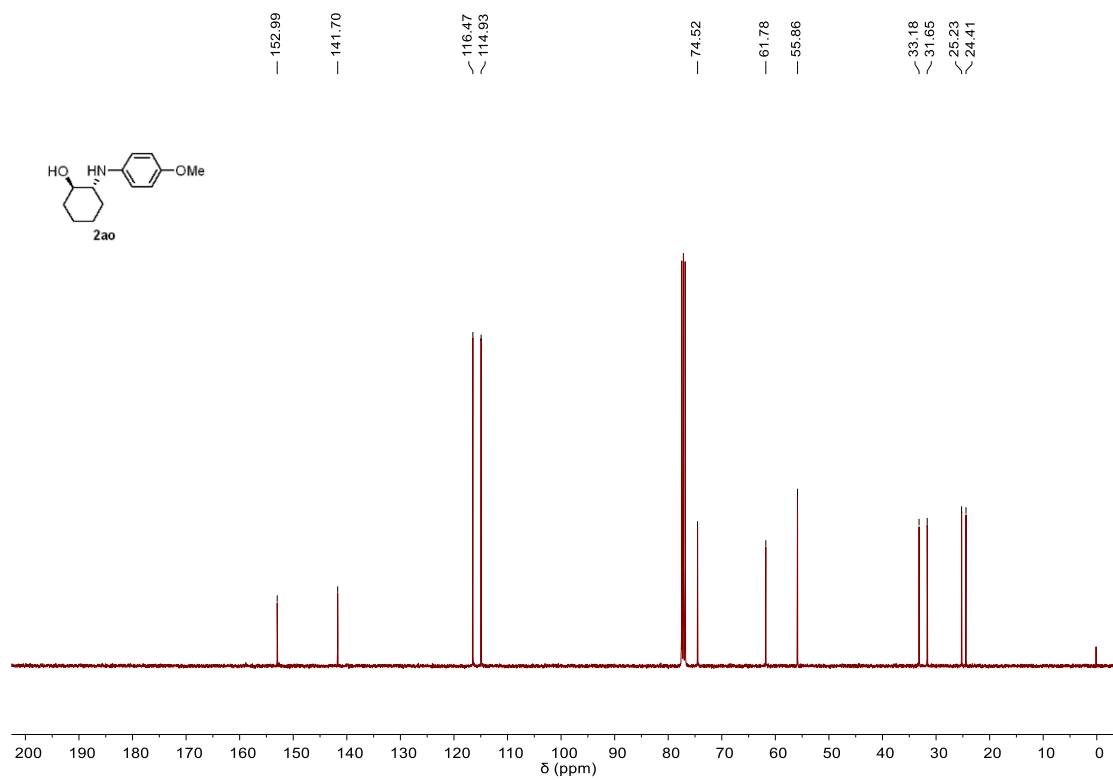

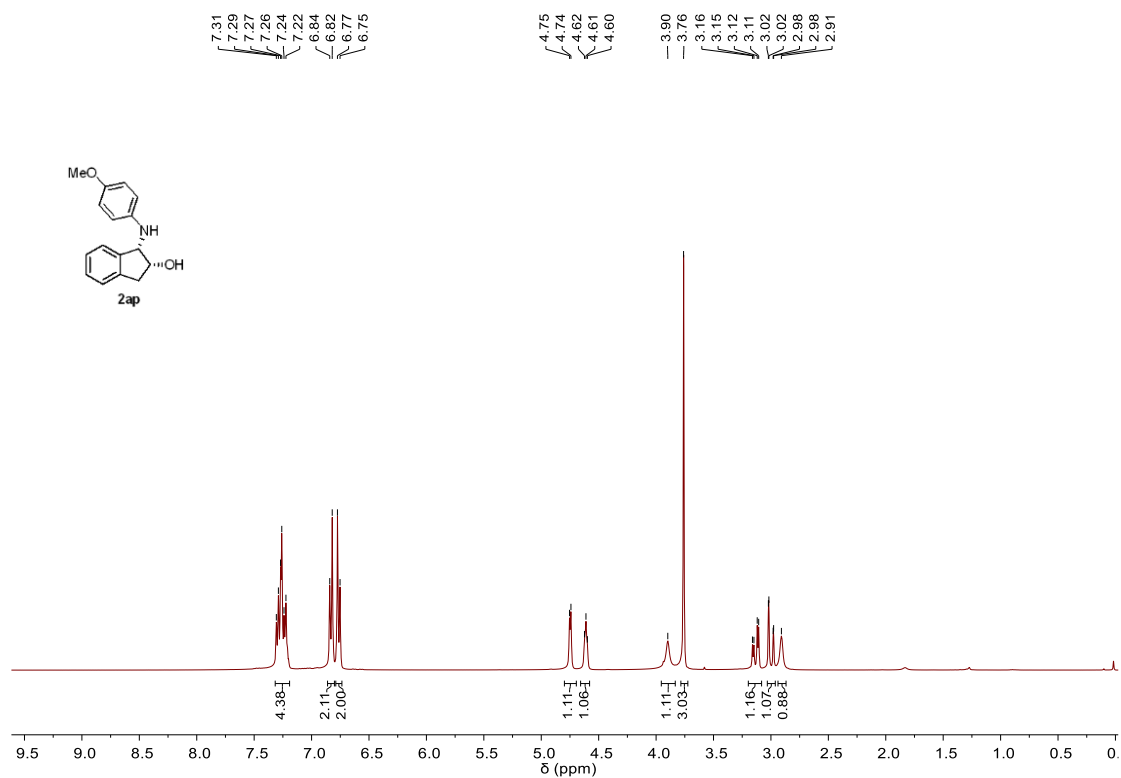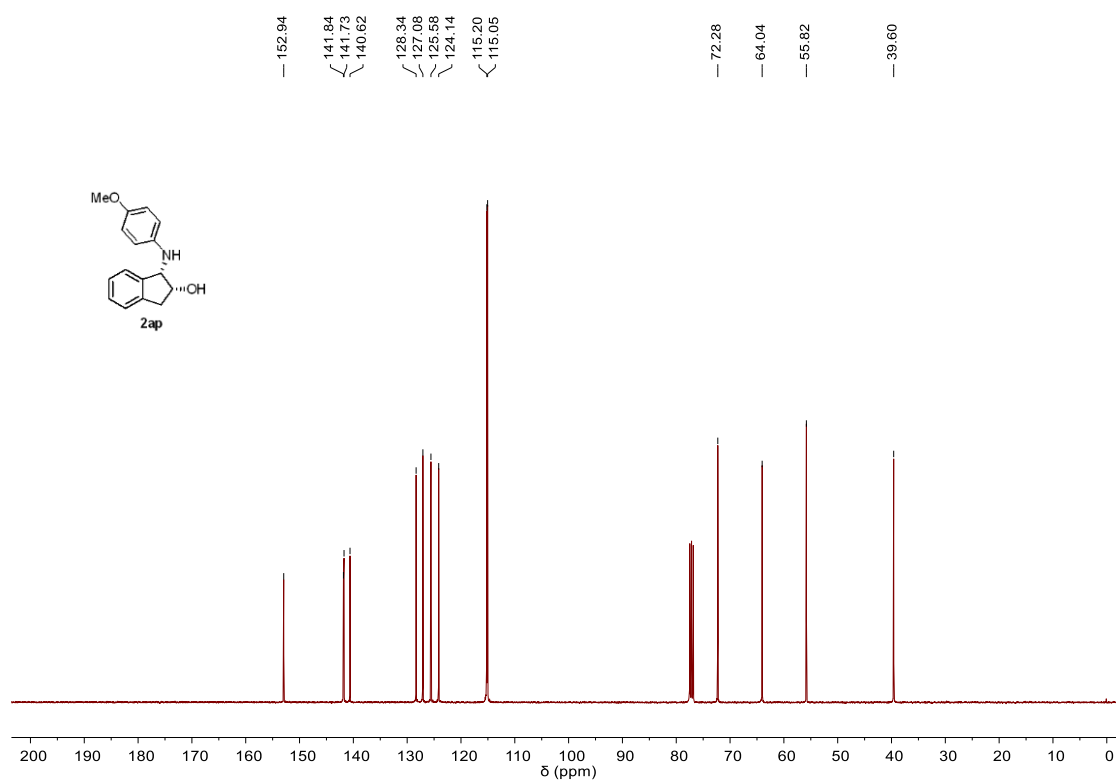

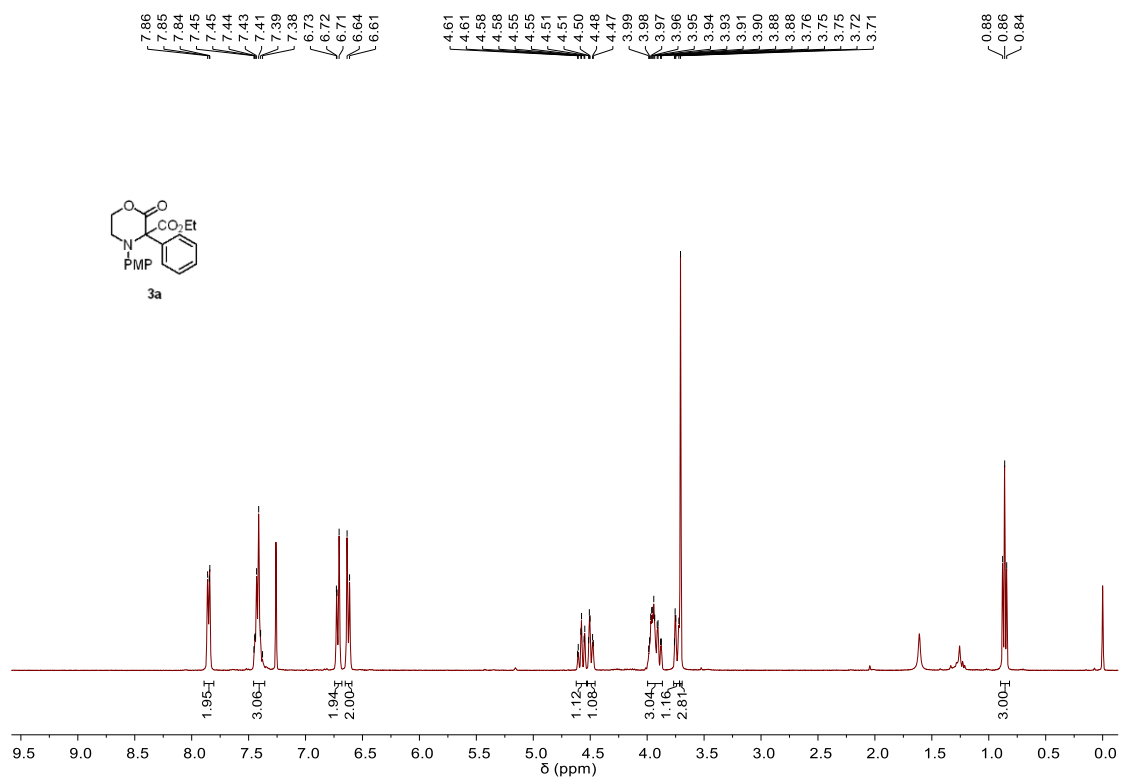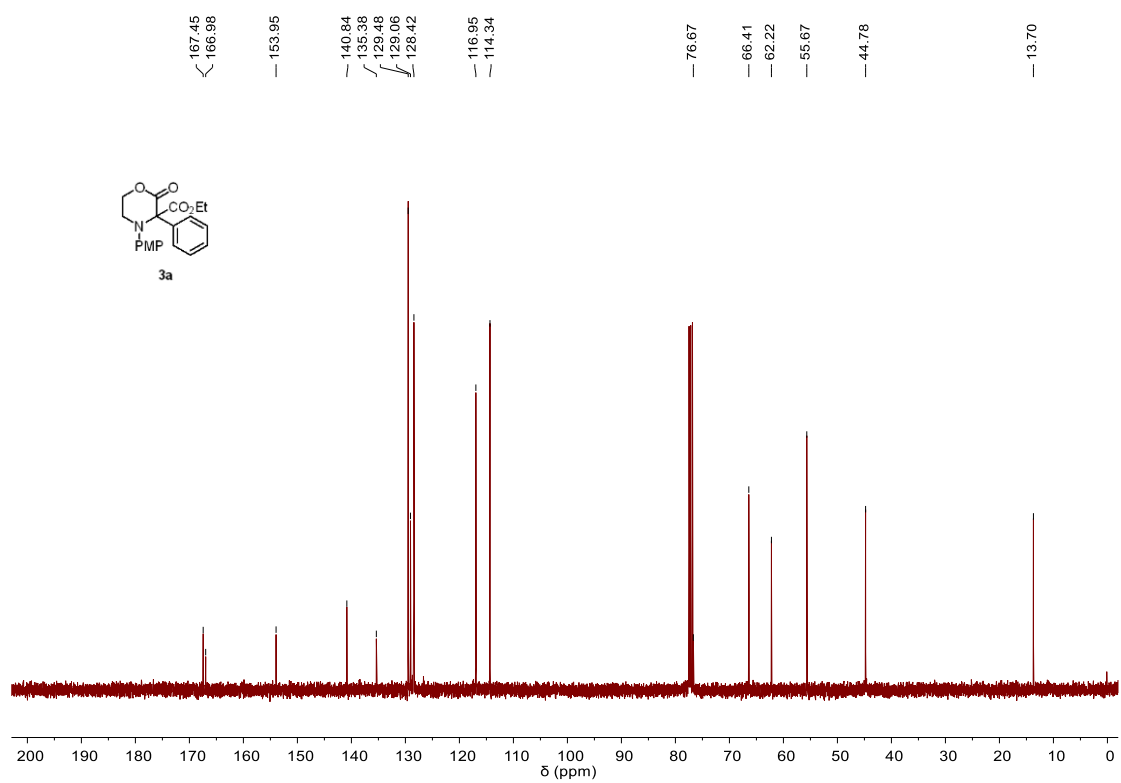

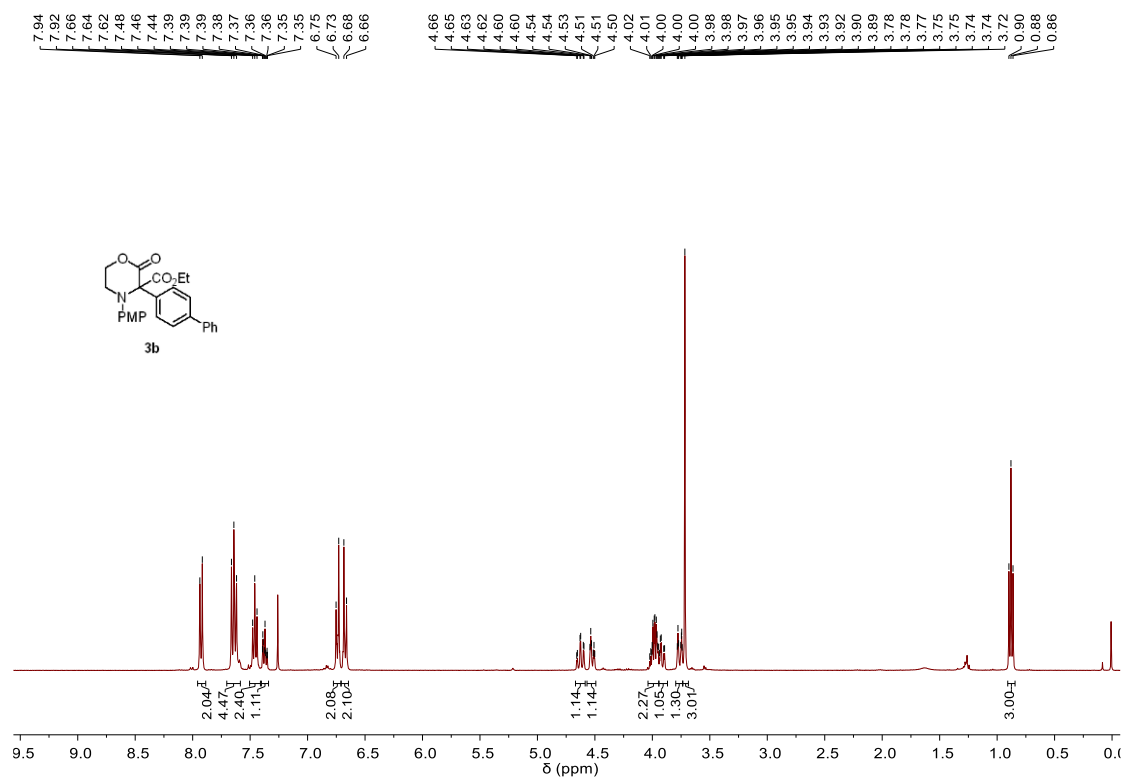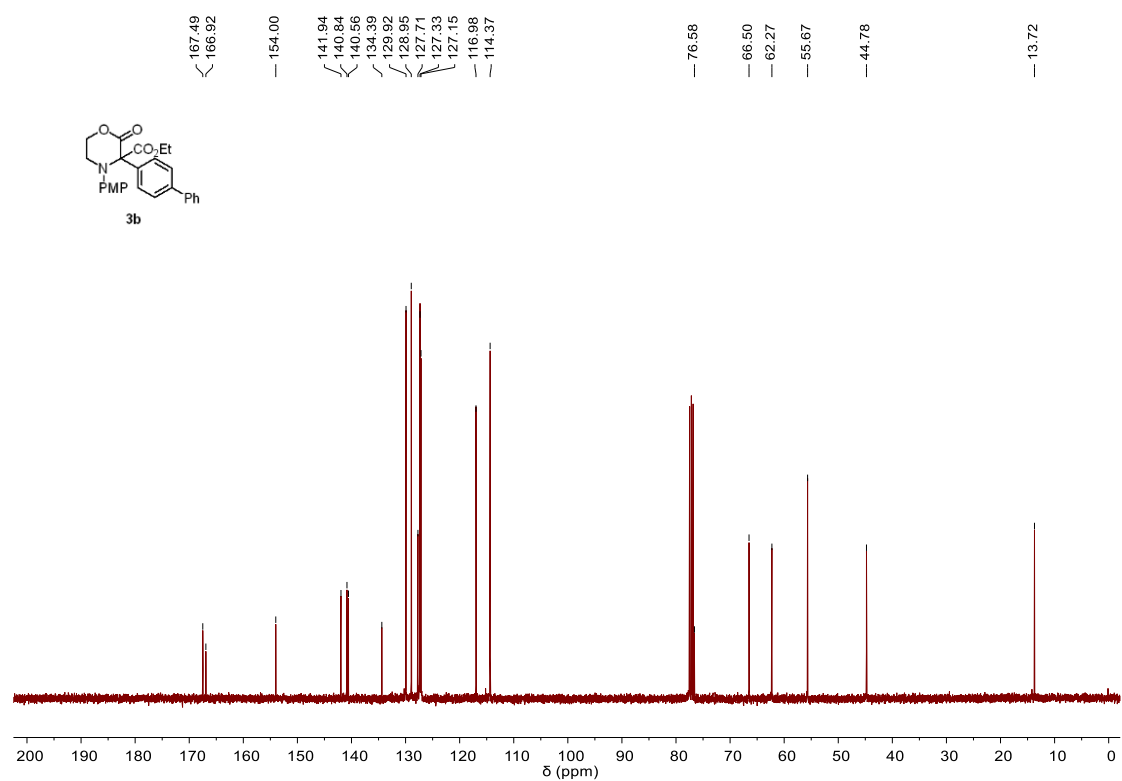

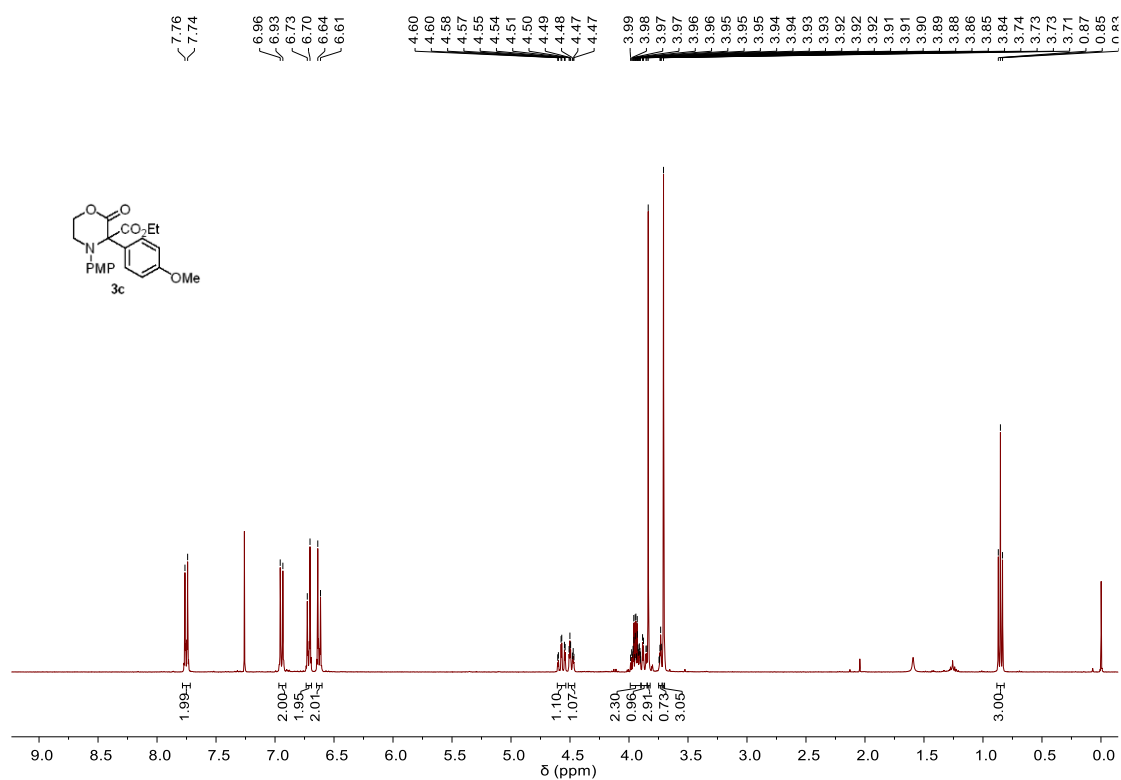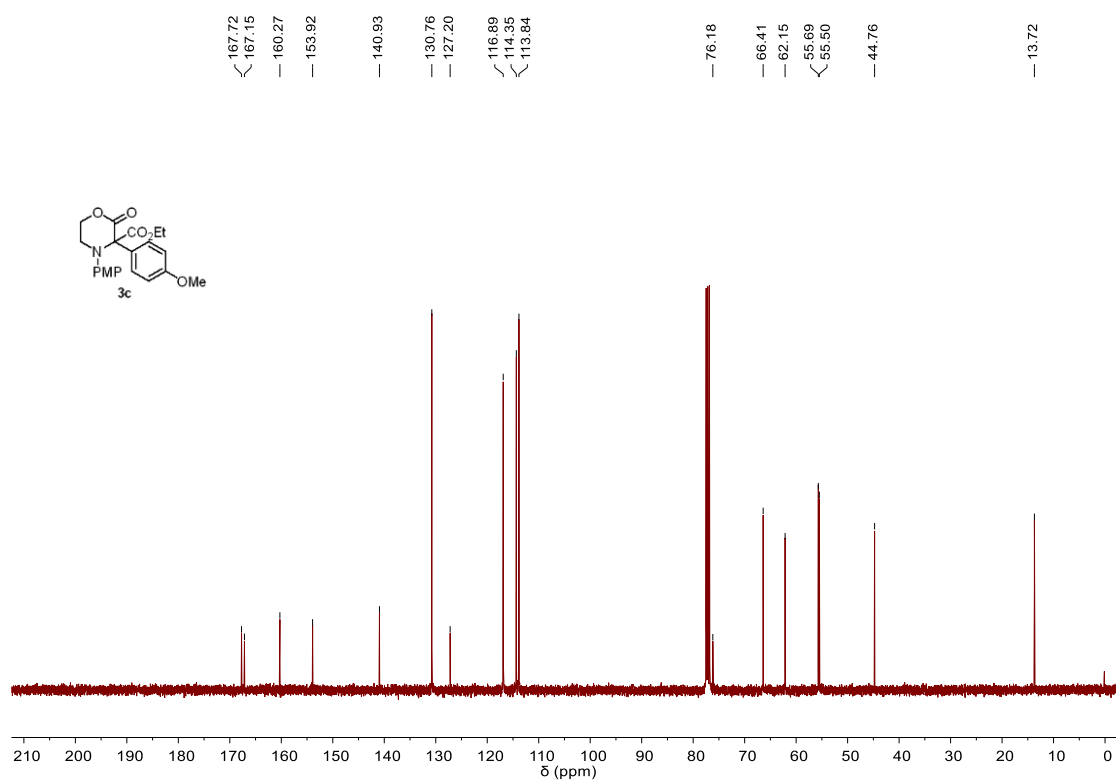

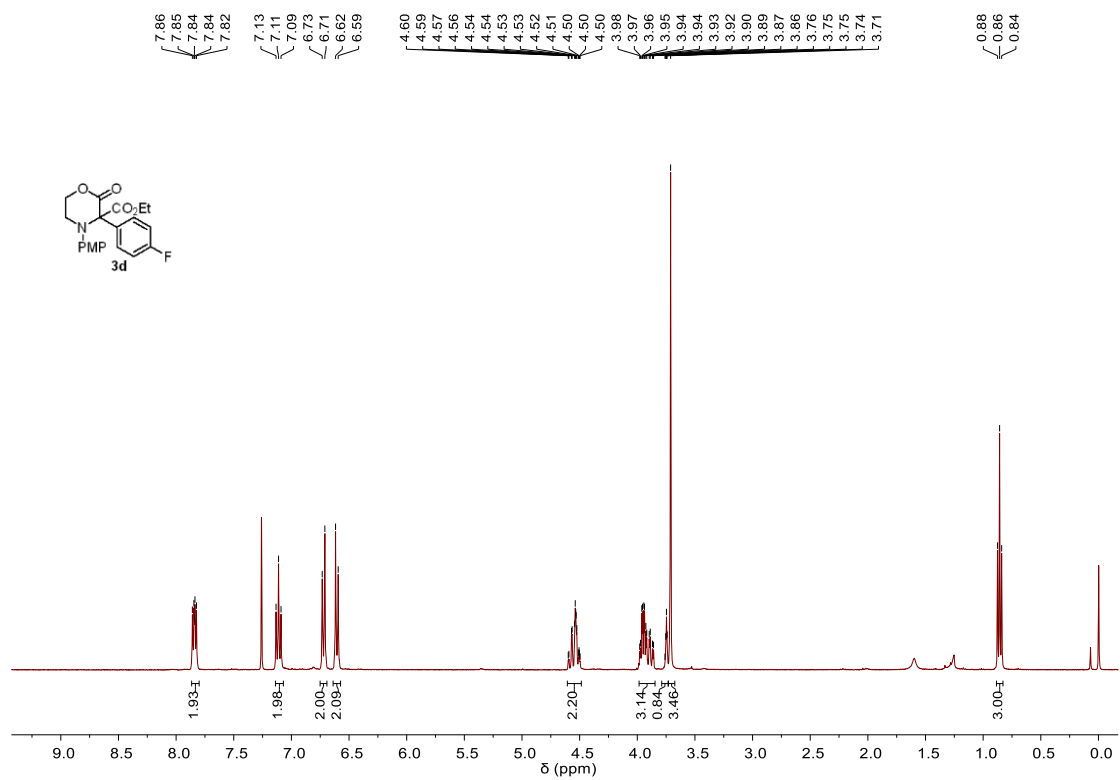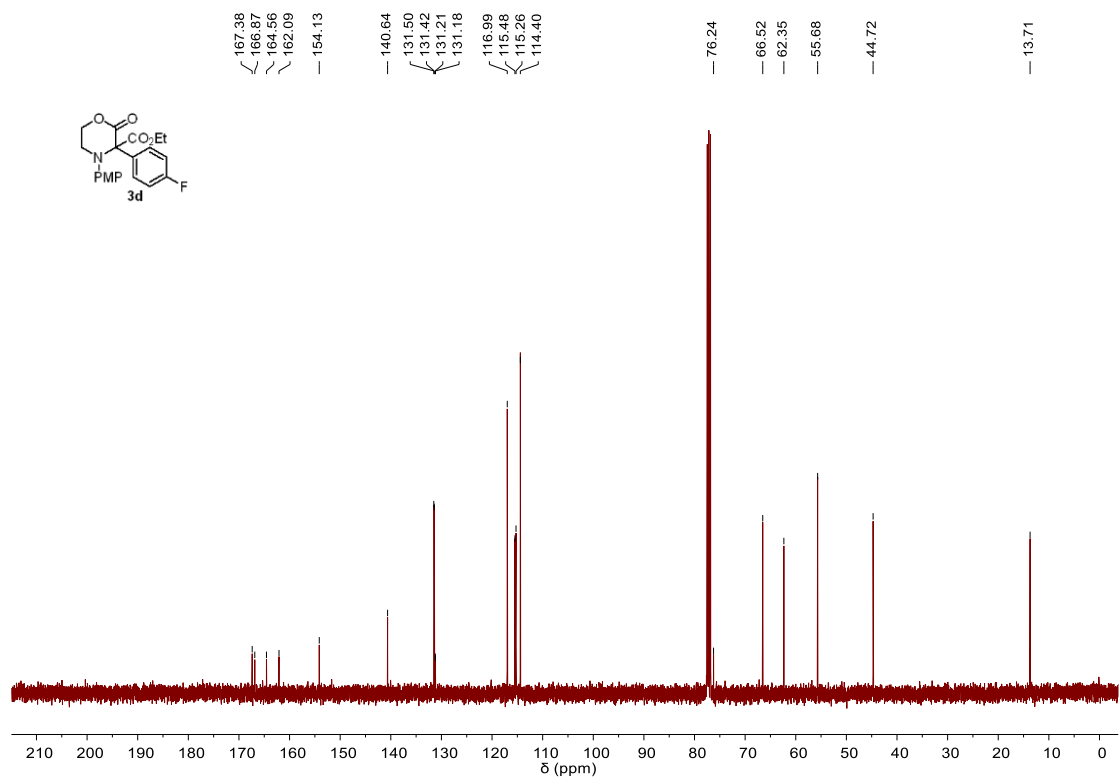

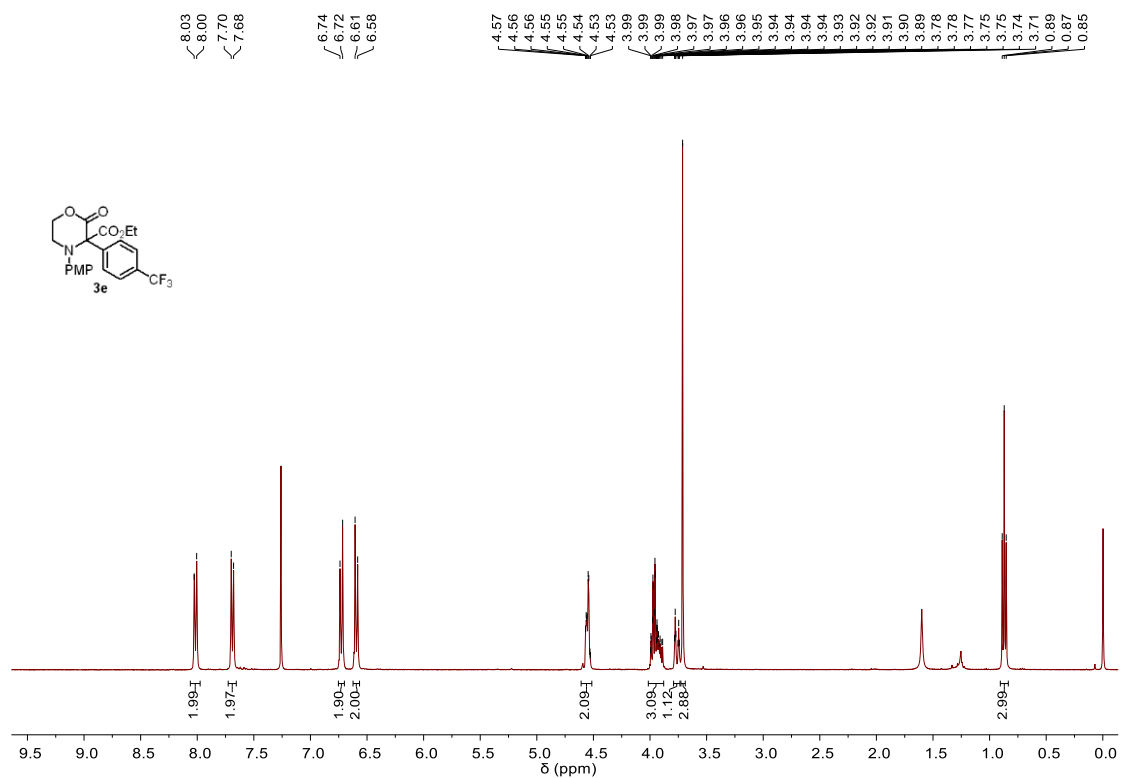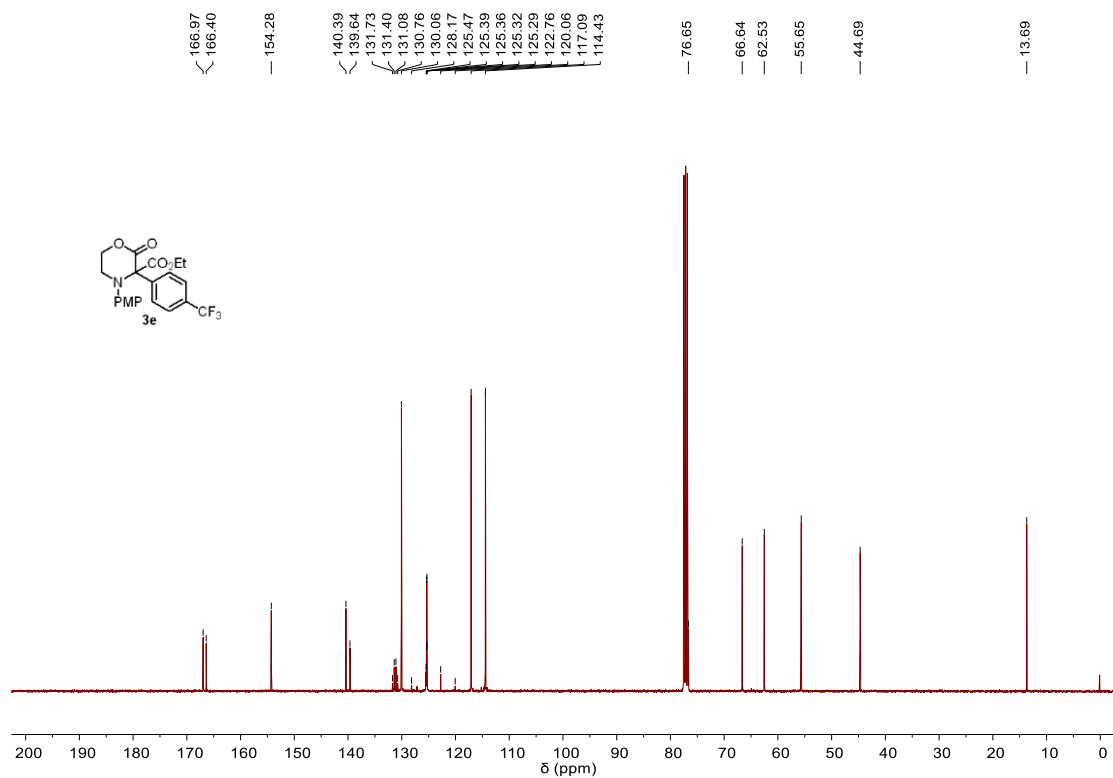

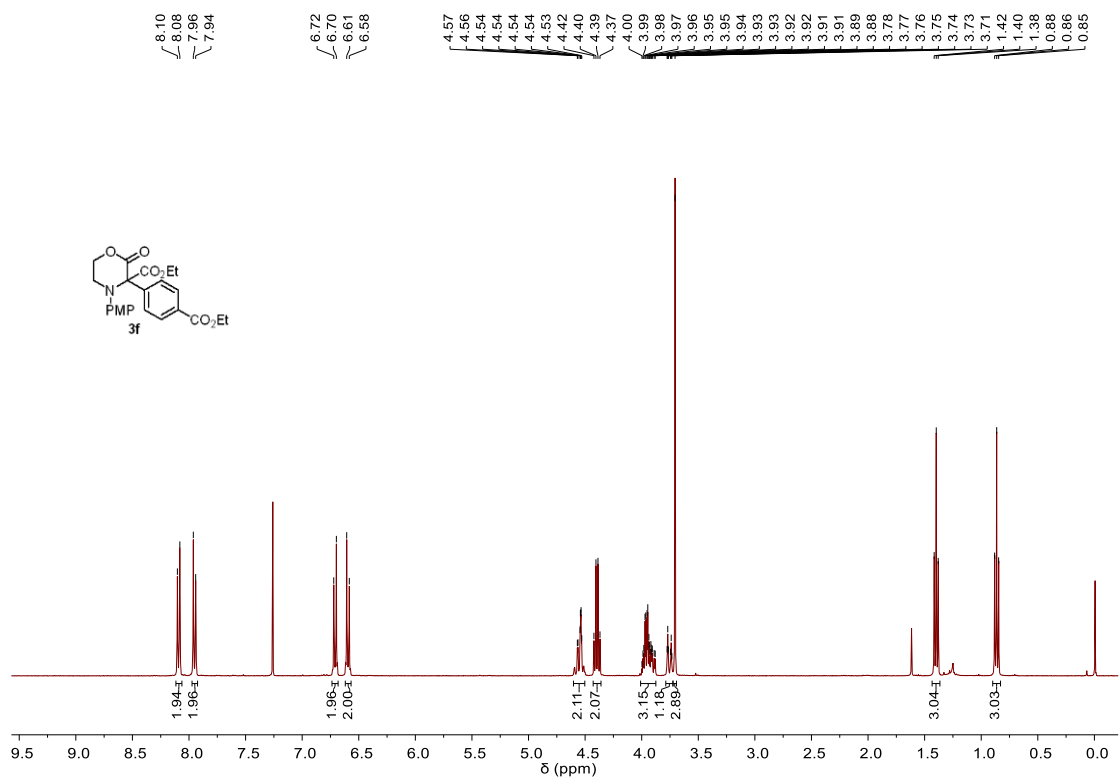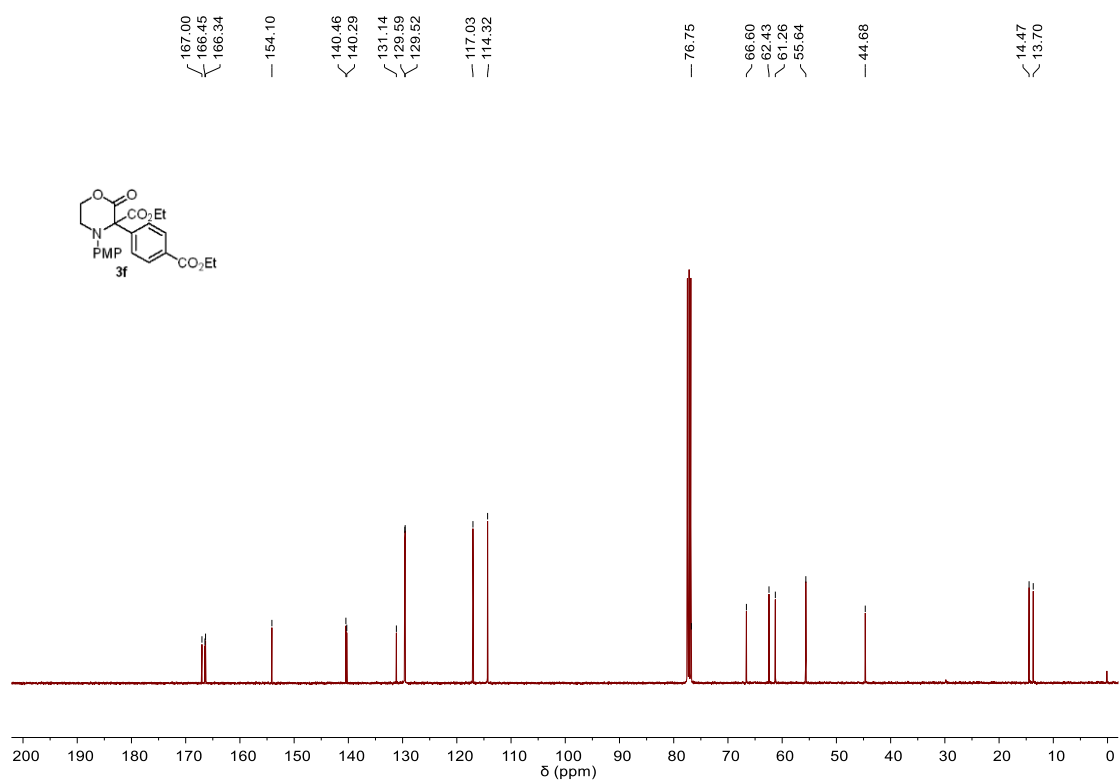

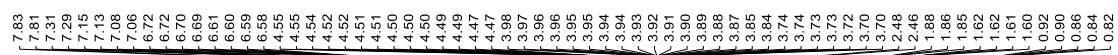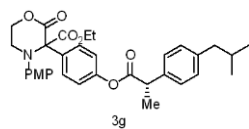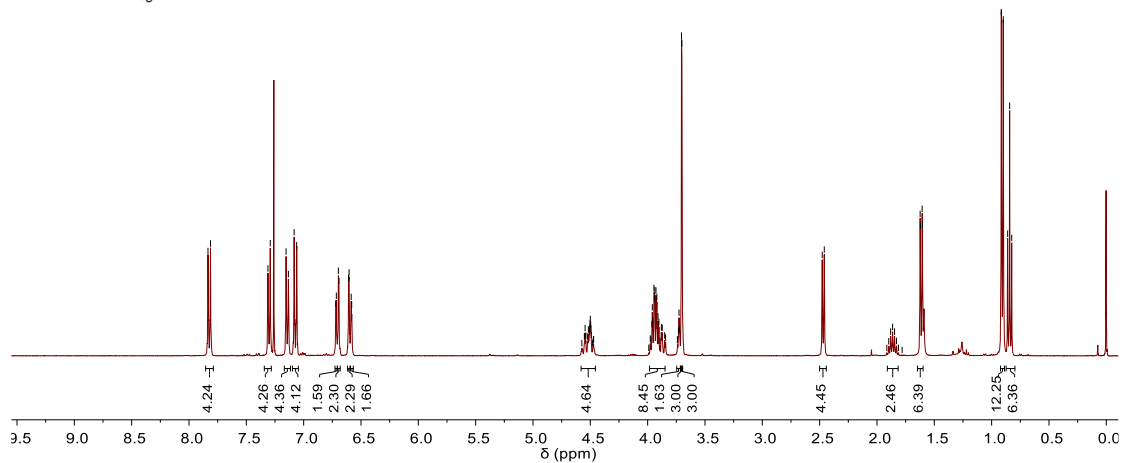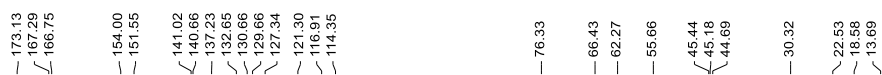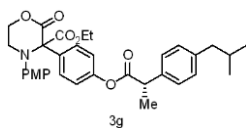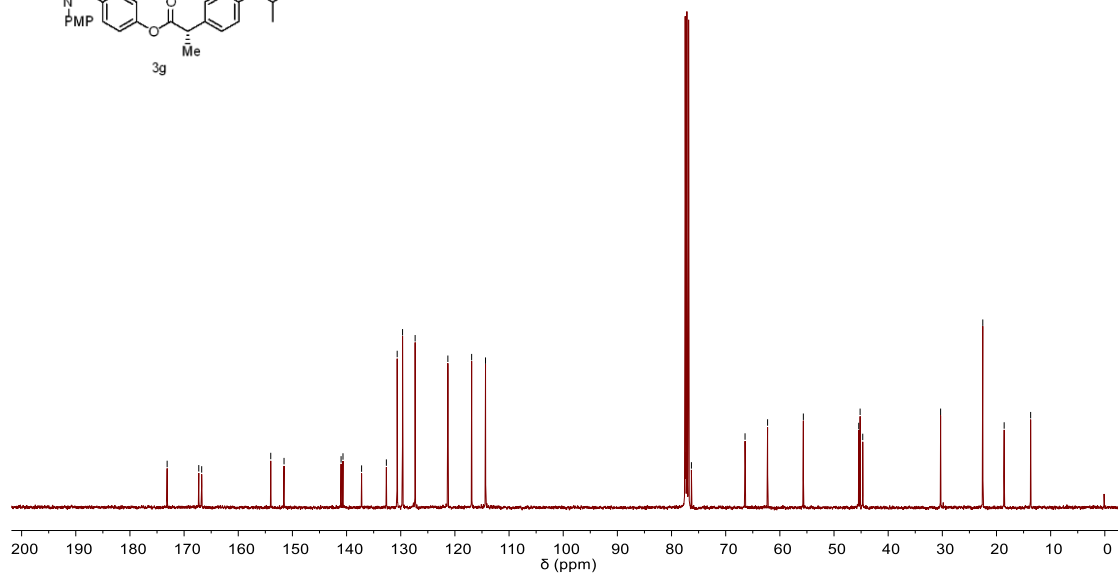

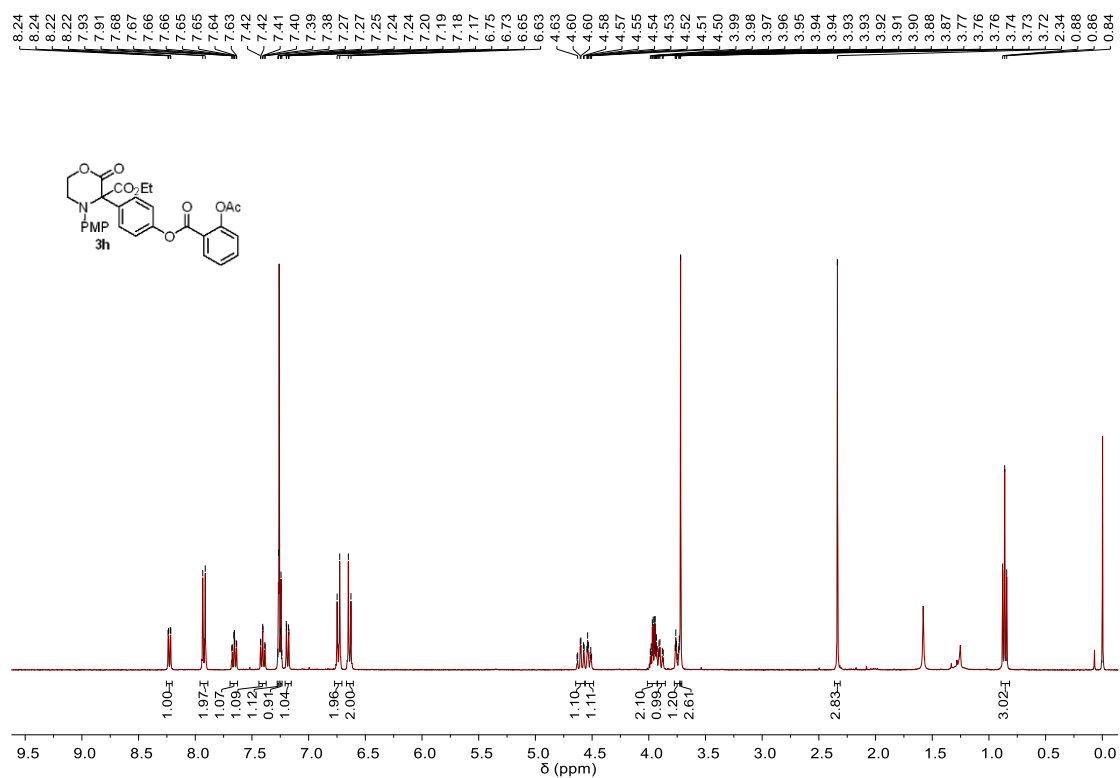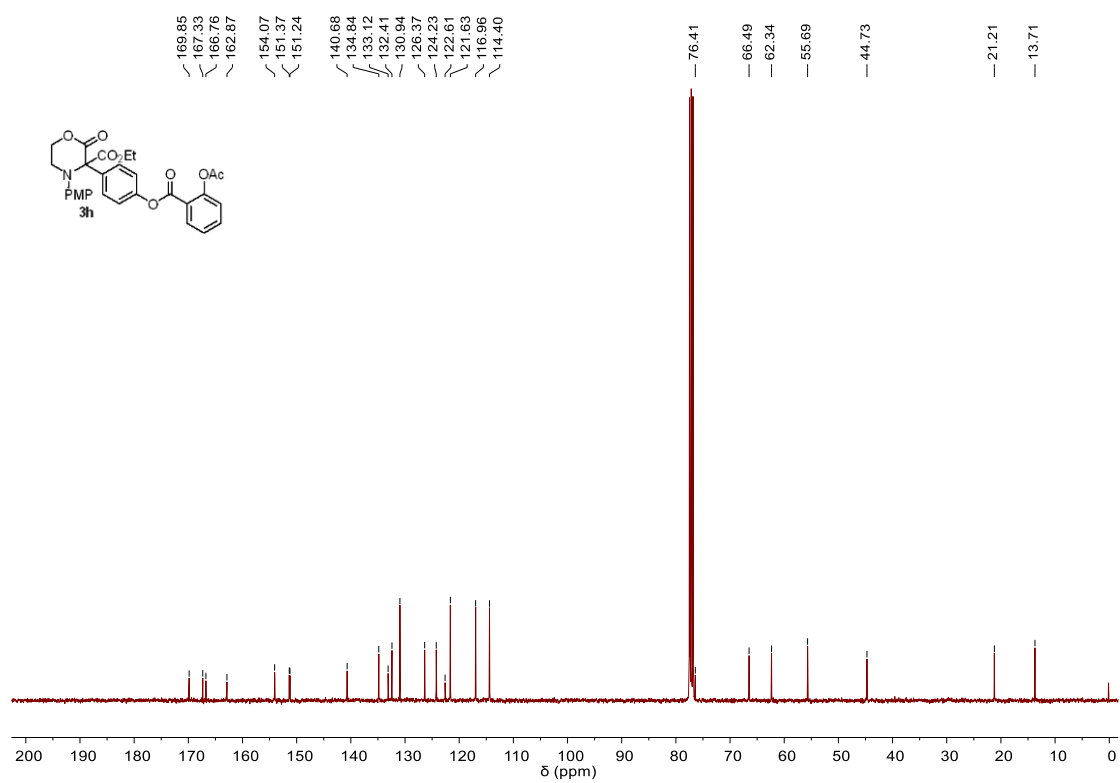

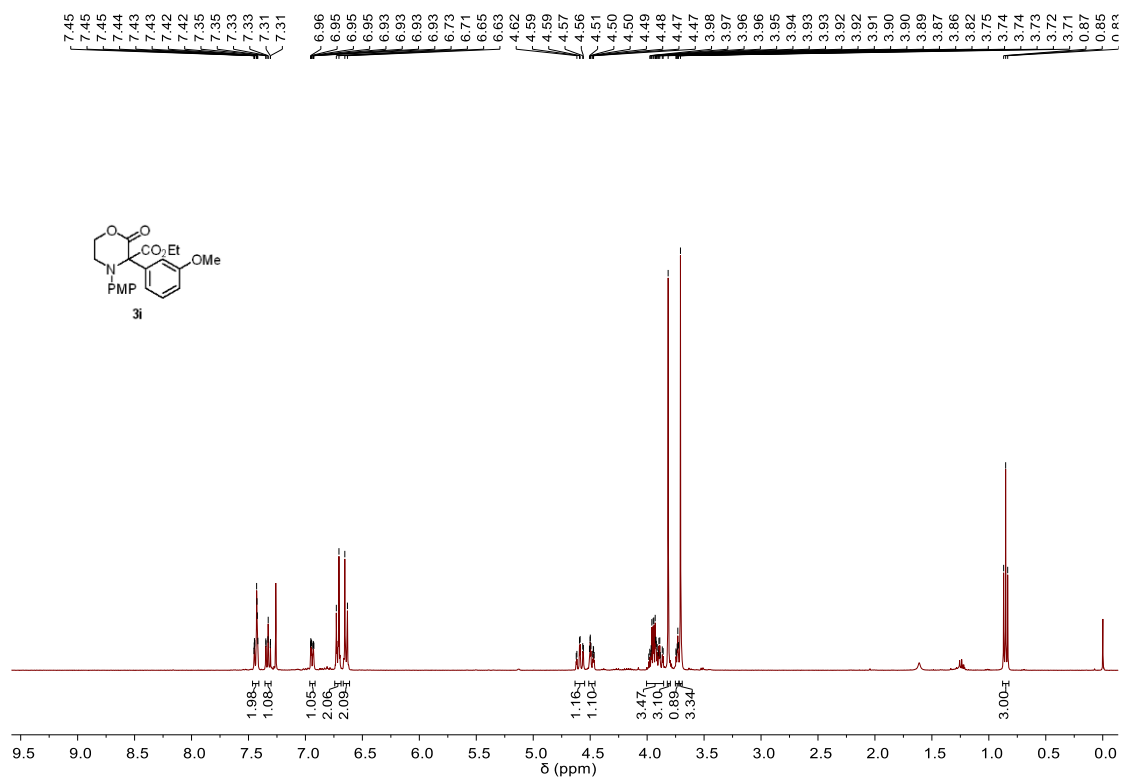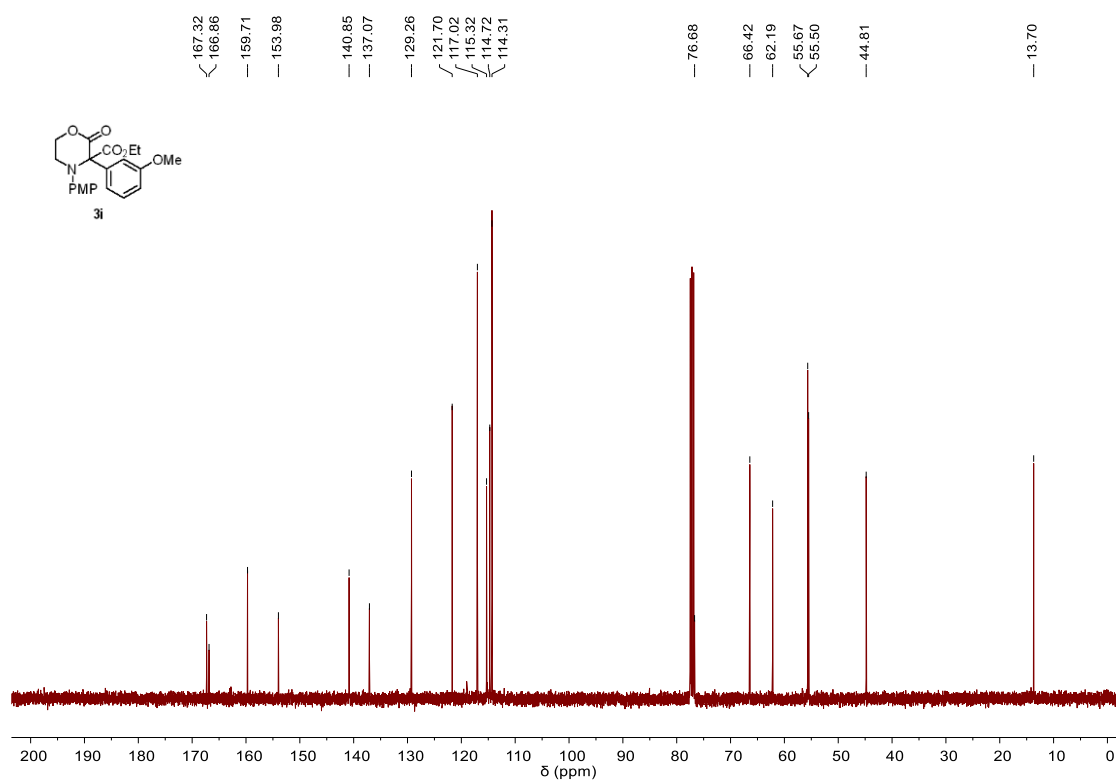

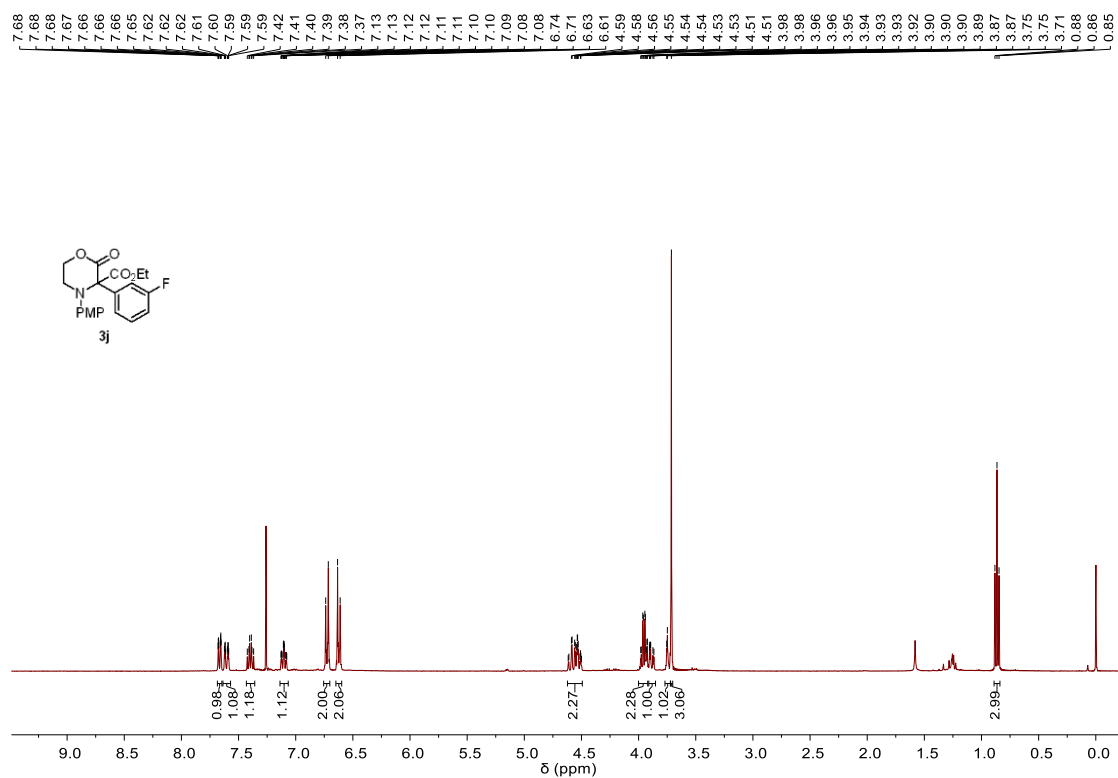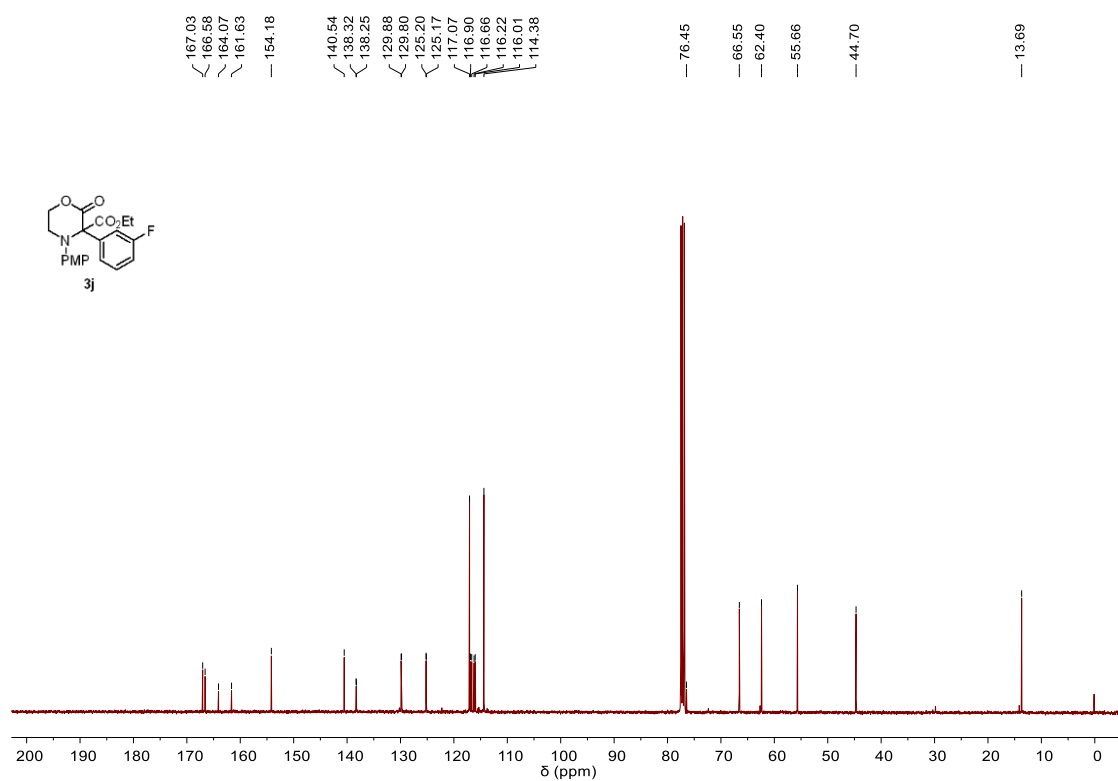

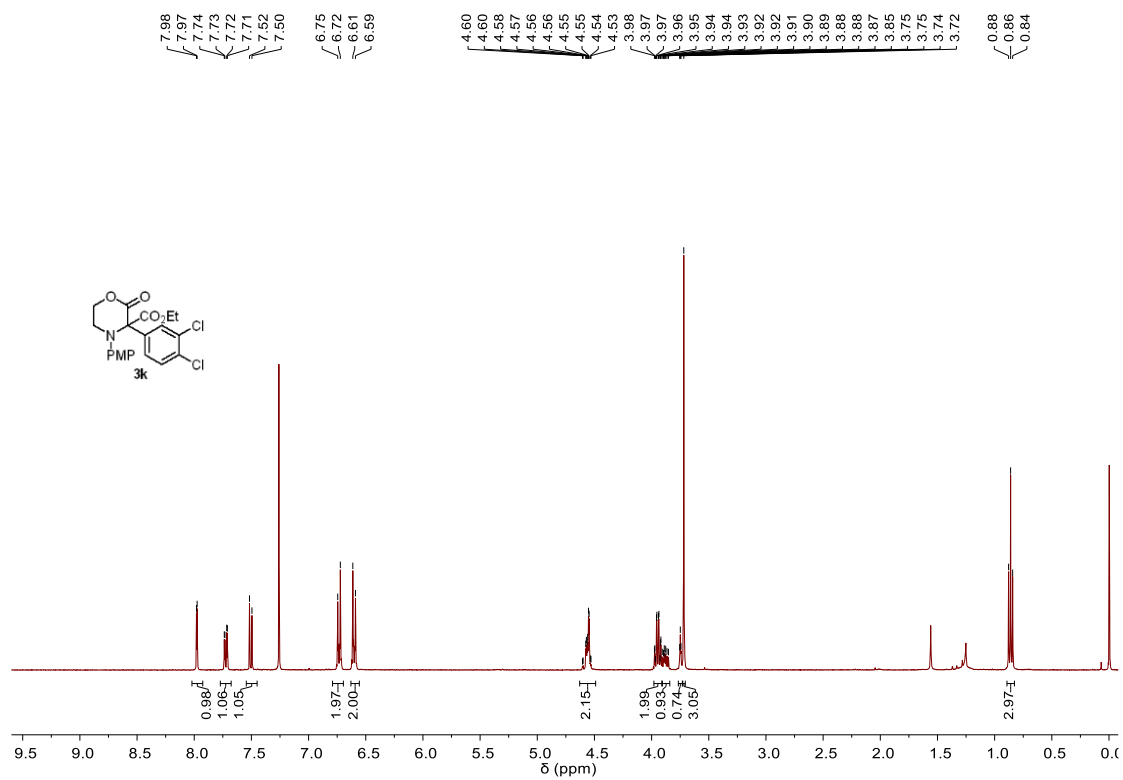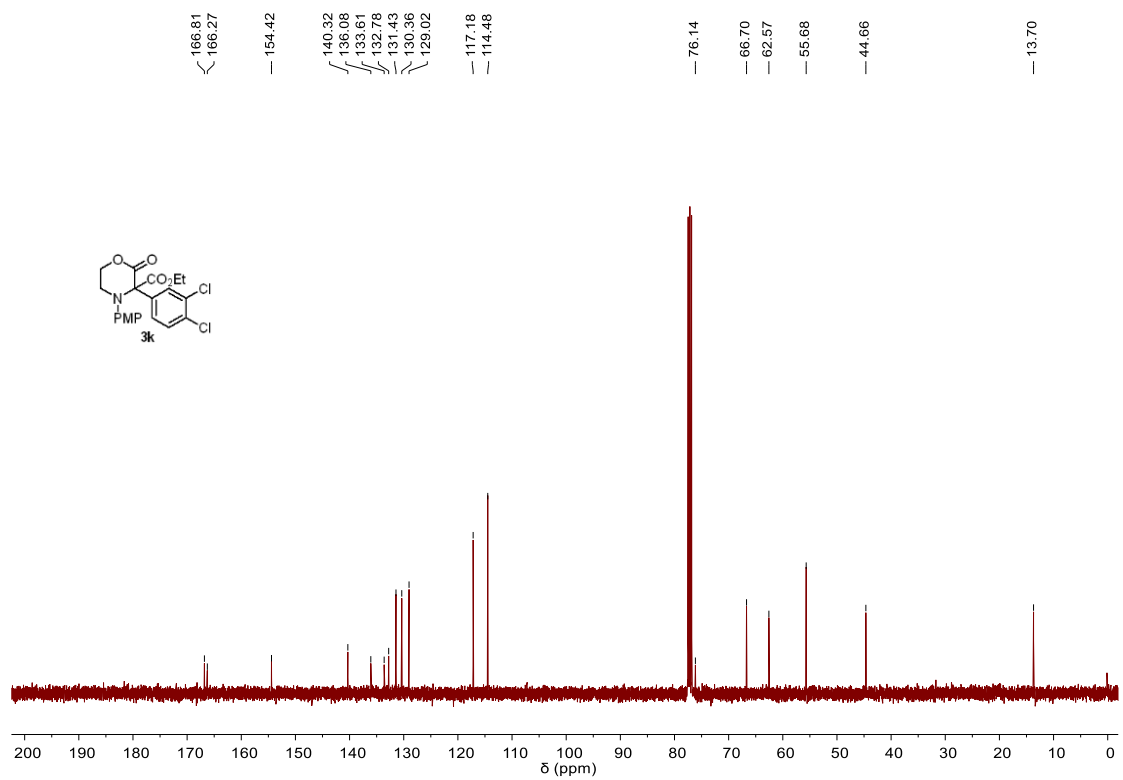

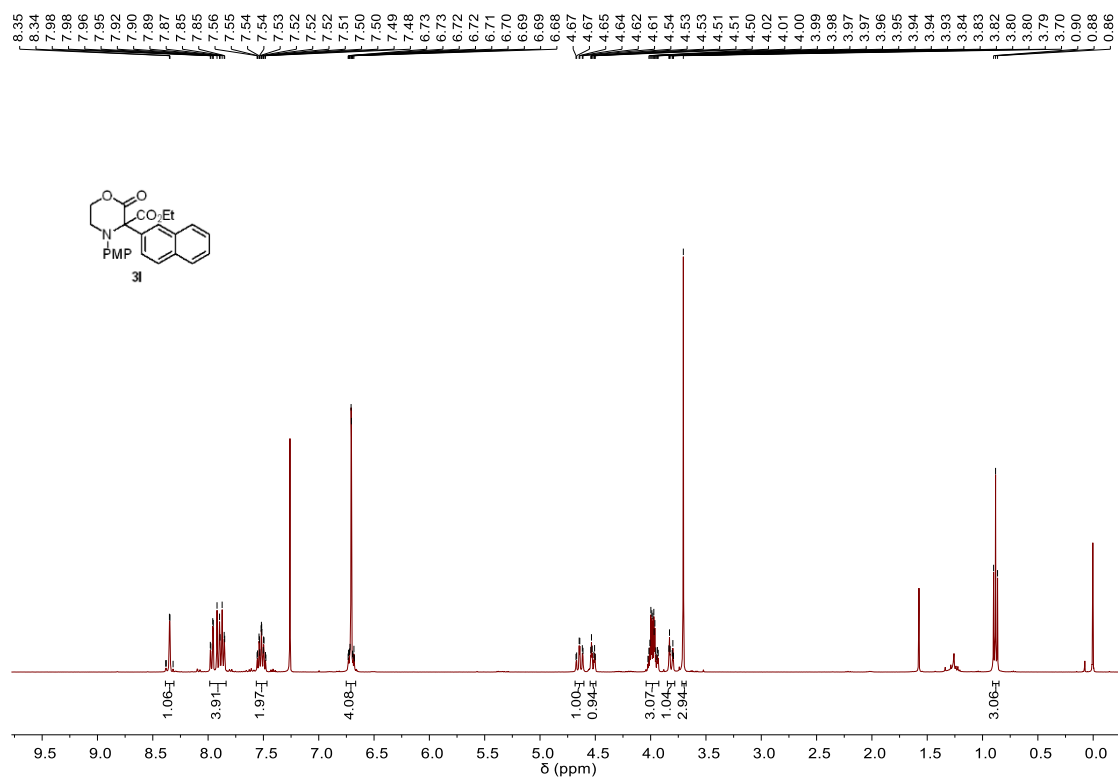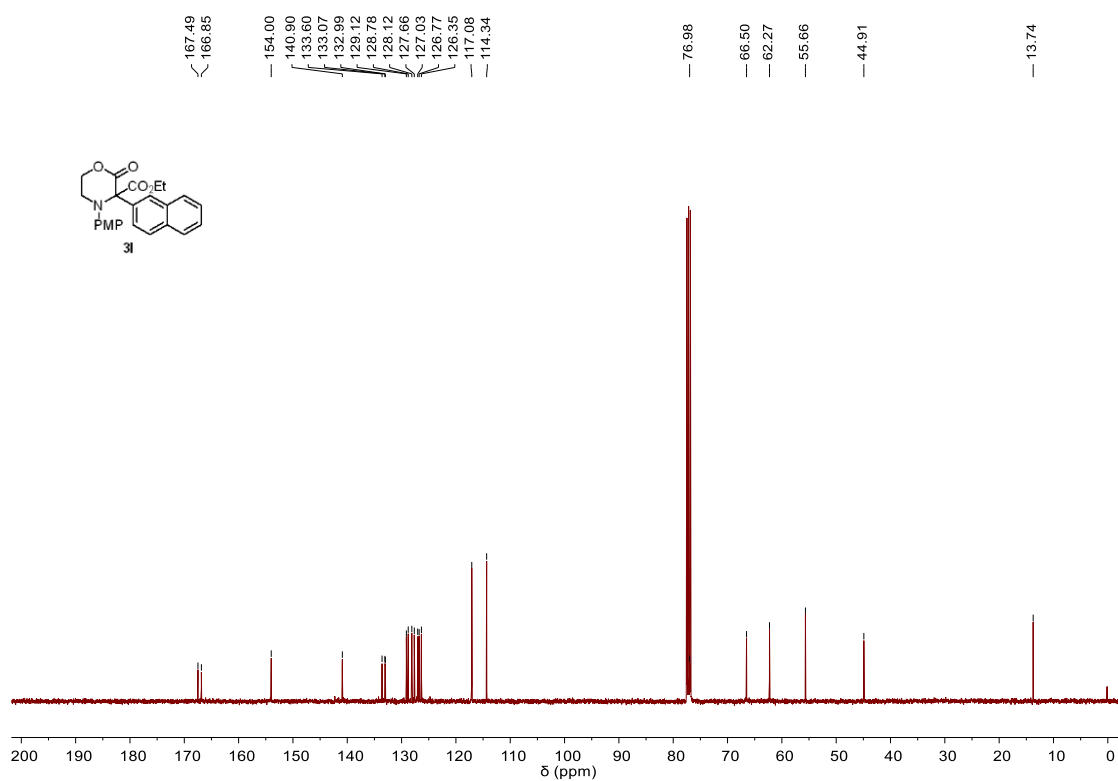

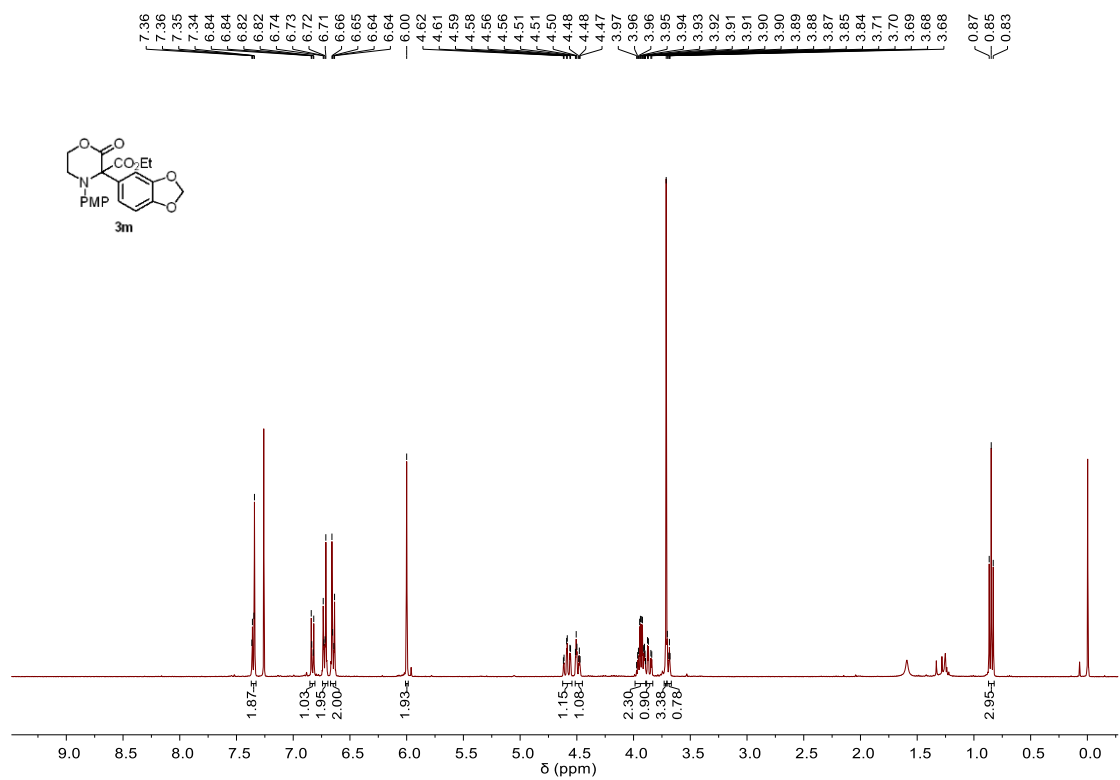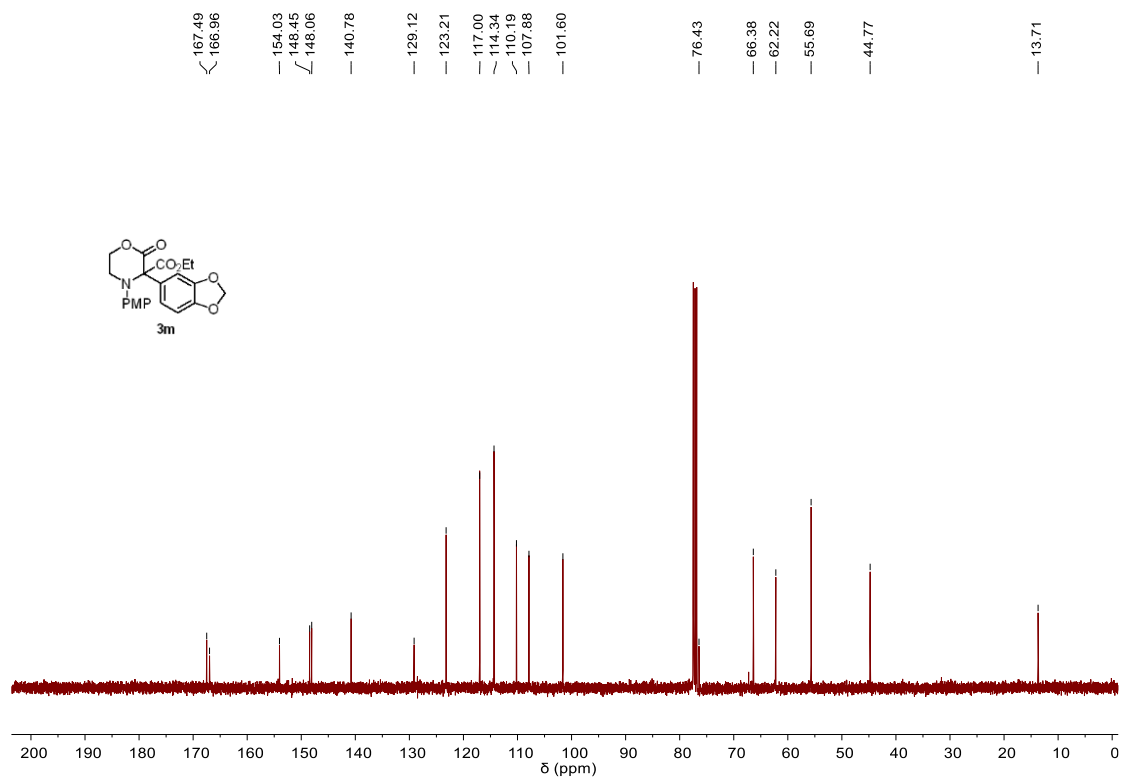

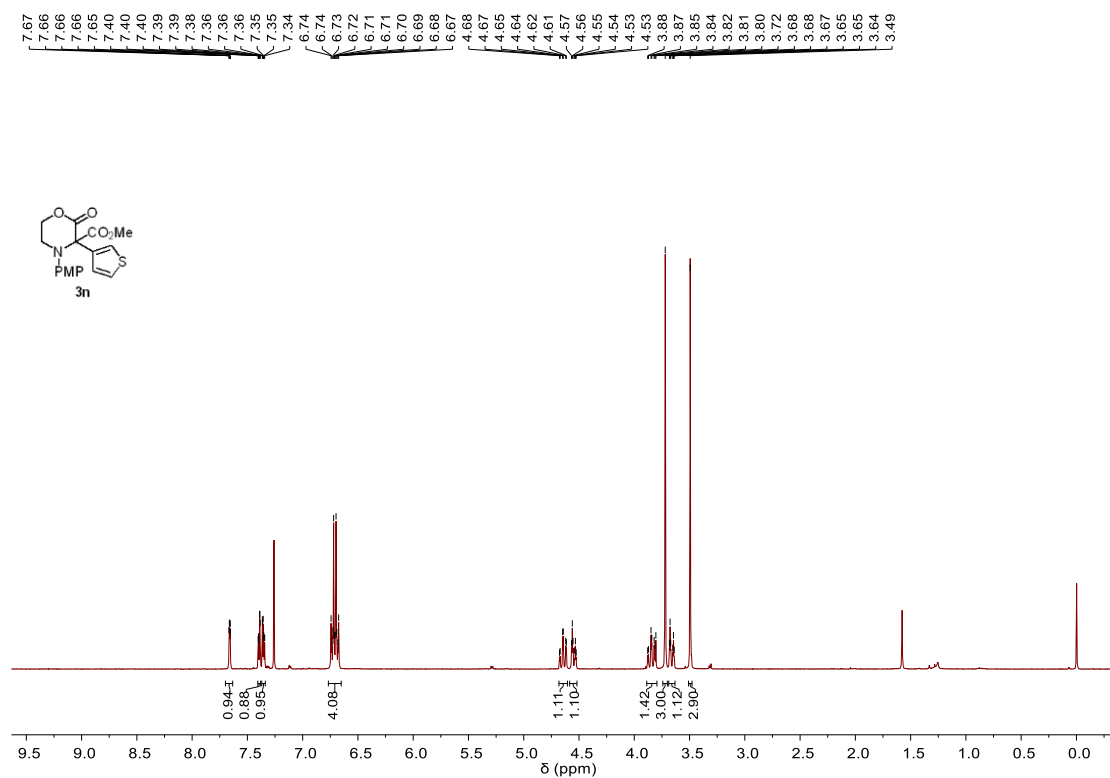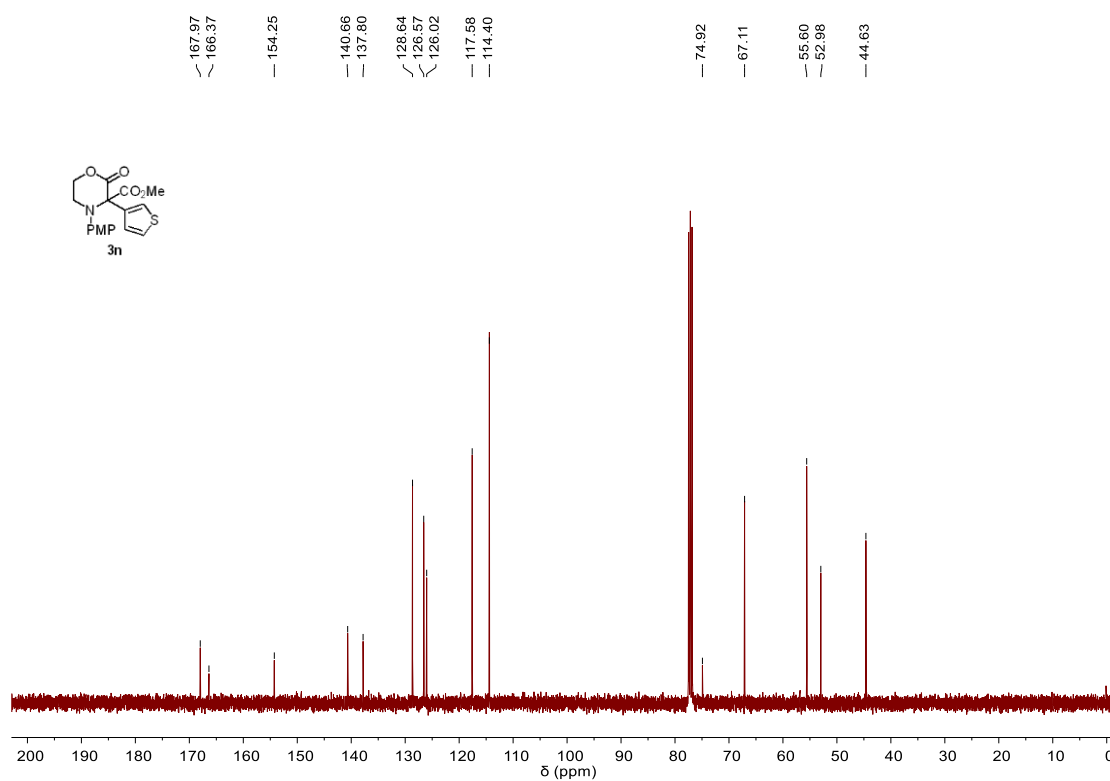

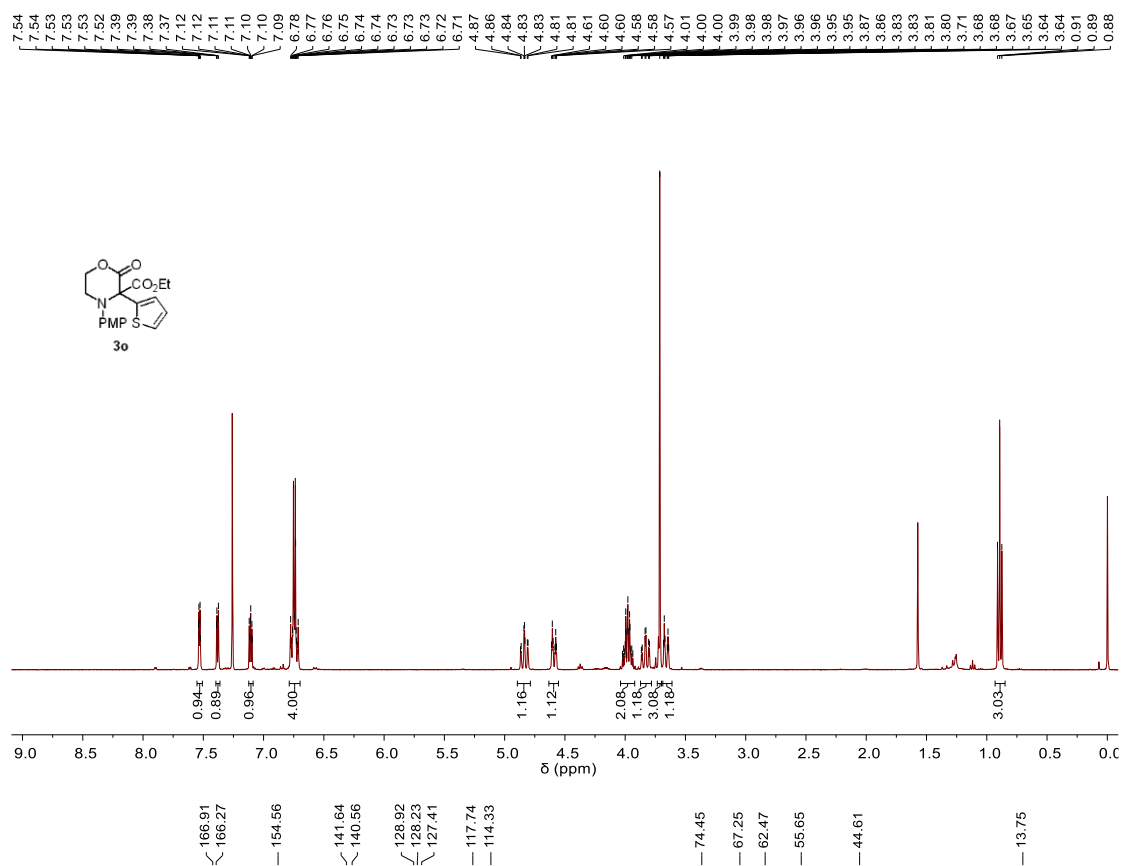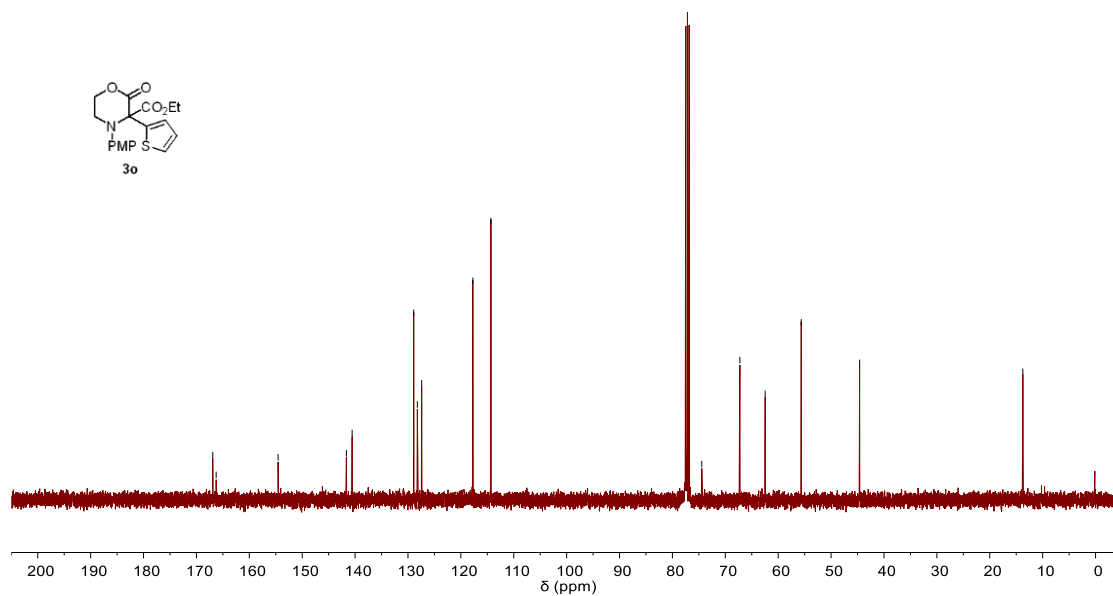

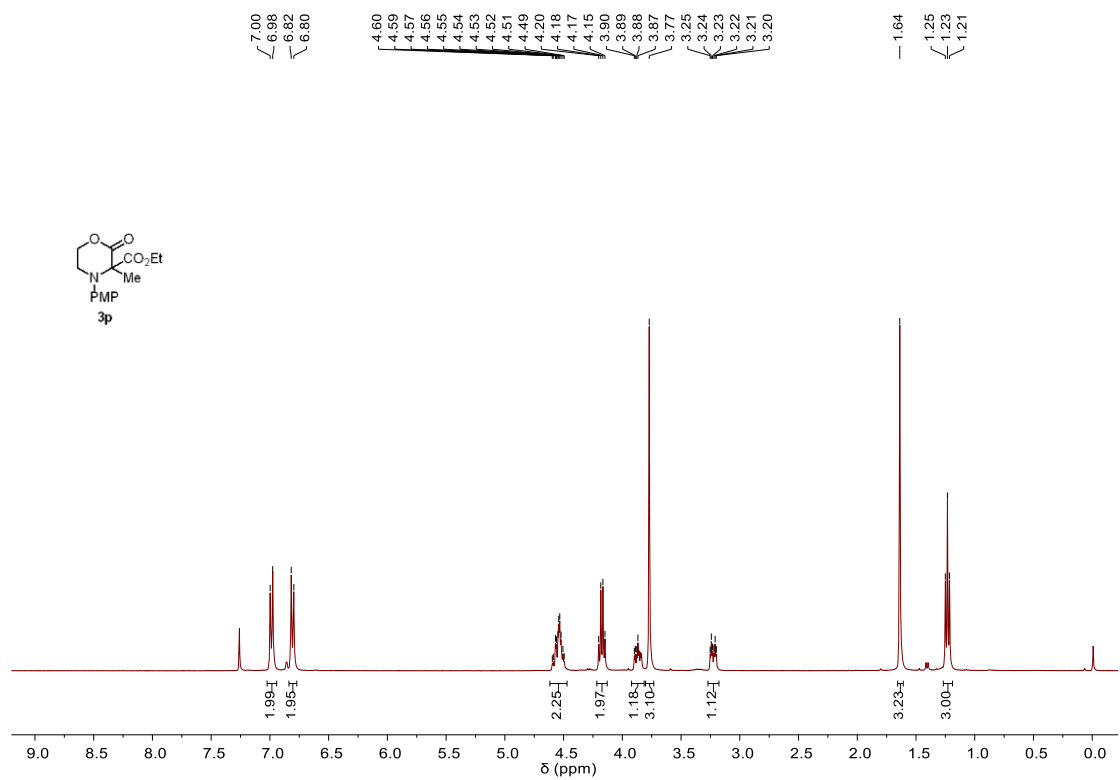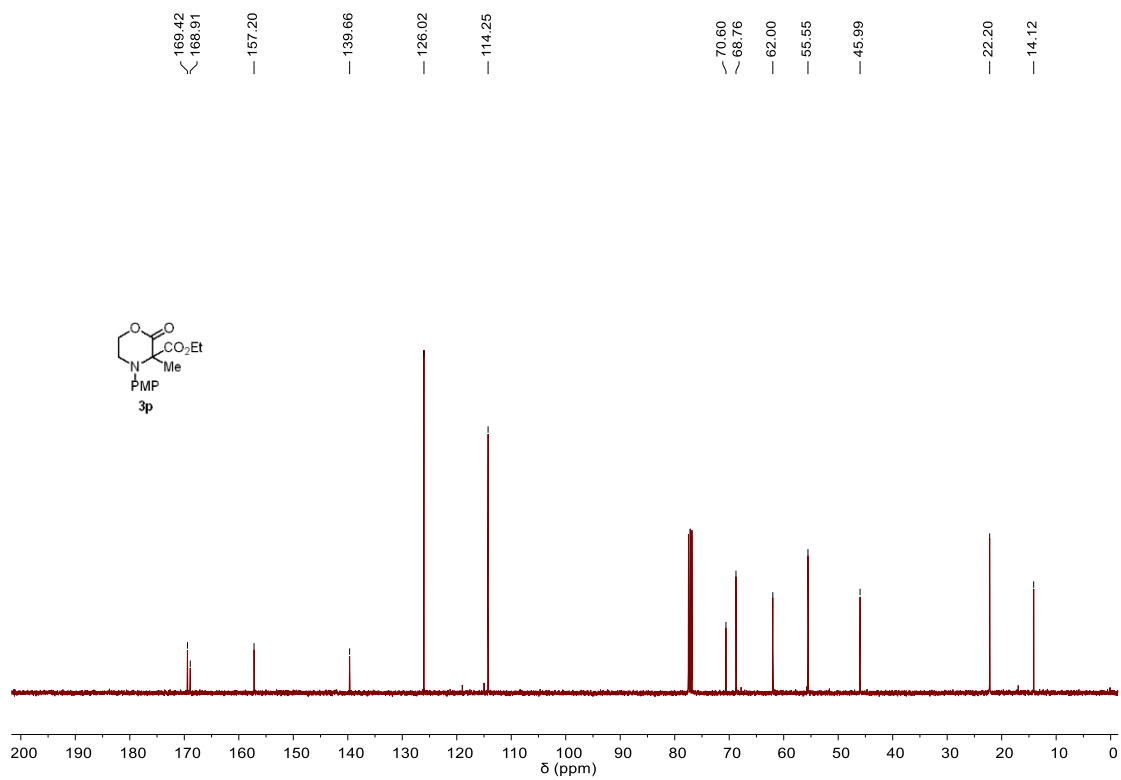

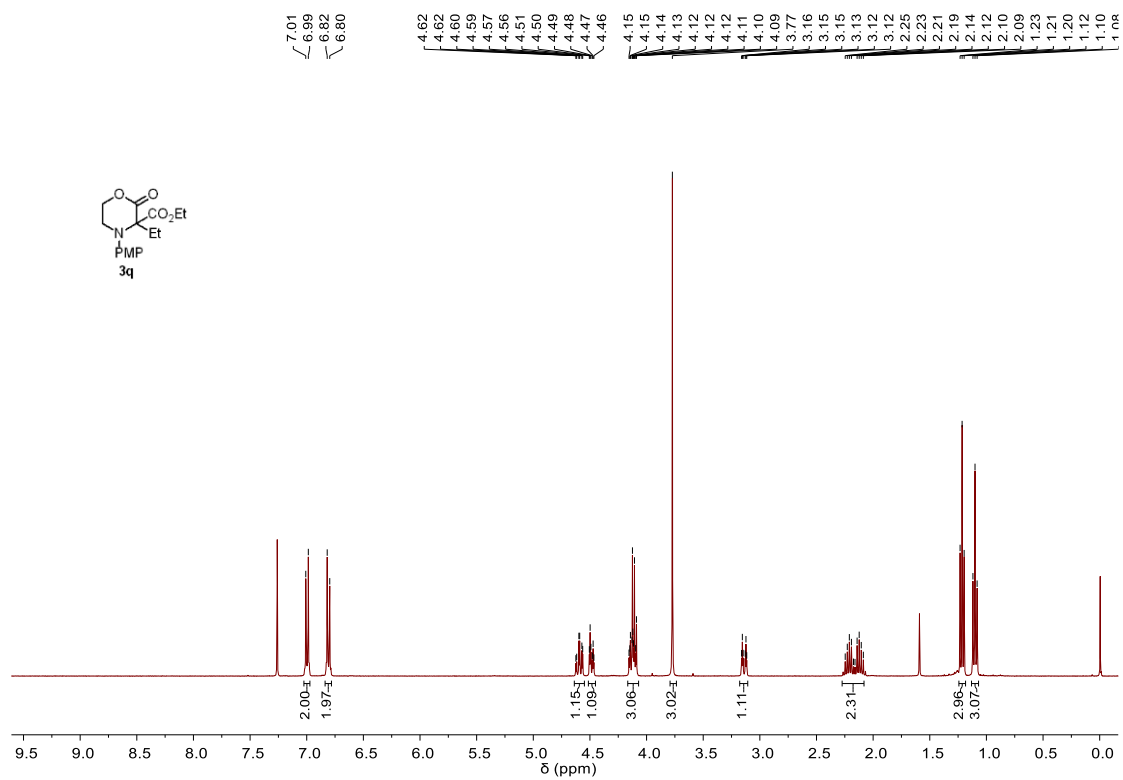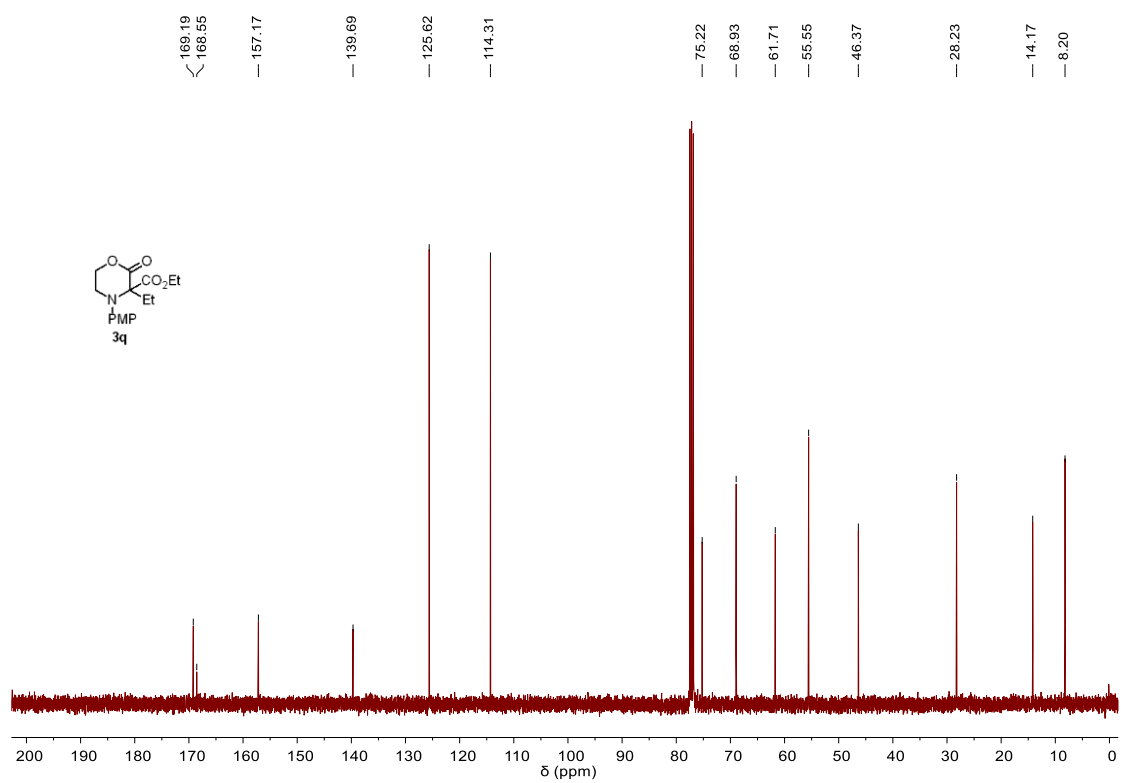

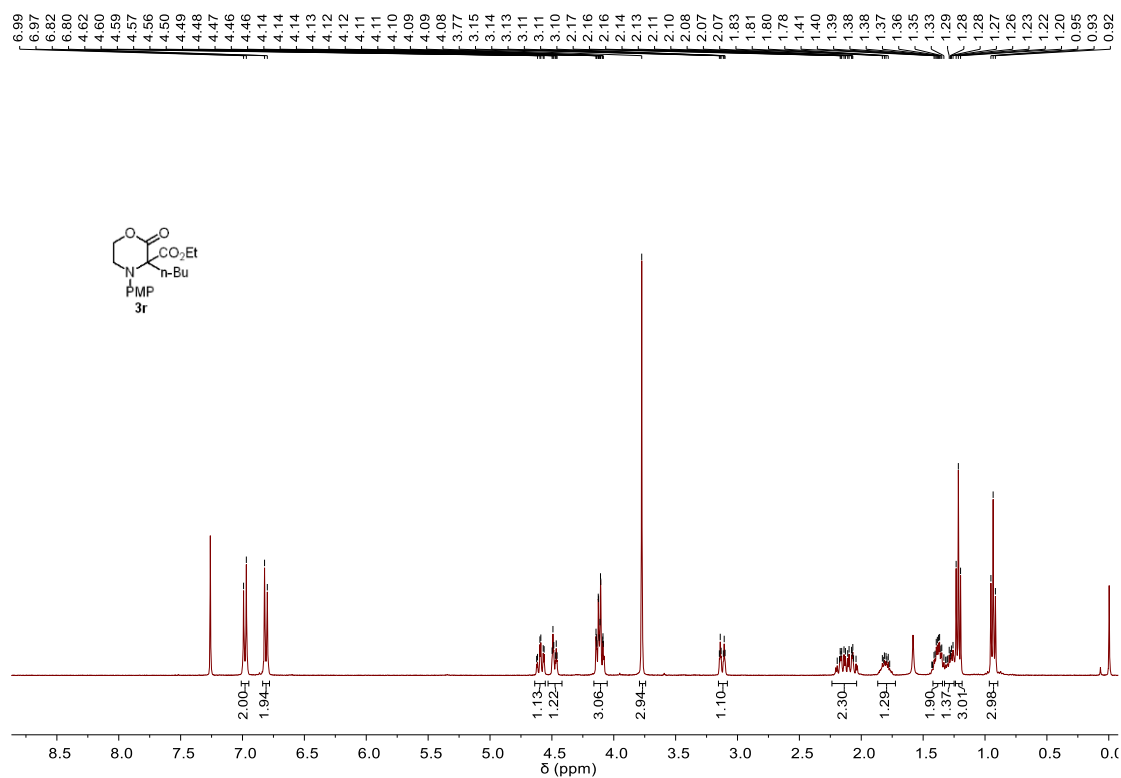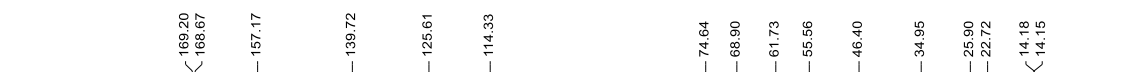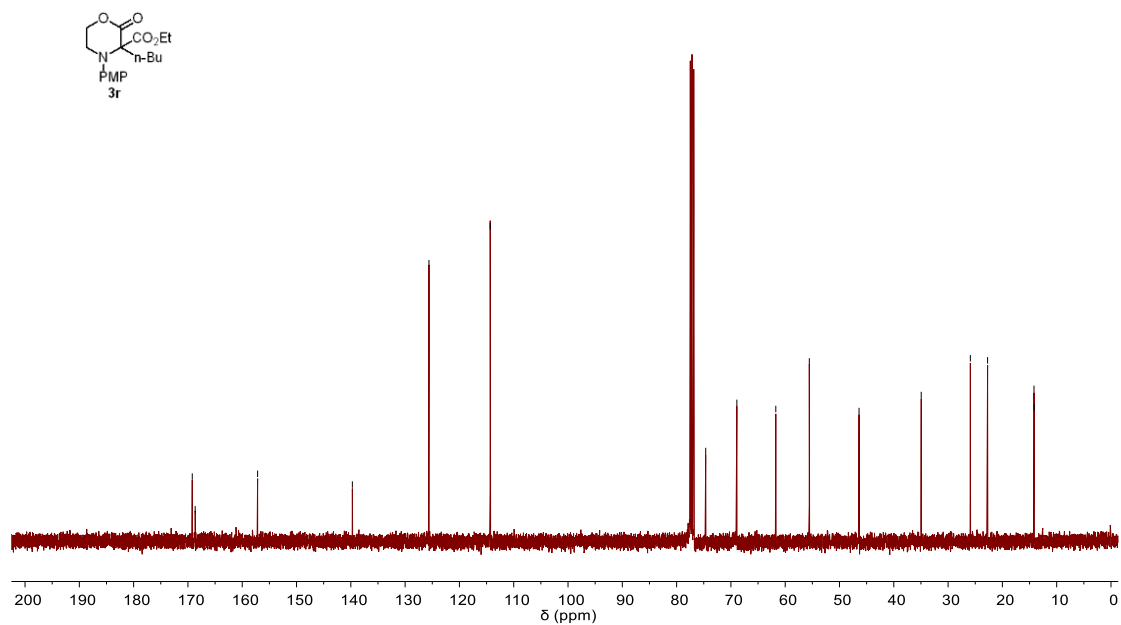

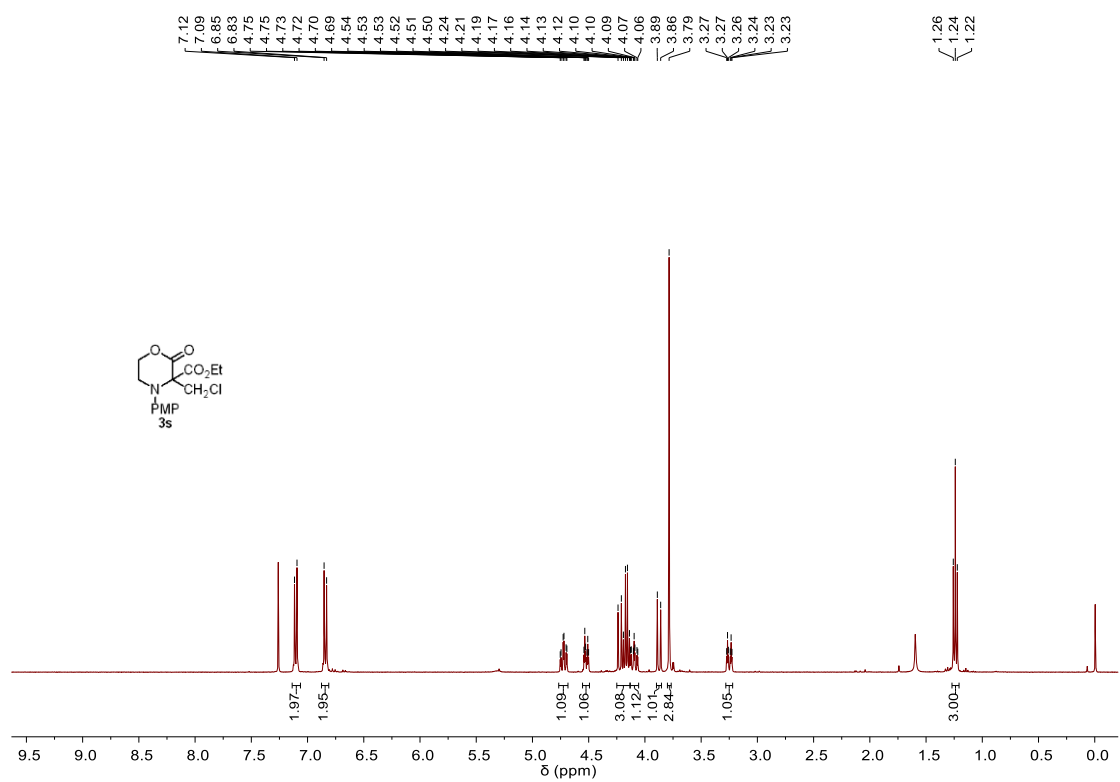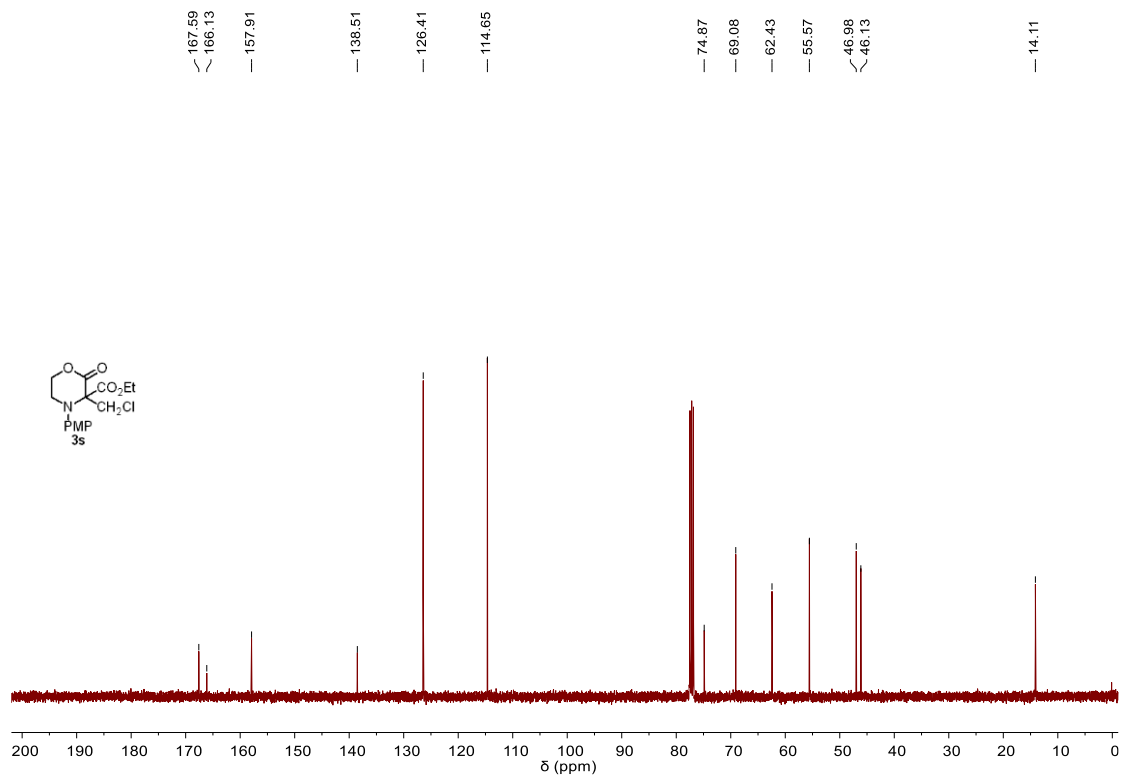

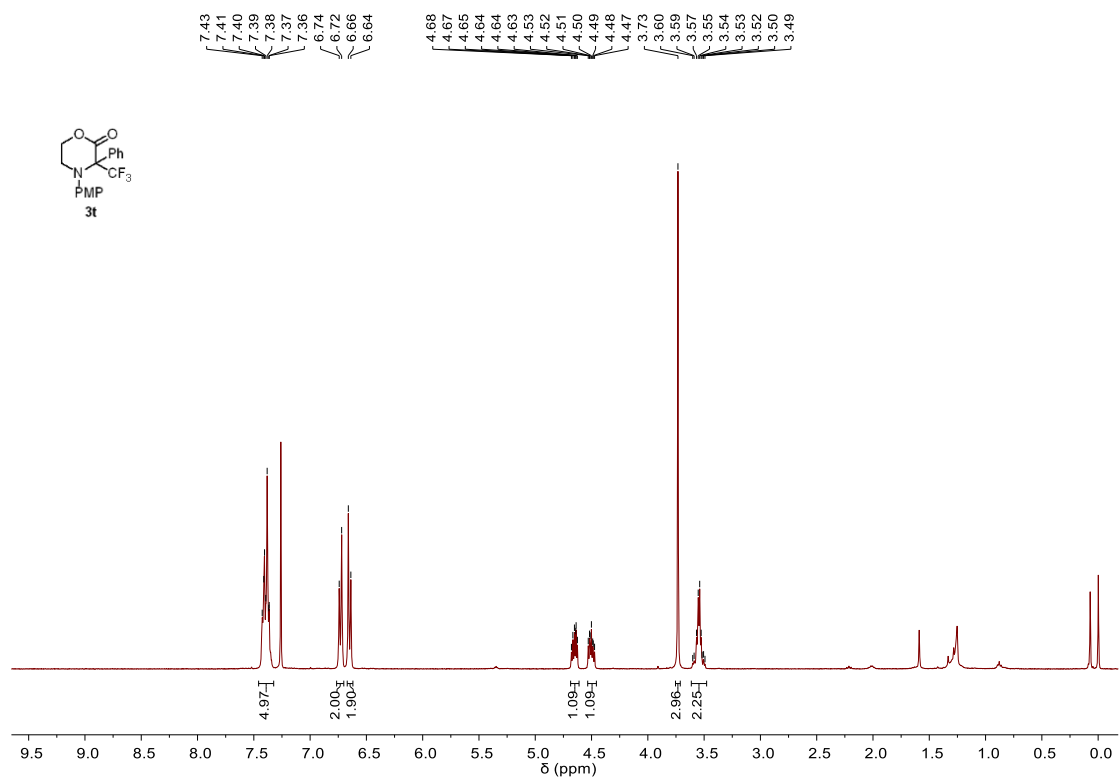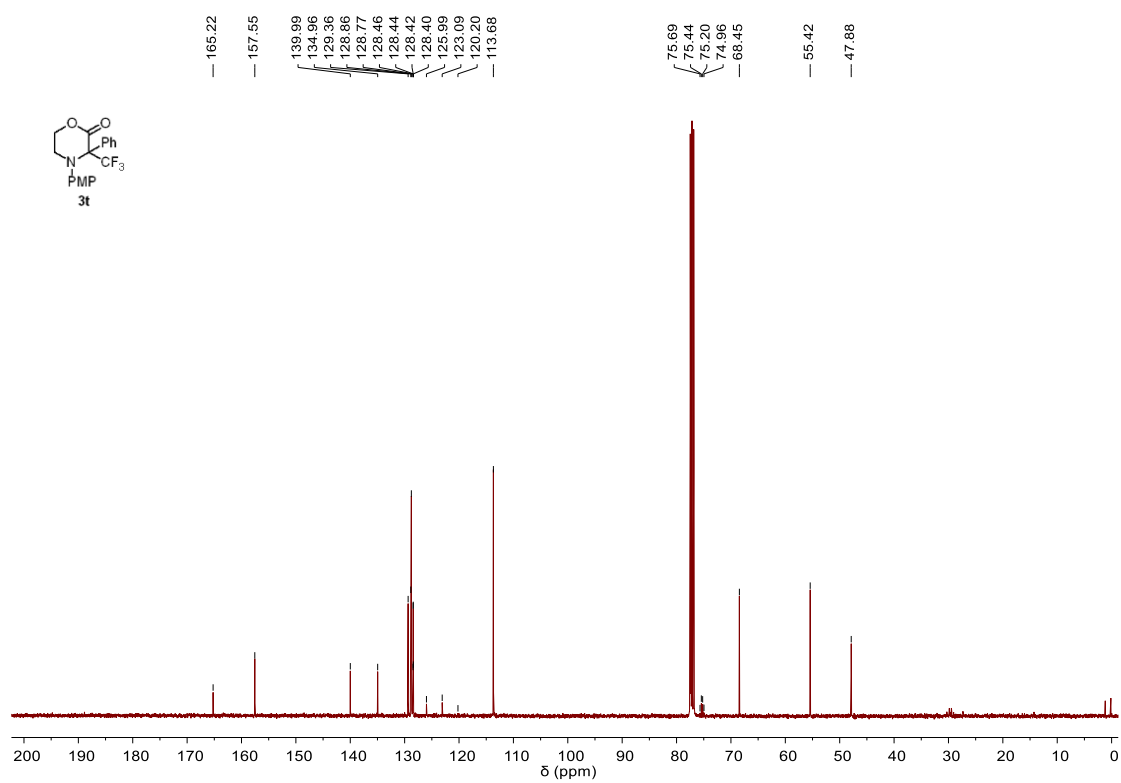

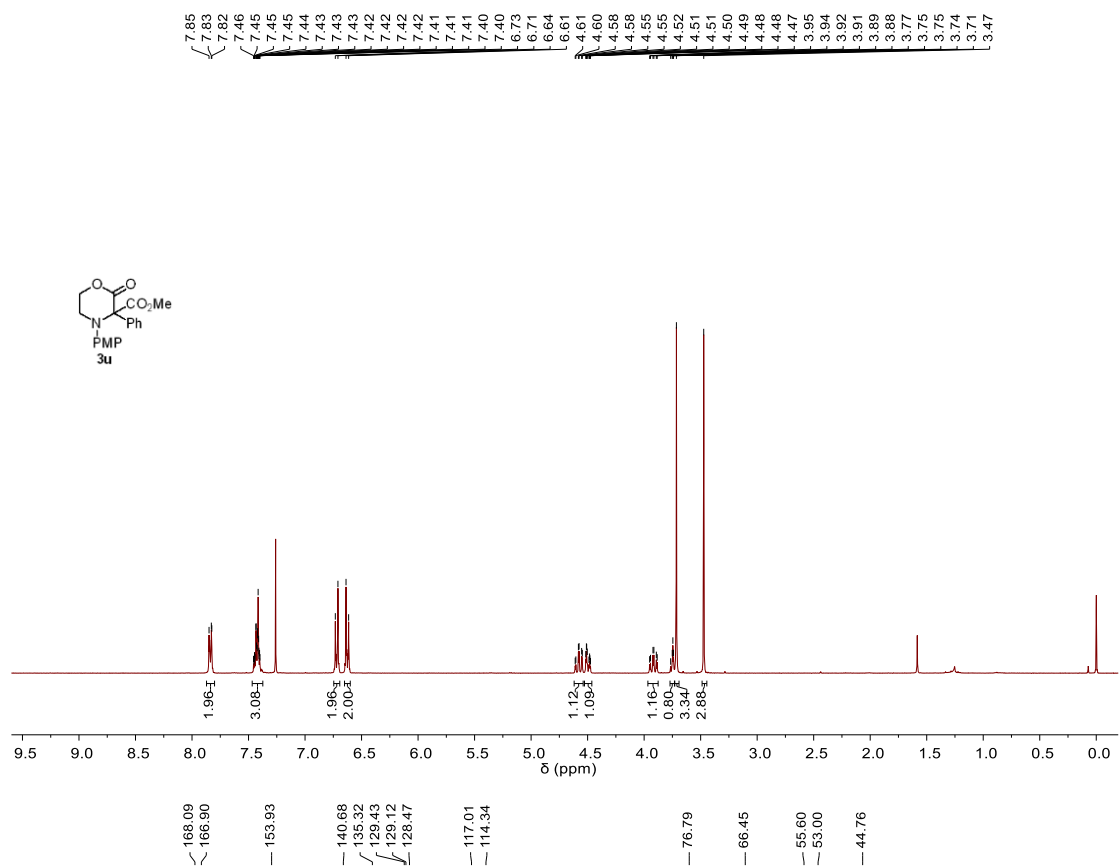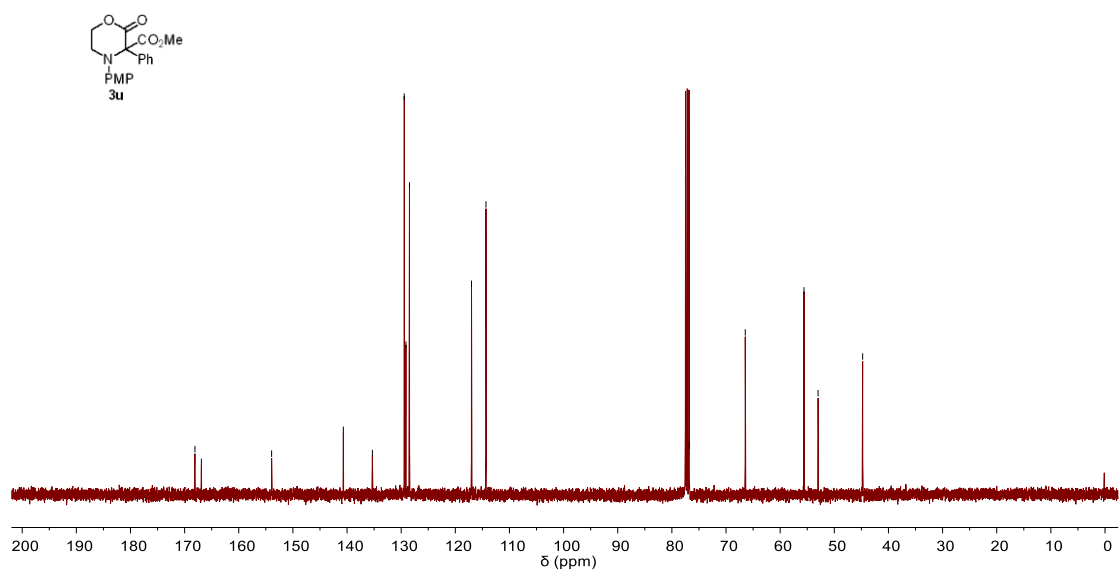

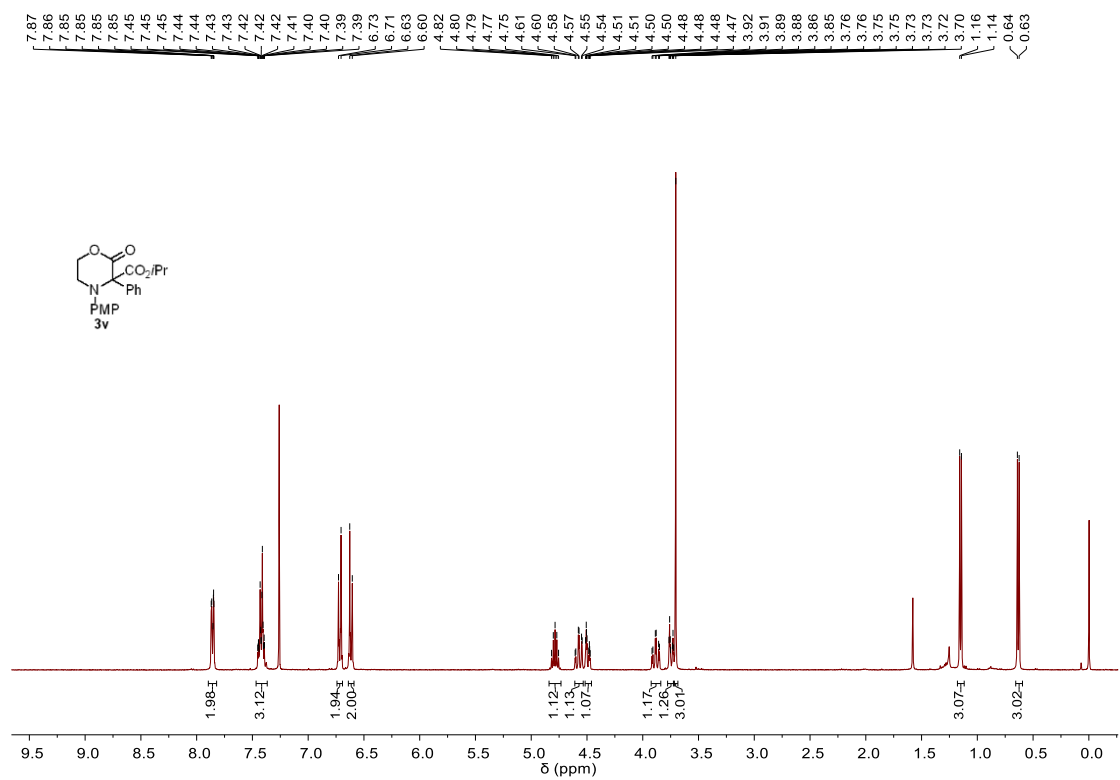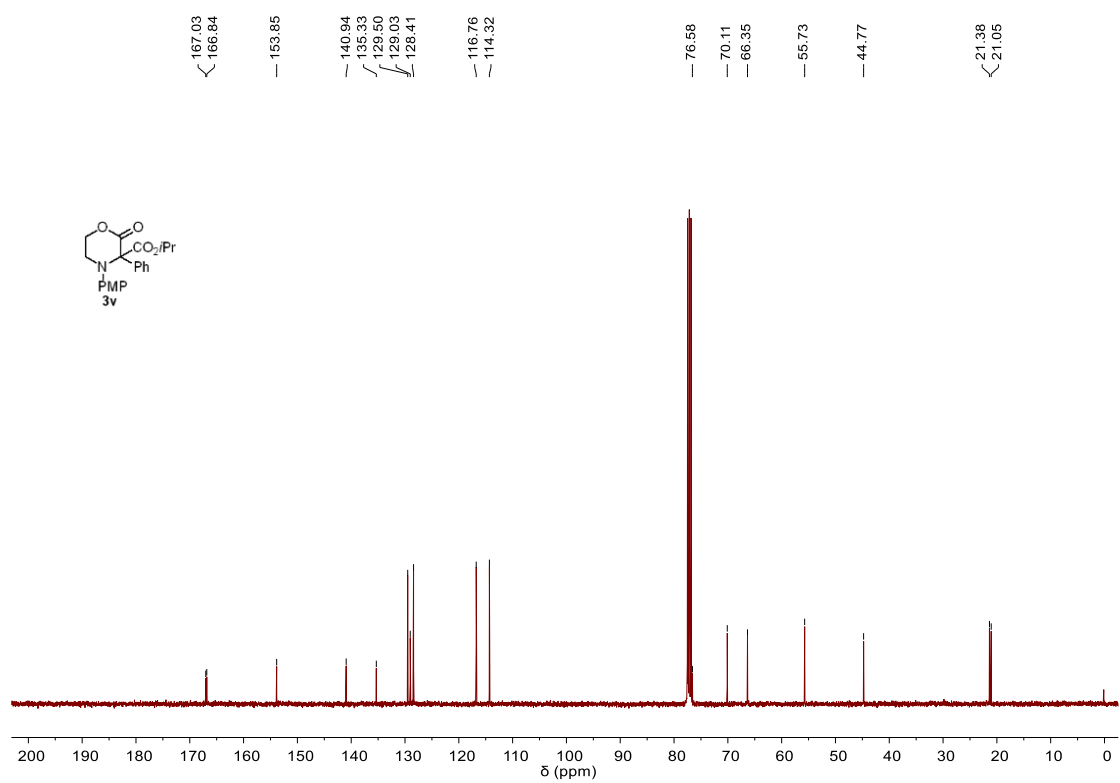

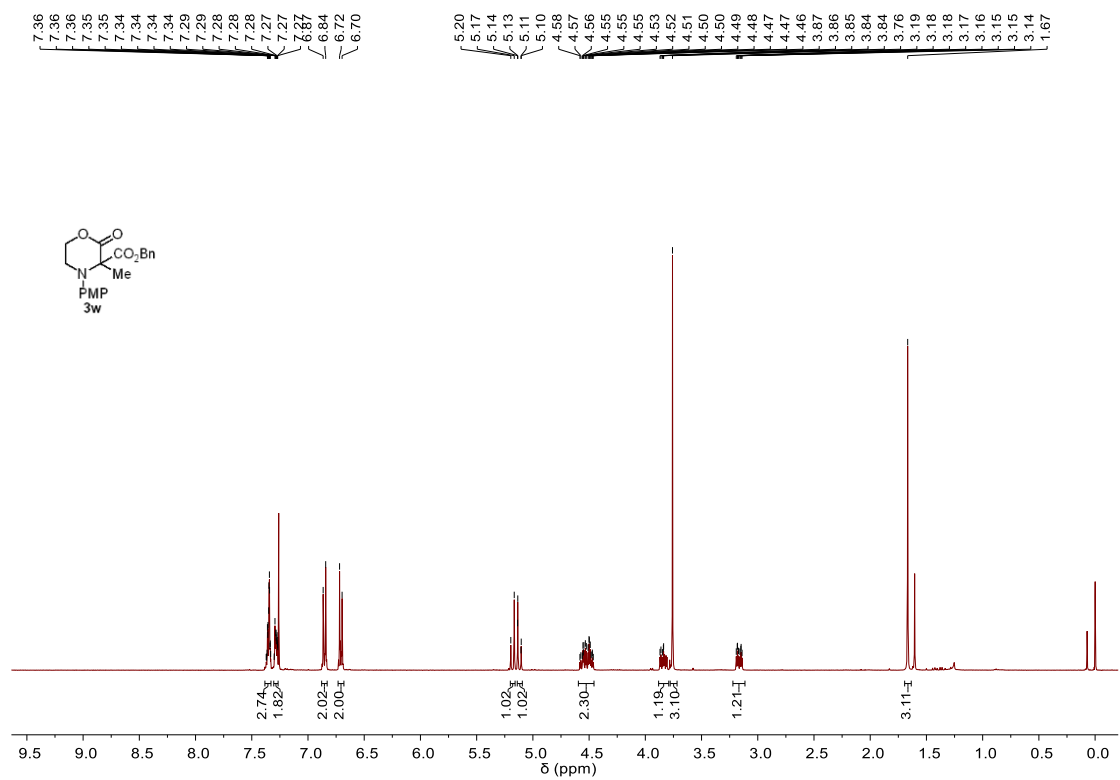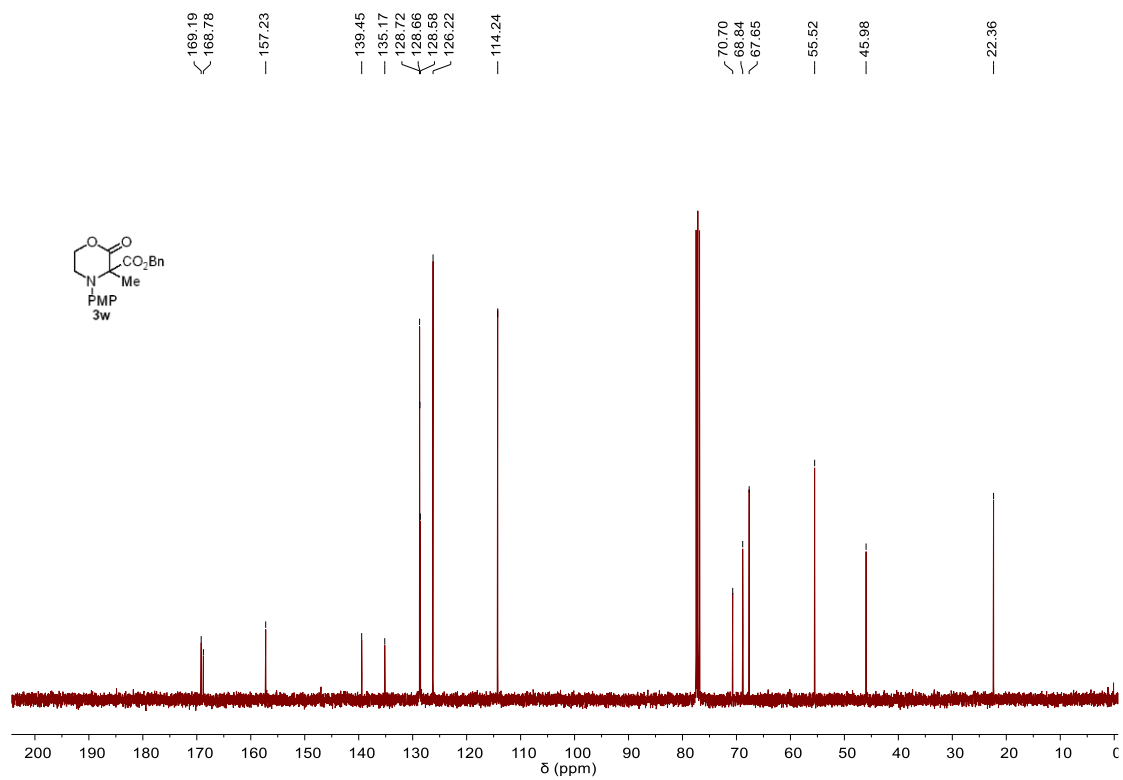

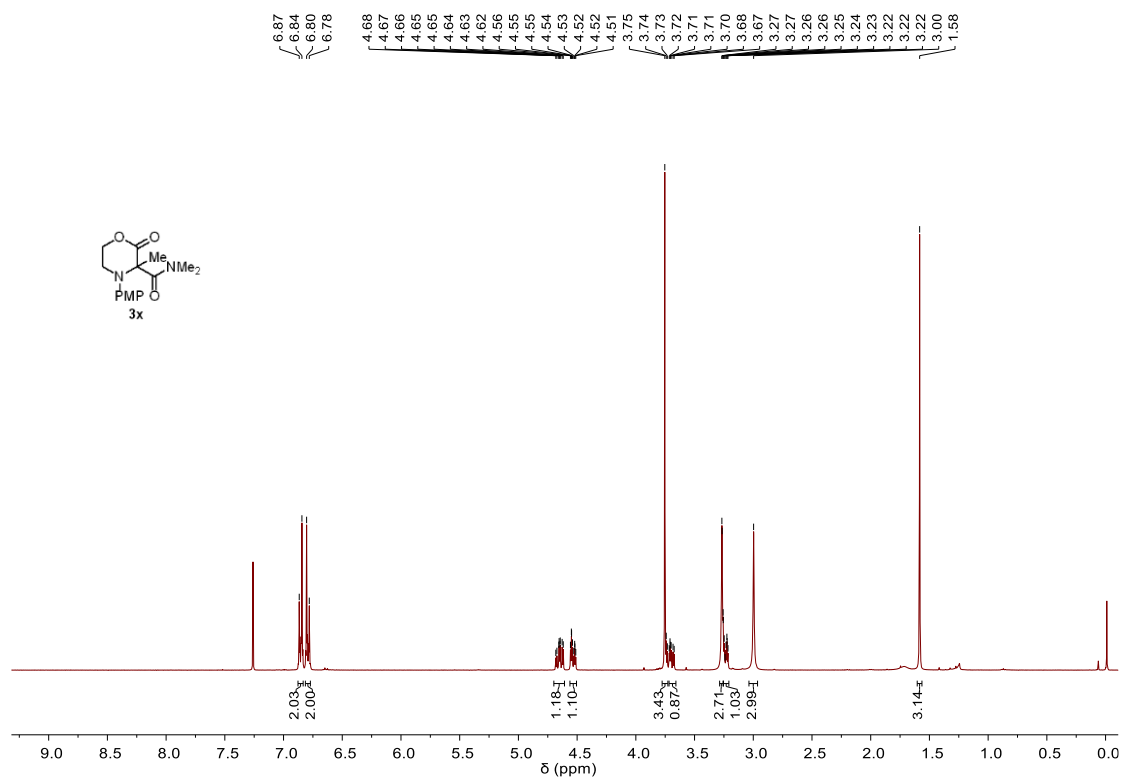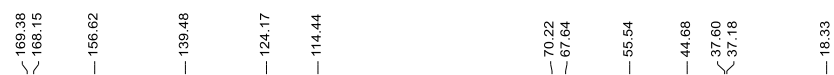

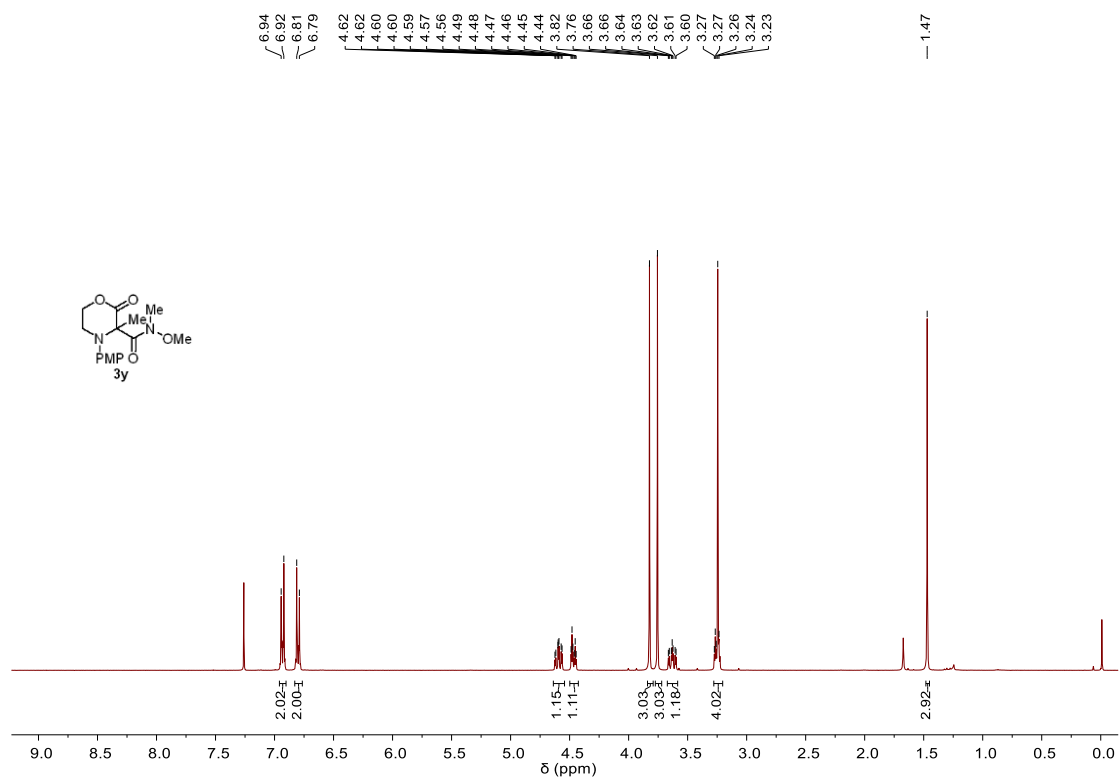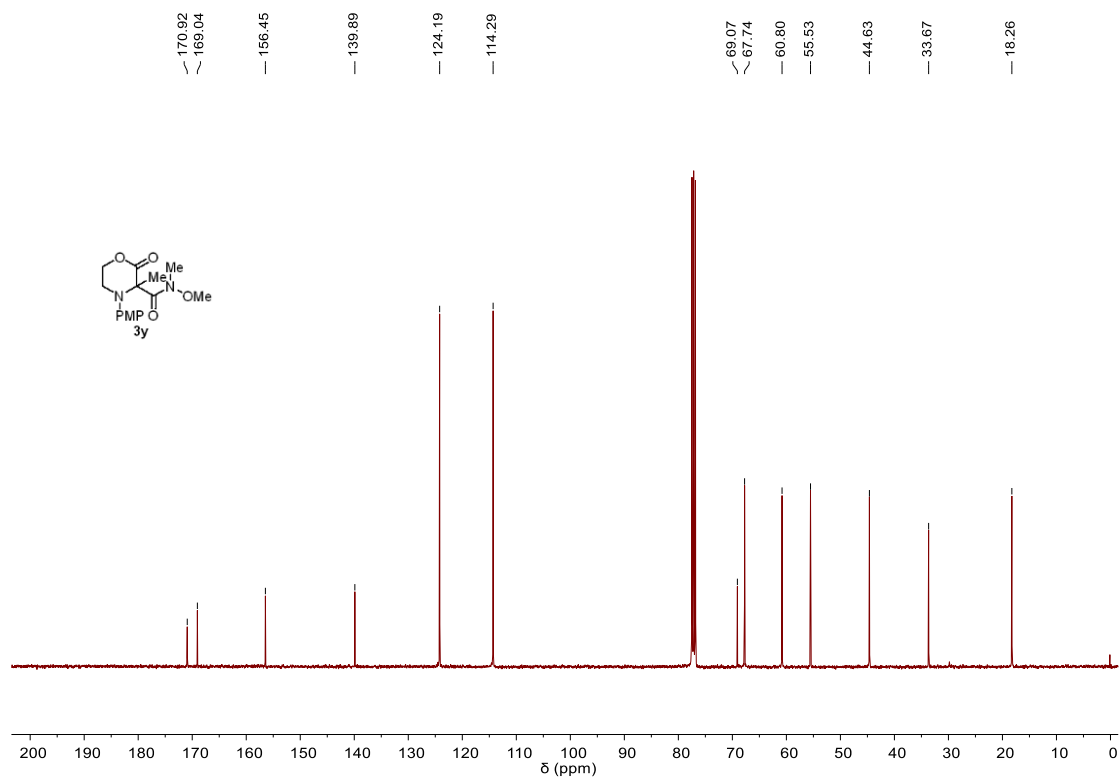

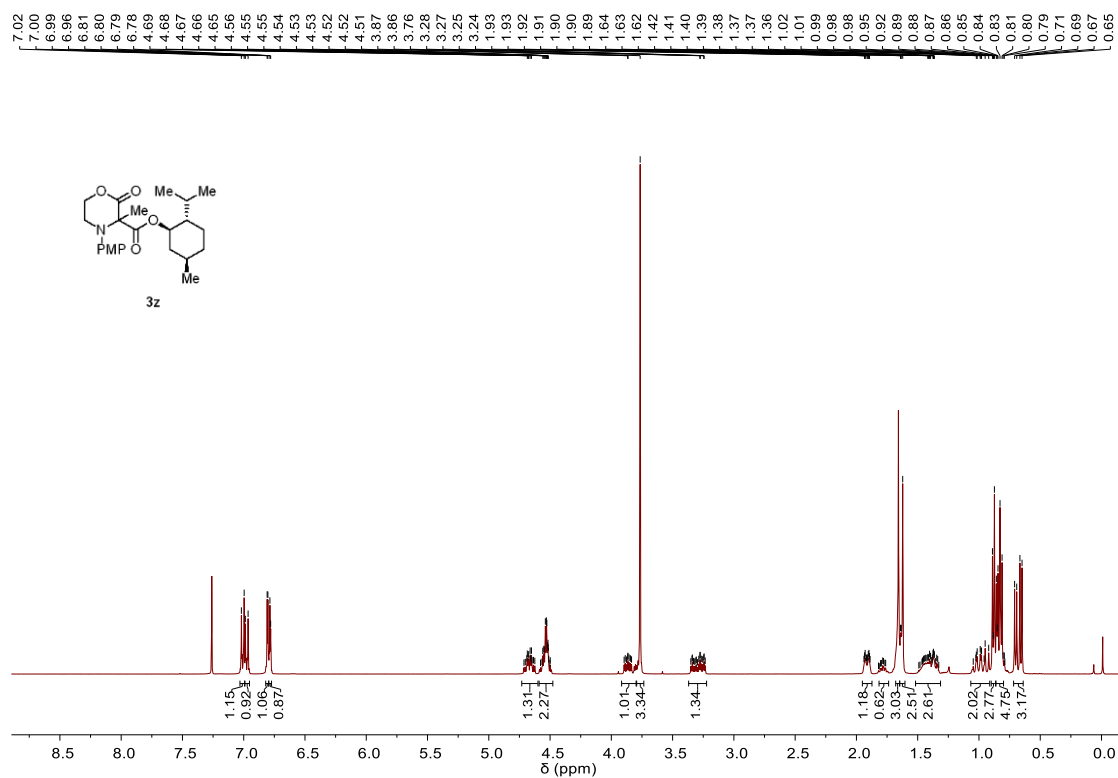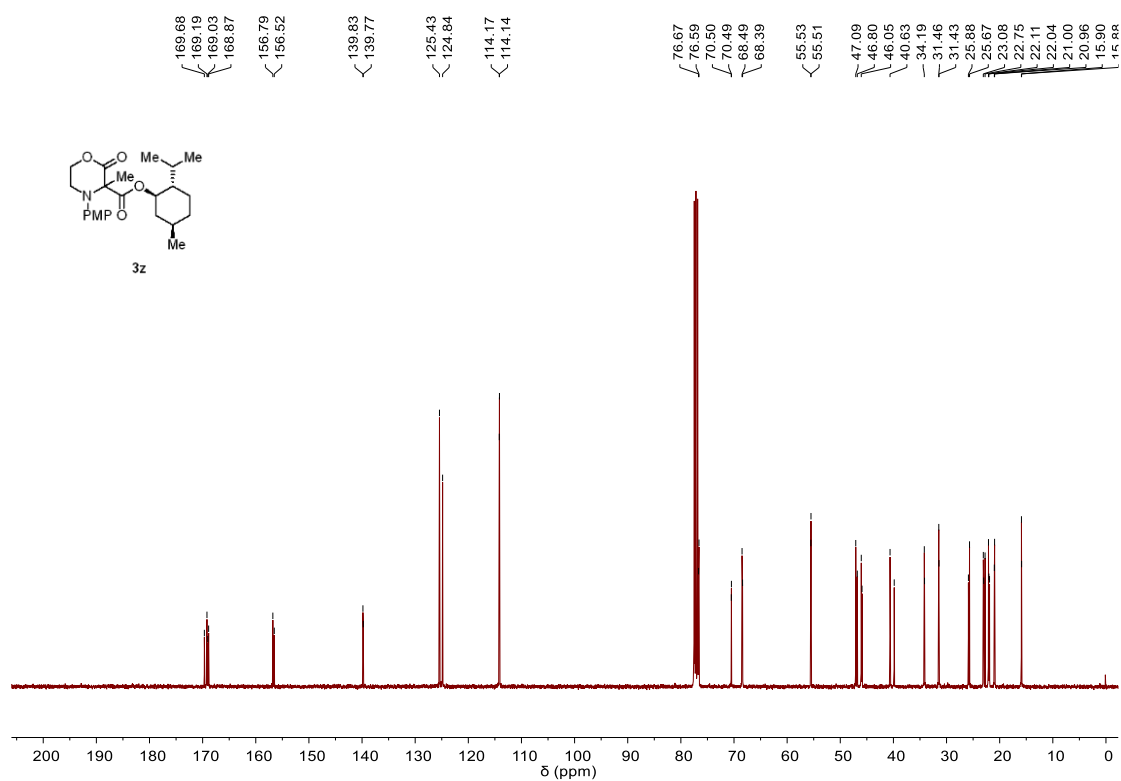

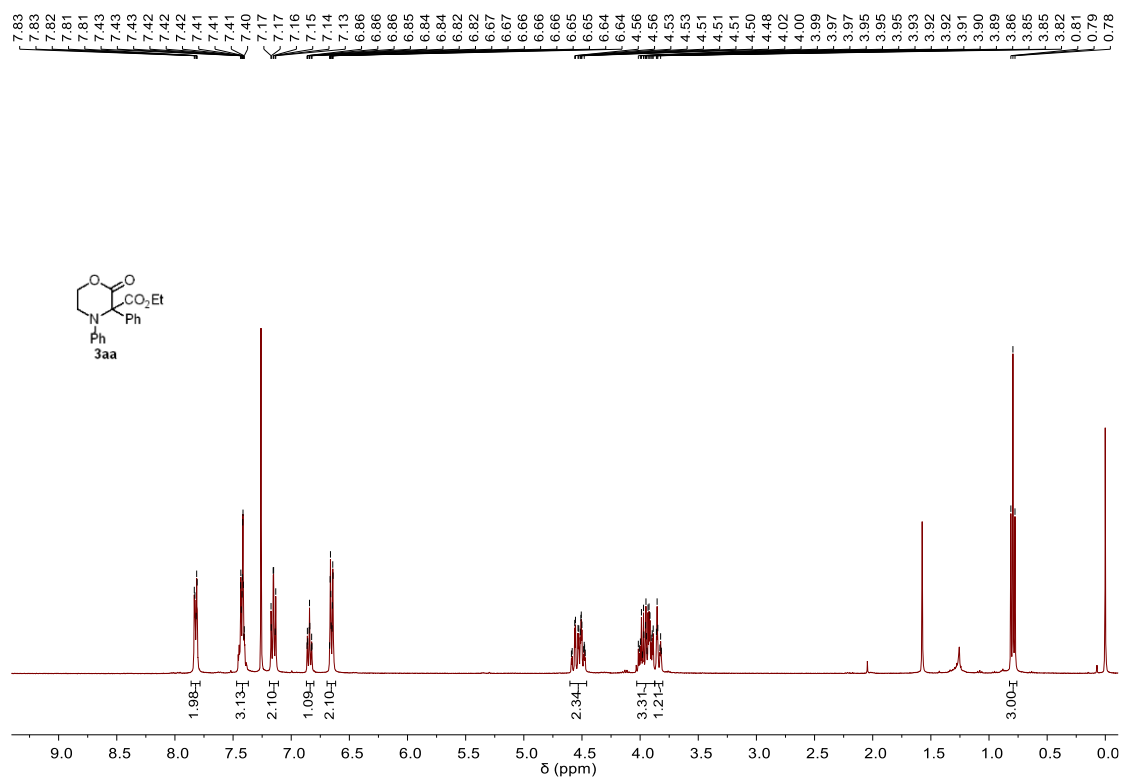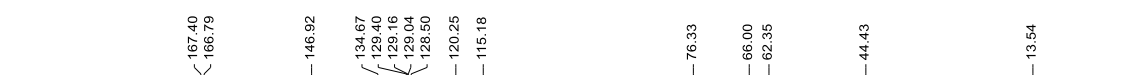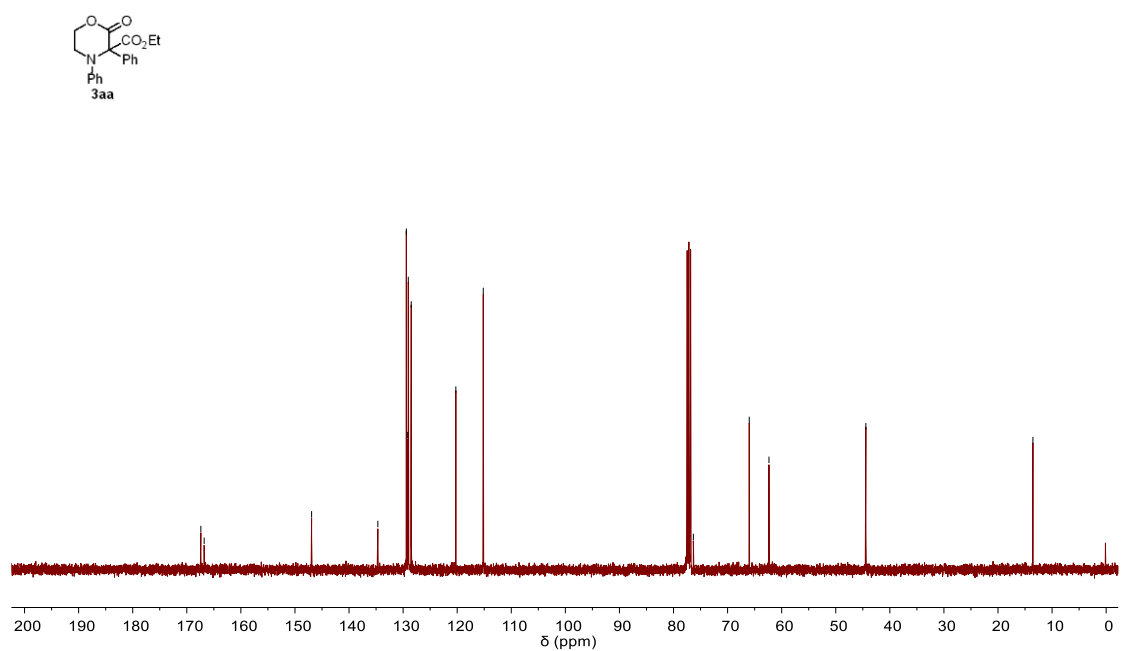

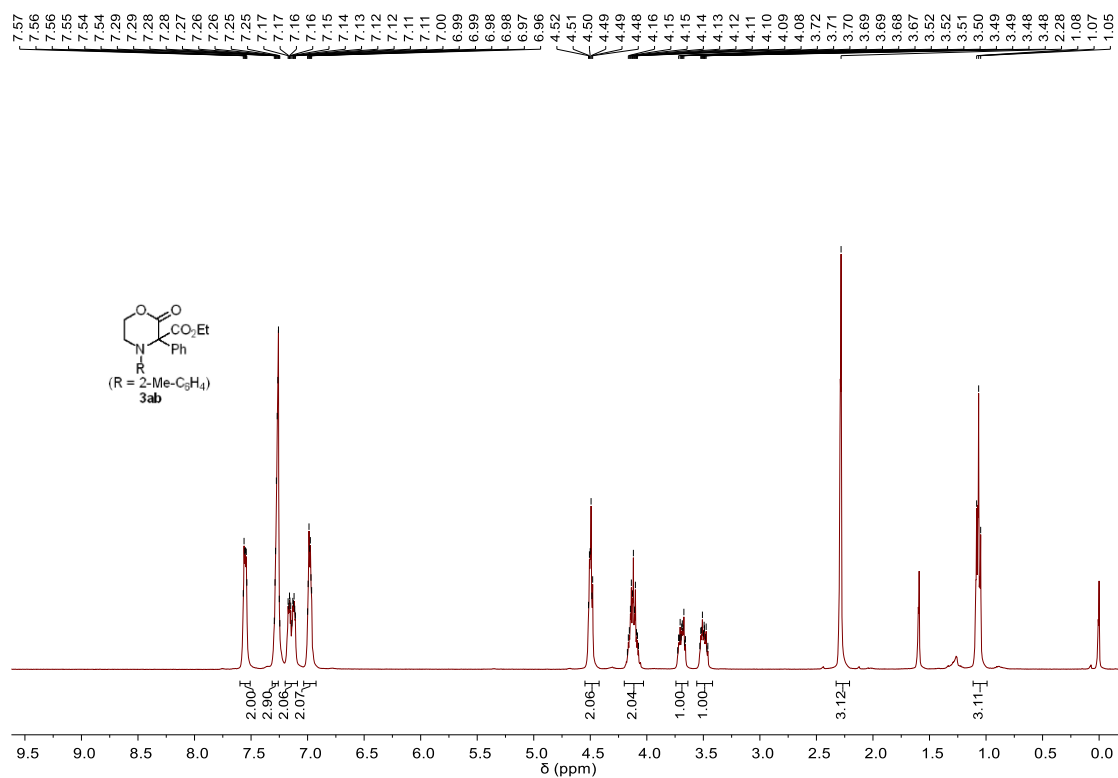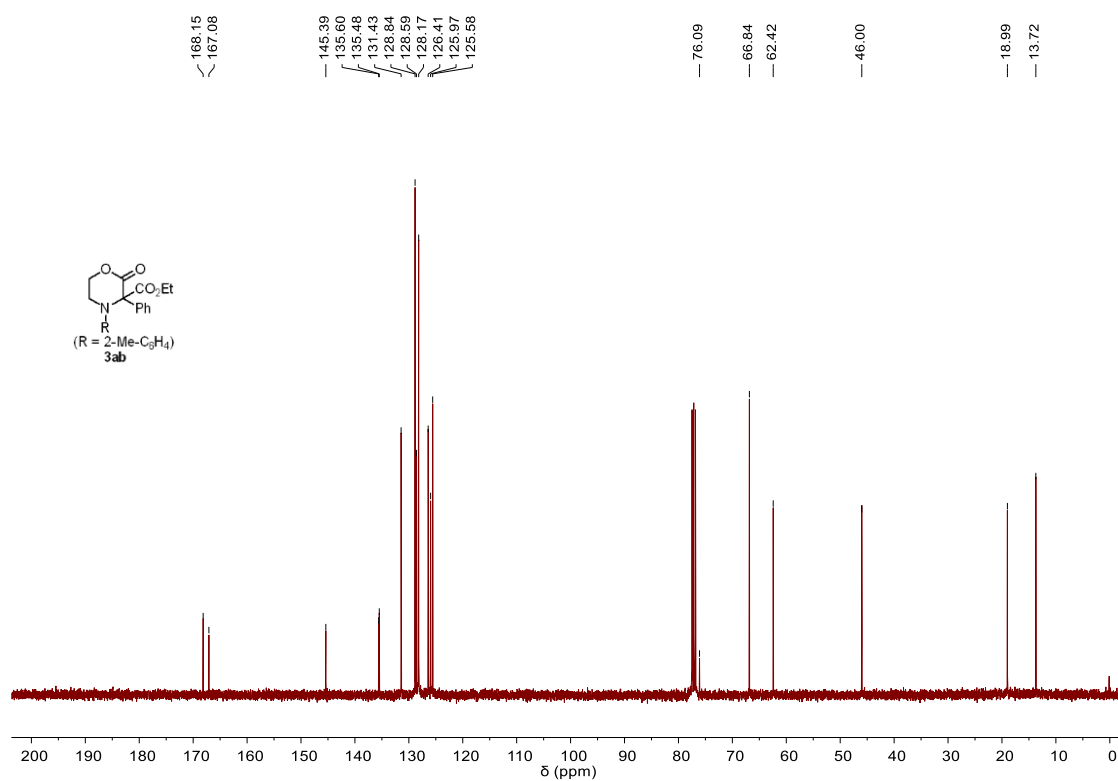

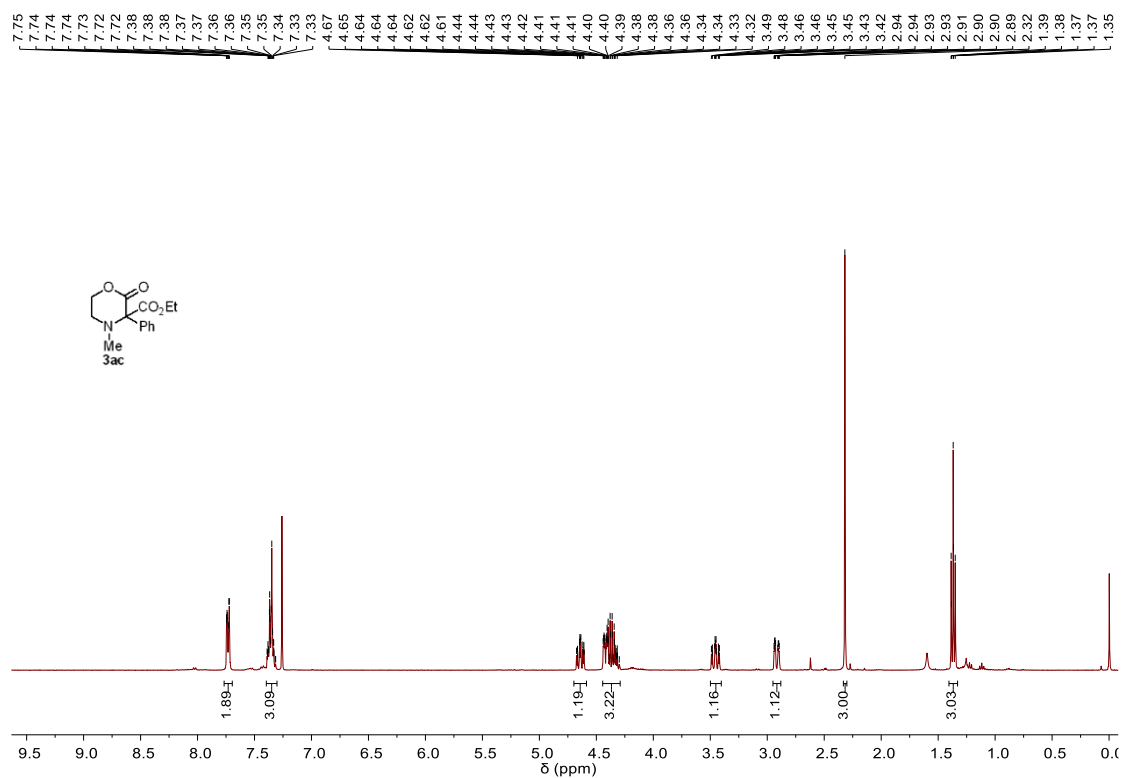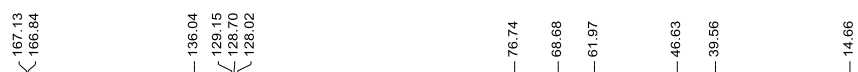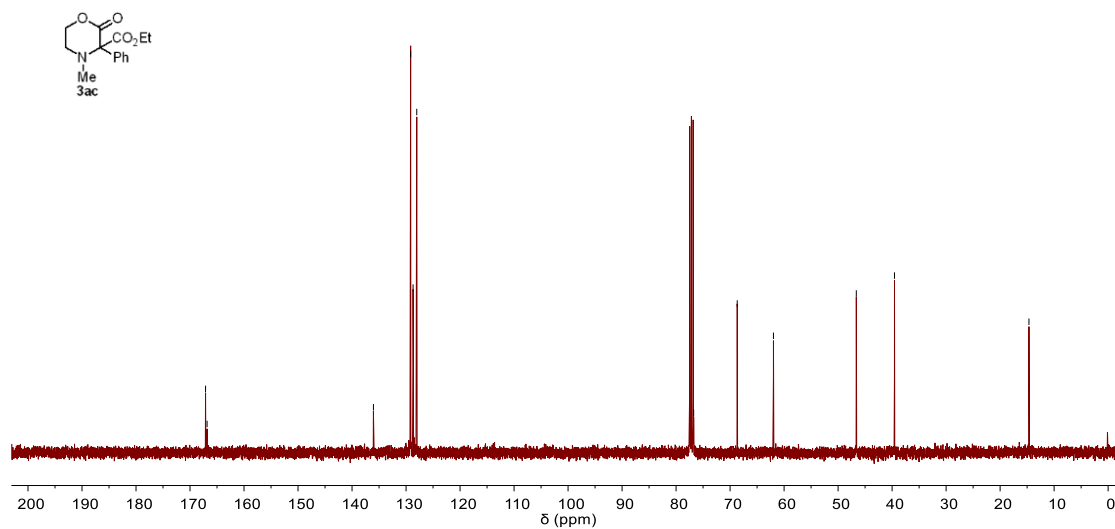

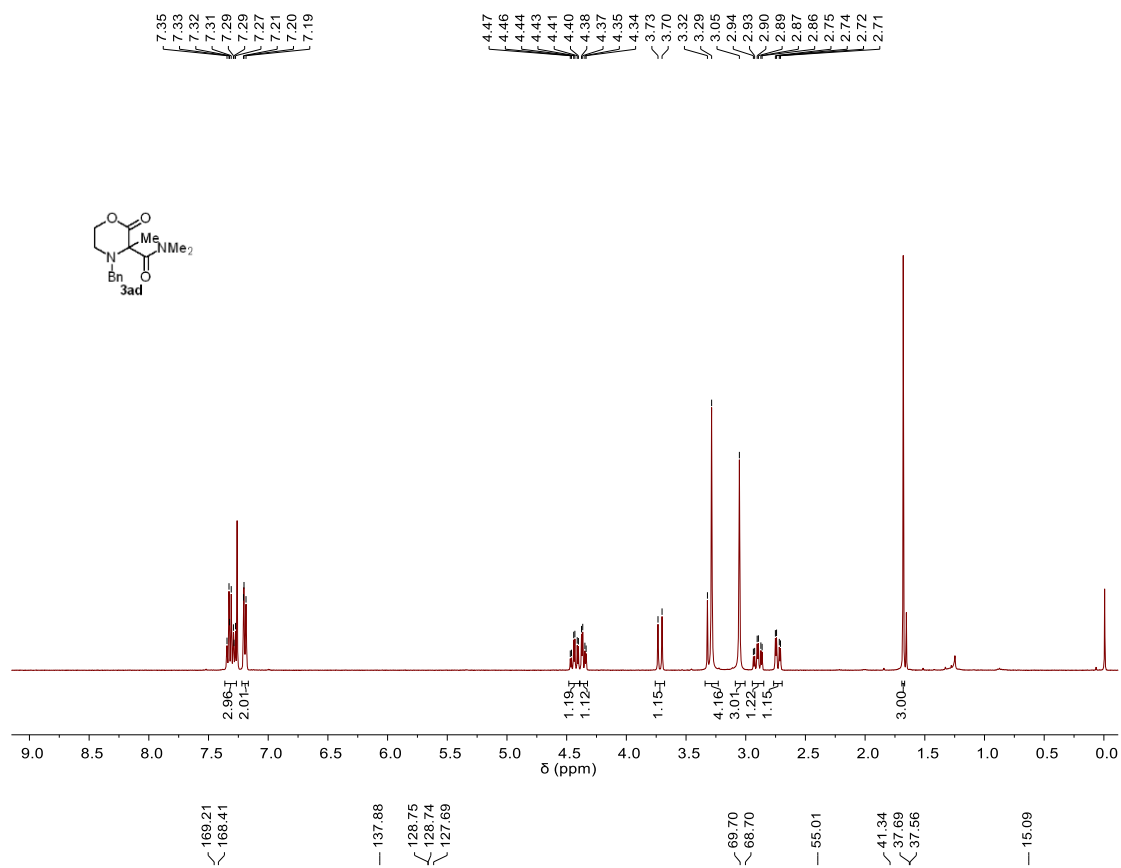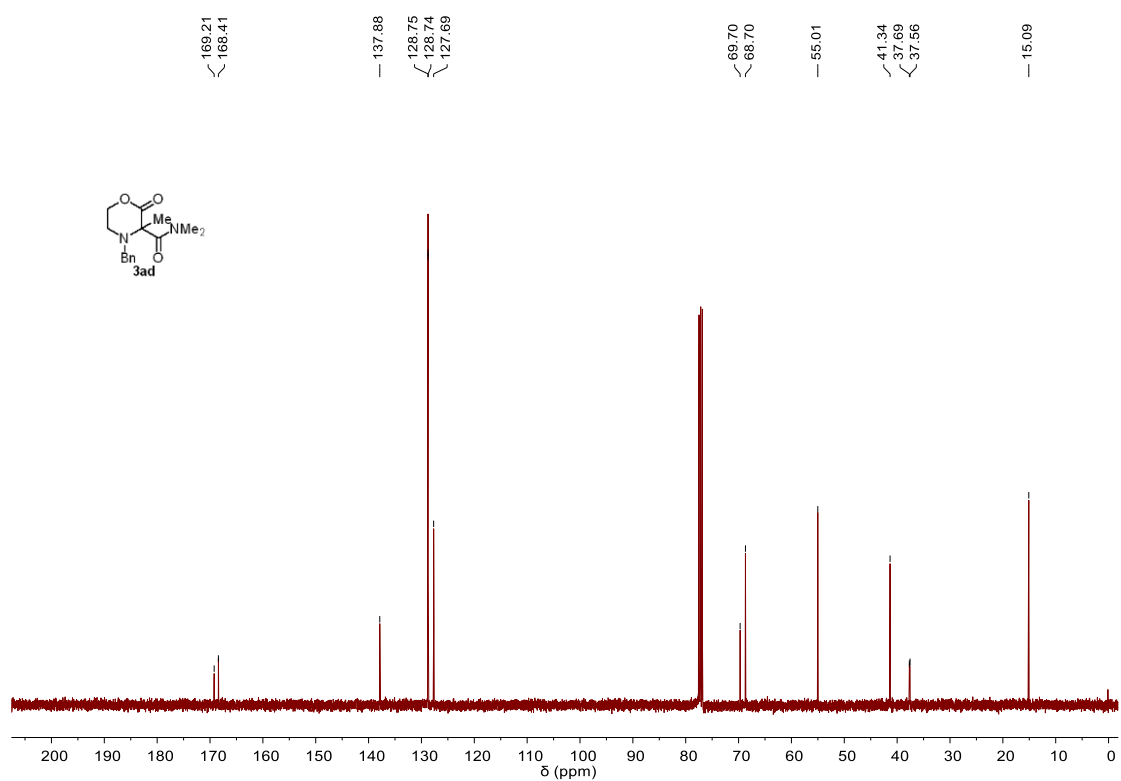

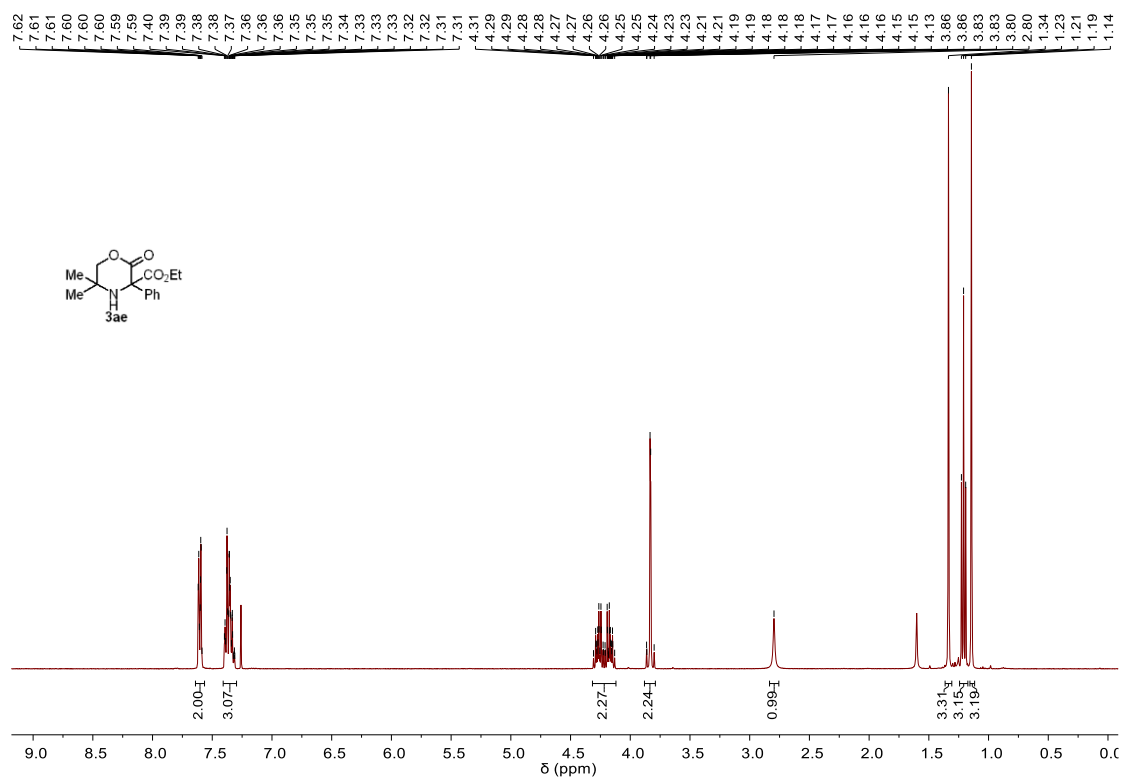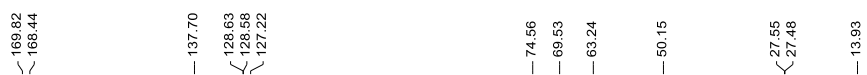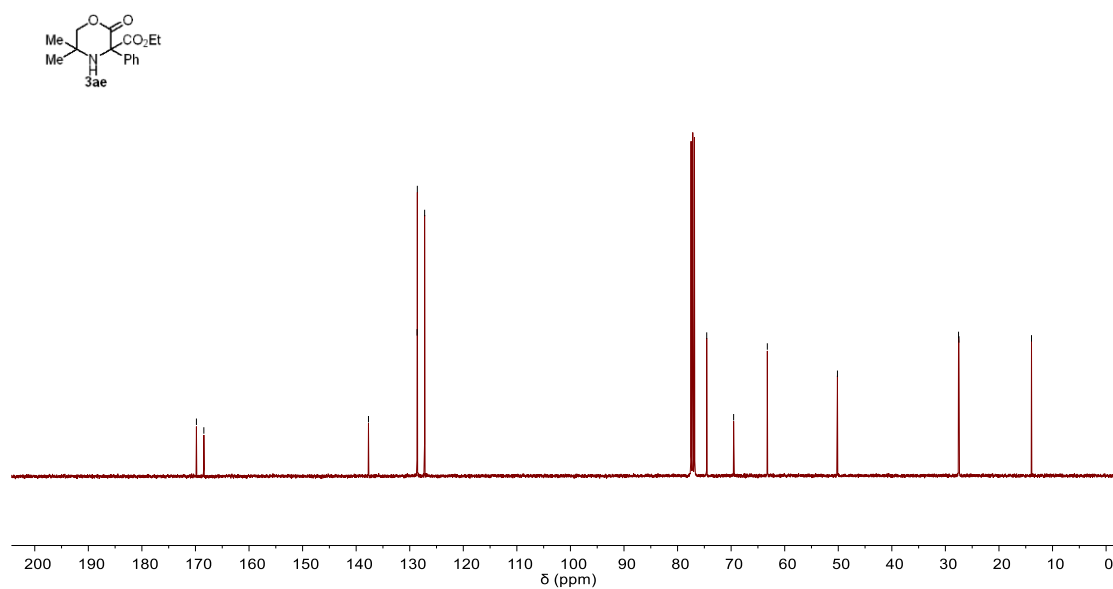

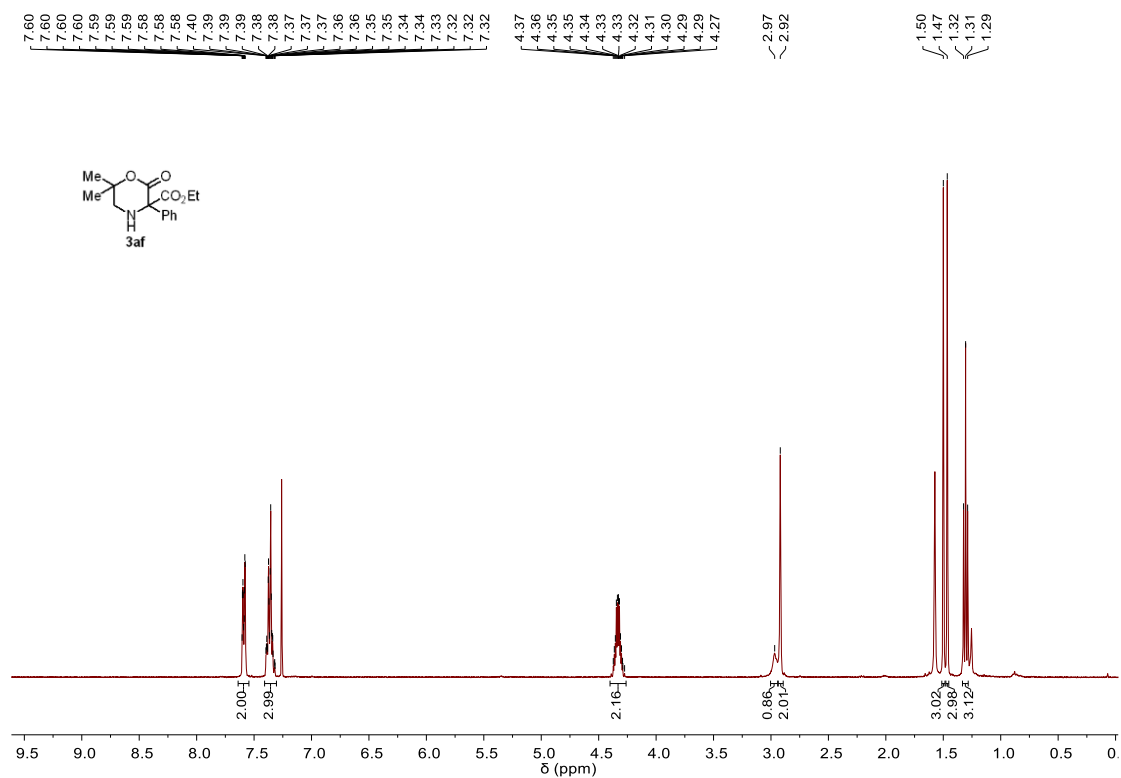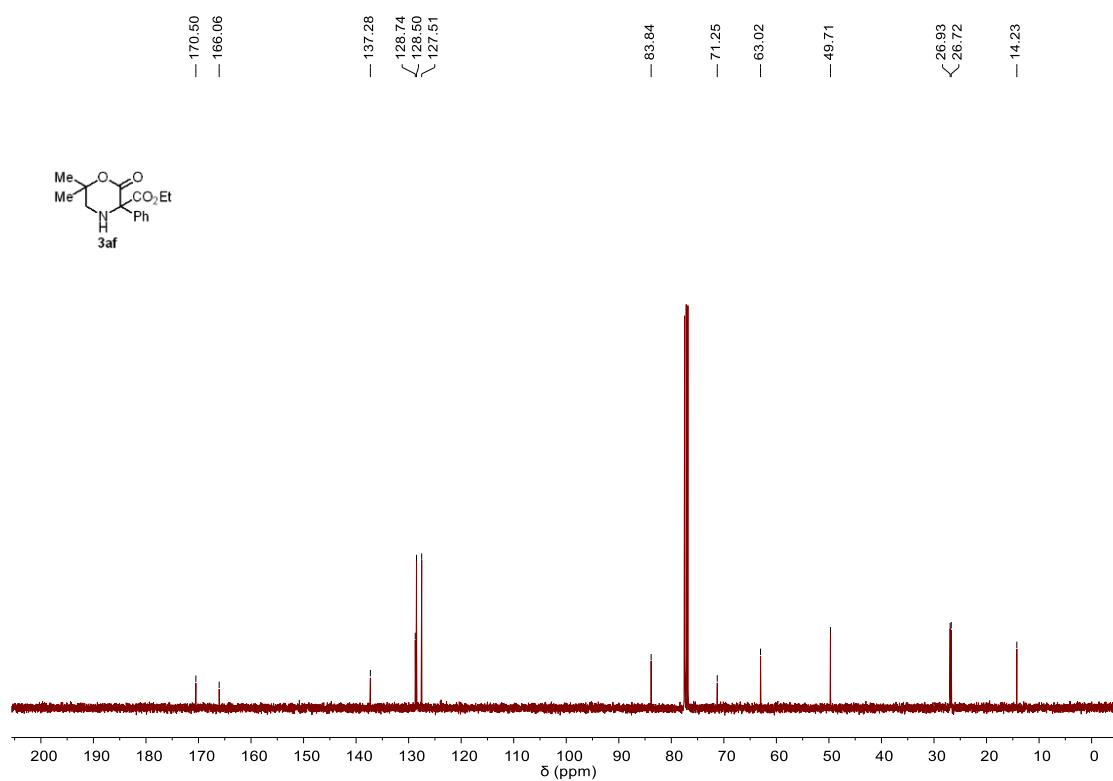

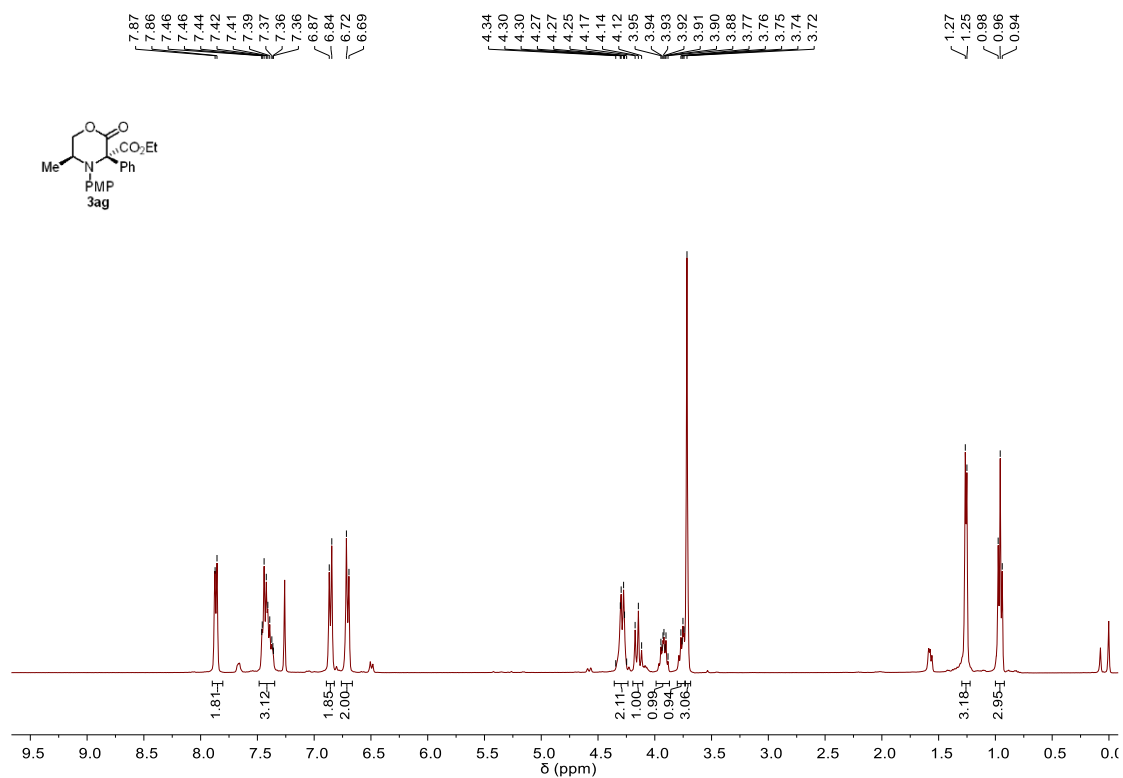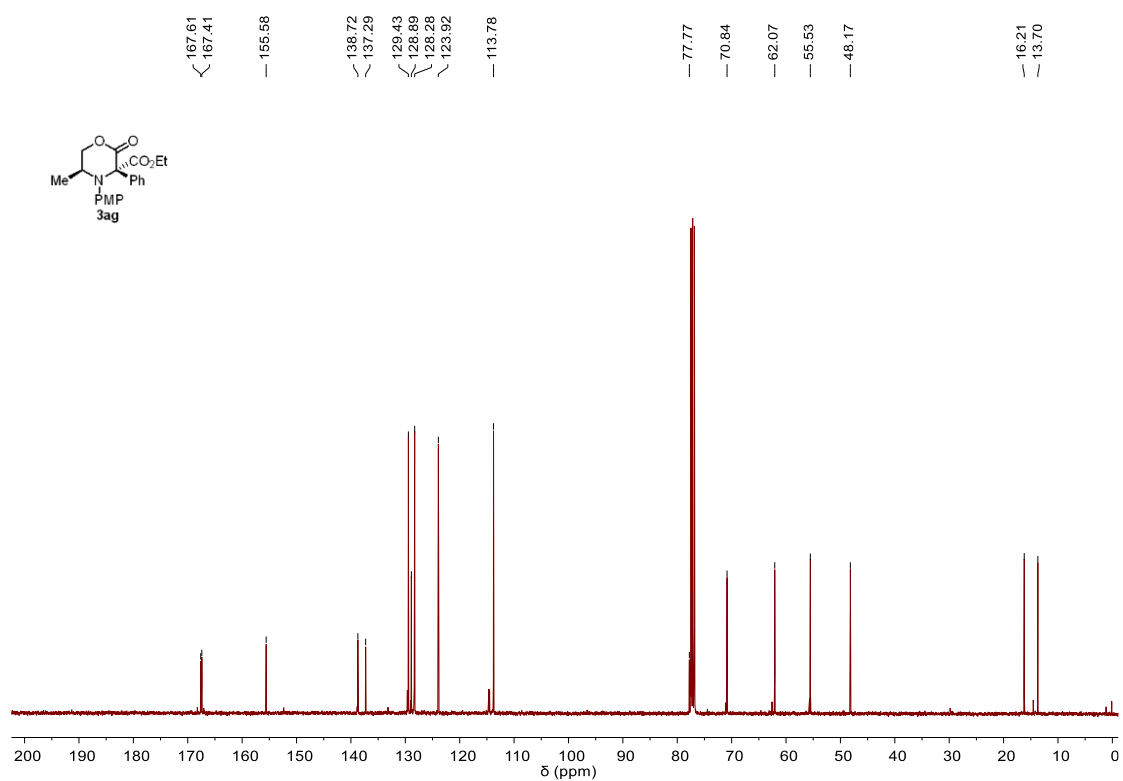

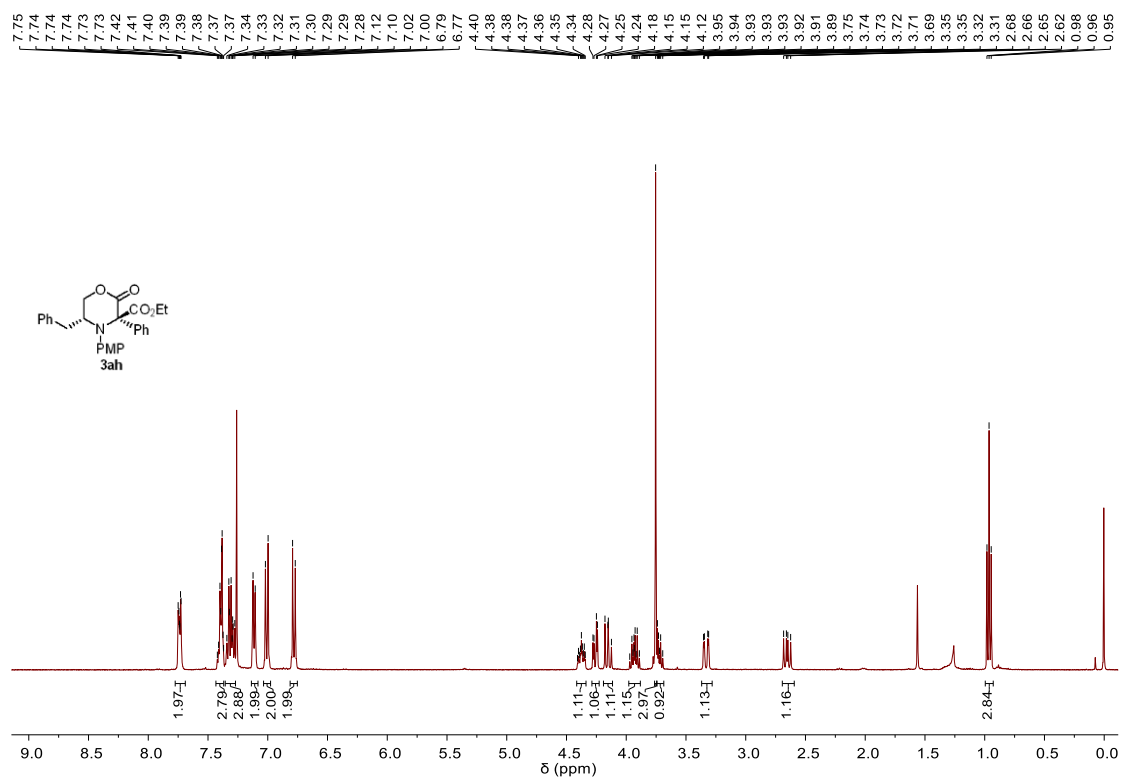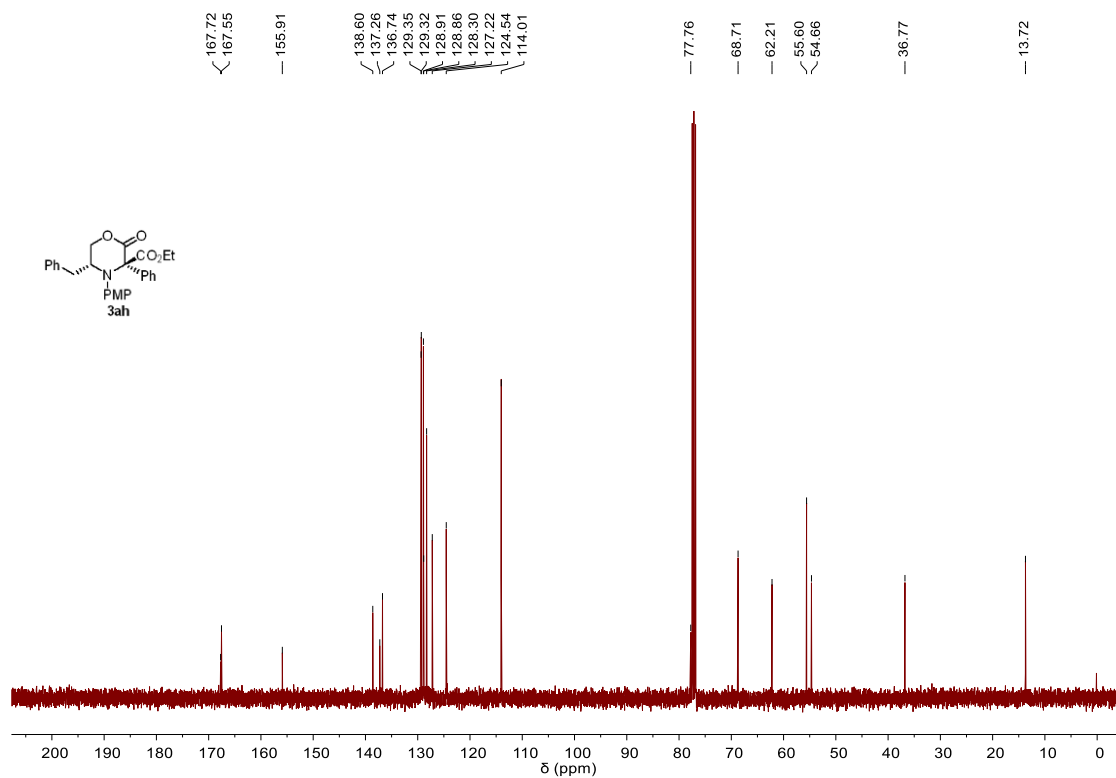

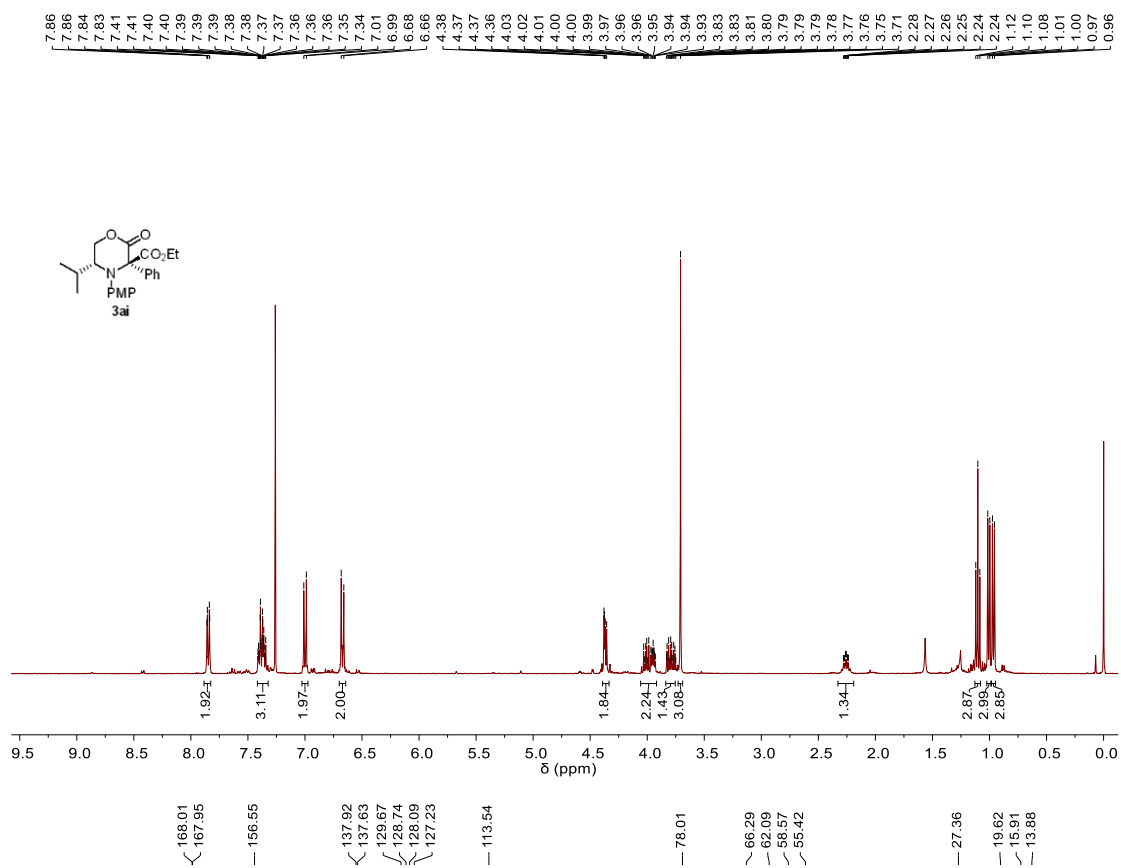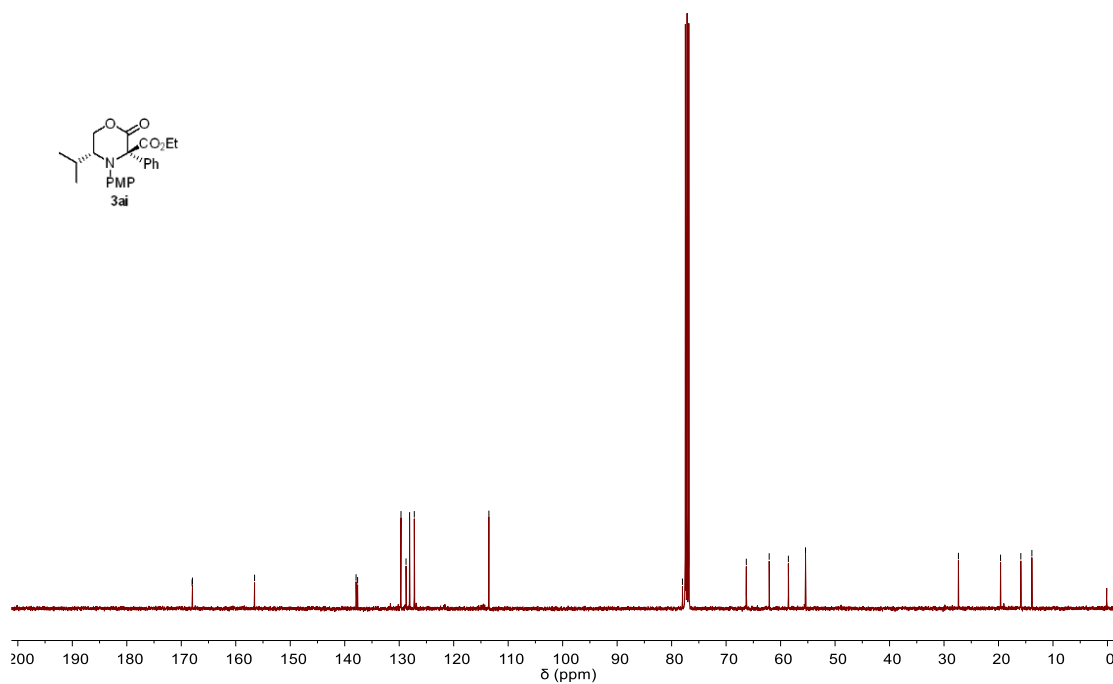

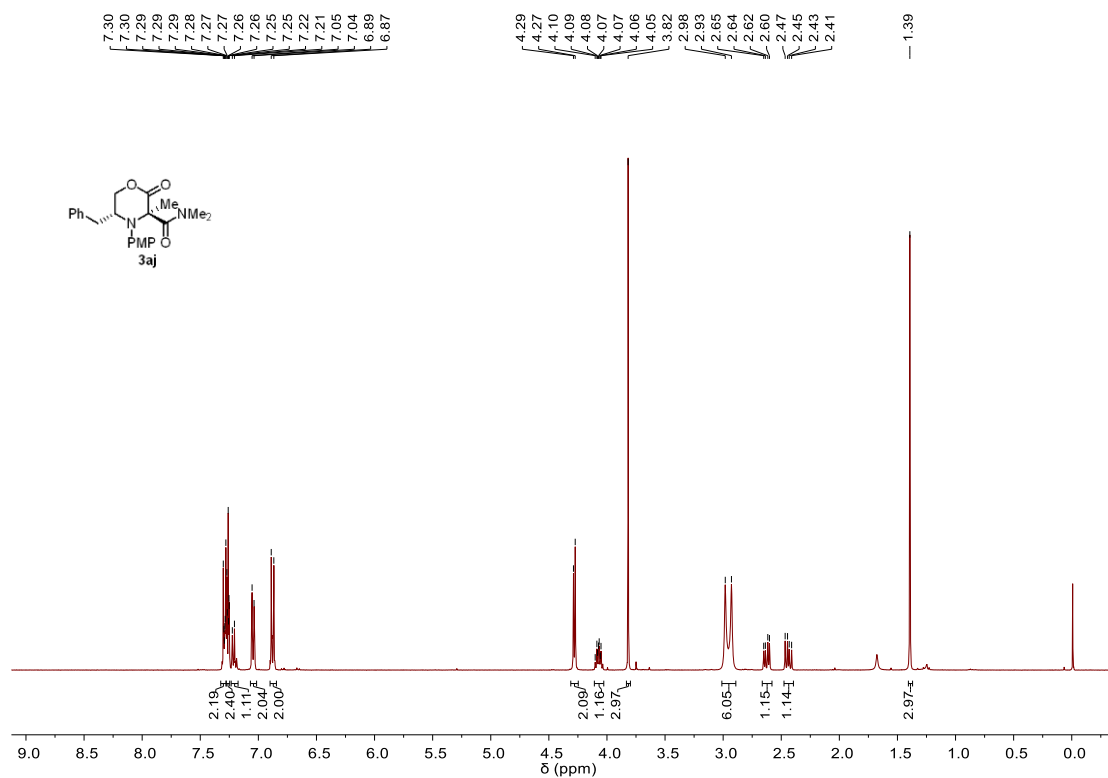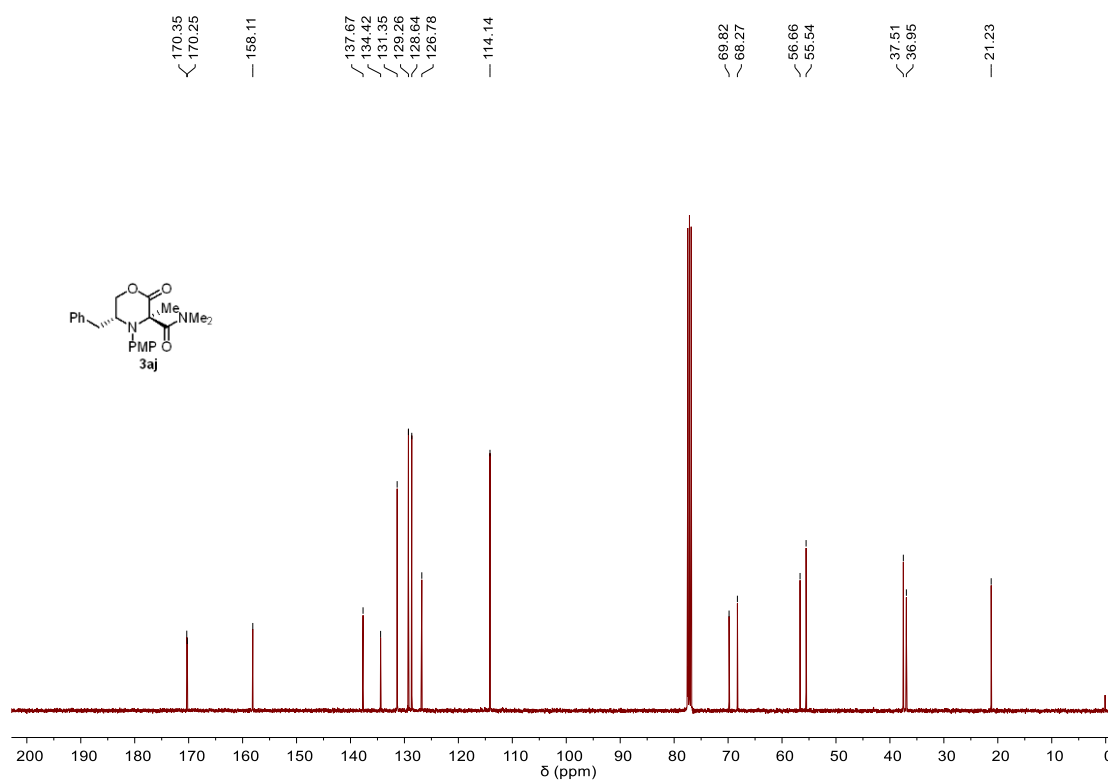

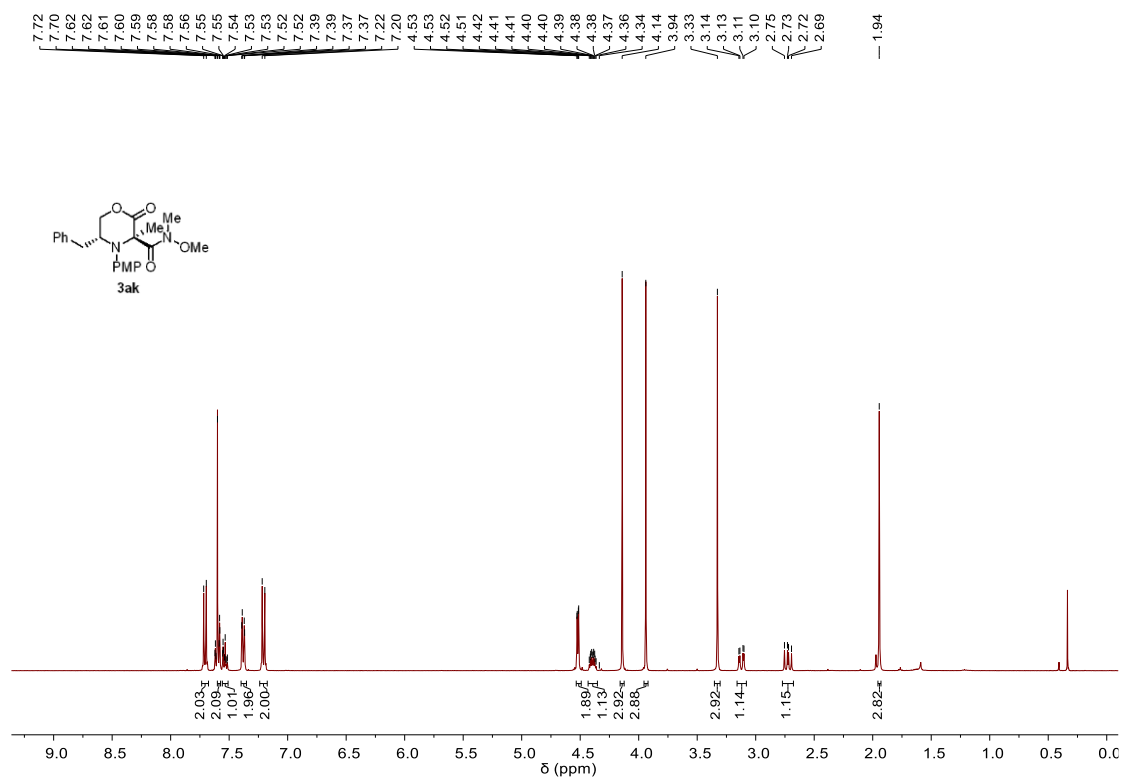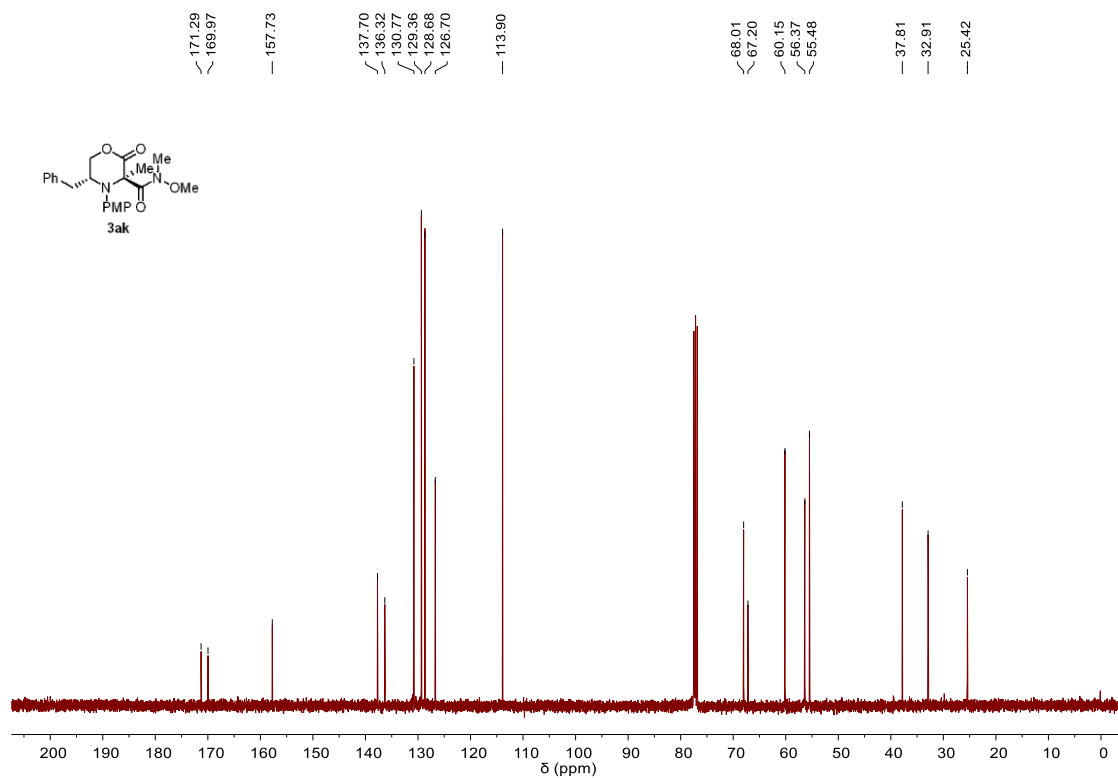

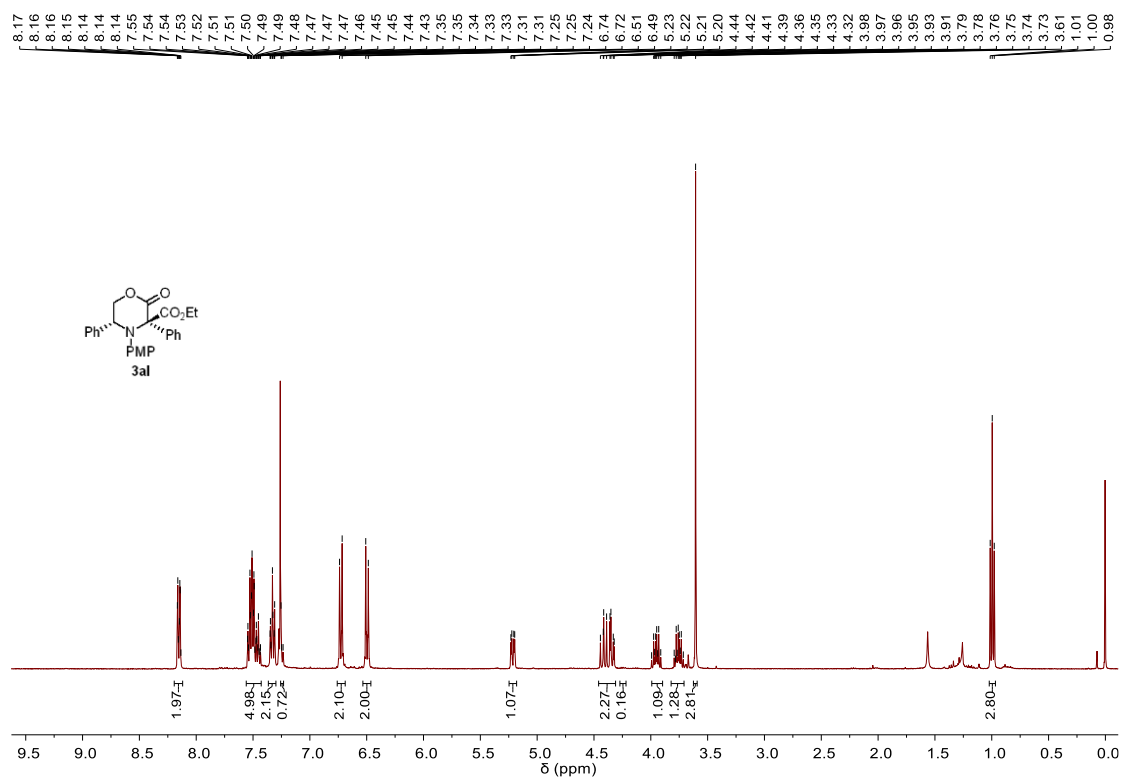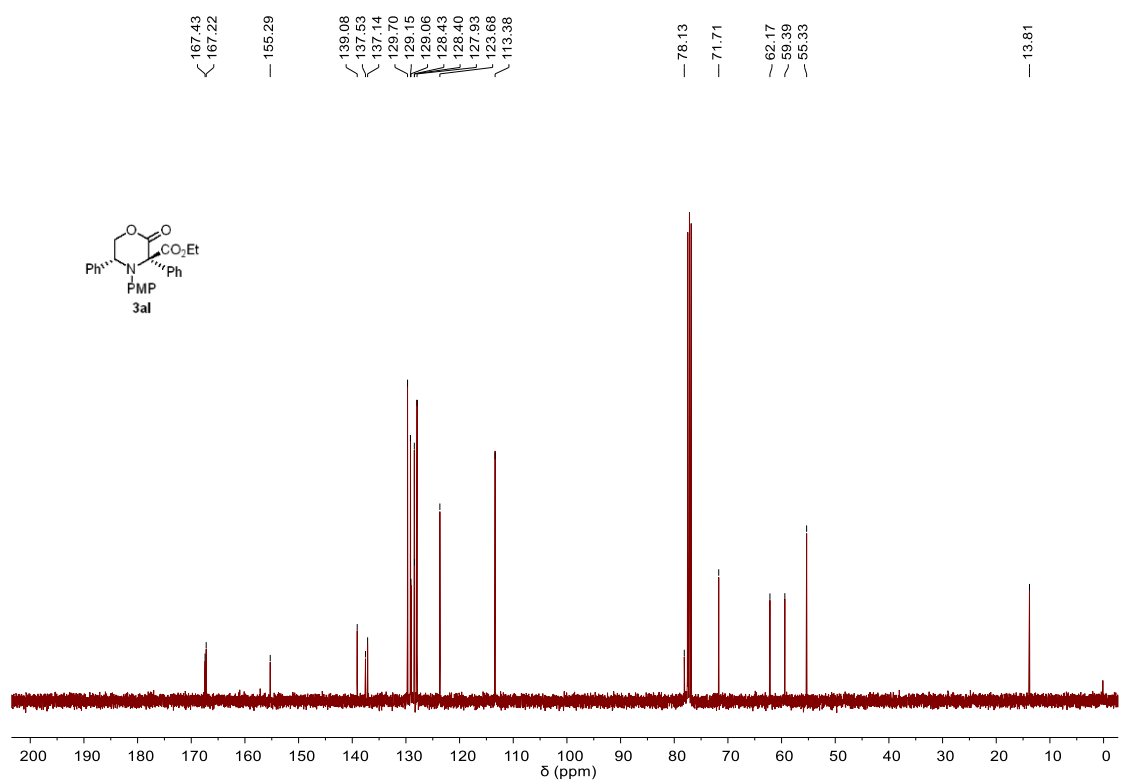

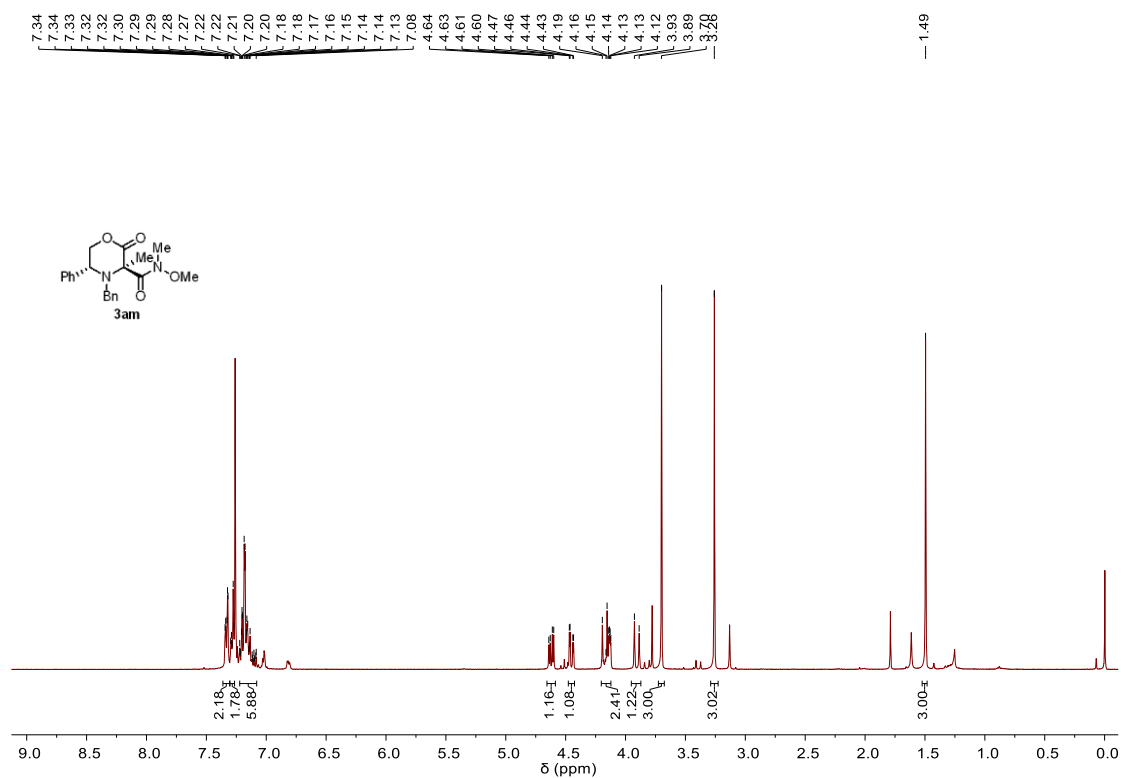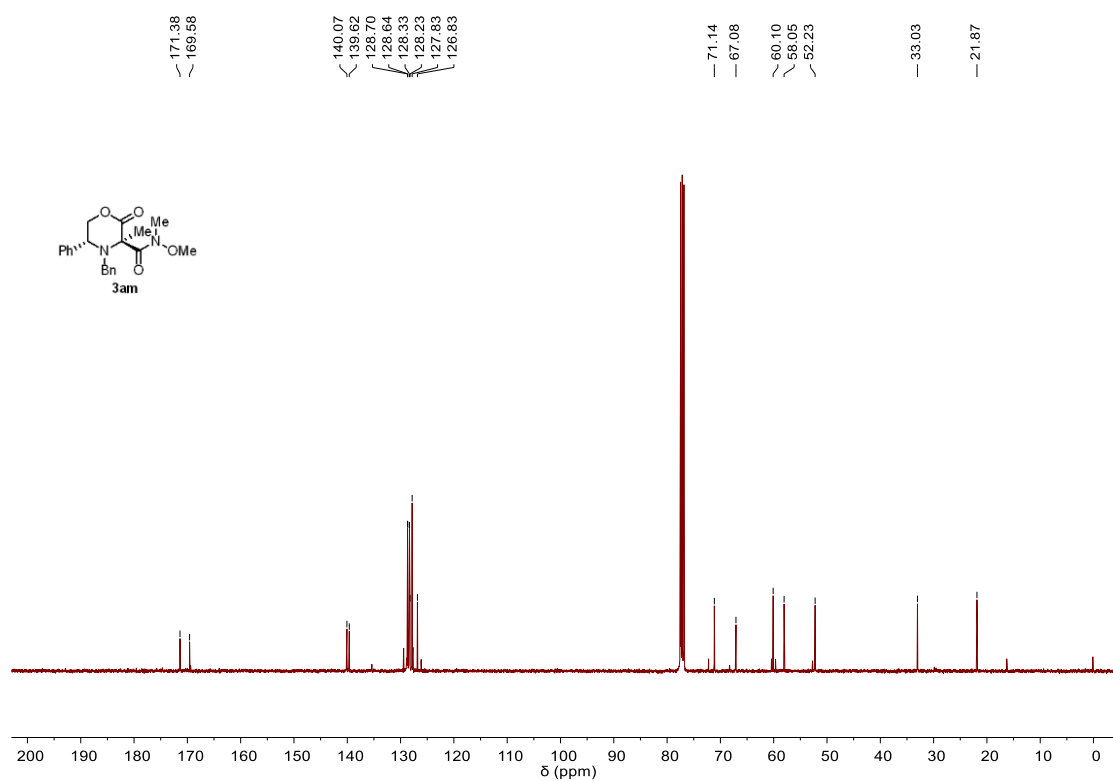

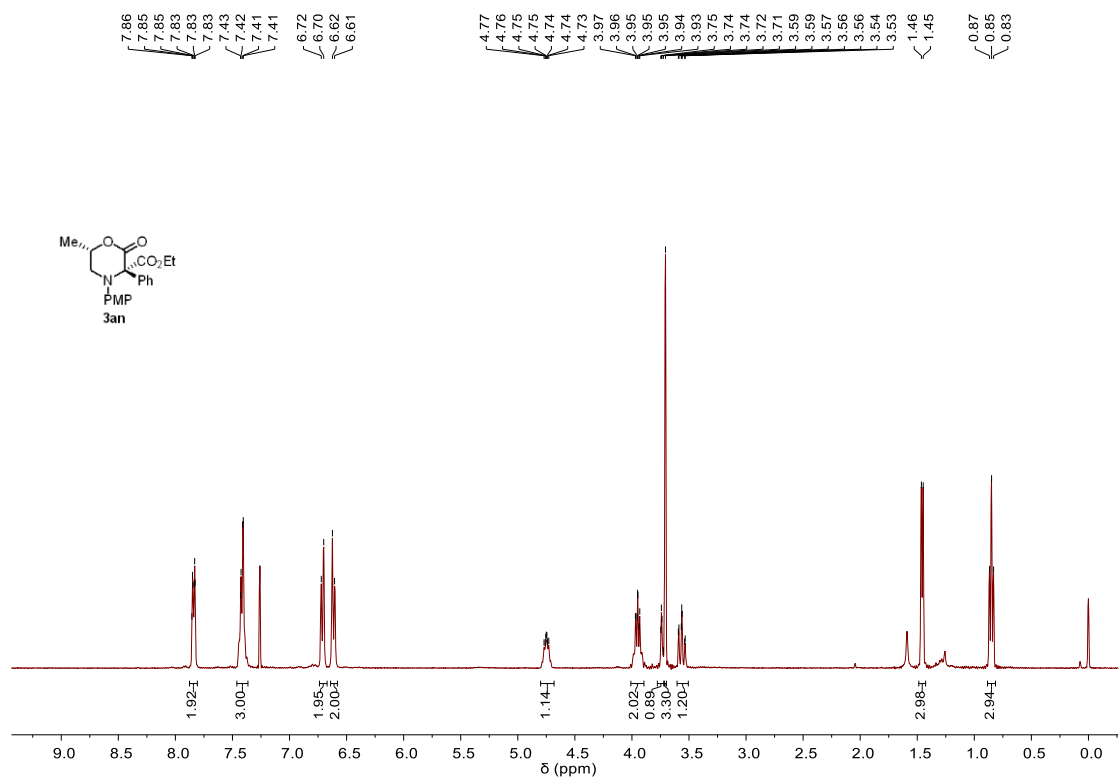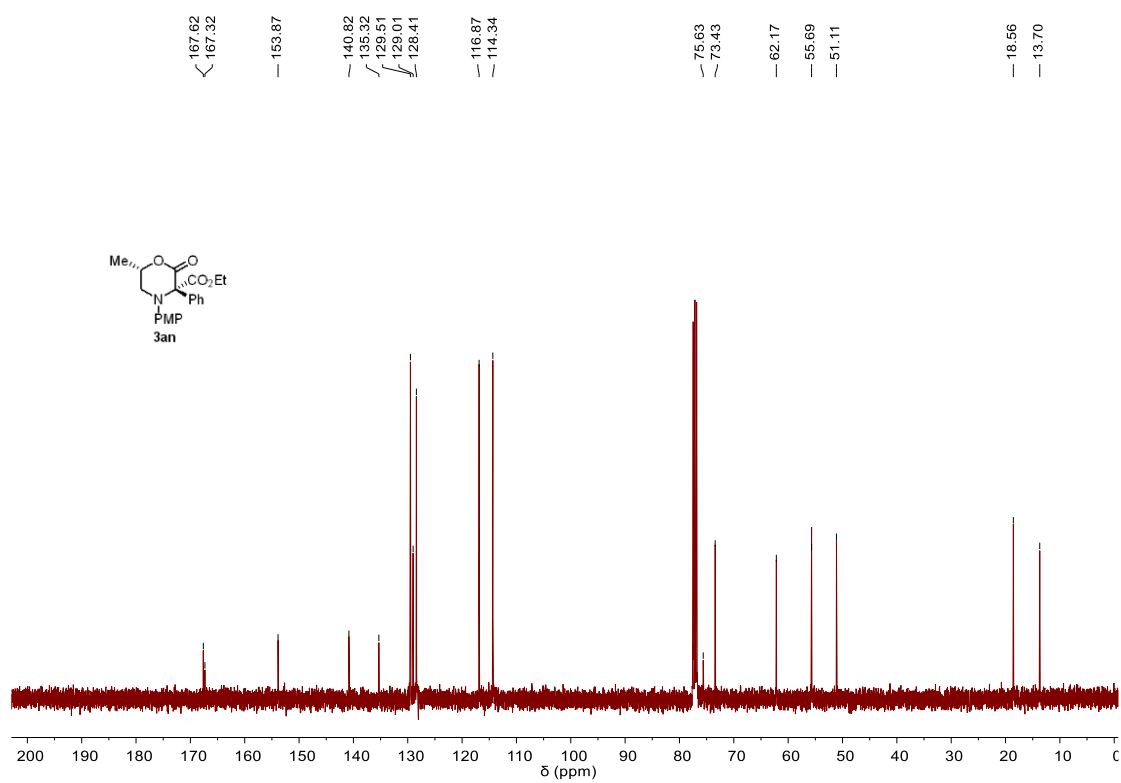

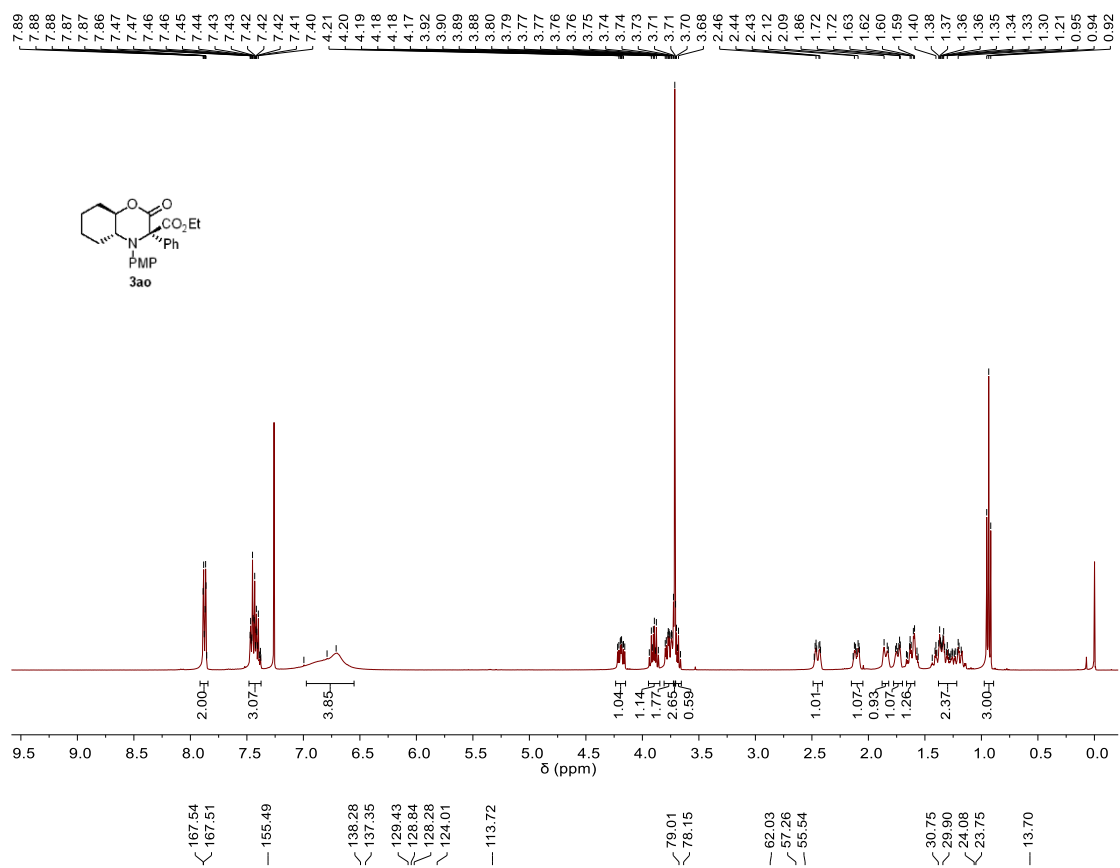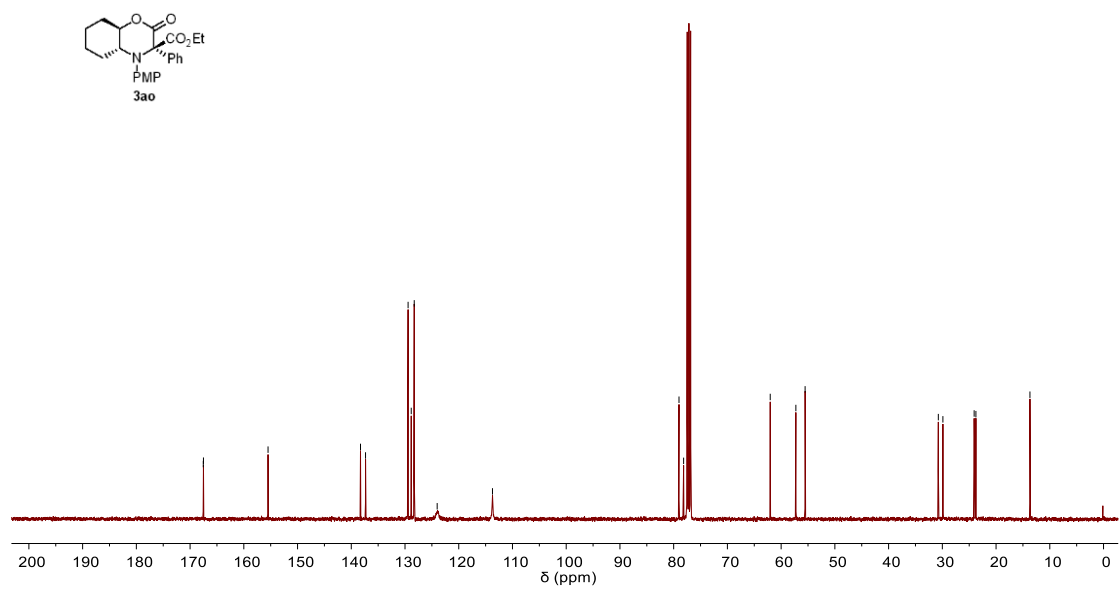

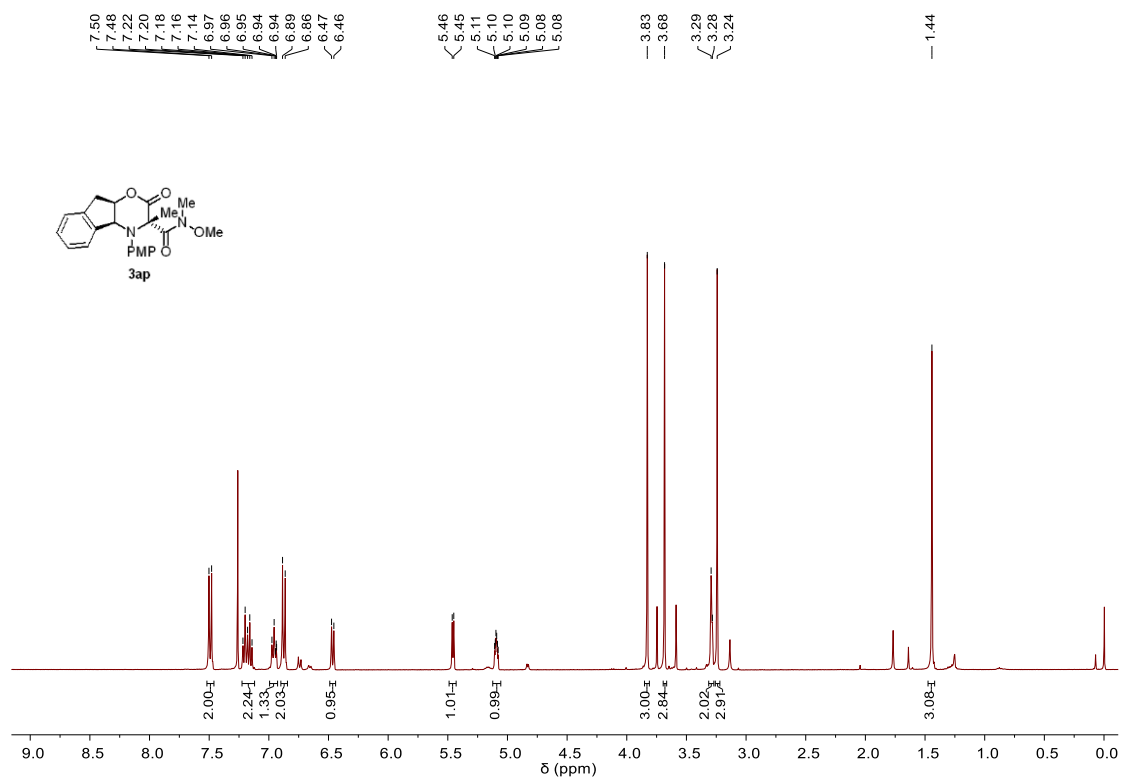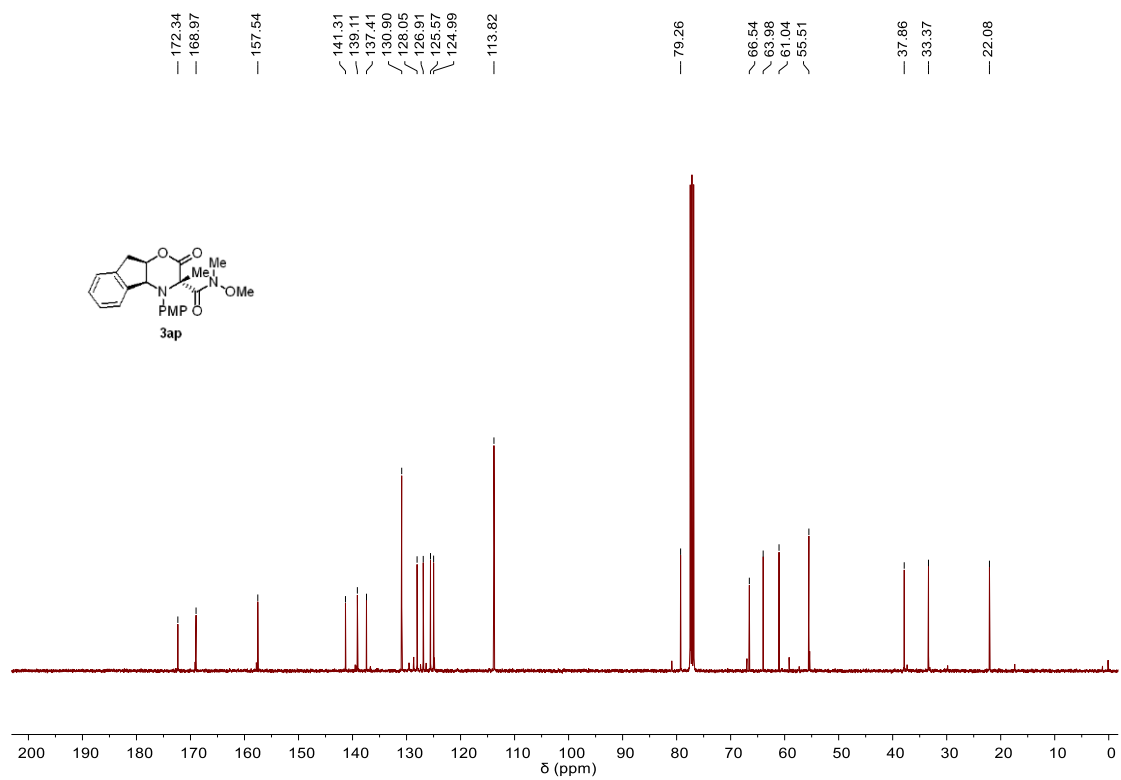

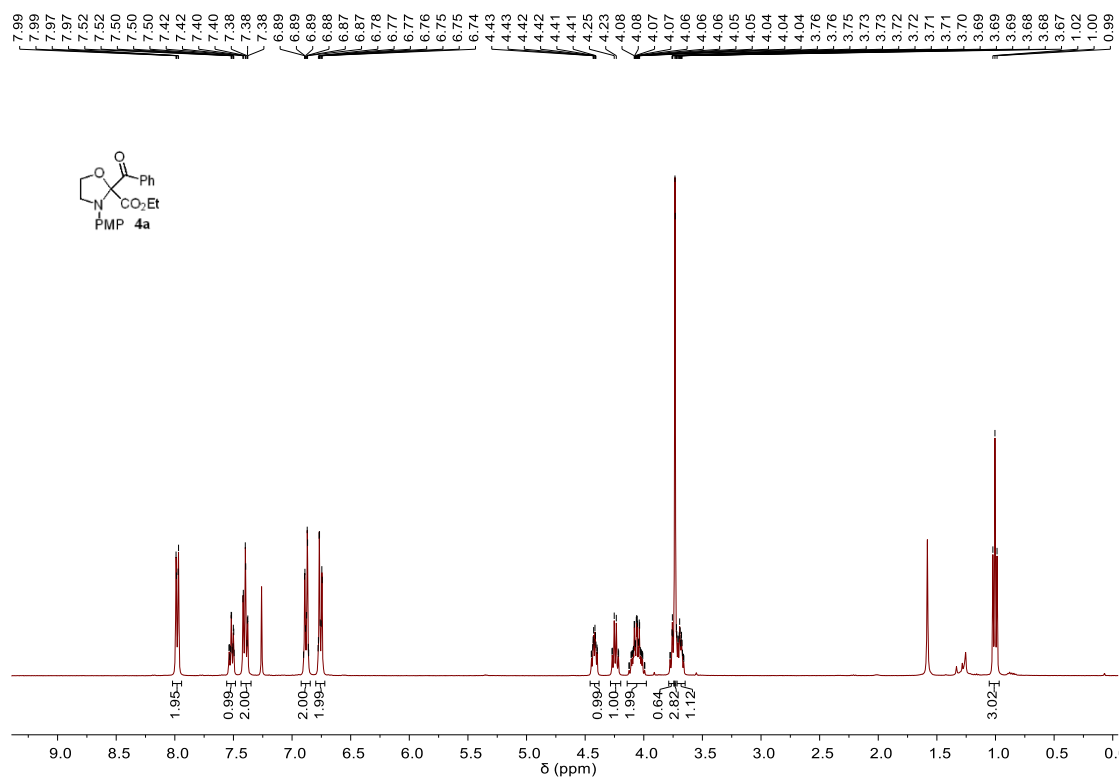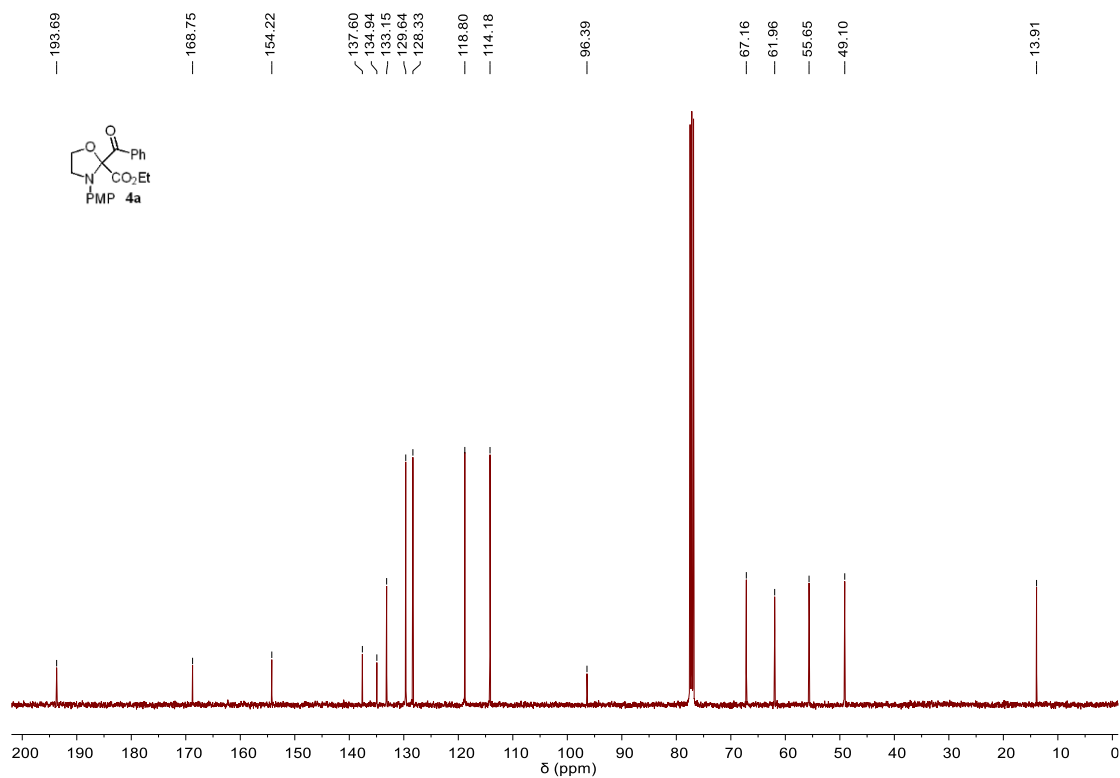

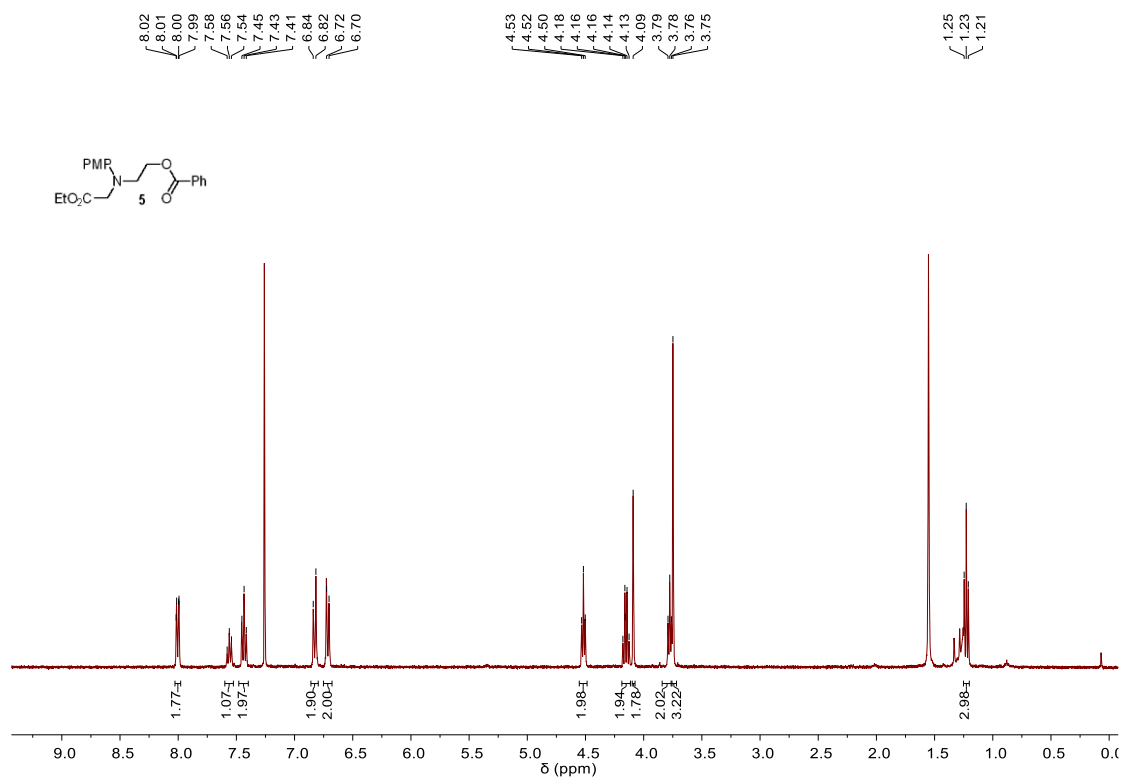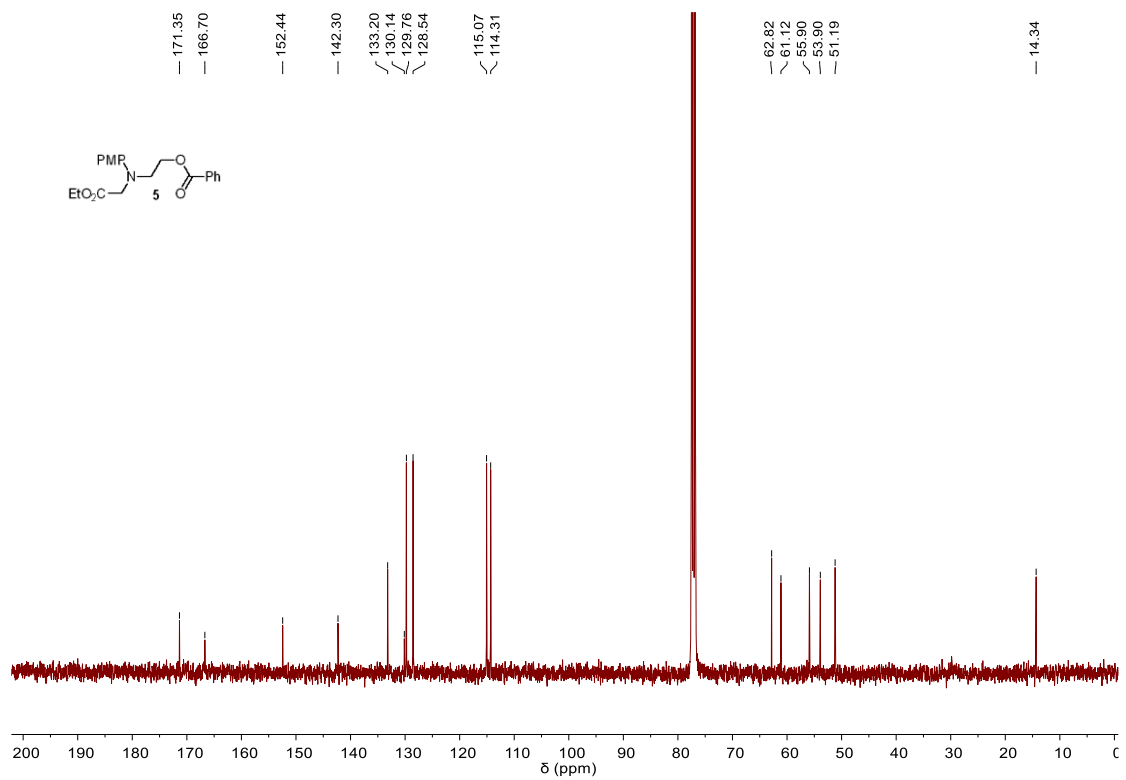

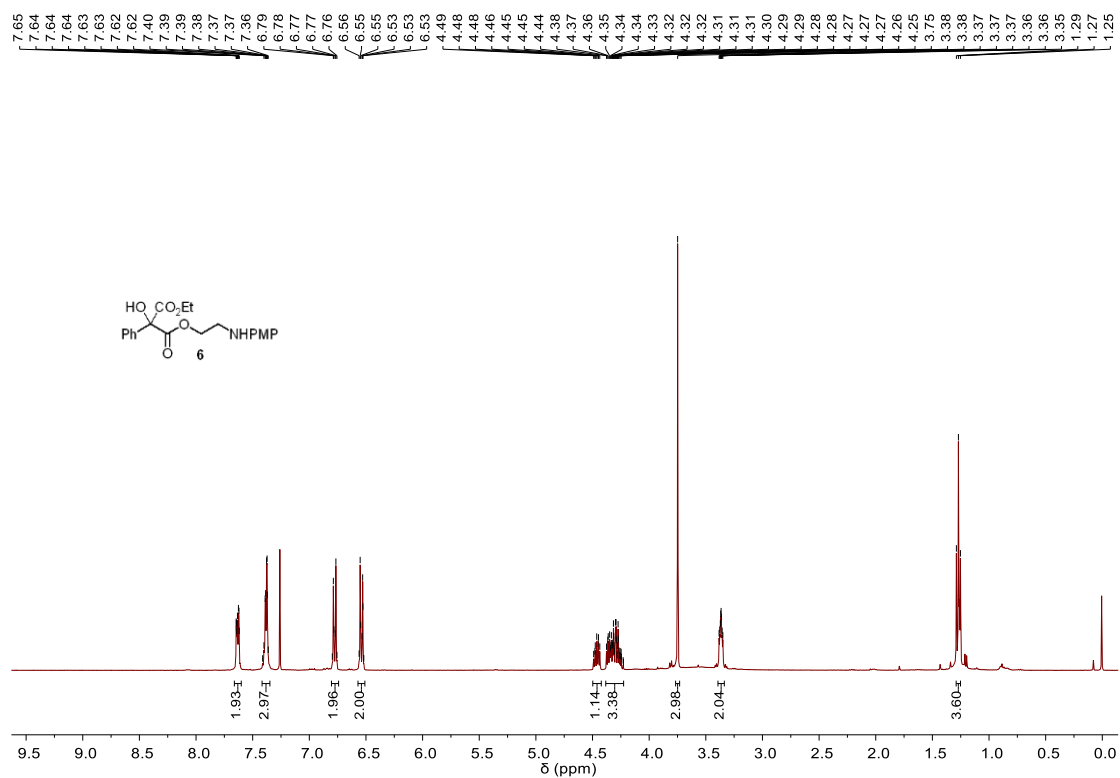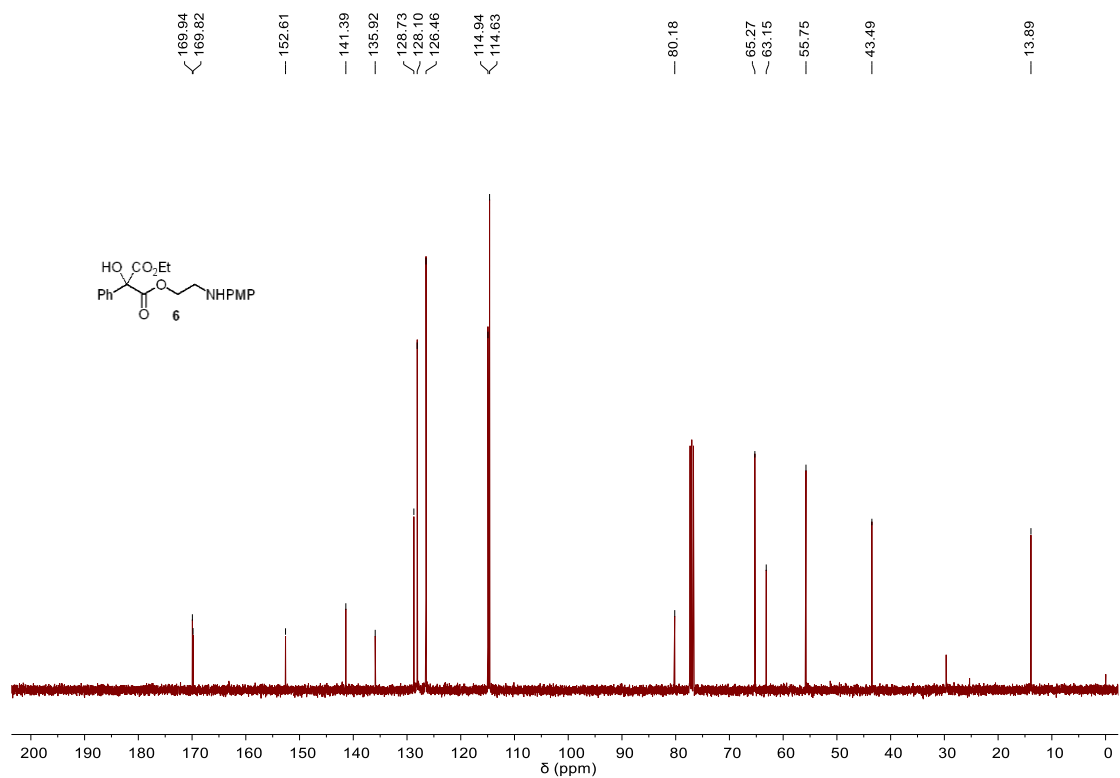

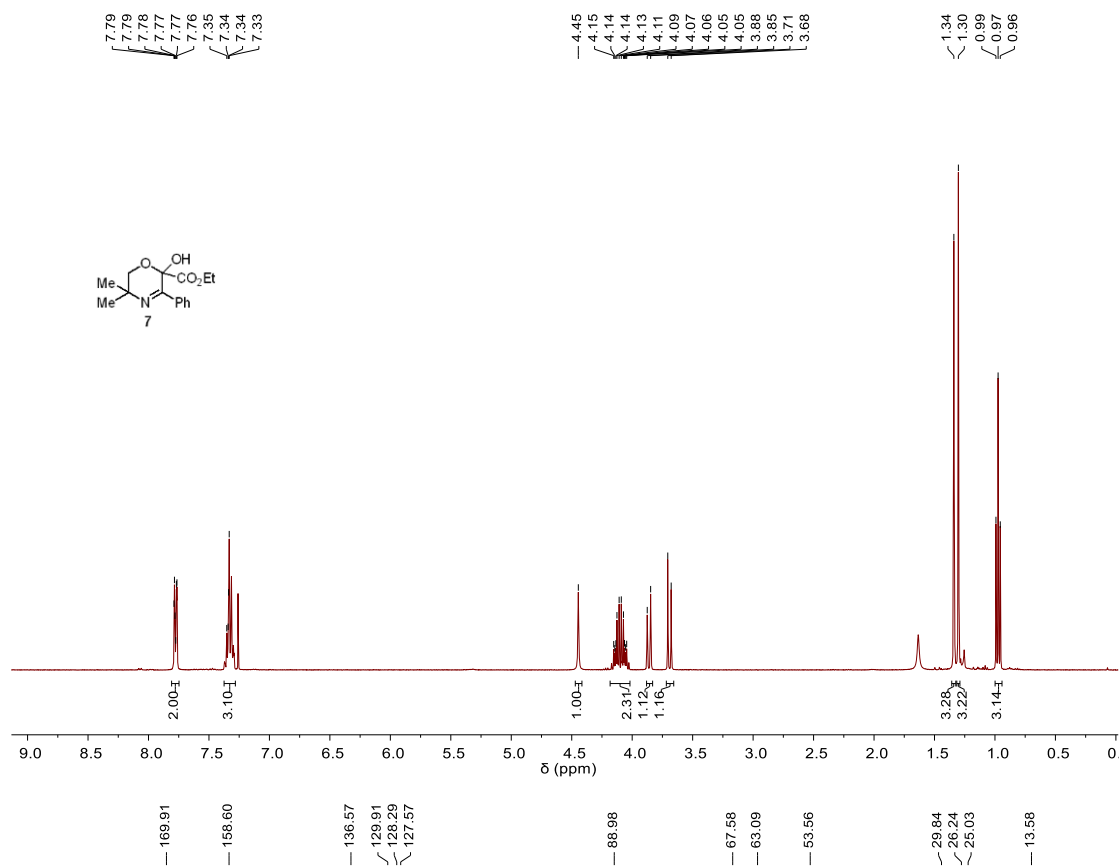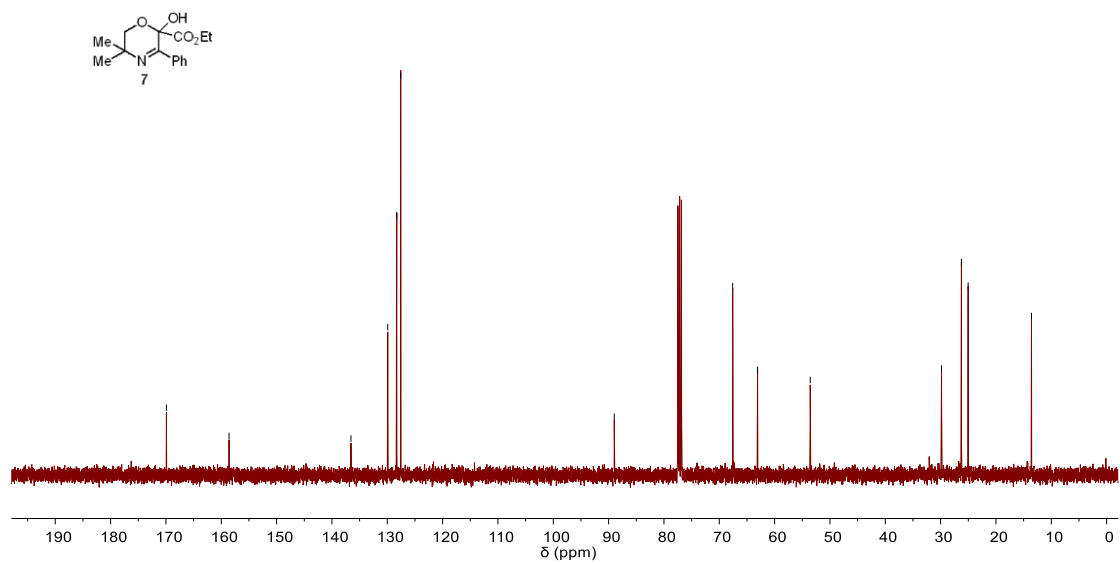

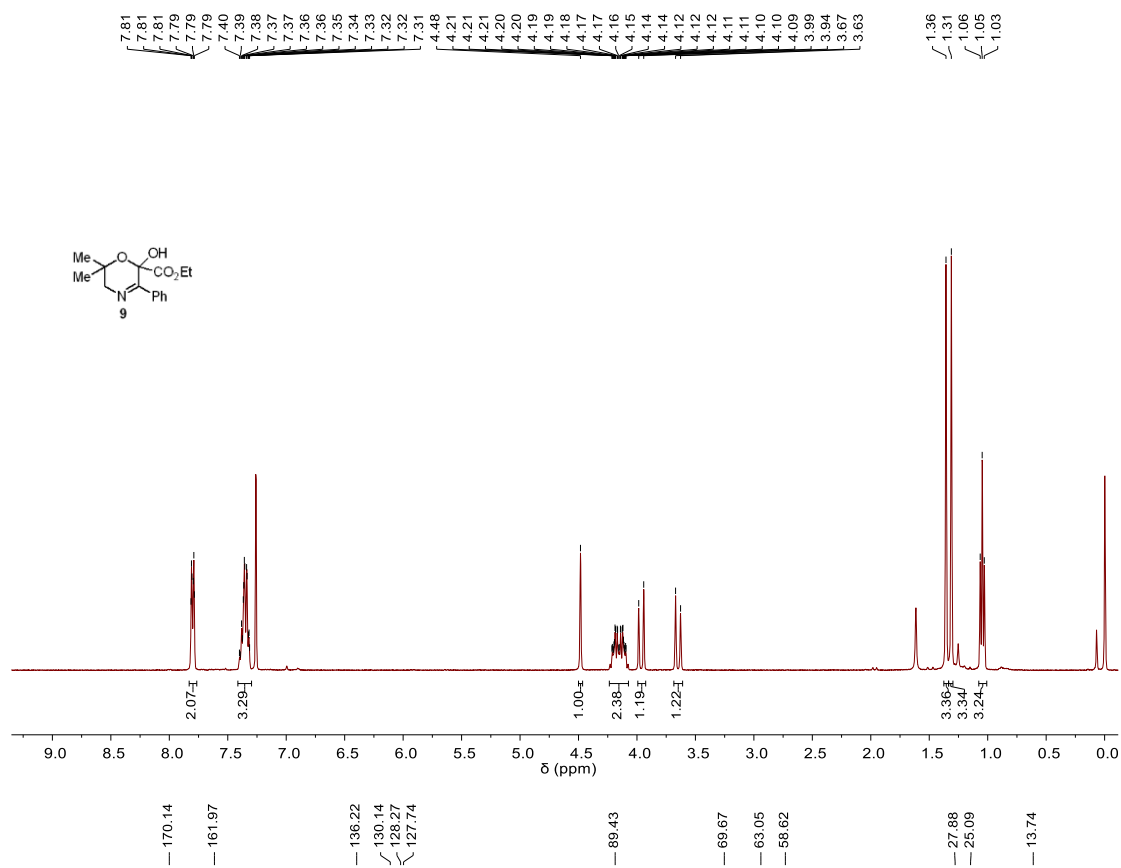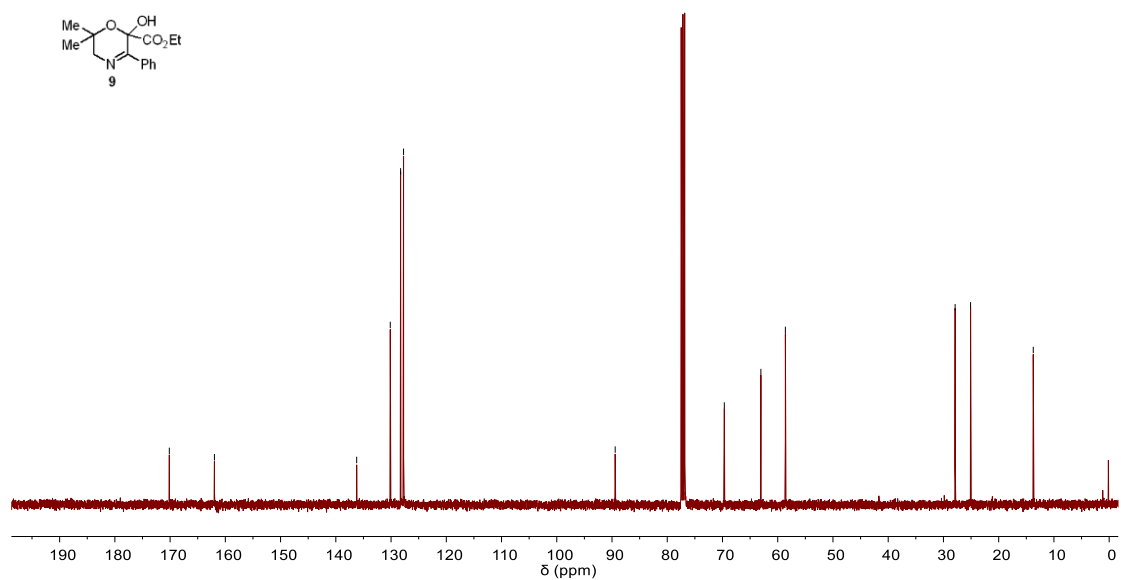

Supplement: Supplementary file 9 — Supplementary Data 7 [file 42004_2023_1016_MOESM9_ESM.pdf]
